# In-situ δ^18^O and ^87^Sr/^86^Sr proxies in an unconformable clastic unit at the Ordovician–Silurian transition

**DOI:** 10.1038/s41598-023-42200-3

**Published:** 2023-09-13

**Authors:** Chaewon Park, Yungoo Song, Namsoo Kim, Sung-Ja Choi, Ueechan Chwae, Yirang Jang, Sanghoon Kwon, Jeongmin Kim, Ha Kim, Youn-Joong Jeong

**Affiliations:** 1 Department of Earth System Sciences, Yonsei University, Seoul, Republic of Korea; 2 Korea Institute of Geoscience and Mineral Resources (KIGAM), Daejeon, Republic of Korea; 3 Department of Earth and Environmental Sciences, Chonnam National University, Gwangju, Republic of Korea; 4 Korea Basic Science Institute (KBSI), Cheongju, Republic of Korea

**Keywords:** Geochemistry, Geology, Mineralogy, Petrology, Sedimentology, Tectonics

## Abstract

Clastic successions found in the carbonate platform of continental margin during the Ordovician–Silurian Transition (OST) period are archives for interpreting paleo-depositional systems. Here, we report in-situ δ^18^O_quartz_ and ^87^Sr/^86^Sr_carbonate_ isotope chemo-stratigraphy for an unconformable clastic unit from the Cathaysia terrane that rifted off the Gondwana Supercontinent in the Early Paleozoic Era. Our results suggest a depositional proxy and model for geological events attributed to rapid changes in the sedimentary environment during the OST period. Importantly, these results present crucial clues that infer the influence of Paleo-Tethys Sea opening, global eustatic regression, and rapid sedimentary provenance change. Our study provides insight into paleo-tracer that could be a key method for interpreting depositional system of carbonate platform based on in-situ mineral isotope chemo-stratigraphy that preserves the original value of provenance and geochemical condition.

## Introduction

**Figure 1:**
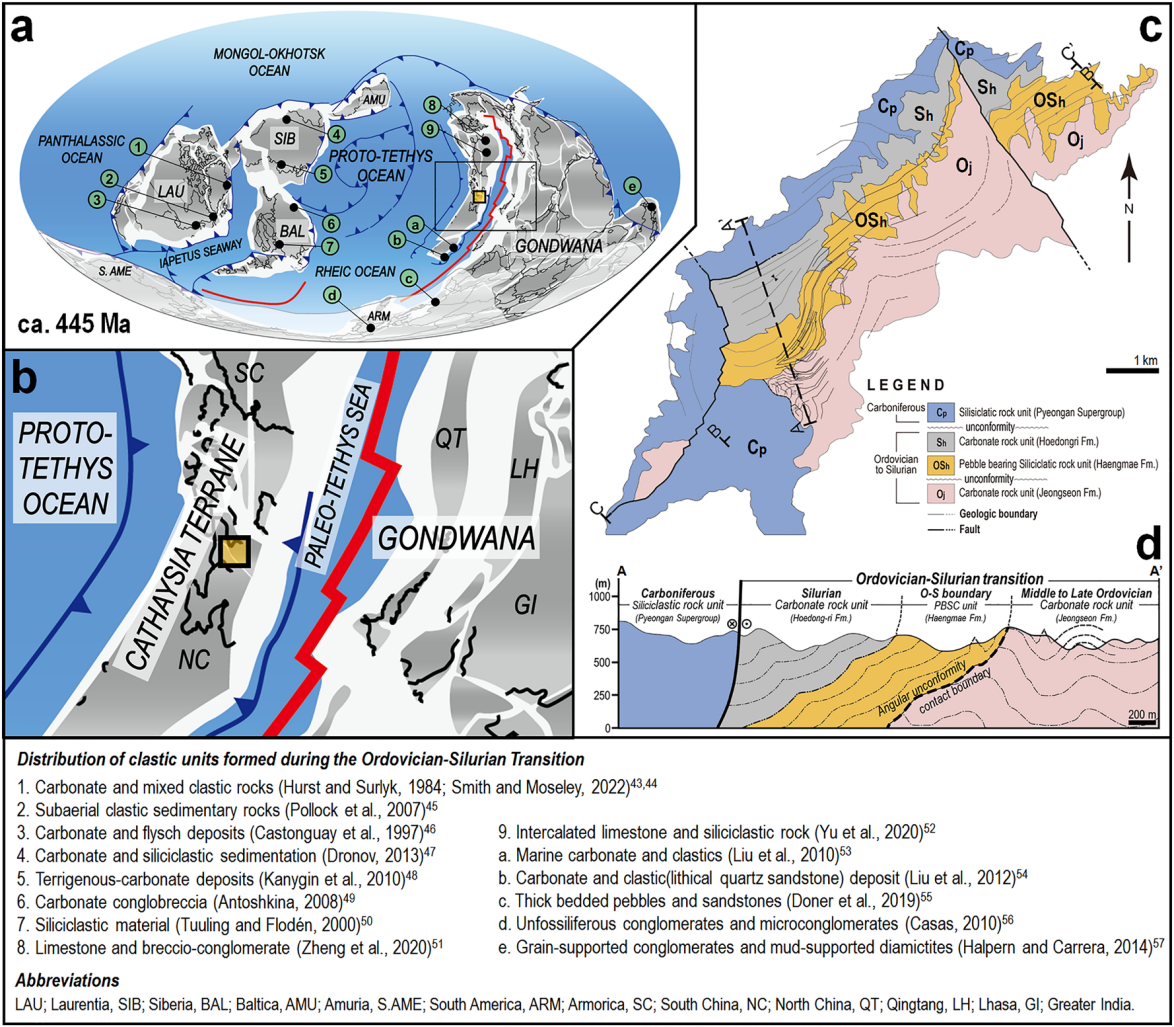
Global creation of clastic units during the Ordovician–Silurian Transition (OST). (**a**) Reconstruction of a global paleogeographic map (ca. 445 Ma) (modified after refs.^7–9^) showing the distribution of clastic O-S boundary strata (Supplementary Table 1). (**b**) Cathaysia terrane’s breaking-off from the Gondwana and subsequent Paleo-Tethys Sea opening. Yellow solid square indicates the study area. (**c**) Simplified geologic map of the study area showing the distribution of clastic succession. The target unit is a pebble-bearing fine sand-sized clastic rock (PBSC) unit, which is in unconformable contact with the underlying carbonate unit. (**d**) Interpretative A-A’ cross-section of the study area. It shows that the PBSC and the underlying carbonate units are unconformable. Figures were drawn using Adobe Illustrator CS6 version 16.0.0 (https://www.adobe.com/) and Microsoft PowerPoint Office 365 ProPlus version (https://www.microsoft365.com/).

The Ordovician–Silurian transition (OST) is a period of global upheaval. Above all, a big biota mass extinction event occurred that wiped out up to 60% of the marine genera^1^. One of the main factors of global environmental change that led to this cataclysm was the global glaciation that started in Antarctica and reached its maximum during the Hirnantian stage of the Latest Ordovician^2–6^. This event caused major changes not only in the biosphere but also in the lithosphere. In particular, the global eustatic regression caused by this glaciation greatly increased the continental free board in Gondwana Supercontinent, Baltica, Laurentia, and Siberia^7,8^, which may have resulted in a depositional transition due to rapid change in the sedimentary environment and the provenance. The clastic successions of OST found between marine sedimentary sequences in the margins of each continent may be a geological proxy related to these changes (Fig. 1a).

The OST carbonate platform found in each continent’s marginal environment is a key archive of a paleo-depositional system with a record of geological changes (Fig. 1a). The characteristics of each regional OST clastic unit are summarized as follows (Supplementary Table 1): (1) a wide range of terrigenous lithofacies, from conglomerate to sandstone mixed with carbonate rock in clastic sequences, (2) in general, unconformably overlying the carbonate rock unit that was formed in a marginal shallow marine environment, and (3) conformably underlying another carbonate rock unit. Here, it suggests that two main factors may have contributed: (1) eustatic regression by global glaciation in the Late Ordovician period would have contributed to their creation process, and (2) regional increases in the continental free board would have derived from uplifting by tectonic events.

One of the key tectonic events of the OST period was the Cathaysia terrane’s breaking-off from the Gondwana and the subsequent Paleo-Tethys Sea opening^7–9^ (Fig. 1b). This would have provided a compressive stress field on the eastern margin of the Cathaysia terrane resulting in tectonic uplifting and the subsequent increase of continental freeboard. A unique clastic unit formed during the OST period was also found in this area (Fig. 1b and c). It is highly likely that this clastic unit was affected by this tectonic event along with worldwide glaciation during its creation.

Despite previous studies on the clastic successions of the OST period (Fig. 1a), the results based on descriptive approaches did not provide any direct evidence of a change in the provenance of these clastic units. A number of studies through stable isotope analysis have also been attempted^10–14^, but there were difficulties in separating the target mineral from the whole rock sample and overcoming the limitations of the resulting analysis precision and accuracy. The recent progress of in-situ technique has been able to determine the isotopes for several tens of micro-sized minerals in sedimentary rock, while preserving the micro-texture. Since the stable isotopic feature of minerals reflects the chemical conditions of their formation, the in-situ isotopic analyses of minerals can precisely identify some changes in their provenance. In this study, we studied a carbonate platform developed along the margin of a Cathaysia terrane rifted off the Gondwana continent (Fig. 1c and d). We firstly conducted in-situ δ^18^O_quartz_ and ^87^Sr/^86^Sr_carbonate_ isotope chemo-stratigraphy on clastic mineral (quartz) and carbonate minerals (calcite and dolomite) in the sedimentary succession of the OST period. Through this, we tried to find out whether the in-situ isotope chemo-stratigraphic proxy indicates the change in the provenance of detrital materials in the clastic unit, and furthermore, whether this becomes a decisive clue to infer the influence of global eustatic regression and Paleo-Tethys Sea opening during the OST period.

## Results

### An unconformable clastic unit in Gondwana

The study target is a unique clastic unit of the OST period appearing among sedimentary successions of carbonate rocks in the Korean Peninsula. This area was located in the Cathaysia terrane, the northwestern margin of the Gondwana during the Early Paleozoic Era (Fig. 1a and b). It is currently located on the eastern margin of the North China Block (Sino-Korean Block)^15,16^ (Supplementary Fig. 1a). In the previous study, the authors reported the distribution of this target unit through precise mapping^17^. A revised version of the geological map is presented (Fig. 1c; Supplementary Fig. 1).

**Figure 2:**
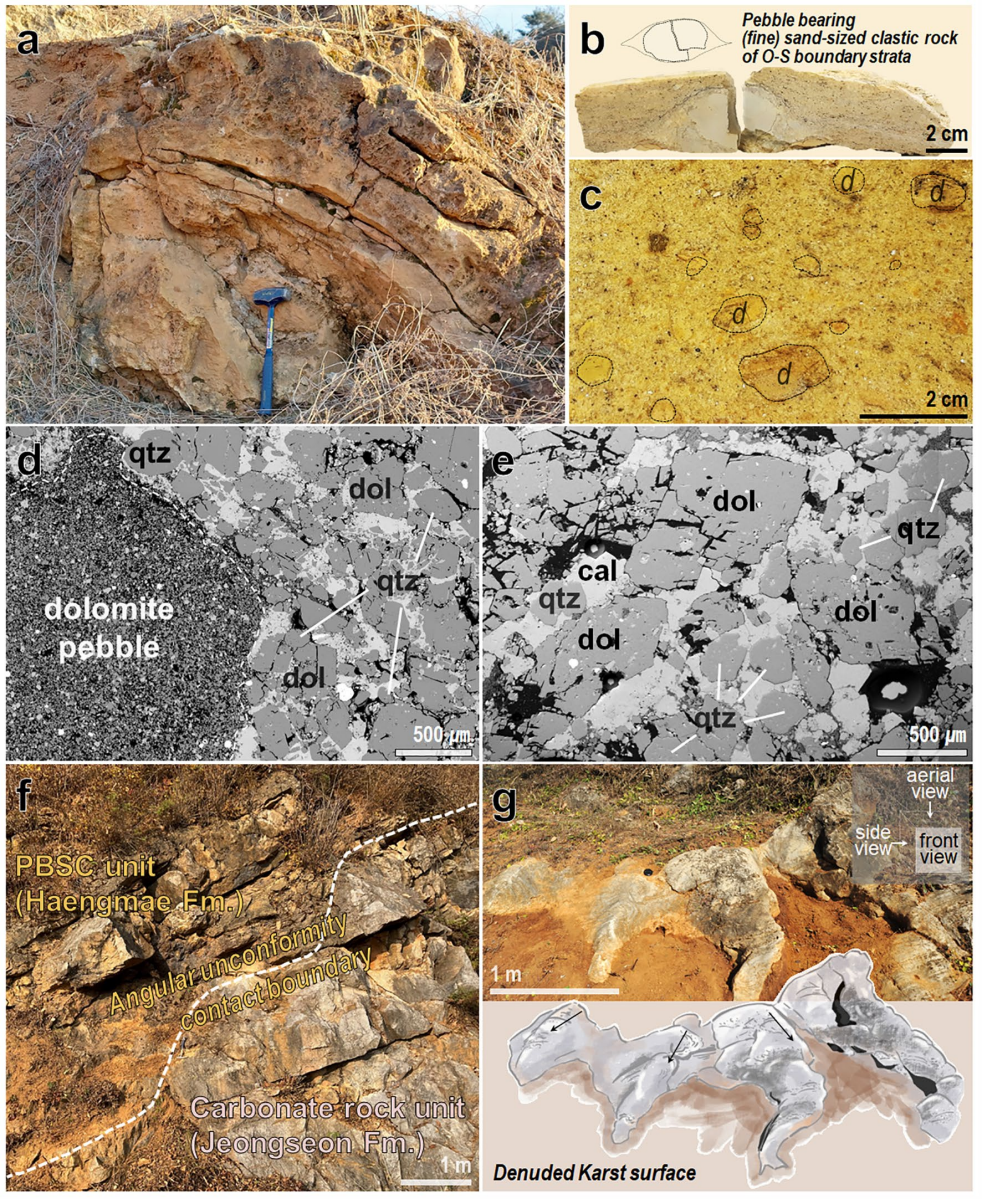
Compilation of outcrop, lithological, and micro-textural characteristics of the PBSC and carbonate rock units. (**a**) Photograph of a yellowish-brown PBSC unit (Haengmae Fm.) outcrop. (**b**) Polished slab of elongated dolomite pebble (aggregates of fine-grained dolomite with 10–20 μm in size) by shearing in the PBSC unit. (**c**) Polished slab showing the macro-textural characteristics of the PBSC unit containing pebbles of various rock types, including dolomite pebbles ‘*d*’ ranging in size from a few mm to several cm. (**d**–**e**) SEM-BSE image showing the micro-textural features of the PBSC unit. It contains dolomite pebble of several millimeters or more, fine sand-sized quartz (qtz) and dolomite (dol) single grains, and calcite cement that fill the pore space. (**f**) Outcrop showing the angular unconformity contact boundary between the PBSC and the lower carbonate units. (**g**) The denuded `Karst’ surface and its sketch at the uppermost subsurface of the carbonate unit (Jeongseon Fm.) below the PBSC unit.

The clastic unit, the so-called Haengmae Formation, is a unique pebble-bearing fine sand-sized clastic rock (PBSC) unit with a layer thickness of about 100 m, which is vulnerable to weathering and forms a gentle topography (Figs. 1c, d, 2a, and b). The PBSC unit covers the lower carbonate unit (Jeongseon Fm.), which is mainly composed of gray limestone and is covered by the upper carbonate unit (Hoedongri Fm.), which mainly consists of packstone and wackestone^17^. The PBSC unit generally maintains low-angle bedding with an inclination of less than 15°. Although some folding structures appear, overlapping folding is not observed in the PBSC unit^17^ (Fig. 2a; Supplementary Fig. 2).

In contrast, the bedding plane of the lower carbonate unit shows a moderately inclined slope, and its trajectory shows an S-shaped overlapping fold (F2)^17^ (Supplementary Fig. 3). This indicates that this unit has undergone at least two-time deformation events. The distinct difference in deformation structure characteristics between the PBSC and the lower carbonate units can be reasonably inferred that there was a large temporal gap between the two strata. In this study, we found several new evidences in the outcrop to confirm the above inference. Above all, the angular unconformity contact between the PBSC and the lower carbonate units was first discovered in the outcrop (Fig. 2f). In addition, the ‘Karst’ surface was found on the uppermost subsurface of the lower carbonate rock unit, which is directly connected to the PBSC unit (Fig. 2g; Supplementary Fig. 4). This is a clear geological signature indicating that the carbonate unit was exposed and denuded on the surface by uplifting through deformation after deposition.

### Age constraints for PBSC unit

The deposition period of the PBSC unit could be inferred through U–Pb age dating of the detrital zircon. The results of this study and previous studies are summarized in Supplementary Table 5. Jang^18^ reported 449.5 ± 1.5 Ma (n = 18) with high reliability as the maximum deposition age of the PBSC unit. The frequency graph (Supplementary Fig. 7) combining data from this study and previous studies^17,18^ strongly indicates that the PBSC unit was deposited during at least the Late Ordovician or Early Silurian period, that is, the OST period.

### Macro-/micro-textural features of PBSC unit

The PBSC unit shows the macro-textural characteristics of a typical clastic rock with a granular matrix containing detrital pebbles of various rock types, including dolomite pebbles ranging in size from several mm to several cm^17^ (Fig. 2b and c; Supplementary Fig. 5). Dolomite pebbles with gray-brown to yellow–brown fine-grained (10–20 μm) aggregates are predominant in the pebbles. Small amounts of pebbles of micritic mudstone, quartzite, sandstone, and phyllite are included, and white phengite series aggregates ranging in size from several mm to several cm also appear^19^. The PBSC unit was partially deformed, and some dolomite pebbles were elongated, showing a shearing sense (Fig. 2b; Supplementary Fig. 5).

The PBSC unit matrix confirmed by XRD and SEM-BSE observation consists of quartz, single-crystalline dolomite, calcite cement, polycrystalline phengite series aggregates, and rock fragments with a size of less than 2 mm (Fig. 2d and e; Supplementary Fig. 5 and Table 2). In our previous micro-textural study, we defined only clastic quartz and rock fragments as sediments in the syn-depositional stage among the constituents of the PBSC unit matrix, and the other minerals as secondary phases formed in the post-depositional stage^17,19^. Based on these mineral compositions and micro-texture characteristics, the PBSC unit is inherently a typical clastic rock unit and appears to have undergone several post-depositional events.

### In-situ δ^18^O_quartz_ and ^87^Sr/^86^Sr_carbonate_ chemo-stratigraphic proxies

**Figure 3:**
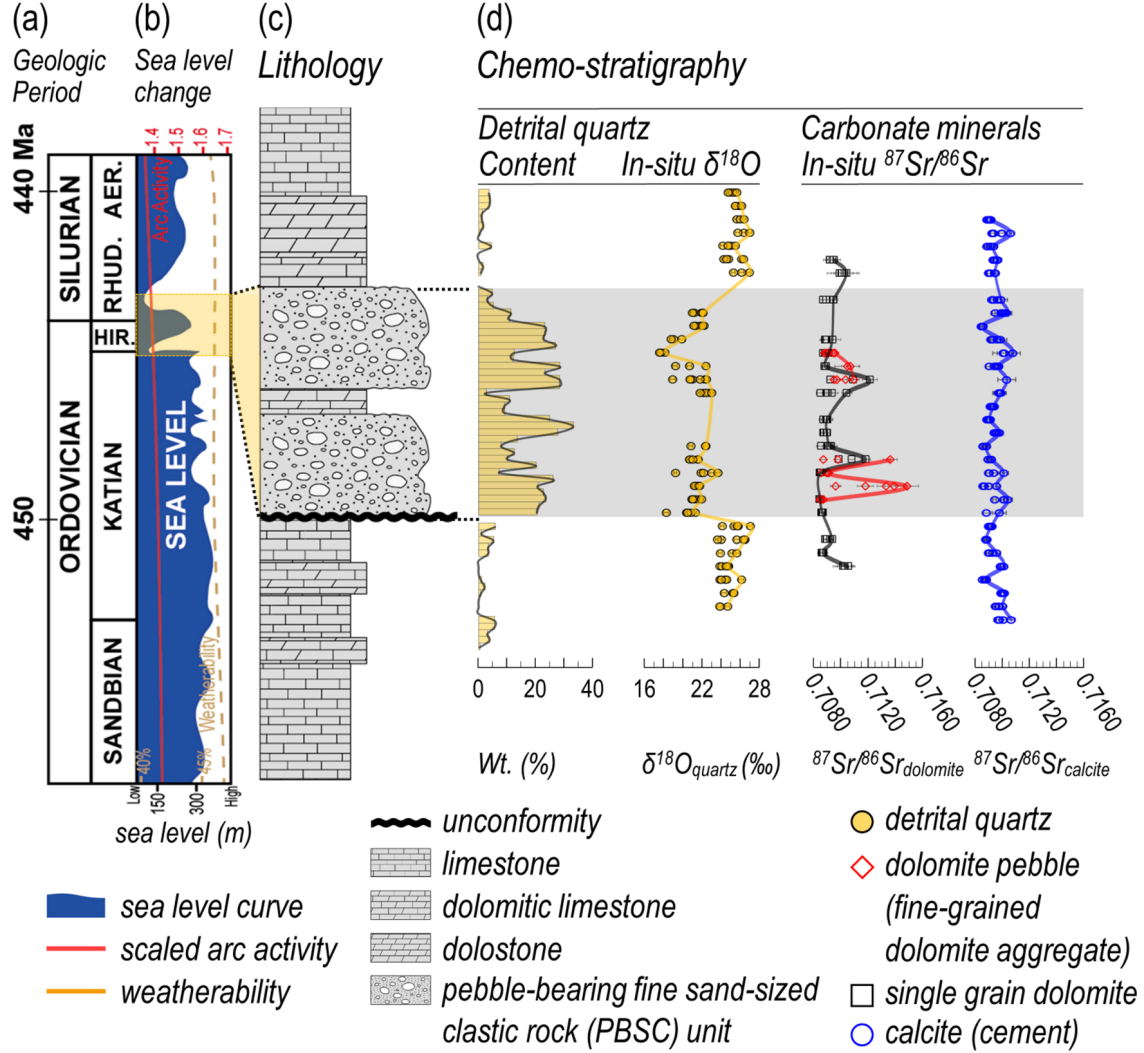
High-resolution in-situ δ^18^O_quartz_ and ^87^Sr/^86^Sr_carbonate_ chemo-stratigraphy at OST. Diagram including geologic time scale (**a**), Late Ordovician to Early Silurian (Llandovery) sea level fluctuations (blue shaded area) (**b**) with related arc activity (red solid line) and weatherability (yellow dotted line)(adapted from refs^9,58–60^), columnar section showing the lithology of the target area (**c**), and plots showing in-situ δ^18^O_quartz_ and ^87^Sr/^86^Sr_carbonate_ chemo-stratigraphic results with detrital quartz contents (**d**), in which lines connect the maximum values of each point and error bars (2σ) are specified. The yellow-shaded area is the Ordovician–Silurian transition (OST) zone based on U-Pb zircon age dating. The gray shaded area is the chemo-stratigraphy for the PBSC unit. In the PBSC unit, an abrupt drop in in-situ δ^18^O_quartz_ values accompanied by a sharp increase in detrital quartz content, and a rapid change in in-situ ^87^Sr/^86^Sr_dolomite_ values in the clastic dolomite pebble (fine-grained dolomite aggregate) were observed.

We conducted in-situ isotope chemo-stratigraphic studies on the PBSC unit and two carbonate rock units (Fig. 3; Supplementary Tables 3 and 4).

*Quartz* Based on SEM-BSE image observation, quartz, which is the target of in-situ δ^18^O analysis, shows micro-textural characteristics of typical clasts such as various grain sizes, poor sorting, and subangular shape in both PBSC (Supplementary Fig. 5) and carbonate (Supplementary Fig. 6) units. In particular, the quartz content increases rapidly in the PBSC unit, which clearly shows that there was an influx of a large amount of terrestrial clasts (Fig. 3). In in-situ oxygen isotope analysis of detrital quartz, the δ^18^O_quartz_ values (17.55 ~ 23.65 ‰) in the PBSC unit is significantly lower than those of the two carbonate units (23.56 ~ 27.01 ‰) (Fig. 3). Since quartz is resistant to secondary alteration^10,20^, its primary isotope values can be preserved and used as a paleo-tracer for provenance interpretation. Therefore, the sharp drop of δ^18^O_quartz_ values in the PBSC unit strongly indicates that the provenance of quartz in this unit was clearly changed.

*Carbonates* The in-situ ^87^Sr/^86^Sr isotope values of carbonate minerals reflect the geochemical conditions of the syn-, or post-depositional stage^21–24^. In the PBSC unit, the in-situ ^87^Sr/^86^Sr_dolomite_ values of clastic dolomite pebble (fine-grained dolomite aggregate) fluctuate markedly from 0.7085 to over 0.7149 (Fig. 3). Considering that the carbonate successions in the study area were placed in the same geochemical environment after deposition, this suggests that the clastic dolomite pebbles were formed under geochemical conditions with distinct differences in ^87^Sr/^86^Sr isotope values, and its provenance was different from that of other carbonates.

**Figure 4:**
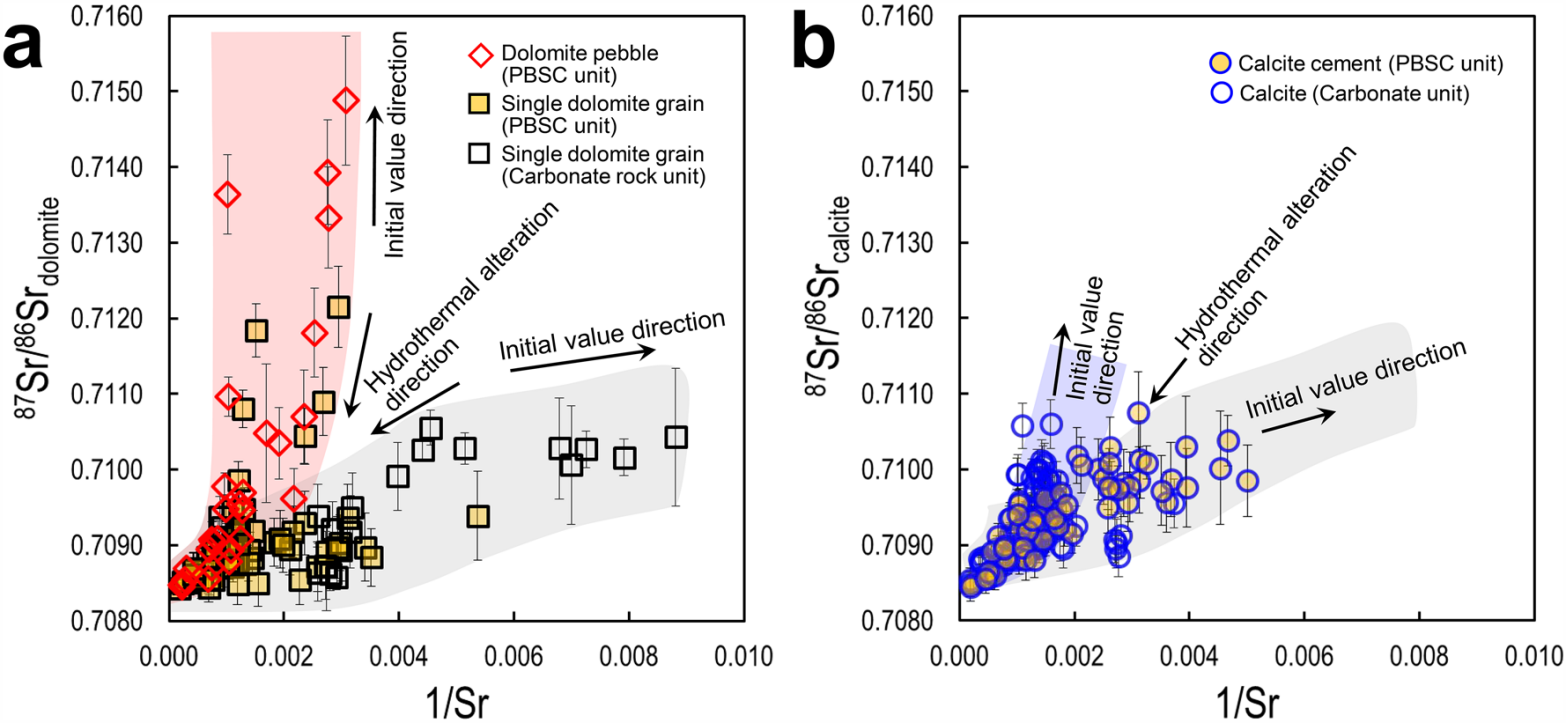
^87^Sr/^86^Sr_carbonate_ vs. 1/Sr correlation diagrams. (**a**) ^87^Sr/^86^Sr_dolomite_ versus 1/Sr correlation of PBSC and carbonate units. Each trend of clastic dolomite pebble and single dolomite of carbonate units show a distinctly different pathway, and the ^87^Sr/^86^Sr_dolomite_ values of the two trends converged to about 0.7084. (**b**) ^87^Sr/^86^Sr_calcite_ versus 1/Sr correlation of PBSC and carbonate units. The trend of all ^87^Sr/^86^Sr_calcite_ values also converged to about 0.7085. Each trend of calcite cement in PBSC unit and calcite in carbonate units show a slightly different pathway reflecting the difference in geochemical conditions at the time of creation. Error bars for each point represent 2σ.

In the ^87^Sr/^86^Sr_dolomite_ vs. 1/Sr correlation, the trend of all ^87^Sr/^86^Sr_dolomite_ values converged to about 0.7084 (Fig. 4a). This indicates that there was an effect of hydrothermal fluid after deposition^23^, and the creation of phengite series in the PBSC unit also supports it^17,19^. This trend of the clastic dolomite pebble shows a completely different pathway from the trend of the single dolomite in the upper and lower carbonate units (Fig. 4a). The ^87^Sr/^86^Sr_dolomite_ ranged from 0.7095 to 0.7106 according to the Sr variation of the carbonate rock unit (0.0008 to 0.0107). The dolomite pebble of the PBSC unit shows a large variation, with ^87^Sr/^86^Sr_dolomite_ ranging from 0.7085 to 0.7149 according to Sr variation (0.002–0.0031). This indicates that the clastic dolomite pebble was introduced from a provenance in which the initial ^87^Sr/^86^Sr_dolomite_ values was distinctly different, and was subsequently reset due to the influence of post-depositional hydrothermal influx.

In the calcite cement of the PBSC unit and calcite of the upper and lower carbonate rock units, there was no significant change in the in-situ ^87^Sr/^86^Sr_calcite_ values (Fig. 3). It behaves within a similar range (within the range < 0.7110). However, the trend of all ^87^Sr/^86^Sr_calcite_ values in the ^87^Sr/^86^Sr_calcite_ vs. 1/Sr correlation also converged to a value of about 0.7085 (Fig. 4b). This suggests that it is highly likely that the carbonate successions in the study area were also affected by a hydrothermal fluid event after deposition. The calcite in the PBSC unit is cement, and the ^87^Sr/^86^Sr_calcite_ vs. 1/Sr trend shows a slight difference from the calcite in the carbonate rock units, which seems to be due to the difference in geochemical conditions at the time of each calcite formation (Fig. 4b).

## Discussion

### Chronology of PBSC unit

The age of the PBSC unit should be considered comprehensively with the age of the lower and upper carbonate rock units. (1) The age of the upper part of the lower carbonate rock unit was constrained from the latest Middle Ordovician to the earliest Late Ordovician by Hong and Lee^25^. Considering conodont biostratigraphy by Lee^26^ and Cheong et al.^27^, they proposed the age using correlation of carbon isotope based chemo-stratigraphy as an alternative approach due to the lack of a fossil index in this unit. (2) For the age of the PBSC unit, an age frequency graph was presented by combining the highly reliable U–Pb zircon age dating results reported by Jang^18^ and the results of several sites in the PBSC unit (Supplementary Fig. 7). (3) In addition, based on Conodont biostratigraphy by Cheong et al.^27^ and Lee^28^, the upper carbonate rock unit was considered a Silurian formation. Considering the above points comprehensively, we constrained the deposition period of the PBSC unit as the OST period.

In the recently reported Conodont Biostratigraphy, the upper part of the lower carbonate rock and PBSC units were suggested as Middle Ordovician (Darriwilian)^29^, and the upper carbonate rock unit as Upper Ordovician (Sandbian)^30^. Compared to the results we used in the previous studies and this study mentioned above, there are some conflicting points. Since both detrital dolomite pebbles and single dolomite grains are observed in the microfacies of PBSC units, Middle Ordovician (Darriwilian) conodonts could be sufficiently found in conodonts derived from PBSC units with these characteristics. However, in the conodont study of the upper carbonate rock unit, the species of the previous research group^28^ and the latest research group^30^ were not completely matched, and no direct comparison with similar species was presented. The only one mentioned together in both groups was *Panderodus gracilis* (*Panderodus species*), which was one of the dominant species in the upper carbonate rock unit. This species has been reported to have relatively long biostratigraphic ranges (mainly Middle Ordovician to Silurian; Rexroad and Craig^31^). Thus, there is still a prolonged debate about dating by biostratigraphy alone.

We respect conodont-based biostratigraphy as one of the powerful correlation methods. However, we clarify the following points in this study: (1) The age of the upper part of the lower carbonate rock unit was constrained as at least Middle to Late Ordovician based on carbon isotope stratigraphy considering conodont biostratigraphy. (2) The deposition period of the PBSC unit was constrained to the OST period based on the frequency of zircon U–Pb age dating results. (3) The possibility of a detrital conodont could not be ruled out in the carbonate platform undergoing the advent and retreat of a shallow marine environment as the sea level rises and falls. Therefore, the results of zircon age dating, chemo-stratigraphy, and biostratigraphy should be complemented mutually and interpreted comprehensively.

### δ^18^O isotope, an indicator for provenance change

In the carbonate rock and PBSC units, the characteristics of quartz, including micro-texture, abundance, and isotope value, are completely distinct.

In the micro-texture of the carbonate rock unit, quartz has fine-sized grains (usually several μm to tens of μm, rarely up to 200 μm), subhedral to anhedral shape, and distal influx characteristics are observed (Fig. 3d; Supplementary Fig. 6). In the micro-texture of the PBSC unit, quartz has various grain sizes (tens to hundreds of μm), poor sorting, subangular shape with poor roundness, and proximal influx characteristics are observed (Fig. 2d and e; Supplementary Figs. 2 and 5).

**Figure 5:**
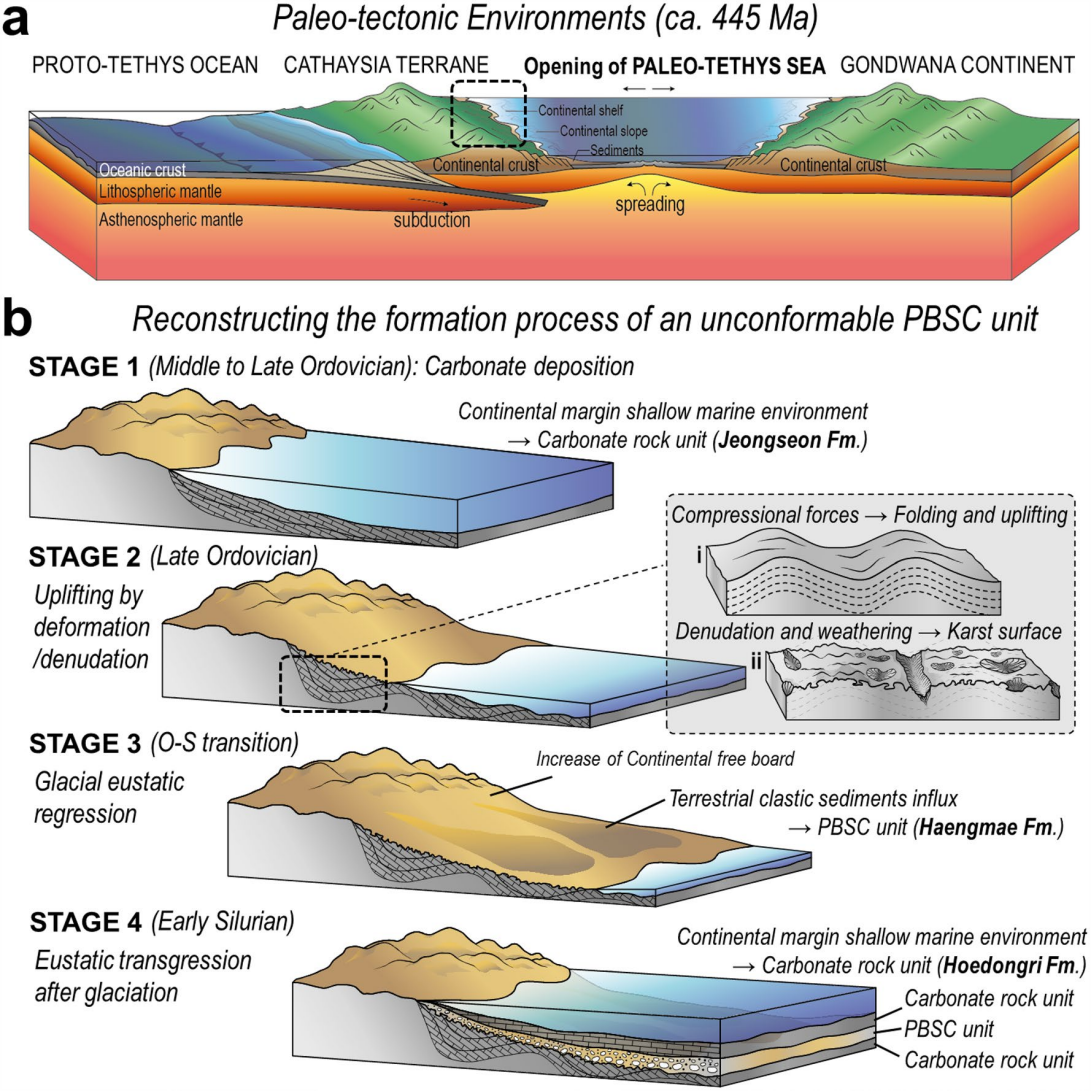
Conceptual illustration of paleo-tectonic environments and paleo-depositional model. (**a**) Conceptual illustration showing the northwest margin of Gondwana at ca. 445 Ma, including the Cathaysia terrane breaking-off from the Gondwana and subsequent Paleo-Tethys Sea opening. (**b**) A proposed depositional model for the PBSC unit creation in the Cathaysia terrane, inferred from its clastic lithology, the unconformable relationship with the lower carbonate unit, in-situ δ^18^O_quartz_ and ^87^Sr/^86^Sr_carbonate_ proxies, and the global events during the OST period. Detailed explanations of each stage are described in the text.

The quartz abundance of the PBSC unit is closely related to the geological processes that acted on this carbonate platform. The lower carbonate rock unit underwent folding and uplifting by compressive force (Figs. 1 and 5) and formed karst by denudation, weathering, and erosion (Figs. 2g, 3d, and 5; Supplementary Figs. 1 and 4). The exposure of the continental board due to geological events (uplifting, weathering, and erosion) provides sufficient conditions to increase the supply of clasts to the PBSC unit. The decisive event was the increase in the continental free board due to the rapid decrease in sea level during the OST period (Supplementary Fig. 5). This massively increased the amount of clasts that could be deposited. We confirmed a significant influx of terrestrial clasts (especially quartz, dolomite pebble, and dolomite) in both micro-texture observation and quantitative XRD analysis (Fig. 3d).

Since the quartz in the carbonate rock units was deposited being distal, it is ambiguous to estimate the provenance. However, from the exposed continental board, quartz deposited in the PBSC unit as being proximal has δ^18^O values ranging from 17.55 to 23.65 ‰. Unlike quartz of carbonate rock units, which has a range of 23.56 ~ 27.01 ‰, it strongly indicates that it is a completely different provenance material. The sedimentary structure of the detrital clast unit is very poor and vulnerable to various post-depositional events. Nevertheless, since quartz is very resistant to oxygen isotope exchange^20^, it preserves the original oxygen isotope value. It is largely unaffected by post-depositional alterations such as intense weathering and denudation. Therefore, in-situ δ^18^O_quartz_ considering the micro-texture and content characteristics of quartz is a useful proxy as an indicator of provenance change before and after OST.

### ^87^Sr/^86^Sr isotope, a geochemical indicator for syn-/post-depositional event

We found records of different geochemical conditions from the Sr isotope values of carbonate minerals (calcite and dolomite) by attempting in-situ mineral analysis (Figs. 3d and 4). There are two things to note here: First, two trends are evident in the ^87^Sr/^86^Sr_dolomite_ value of carbonate rock and PBSC units (Fig. 4a). Considering the characteristics of the PBSC unit, a significantly fluctuating ^87^Sr/^86^Sr ratio and relatively high Sr composition were confirmed in single dolomite grain and dolomite pebble. This means that dolomite with syn-depositional values under different geochemical conditions was introduced into the PBSC unit. Second, the ^87^Sr/^86^Sr isotope value converges to a specific value (ca. 0.7084) under the influence of hydrothermal alteration as a post-depositional event. A PBSC unit is a typical clastic rock unit. Compared to the lower and upper carbonate rock units, this unit shows porous characteristics in both macro and micro scales (Fig. 2a; Supplementary Fig. 2). Because of this, after the syn-depositional event, this unit could act as a channel for hydrothermal fluid flowing from the fault zone (Fig. 1c; Supplementary Fig. 1c). Park et al.^19^ reported phengite with muscovite–celadonite solid solution relationship as evidence of hydrothermal fluid introduced into the syn-sedimentary growth-fault in the PBSC unit. In micro-texture, phengite is observed in crystalline and pore-filling types, which are fluid-filled, not detrital.

The value of ^87^Sr/^86^Sr records both syn-depositional and post-depositional geochemical conditions. There are many factors that affect ^87^Sr/^86^Sr^14,21^, and the causes have been proposed as follows. Potential causes of increase include (1) diagenetic alteration, (2) contamination from detrital siliciclastic; Potential causes of the decrease include (1) increased rates of seafloor spreading and hydrothermal activity that provided a higher input of non-radiogenic Sr to the ocean (2) hydrothermal activity, (3) eustatic sea level rise that flooded radiogenic source area, increased weathering rates of volcanic rock, (4) ocean ventilation, non-radiogenic Sr reservoir. Based on this, in our study, the detrital clastic unit, PBSC, has a higher initial value than the ^87^Sr/^86^Sr_dolomite_ value of the carbonate rock unit, which means that it is derived from completely different geochemical conditions at the time of syn-deposition. In this carbonate platform, the ^87^Sr/^86^Sr value follows a decreasing trend (hydrothermal alteration trend) due to the Sr exchange of carbonate mineral under the influence of hydrothermal fluid after deposition. Therefore, the ^87^Sr/^86^Sr isotope of carbonate minerals is a useful proxy as a geochemical indicator of syn-/post-depositional events.

### Depositional model for the PBSC unit creation

Summarizing the characteristics of the PBSC unit, the unit (1) shows typical terrestrial clastic lithology characteristics, (2) has an angular unconformity relationship with the lower carbonate unit, (3) was temporally formed at the time of the Ordovician–Silurian transition, (4) was spatially located in the Cathaysia terrane of the Gondwana Supercontinent. Therefore, it is clear that these characteristics of the PBSC unit are closely related to the geological events that occurred in the Gondwana during the OST period. The most representative geological events are the Paleo-Tethys Sea opening and global glacial eustatic regression. In particular, in the PBSC unit, which has an age constraint as an OST period, the continental free board increased according to the abrupt drop in sea level, resulting in a large amount of terrestrial clasts influx. By linking these geological events with the clastic lithology and in-situ isotope chemo-stratigraphic characteristics of the PBSC unit, it can be inferred that the PBSC unit was formed through the following steps (Fig. 5).

Stage-1: Sedimentation of a carbonate unit (Jeongseon Fm.) begins in the Middle Ordovician period in the shallow marine environment of the terrestrial margin formed by the break-up of the Cathaysia terrane from the Gondwana.

Stage-2: During the Late Ordovician period, the separation of the Cathaysia terrane and the subsequent opening of the Paleo-Tethys Sea was in full swing, and as plate movement occurred on the opposite side of the Cathaysia terrane, the southeastern Cathaysia margin was placed under a compressional condition from both sides. This causes deformation and uplifting of the carbonate unit (Jeongseon Fm.) deposited in the Cathaysia terrane (Stage-2i), and is exposed to the surface and denuded by weathering to create the Karst surface (Stage-2ii).

Stage-3: The eustatic sea-level regression by the global glaciation maximum in the Latest Ordovician period leads to an increase in continental exposure during the OST period, and a large amount of terrestrial clasts produced by erosion occurs. These cover the Karst surface of the carbonate unit and form an unconformable PBSC unit.

Stage-4: As the global glaciation ends in the Silurian period, the top of the PBSC unit turns into a shallow marine environment again through gradual eustatic transgression, and the deposition of another carbonate unit (Hoedongri Fm.) begins. Subsequent geological processes cause various alterations to the relatively porous PBSC unit and precipitate secondary minerals (dolomite, phengite, and calcite cement).

In conclusion, the formation process model of the PBSC unit in the Cathaysia terrane region can be a criterion to explain the clastic sedimentary succession created in each continent during the OST period.

## Methods

### Sample preparation

Several field works were conducted from November 2019 to April 2023 in the Taebaeksan Basin of the Republic of Korea (South Korea). Based on the structural, sedimentological, and mineralogical characteristics of the traceable PBSC and carbonate rock unit, mapping was performed and a geological map was drawn (Supplementary Fig. 1). All PBSC and carbonate rock samples were polished as slabs, thin sections, and epoxy mounts. Polishing was conducted in several steps, starting at 20 μm and finishing at 0.25 μm. Mineralogical and micro-textural characteristics were examined using a polarizing microscope and SEM–EDS with a back-scattered Electron (BSE) detector high-vacuum (HV) SEM (JSM-5610LV; JEOL Ltd., Tokyo, Japan) and an EDS system (Oxford Instruments, Abingdon, UK) at Yonsei University, Seoul, South Korea. In order to apply the in-situ method to isotope analysis, a route map was created to confirm the distribution and texture of the target mineral with merging compo images.

### In-situ oxygen isotope analyses of quartz minerals

A total of 313 (including reference data) in-situ oxygen isotope analyses of quartz minerals were performed with LG-SIMS (CAMECA IMS1300-HR^3^ Large-Geometry SIMS) from KBSI (Ochang, South Korea. On the Au-coated epoxy mount surface with a thickness of 20 nm, δ^16^O_quartz_ and δ^18^O_quartz_ signals were obtained from the core to the rim of the grain with a 10 μm spot beam size. The signal was obtained in 20 cycles, and the measurement time was about 7–8 min. As a reference material, Unil-Q^32^, natural crystals of quartz from Chile, provided by Lausanne University, was used. In this study, the in-situ δ^18^O_quartz_ value of Unil-Q was 9.8564 ± 0.2604‰ (2 s), which has high reliability and reproducibility. Raw data and calculated δ^18^O_quartz_ values are reported in Supplementary Table 3.

### In-situ strontium isotope analyses of carbonate minerals

A total of 591 (including reference data) in-situ strontium isotope analyses of carbonate minerals were performed with LA-MC-ICP-MS (Nu Plasma II MC-ICP-MS with NWR 193uc laser system) from KBSI (Ochang, South Korea). Calcite cement, dolomite grains, and dolomite pebbles were targeted and analyzed with the following ablation conditions: 100 μm spot beam size of single hole drilling ablation mode, 30 secs dwell time, and a fluence of ~ 4.0 J/cm^2^. The ^87^Sr/^86^Sr value of the reference material (KRNC-1) is 0.708604 ± 0.000022, which has high reliability and reproducibility. Reference material, NIST, and calculated ^87^Sr/^86^Sr of carbonate mineral (calcite, dolomite) values are reported in Supplementary Table 4.

### X-ray diffraction with RIR method

A total of 66 powdered bulk rock samples were analyzed with the Rigaku Miniflex II X-ray Diffraction (XRD) system from Yonsei University. All rock samples were ground into fine-sized powder using a mortar and pestle, and mixed homogeneously with a corundum (Al_2_O_3_) powder in a weight ratio of 1:1. With the prepared powder samples, the XRD system was performed under CuKα radiation, step-scan mode 0.02° step, 1 s/step scanning time, and 2–55° 2θ ranges conditions using a 6-sample holder. Semi-quantitative analysis of mineral composition was conducted with the Reference Intensity Ratio (RIR) method^33–37^. From the results of X-ray diffraction analysis, the ratio of peak intensities of corundum and major minerals (calcite, dolomite, quartz, illite, etc.) was determined. Quantitative values were then calculated using the theoretical RIR values. The quantitative analysis results for major minerals are presented in Supplementary Table 2, and among the data, the results for quartz are shown in Fig. 3d.

### Zircon U–Pb age dating

The PBSC unit rock sample was crushed and the heavy mineral concentrate obtained by the heavy liquid separation process was obtained. The zircon grains were handpicked through a stereoscopic microscope. The separated zircon grains were mounted in epoxy resin with a reference zircon. After polishing the mount, analytical points were considered by observing the micro-texture with SEM-BSE. U–Pb age dating was performed using a Nu Plasma II multi-collector inductively coupled plasma mass spectrometry (LA-MC-ICP-MS) instrument equipped with a New Wave Research 193-nm ArF excimer laser ablation system at the KBSI (Korea Basic Science Institute). All analyses were conducted with a spot size of 15 μm, a repetition rate of 5 Hz, and an energy density of 4.5 J/cm^2^. The background intensity time of 30 secs, dwell time of 30 secs, and washout time of 20 secs were set. As reference zircons, 91,500 and Plesovice^38,39^ were used. All age values were calculated after data reduction using Iolite^40,41^ and Isoplot^42^ softwares. Frequency histogram of detrital zircon U–Pb age dating for PBSC unit (Haengmae Fm.) integrating unpublished data (In this study) using LA-MC-ICP-MS and the results of previous results^17,18^ using LA-MC-ICP-MS and SHRIMP is presented in Supplementary Fig. 7. Raw data is reported in Supplementary Table 5.

### Supplementary Information

Supplementary Information.

## Data Availability

The authors confirm that the data supporting the findings of this study are available within the article and its supplementary materials.
