# Supplementary material for: In-situ δ18O and 87Sr/86Sr proxies in an unconformable clastic unit at the Ordovician–Silurian transition
Source: Sci Rep. 2023 Sep 13;13:15174. doi: 10.1038/s41598-023-42200-3 (PMC10499834; doi:10.1038/s41598-023-42200-3)
Supplement: Supplementary file 1 — Supplementary Information. [file 41598_2023_42200_MOESM1_ESM.pdf]

SUPPLEMENTARY MATERIALS

CONTENTS

- **SM1. LIST OF SUPPLEMENTARY FIGURES**

  Figure S1. Detailed Geological map.

  Figure S2. Outcrop of the PBSC unit.

  Figure S3. Outcrop of the lower carbonate unit.

  Figure S4. Karst surface of the lower carbonate unit.

  Figure S5. Lithological facies and micro-texture of the PBSC unit.

  Figure S6. Lithological facies and micro-texture of the upper/lower carbonate unit.

  Figure S7. Frequency histogram of detrital zircon U-Pb age dating for the PBSC unit.

- **SM2. LIST OF SUPPLEMENTARY TABLES**

  Table S1. References for distribution of O-S boundary strata

  Table S2. Quantitative XRD analyses for major mineral composition of target samples.

  Table S3. Analytical conditions and raw data for oxygen isotope of quartz using LG-SIMS.

  Table S4. Analytical conditions and raw data for strontium isotope of carbonate minerals (calcite and dolomite) using LA-MC-ICP-MS.

  Table S5. Zircon U-Pb isotope age data of the PBSC unit (SHRIMP and LA-MC-ICP-MS).

SM1. LIST OF SUPPLEMENTARY FIGURES

Figure S1. Detailed Geological map.

Figure S2. Outcrop of the PBSC unit.

Figure S3. Outcrop of the lower carbonate unit.

Figure S4. Karst surface of the lower carbonate unit.

Figure S5. Lithological facies and micro-texture of the PBSC unit.

Figure S6. Lithological facies and micro-texture of the upper/lower carbonate unit.

Figure S7. Frequency histogram of detrital zircon U-Pb age dating for the PBSC unit.

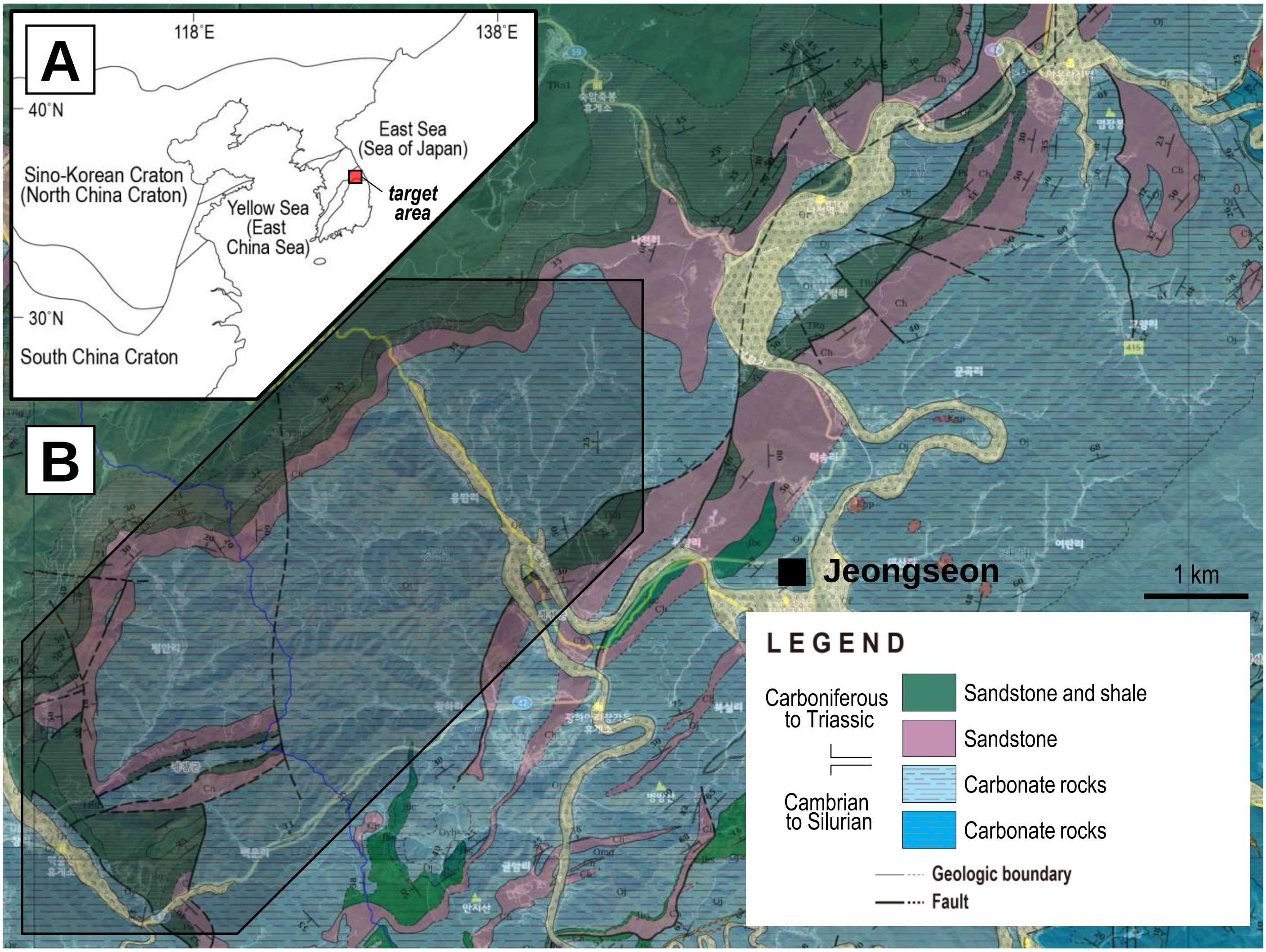

Figure S1. Detailed geological map. (A) Simplified geological map of East Asia. (B) Geological map for northeastern part of Taebaeksan zone, Korea Peninsula (captured from http://mgeo.kigam.re.kr/map/main.do?process=geology_50k). Shaded polygon is a target area in this study. (C) Field-based geological map showing major structures. (D) Cross-section of A-A', B-B', and C-C'. Figures were drawn using Adobe Illustrator CS6 version 16.0.0 (https://www.adobe.com/) and Microsoft PowerPoint Office 365 ProPlus version (https://www.microsoft365.com/).

C

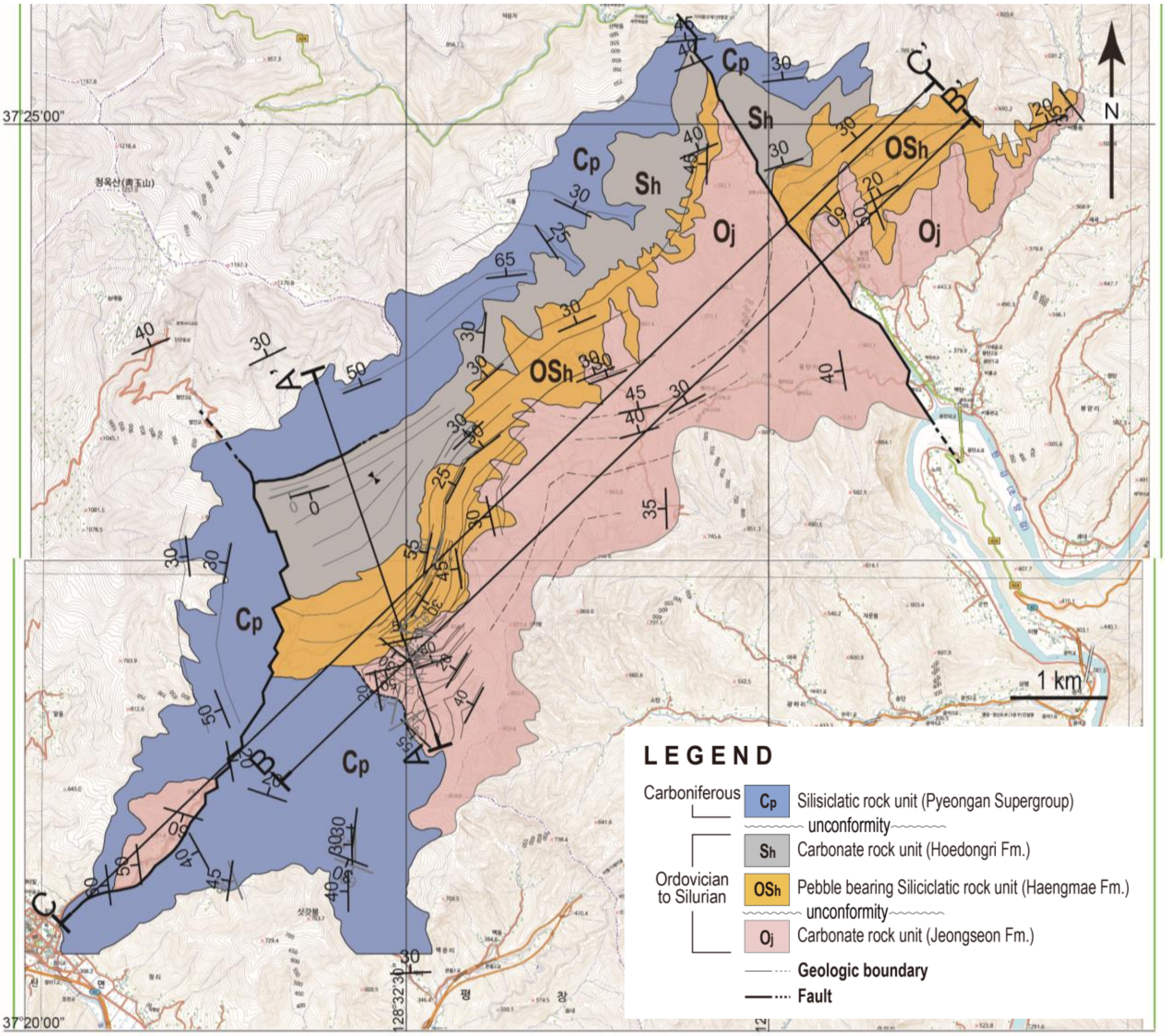

37°25'00"
37°20'00"
128°32'30"
N
1 km
C
C'
B
B'
A
A'
Cp
Sh
OSh
Oj
LEGEND
Carboniferous
Cp Silisiclatic rock unit (Pyeongan Supergroup)
unconformity
Ordovician to Silurian
Sh Carbonate rock unit (Hoedongri Fm.)
OSh Pebble bearing Siliciclatic rock unit (Haengmae Fm.)
unconformity
Oj Carbonate rock unit (Jeongseon Fm.)
Geologic boundary
Fault

D

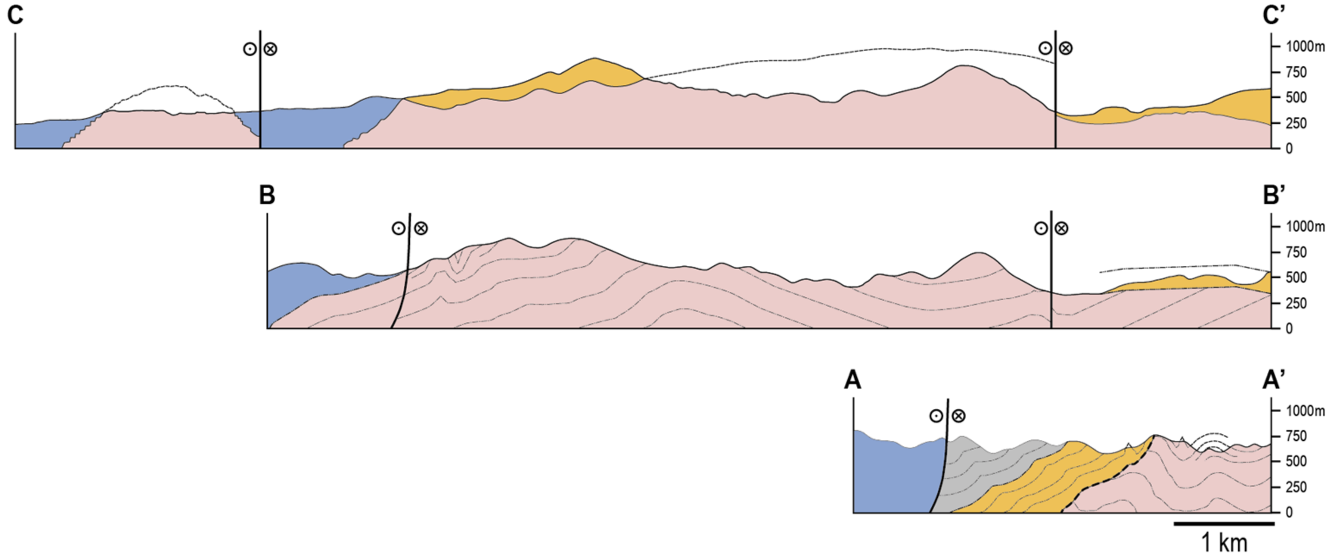

C
C'
B
B'
A
A'
1000m
750
500
250
0
1 km

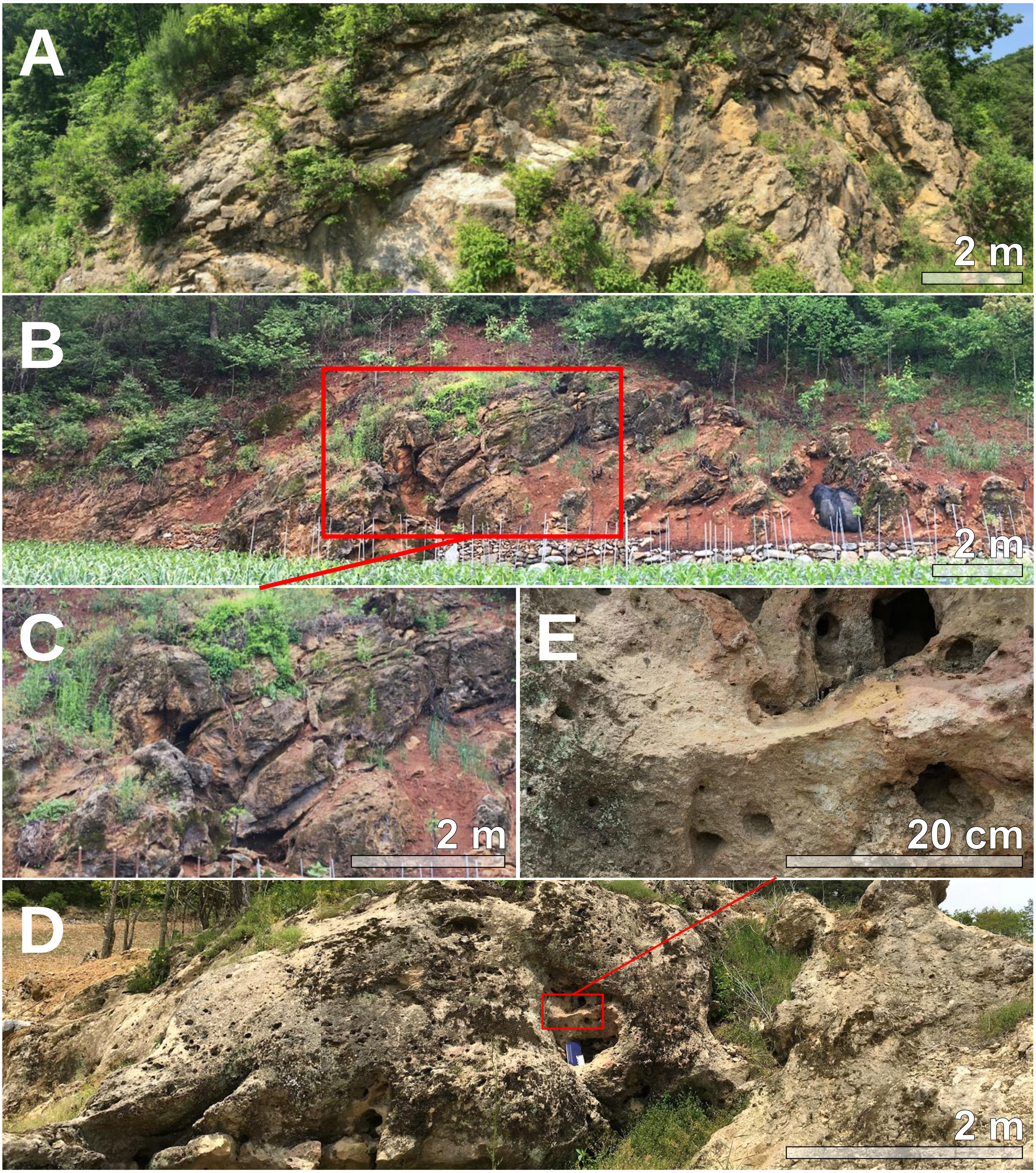

Figure S2. Outcrop of the PBSC unit (Haengmae Fm.).

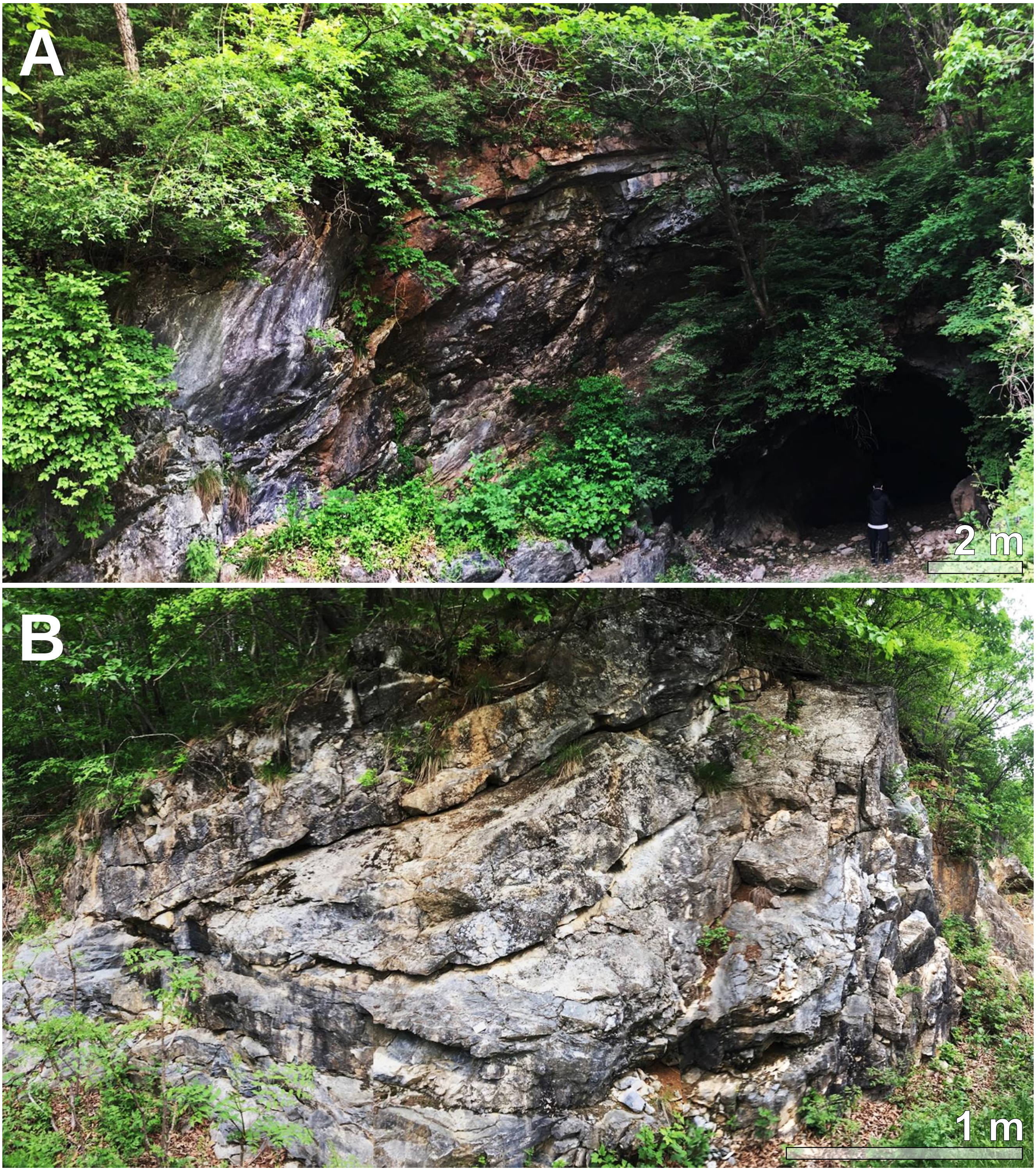

Figure S3. Outcrop of the lower carbonate unit (Jeongseon Fm.).

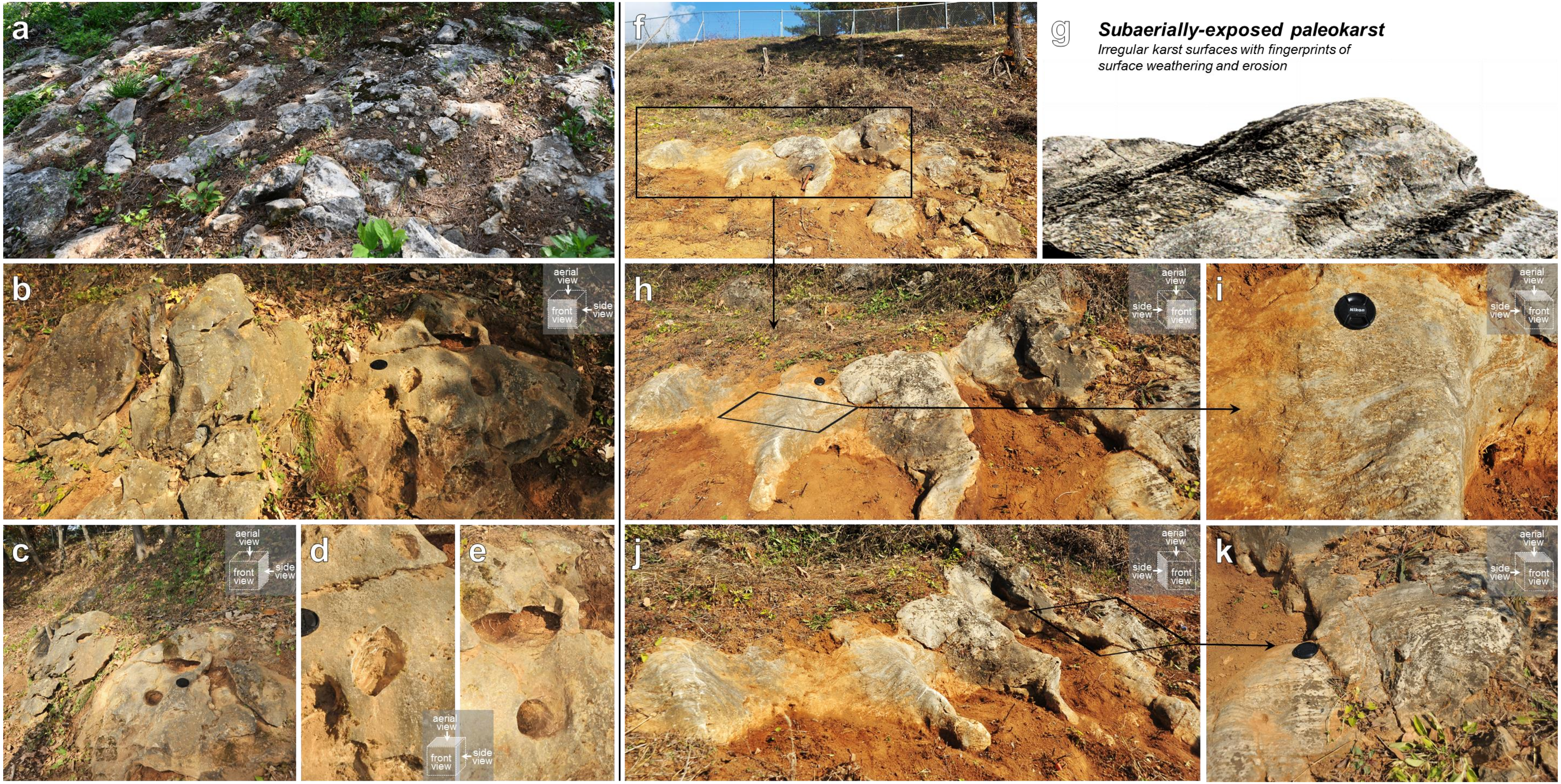

Figure S4. Karst surface of the lower carbonate unit (Jeongseon Fm.).

Figure S5. Lithological facies and micro-texture of the PBSC unit. (A) Representative lithological facies of the PBSC unit. The PBSC unit is characterized by the yellowish-brown in color and typical clastic rock with a granular matrix containing detrital pebbles. Dolomite pebbles composed of fine-grained dolomite(10-20 μm) aggregates are predominant in the PBSC unit. Small amounts of pebbles of micritic mudstone, quartzite, sandstone, and phyllite are included. (B)-(K) Representative micro-texture of the PBSC unit. (B)-(C) Polarized microscopic images of the PBSC unit. Under crossed polarized light (XPL) mode after alizarin red S staining show calcite matrix, detrital quartz and dolomite. (D)-(E) XPL mode and SEM-BSE compo image of dolomite pebble. (F) Plain polarized light (PPL) mode of the PBSC unit. Dolomite pebble and detrital dolomite and quartz grains are distributed calcite matrix. (G)-(J) SEM-BSE compo image of detrital dolomite and quartz grains. (K) Dolomite pebble in the PBSC unit. (L) SEM-BSE compo image of (K). Detrital dolomite and quartz grains are distributed in calcite matrix.

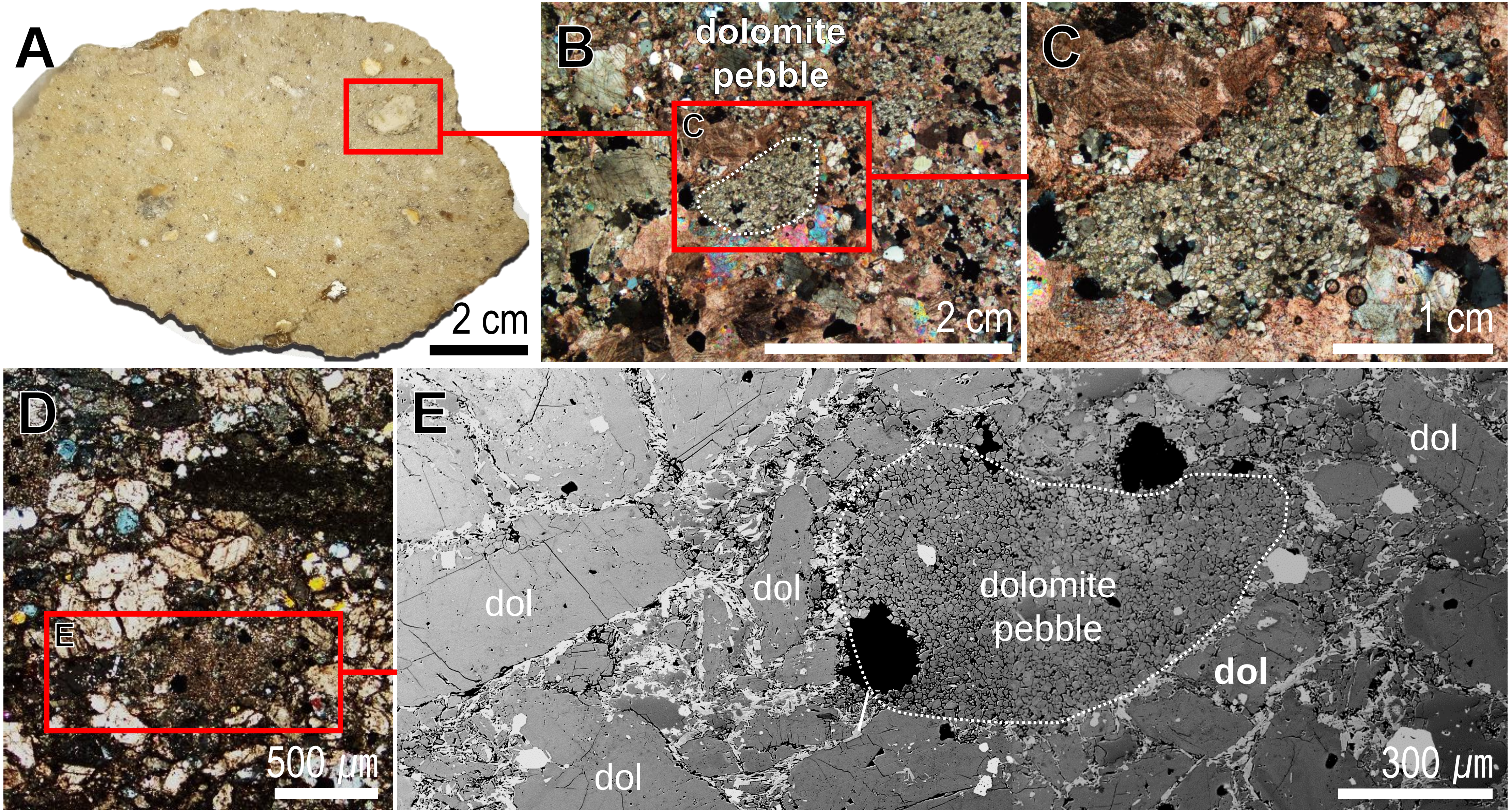

Figure S5. Lithological facies and micro-texture of the PBSC unit.

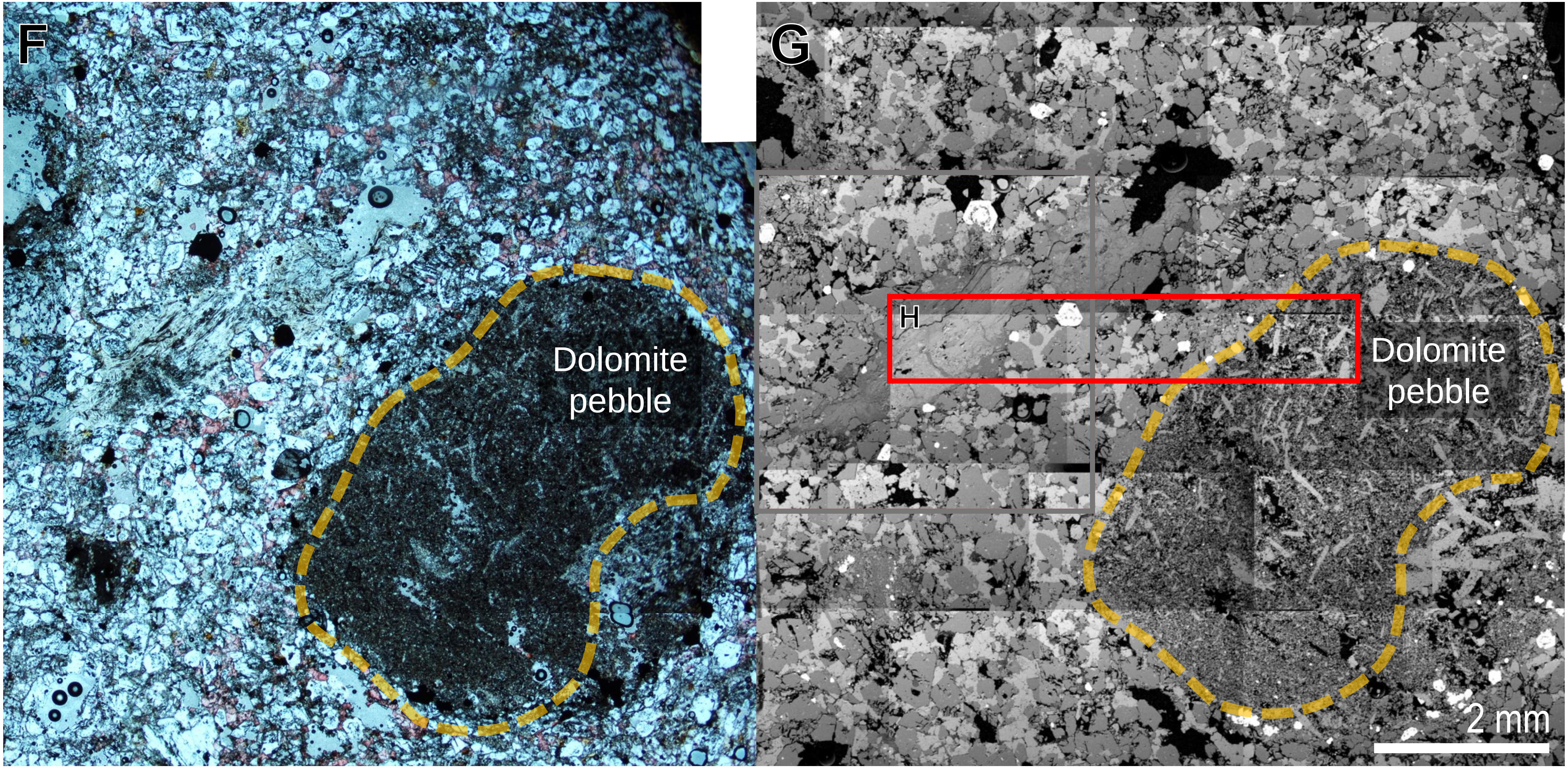

Figure S5. *(continued)*

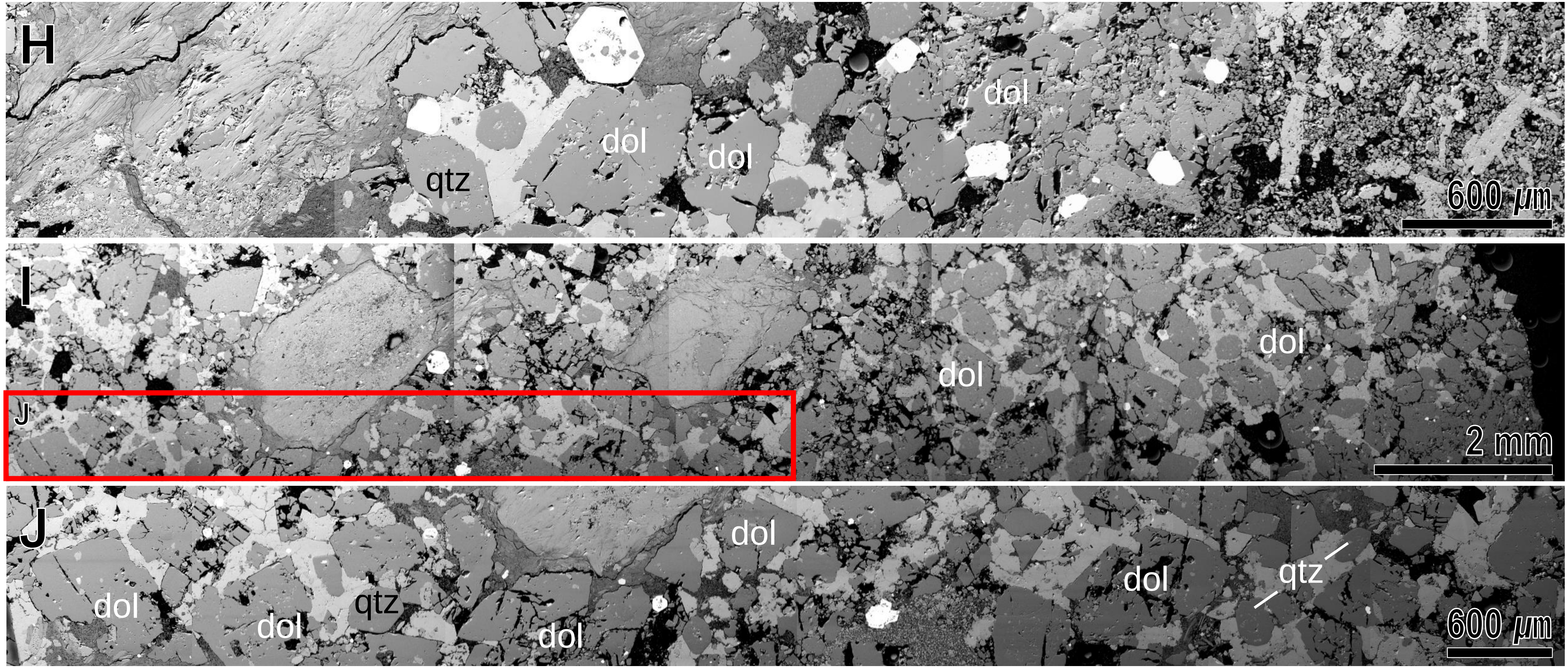

Figure S5. *(continued)*

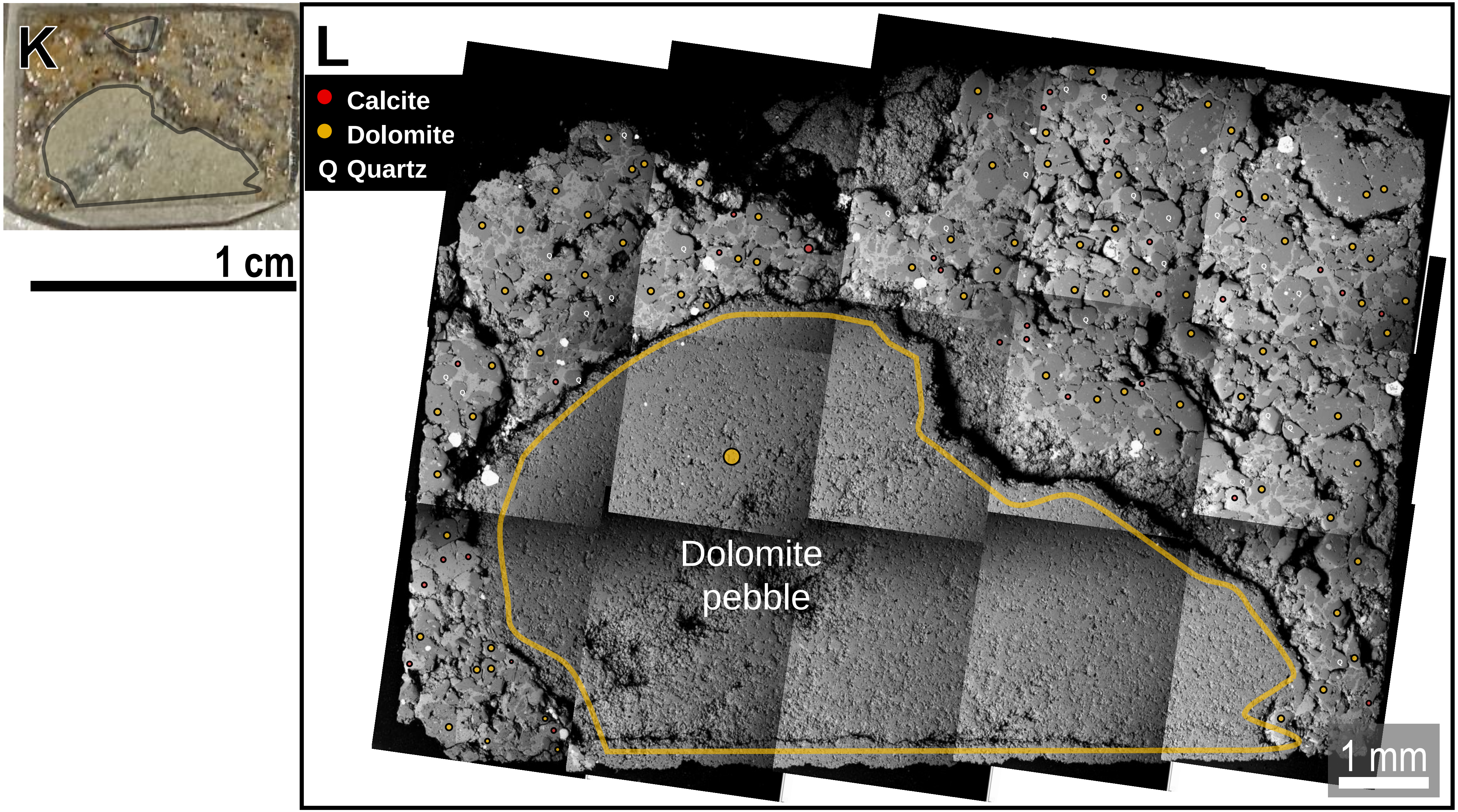

Figure S5. *(continued)*

Figure S6. Lithological facies and micro-texture of the upper/lower carbonate unit.

Figure S6-1. Lithological facies of the upper/lower carbonate unit.
(A)-(D) ***Light gray massive limestone facies***.
(E)-(F) ***Finely laminated dark gray limestone facies***.
(G)-(I) ***Dark gray massive limestone facies***.

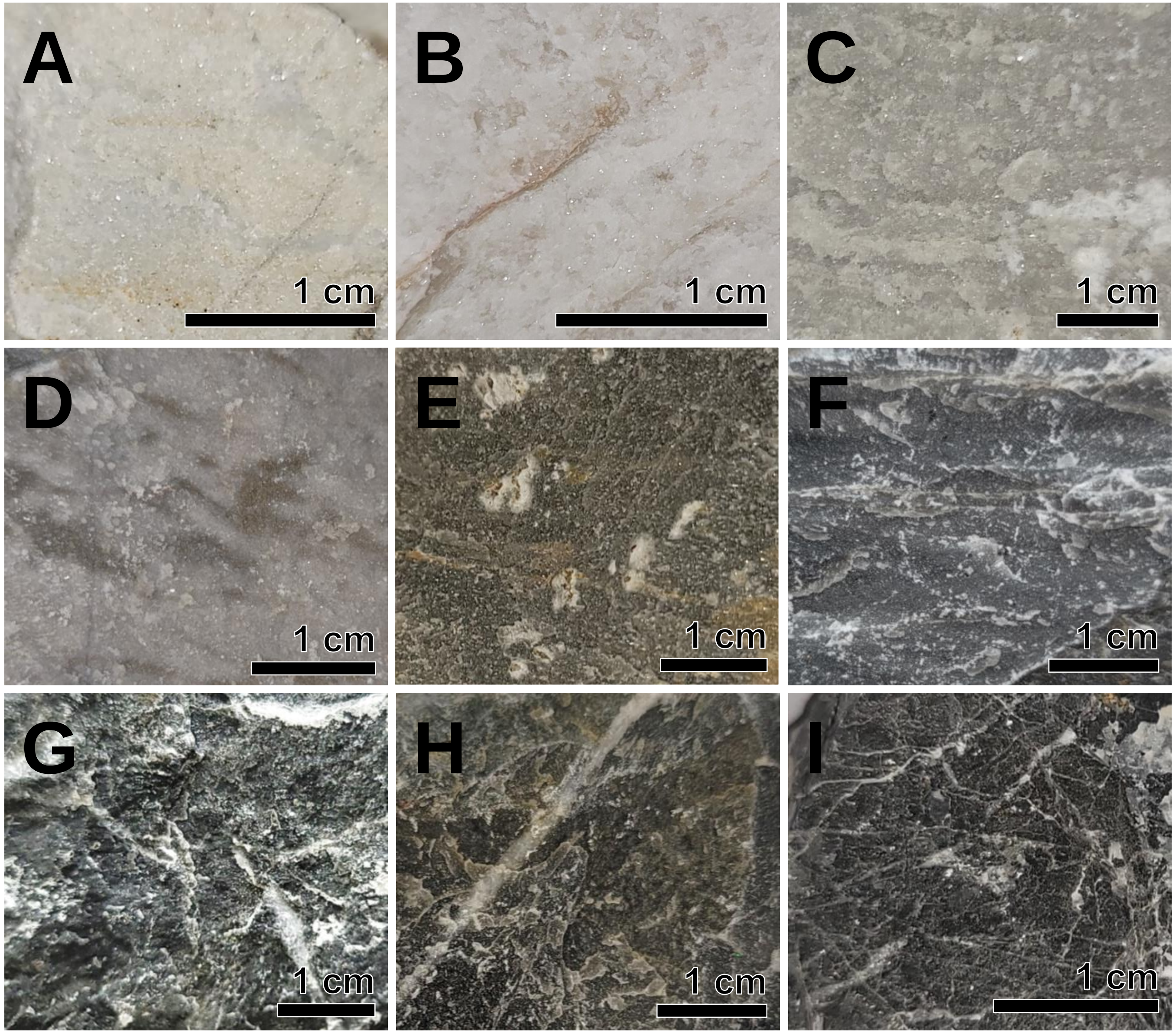

Figure S6-2. The SEM-BSE (Scanning Electron Microscope-Energy Dispersive X-ray Spectrometry) micro-texture images of the upper (A)-(M) and lower (N)-(U) carbonate rock units. In the case of detrital quartz in carbonate rock, it is very difficult to distinguish by macroscopic observation because it has very small-sized grains. In particular, both dolomite and quartz have various sizes ranging from tens to hundreds of μm, and it is difficult to distinguish them by contrast. Therefore, observation based on micro-texture and EDS detect results with SEM-BSE must be preceded. For analyzing ***in-situ*** $\delta^{18}O_{quartz}$ isotope with 10 μm spot size and distinguishing from dolomite grains, we confirmed the micro-texture and distribution of detrital quartz in carbonate rock with various lithofacies.

(A)-(B) ***Dark gray massive limestone facies*** The matrix consists of interlocking crystalline calcite with hundreds of μm in size. (C)-(D) ***Light to dark gray limestone facies*** Dolomite is mainly distributed in the calcite matrix. A few amounts of euhedral to subhedral detrital quartz with tens of μm in size are distributed. (E)-(F) ***Light gray massive limestone facies*** Subhedral to anhedral detrital quartz with hundreds of μm in size is distributed in the calcite matrix.(G)-(H) ***Finely laminated dark gray limestone facies*** Anhedral detrital quartz less than tens of μm in size is distributed in the calcite matrix.(I)-(M) ***Light to dark gray limestone facies*** Subhedral to anhedral detrital quartz and dolomite with tens to hundreds of μm in size are distributed in the calcite matrix.(N)-(O) ***Light gray massive limestone facies*** Subhedral to anhedral detrital quartz and dolomite with tens to hundreds of μm in size are distributed in the calcite matrix. (P)-(Q) ***Dark gray massive limestone facies*** Subhedral to anhedral detrital quartz less than tens of μm in size are distributed in the calcite matrix.(R)-(S) ***Light gray massive limestone facies*** The matrix consists of interlocking crystalline calcite with sizes of several tens of μm.(T)-(U) ***Light to dark gray limestone facies*** A few amounts of subhedral to anhedral detrital quartz less than tens of μm in size are distributed in the calcite matrix.

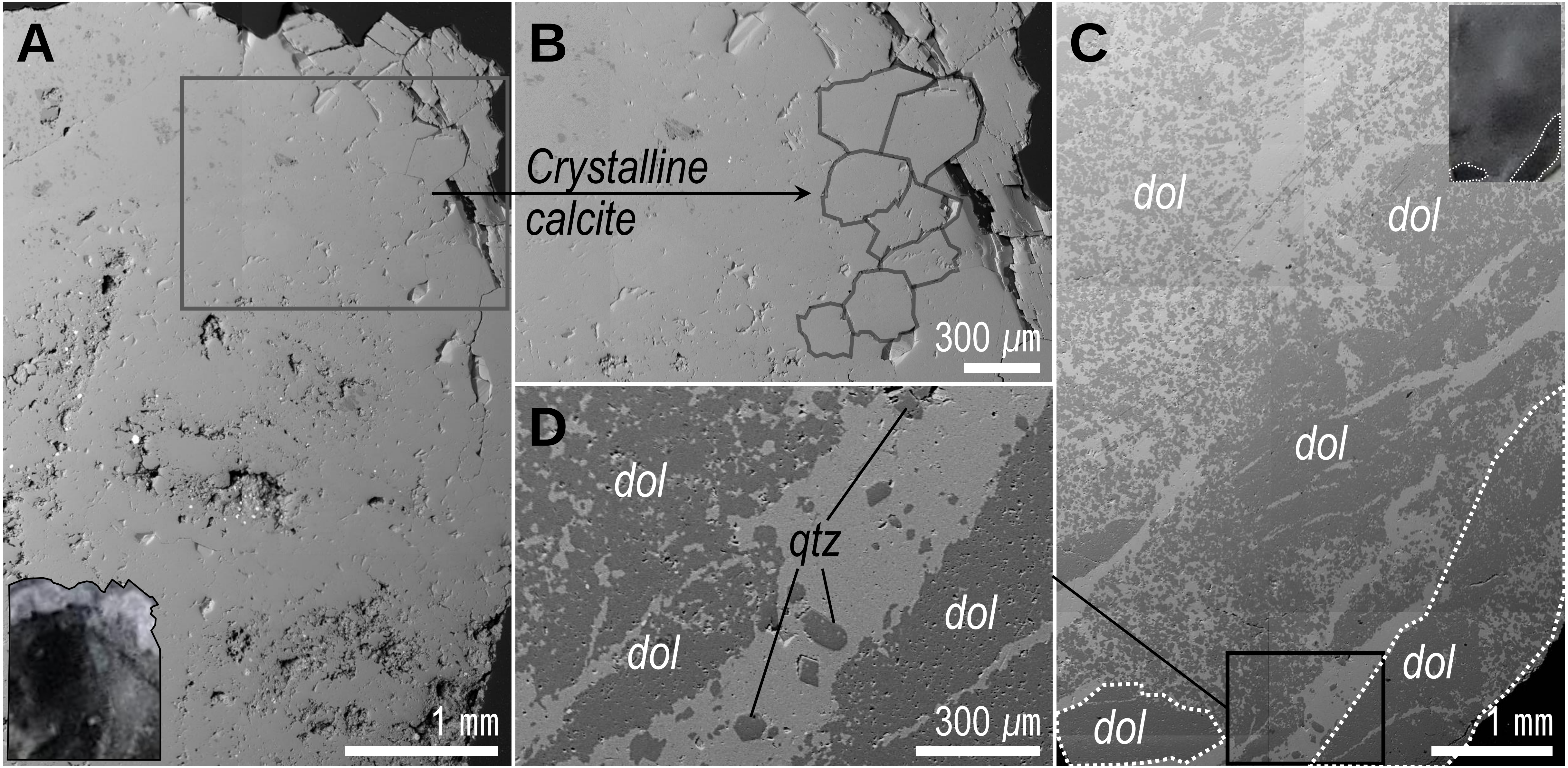

Figure S6-2. The micro-texture of upper/lower carbonate unit.

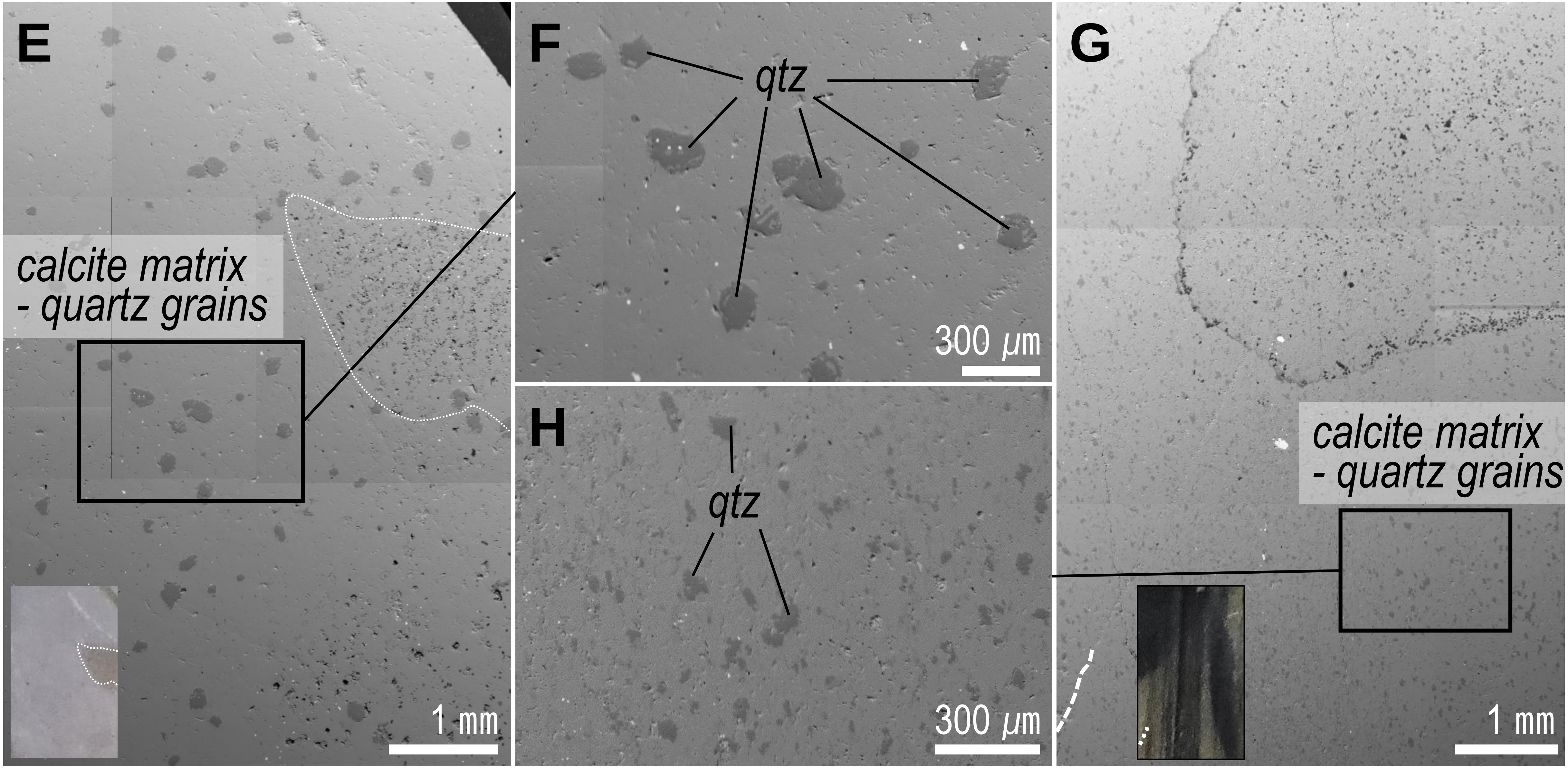

Figure S6-2. *(continued)*

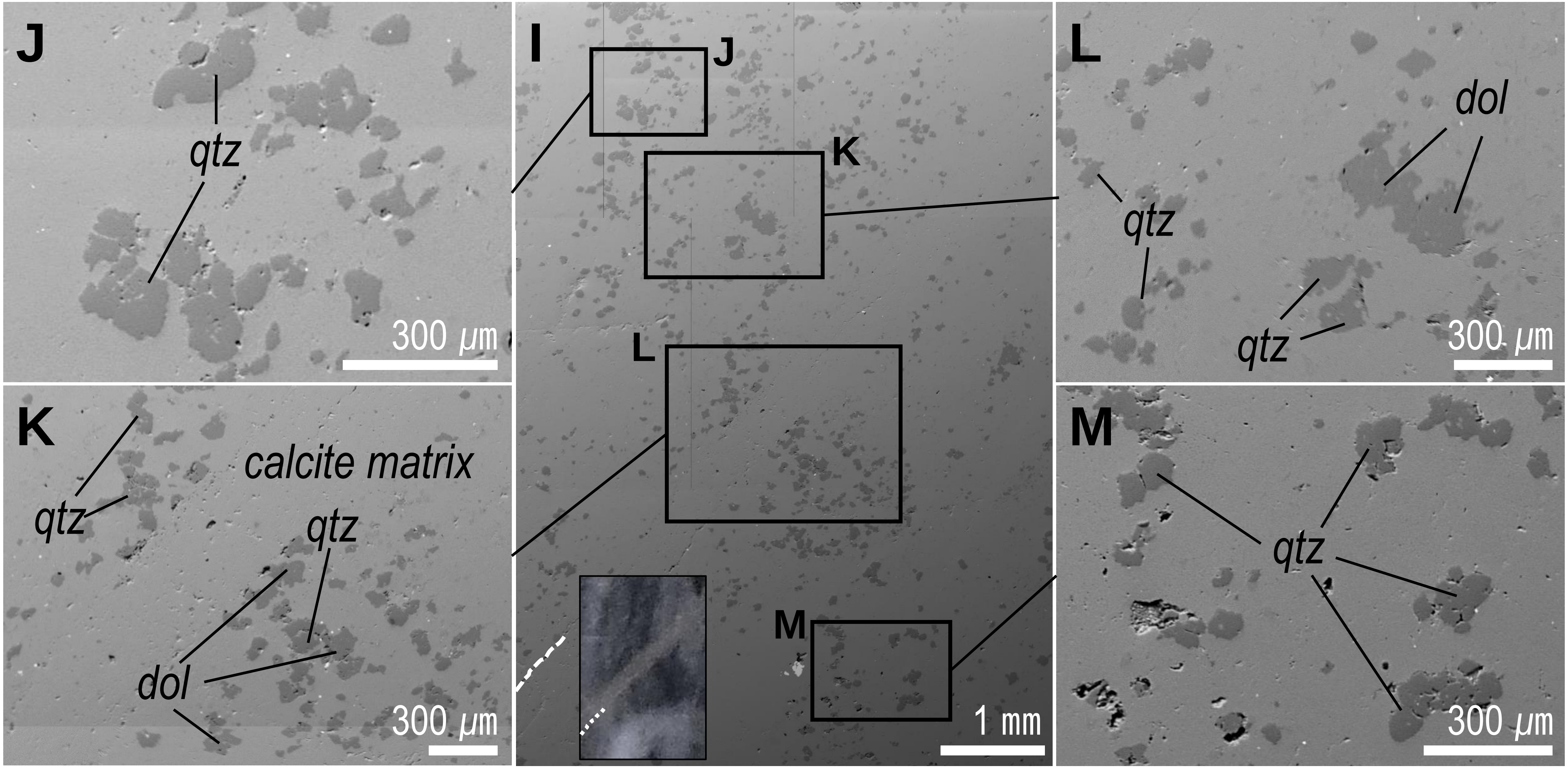

Figure S6-2. *(continued)*

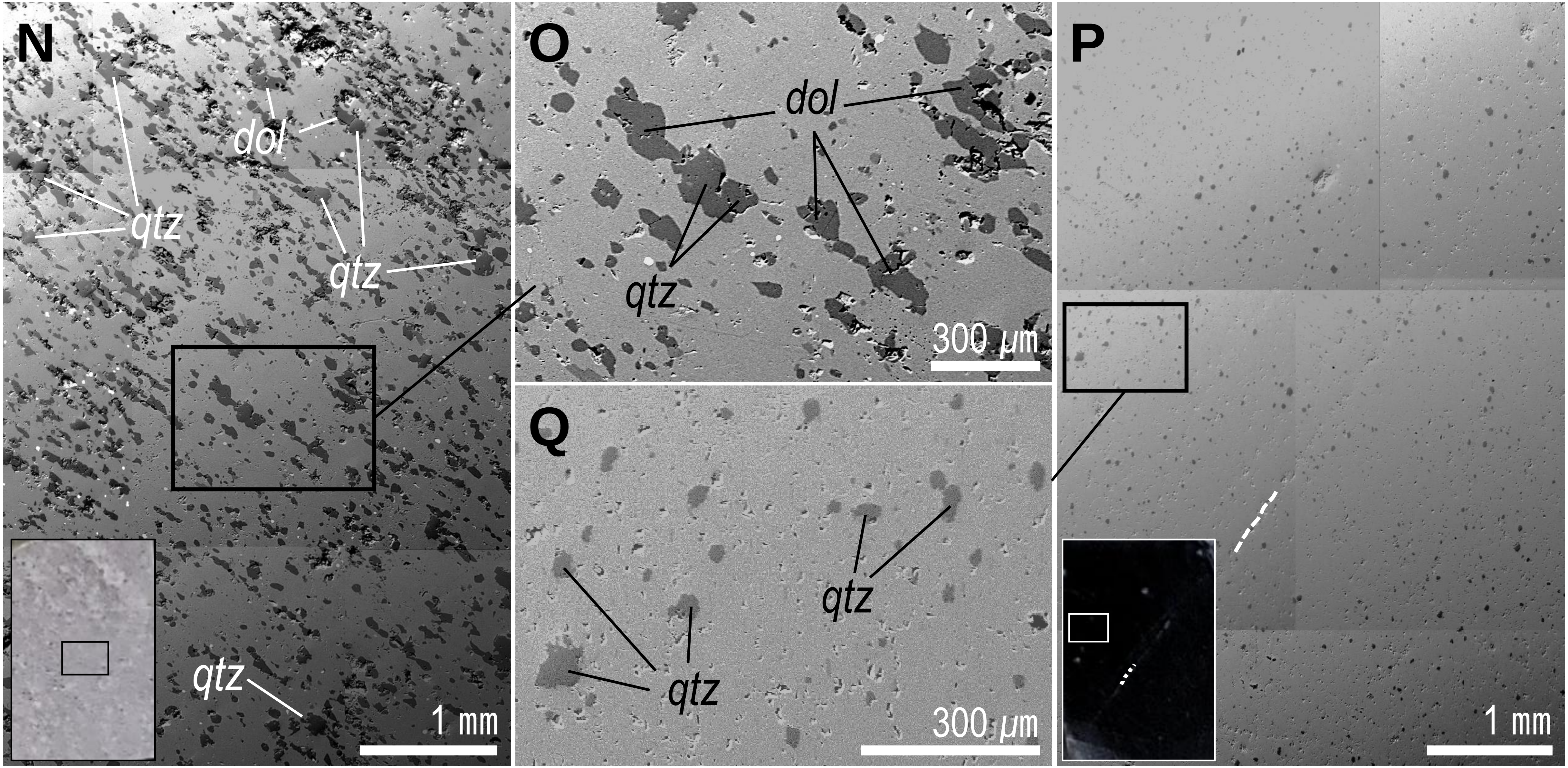

Figure S6-2. *(continued)*

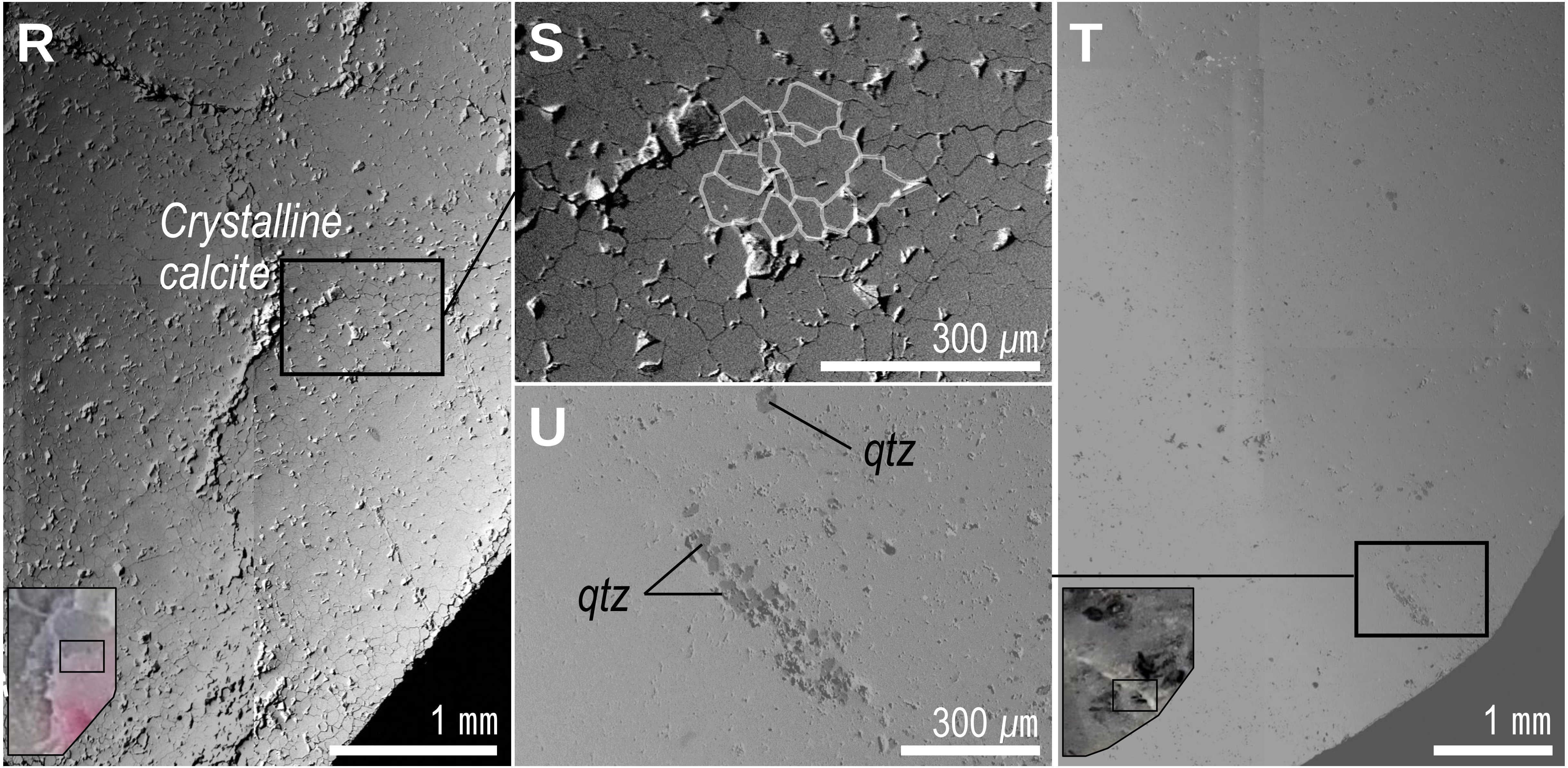

Figure S6-2. *(continued)*

(A) *Detrital Zircon U-Pb age dating for the PBSC unit (Haengmae Fm.)*

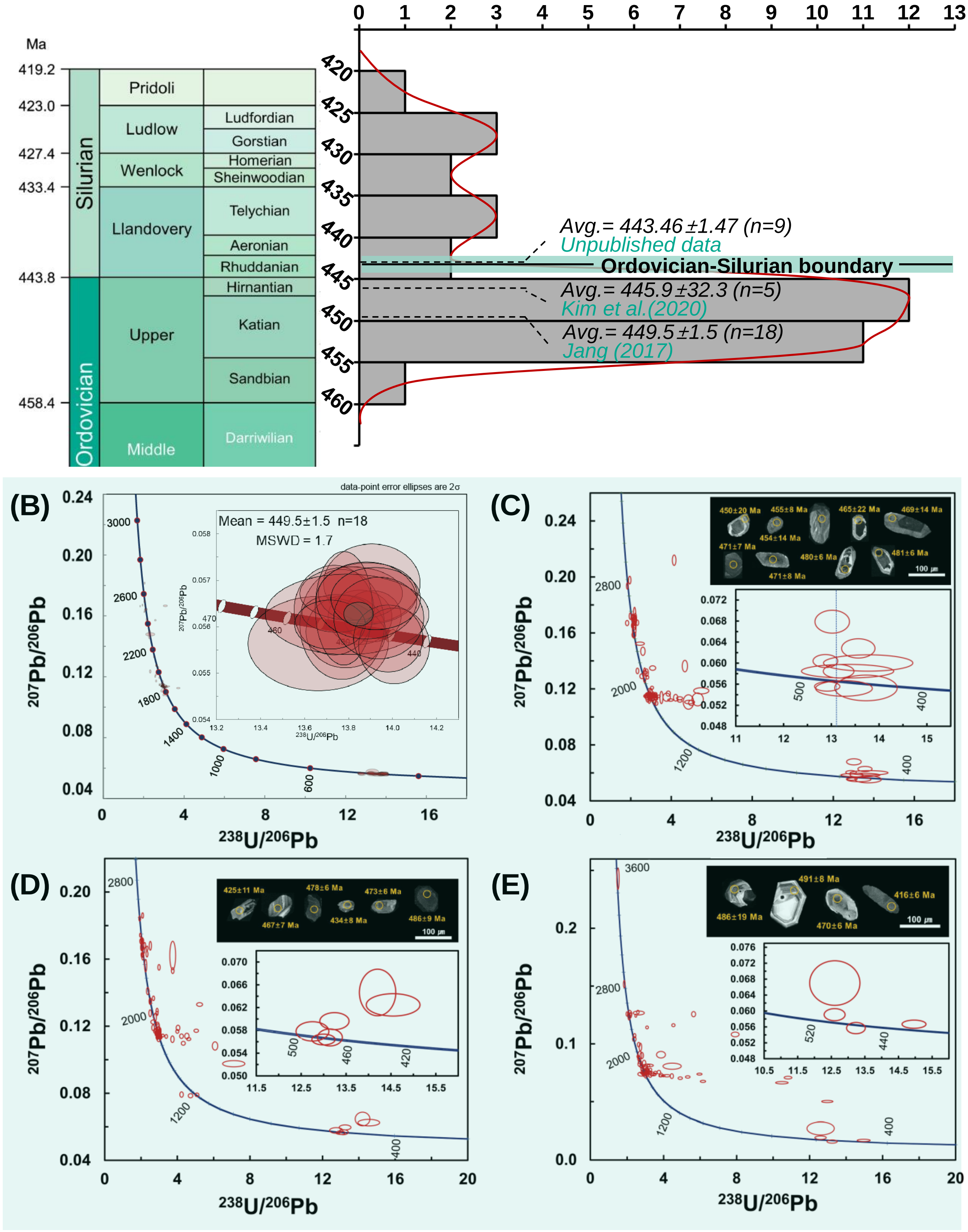

Figure S7. Frequency histogram of detrital zircon U-Pb age dating for the PBSC unit. (A) Unpublished data using LA-MC-ICP-MS (In this study) and the results of previous results (Jang, 2017; Kim et al., 2020) were integrated. (B) Terra-Wasserburg concordia plots for LA-MC-ICP-MS U-Pb isotope data of zircon from the PBSC unit–Biryong-dong site (Jang, 2017). (C)-(E) Terra-Wasserburg concordia plots for SHRIMP U-Pb isotope data of zircon from the PBSC unit–Pyeongan-ri and Haengmae-dong sites (Kim et al., 2020).

SM2. LIST OF SUPPLEMENTARY TABLES

Table S1. References for distribution of O-S boundary strata

Table S2. Quantitative XRD analyses for major mineral composition of target samples.

Table S3. Analytical conditions and raw data for oxygen isotope of quartz using LG-SIMS.

Table S4. Analytical conditions and raw data for strontium isotope of carbonate minerals (calcite and dolomite) using LA-MC-ICP-MS.

Table S5. Zircon U-Pb isotope age data of the PBSC unit (SHRIMP and LA-MC-ICP-MS).

Table S1. References for distribution of O-S boundary strata

<table>
<tr><th>No.</th><th>Area</th><th>Location (Paleocontinent
and columnar section image)</th><th>Age</th><th>Characteristics
(Lithology and sedimentary environment)</th><th>Reference</th></tr>
<tr><td>1</td><td>North Greenland</td><td>Between Laulentia and Baltic
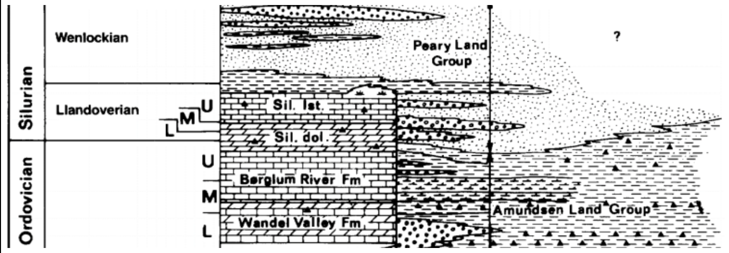
</td><td>Early Silurian (Cambrian to Silurian)</td><td>Turbidite or carbonate conglomerate derived from shelf margin
Carbonate conglomerate wedges accumulated at the base of the slope
Facies-limestone conglomerate_debris flows derived from shelf margin
Siltstone turbidites_overflow of conglomerate wedges
Carbonate conglomerate_mainly debris flow origin</td><td>Hurst, J. M., & Surlyk, F. Tectonic control of Silurian carbonate-shelf margin morphology and facies, North Greenland. AAPG bull. 68, 1-17; https://doi.org/10.1306/AD460959-16F7-11D7-8645000102C1865D (1984).</td></tr>
<tr><td></td><td>North, North-East Greenland</td><td>Between Laulentia and Baltic
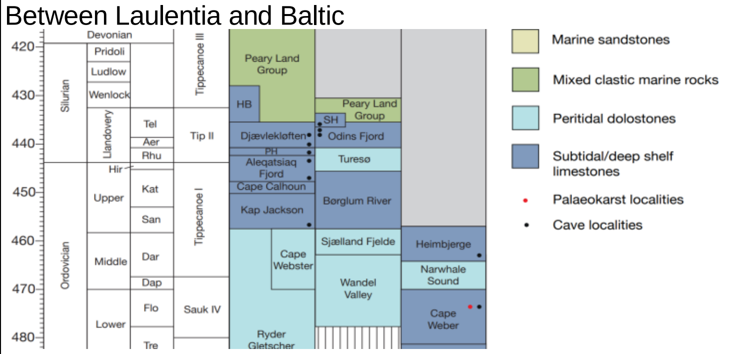
</td><td>Upper Ordovician (Sandbian) to Silurian (Wenlock)</td><td>Late Ordovician_subtidal/deep shelf Limestone, Peritidal dolostone
Silurian_mixed clastic marine rocks
Paleokarst, clastic strata, unconformity surface
Upper Ordovician (Sandbian) to Silurian (Wenlock) shelf Limestones
Llandovery conglomerate</td><td>Smith, P. & Moseley, G. The karst and palaeokarst of North and North-East Greenland – physical records of cryptic geological intervals. GEUS B. 49, 1-23; https://doi.org/10.34194/geusb.v49.8298 (2022).</td></tr>
<tr><td>2</td><td>Appalachians</td><td>Between USA(maine)and Canada(Quebec)
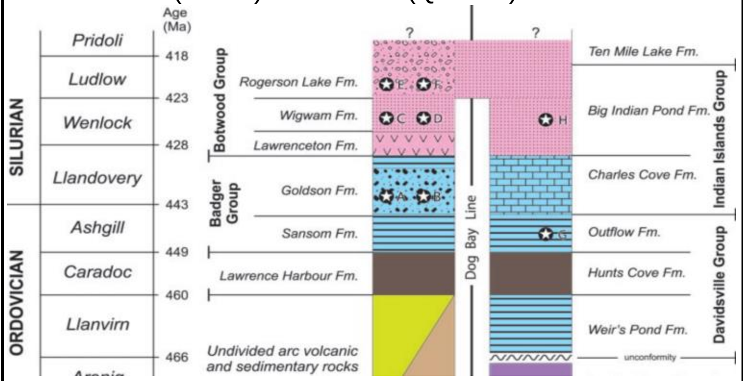
</td><td>Ordovician to Silurian</td><td>Shallow marine and subaerial clastic sedimentary rocks
Subaerial clastic sedimentary rocks
Late Ordovician_black shale, marine graywacke, and conglomerate
Marine limestone conglomerate, subaerial siltstone, and sandstone
443-410 Ma_Shallow marine and subaerial clastic sedimentary rocks
Detritus in Silurian rocks of the Badger Group based on clast Paleocurrents
Late Ordovician unconformity_conformable upwards into the marine Badger and terrigenous Botwood groups in the Early Silurian</td><td>Pollock, J. C., Wilton, D. H. C., Van Staal, C. R. & Morrissey, K. D. U-Pb detrital zircon geochronological constraints on the Early Silurian collision of Ganderia and Laurentia along the Dog Bay Line: The terminal Iapetan suture in the Newfoundland Appalachians. Am. J. Sci. 307, 399-433; https://doi.org/10.2475/02.2007.04 (2007).</td></tr>
<tr><td>3</td><td>Quebec Appalachians</td><td>Between USA(maine) and Canada(Quebec)</td><td>Ordovician to Silurian</td><td>Late Ordovician carbonate and flysch deposits recording the deepening of the Taconian foreland basin

In Newfoundland, Silurian metamorphism is coeval with orogenic activity affecting the Gander and Avalon zones and is hence attributed to a major continent-continent collision between Laurentia and peri-Gondwanan terranes during the Salinian orogeny.</td><td>Castonguay, S. et al. Ordovician and Silurian metamorphic cooling ages along the Laurentian margin of the Quebec Appalachians: Bridging the gap between New England and Newfoundland. Geology 25, 583-586; https://doi.org/10.1130/0091-7613(1997)025<0583:OASMCA>2.3.CO;2 (1997).</td></tr>
<tr><td>4</td><td>Siberia</td><td>Siberian Platform
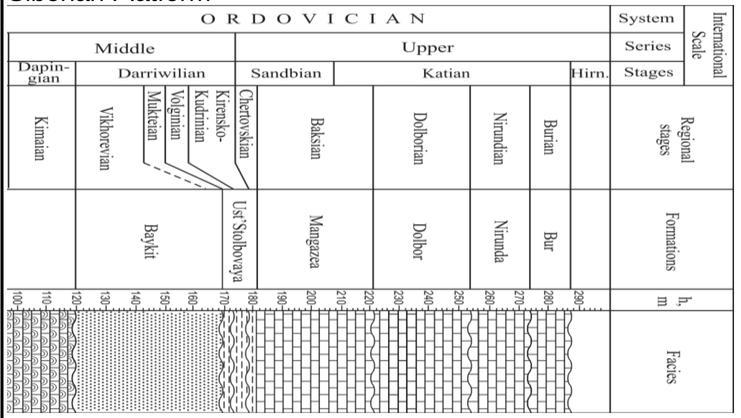
</td><td>Upper Ordovician</td><td>Ordovician succession starts with tropical stromatolite-bearing carbonates which abruptly change to siliciclastic deposits and terminates with cool-water carbonates.
Complete destruction of this long-lived carbonate platform and abrupt change to siliciclastic sedimentation occurred around the Dapingian/Darriwilian boundary when the quartz sandstones of the Baykit Formation were deposited.
The Baykit Sandstone is directly overlain by a relatively thin (10– 17 m) succession of greenish-gray and cherry red siltstones with layers of quartz sandstones and pudding conglomerates with abundant phosphorite grains and nodules.</td><td>Dronov, A. Late Ordovician cooling event: evidence from the Siberian Craton. Palaeogeogr. Palaeoclimatol. Palaeoecol. 389, 87-95; https://doi.org/10.1016/j.palaeo.2013.05.032 (2013).</td></tr>
</table>

No.	Area	Location (Paleocontinent and columnar section image)	Age	Characteristics (Lithology and sedimentary environment)	Reference
5	East Siberia	East Siberia	Early Silurian Ordovician to Silurian	Terrigenous-carbonate deposits<br>Ordovician_sandstone<br>Silurian_limestone, dolostone<br>Sea level change<br>Shallow-water carbonate sedimentation and the accumulation of contrasting terrigenous-carbonate deposits	Kanygin, A.V. et al. Ordovician of the Siberian platform, In: Finney, S. C., and Berry W. B. N.(Eds.), The Ordovician system. Geol. Soc. Am. Spec. Pap. 466, 105-117; https://doi.org/10.1130/2010.2466(07) (2010).
6	Ural	Baltic<br>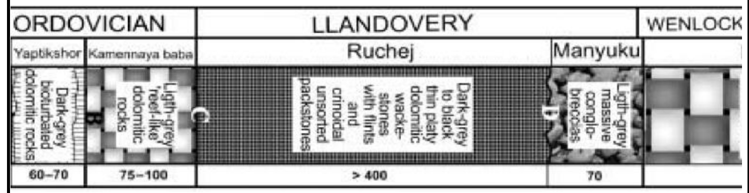	Ordovician to Silurian	Silurian carbonate conglobreccia in Upper Ashgill-Sheinwoodian carbonate succession<br>Sea level drop_Light-grey massive dolomitic microbial and coral-microbial boundstones, skeletal packstones<br>Sea level rise_dark-grey to black thin platy dolomitic mudstones and skeletal wackestones with dark flints of various shape, crinoidal unsorted packstone beds<br>Sea level drop_Light-grey massive dolomitic conglobreccias with clasts of various ages<br>The matrix surrounding clasts is a mixture of sand-sized carbonate particles, The clasts comprise tabular, angular And subangular clasts of dark-grey thin-bedded dolomitic wackestones/ mudstones and microbial boundstones, dark-grey and light-grey microbial boundstones and skeletal packstones.<br>Manyuku Fm. (70m)-carbonate conlobreccia, lacking signs of sub-aerial weathering<br>erosional and transgressive surface before Hirnantian.Late Ordovician_shelf marin, subtidal deep water, lower energy to shelf margin shoal shallow water and high energy<br>Early Silurian_platform ramp, moderate to deep water, lower energy, transgressive surface,<br>Millde Silurian_shelf margin-continental slope submarine moderate water, lower energy	Antoshkina, A. I. Late Ordovician–Early Silurian facies development and environmental changes in the Subpolar Urals. Lethaia 41, 163-171; https://doi.org/10.1111/j.1502-3931.2008.00111.x (2008).
7	Sweden	Baltic<br>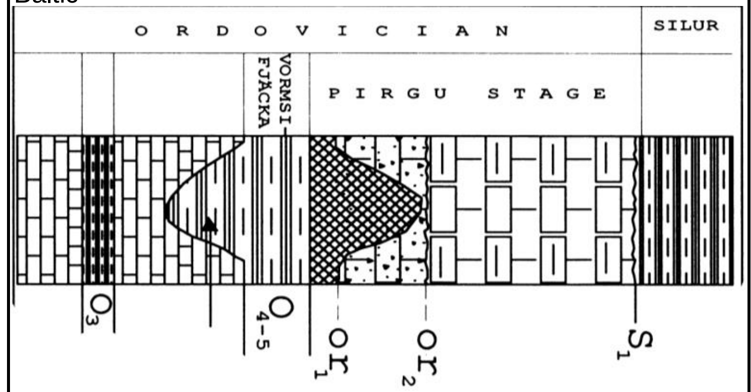	Silurian	(2007)-Carbonate buildup<br>(2000)-Siliciclastic material in Uppermost part of Ordovician<br>(1982)-Paleokarst, tidal erosion surfae and stromatolite ini the Silurian Ejke Fm.<br>Exposed Silurian sequence on gotland (500m thick), shallow marine carbonate sediments of late Llandovery to late Ludlow age. With this background, previous discussions about the lithological succession of the Ordovician–Silurian transitional beds (Kaljo et al. 1988, 1991) and analyses of the distribution of siliciclastic material in the uppermost part of the Ordovician succession (Ainsaar 1995) become most informative. They both support the concept of a marked latest Ordovician drop in sea-level.	Tuuling, I. & Flodén, T. Late Ordovician carbonate buildups and erosional features northeast of Gotland, northern Baltic Sea. GFF 122, 237-249; https://doi.org/10.1080/11035890001222237 (2000).
8	Hunan-Hubei area, South China	South China Block<br>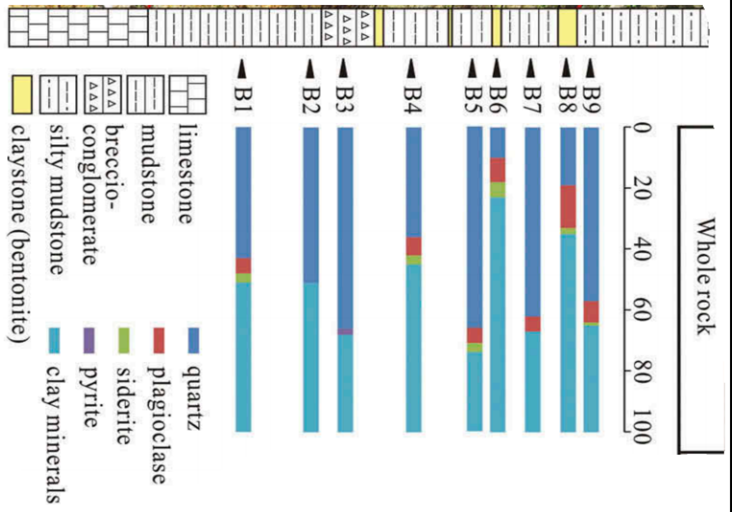	Ordovician to Silurian	Ordovician-Silurian limestone, mudstone, and breccio-conglomerate<br>Late Ordovician global plate reconstruction<br>Subaerial weathering crust<br>Breccio-conglomerate in Wufeng Fm.,<br>Guanyinqiao Fm. Is composed of limestone, argillaceous limestone or carbonaceous mudsone with abundant Hirnanti fauna, a short-lived , widely distributed , relatively shallwo and cool water brachipod assemblage accociated with the low global sea level during the Hirnantian glacial period.	Zheng, B. et al. Nature of the Late Ordovician-Early Silurian Xiaohe section, Hunan-Hubei area, South China: Implications for the Kwangsian Orogeny. Int. Geol. Rev. 62, 1262-1272; https://doi.org/10.1080/00206814.2019.1644541 (2020).
9	Yangtze block, South China	South China Block along the Jiangnan Orogen between the Yangtze and Cathaysia blocks	Cambrian to Silurian	Minor intercalated Cambro-Ordo limestone and Silurian siliciclastic rock<br>Late Ordovician deposition in the SE Yangtze occurred in an active margin setting.<br>Sediments derived from mixed felsic and minor mafic rocks dominated by 455 and 784 Ma.	Yu, T. et al. Provenance of Late Ordovician sedimentary rocks in the SE Yangtze block: Implications for deposition in an active continental margin. Ore Geol. Rev. 127, 1-16; https://doi.org/10.1016/j.oregeorev.2020.103862 (2020).

No.	Area	Location (Paleocontinent and columnar section image)	Age	Characteristics (Lithology and sedimentary environment)	Reference
a	Tarim basin, West China	Tarim basin of Cathayasian Terrane<br>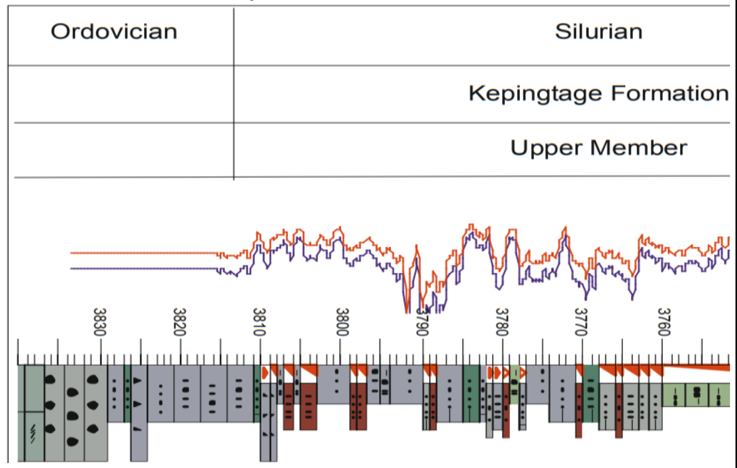 	Ordovician	Dominant Ordovician carbonate sediments to the Silurian-Devonian clastic sediments.<br>Cambrian-Ordovician_marine carbonate<br>Silurian-Devonian_marine clastics<br>Coarse clastic deposits developed in proximal uplift zone.<br>Kepingtage Formation is an important stratum of the Silurian.<br>Paleo-uplift belts_high angular truncated unconformity and intense erosion.<br>Caledonian orogeny (especially, the Late Ordovician period)<br>Unconformity characteristics and distribution of erosion.<br>Parallel unconformity and conformity at high uplift erosion zone and erosion slope zone.<br>During a large sea level rise, the upper member of the Kepingtage Formation began to overlap with the paleo-uplift.	Liu, J. et al. Palaeogeomorphology and its control on the development of sequence stratigraphy and depositional systems of the Early Silurian in the Tarim Basin. Pet. Sci. 7, 311-322; https://doi.org/10.1007/s12182-010-0073-1 (2010).
b	Tarim basin, West China	Tarim basin of Cathayasian Terrane<br>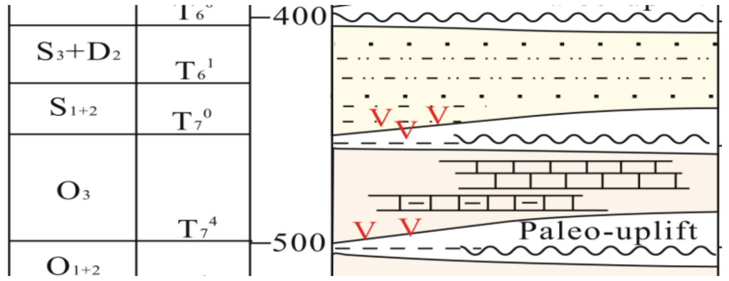 	After Upper Ordovician	Upper Ordovician carbonate deposit and Silurian clastic deposit<br>(Non)angular unconformity<br>Silurian to Devonian_Clastic deposit (quartz sandstone, lithical quartz sandstone)<br>Upper Ordovician_after paleo-uplifting, carbonate platform, and deep turbidite basin<br>Silurian to Devonian_clastic deposit, peripheral foreland basin-cratonic unland depression<br>Local paleo-uplift was the main provenance of the Silurian deposit<br>Silurian sandstones_provenance mainly came from Ordovician Compression and uplift of Altyn orogenic belts, Precambrian old basement, and local uplifts in the basin.	Liu, J. et al. Detrital zircon U-Pb geochronology and its provenance implications on Silurian Tarim basin. J. Earth Sci. 23, 455-475; https://doi.org/10.1007/s12583-012-0268-z (2012).
c	Turkey	Gondwana continent<br>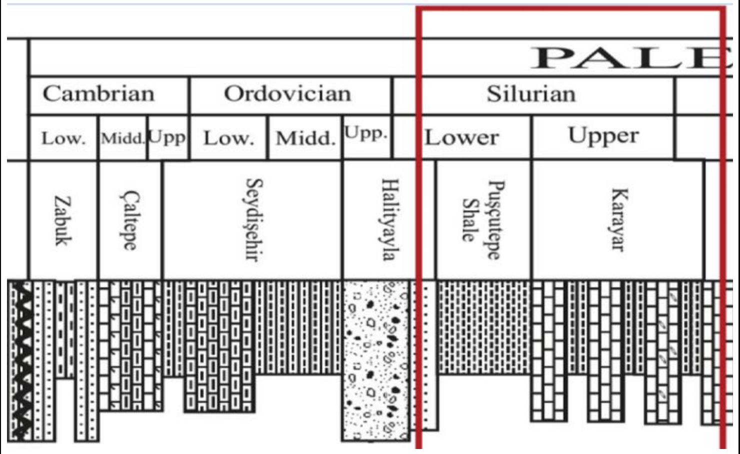 		Late Ordovician-Early Silurian conglomerates<br>Late Ordovician-Early Silurian Halityayla Formation is represented by conglomerates, sandstones, and shales.<br>Thick bedded pebbles and sandstones,(Late Ordovician-Early Silurian)<br>Cambrian-Ordovician_limestone, mid-ordo_siltstone, hirnantian_thick bedded pebble	Doner, Z., Kumral, M., Demirel, I. H. & Hu, Q. Geochemical characteristics of the Silurian shales from the central Taurides, southern Turkey: Organic matter accumulation, preservation and depositional environment modeling. Mar. Pet. Geol. 102, 155-175; https://doi.org/10.1016/j.marpetgeo.2018.12.042 (2019).

No.	Area	Location (Paleocontinent and columnar section image)	Age	Characteristics (Lithology and sedimentary environment)	Reference
d	Pyrenees, Spain	European Peri-Gondwana<br>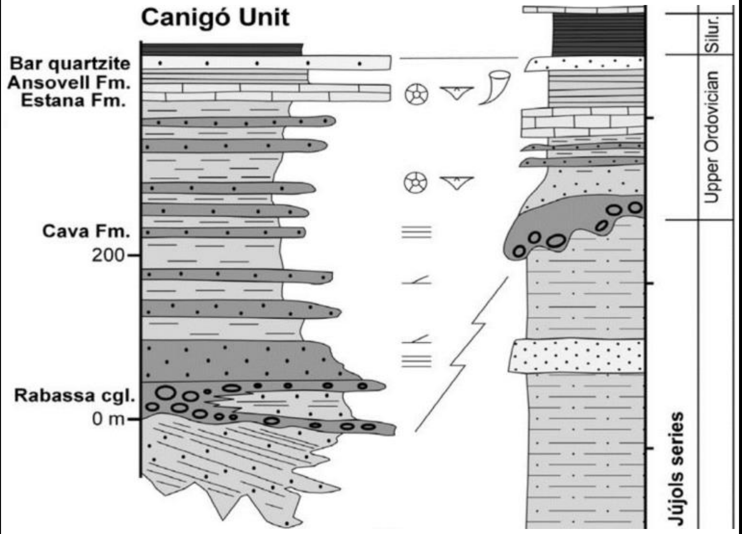	Upper Ordovician	Late Ordovician Rabassa conglomerate unconformable overlie the C-O metasediments<br>Syn-tectonic sediments_Rabassa Conglomerate Formation, the Lower part of the Cava Fms.<br>Post-tectonic sediments_Estana, Ansovell, Bar Fms.<br>Angular unconformity between the Upper Ordovician sediments and the underlying Cambro-Ordovician series.<br>Tilting and truncation of the C-O strata suggest a tectonic uplift, perhaps together with a drop in the eustatic sea level.<br><br>(2010)-The Rabassa Conglomerate Formation is made up of red-purple, unfossiliferous conglomerates and micro conglomerates with lateral thickness variations from a few to 200 metres. Conglomerates are composed of sub-rounded to well-rounded clasts of slates, quartzites and quartz veins that can attain 50 cm in diameter in a green-purple granule-sized matrix.<br>(1970)-Hartevelt attributed the Rabassa conglomerates to the Caradoc.<br>The Rabassa conglomerates unconformably overlie the Cambro-Ordovician metasediments and are overlain by the sandstones of the Cava Formation.<br><br>The Cava Formation is made up of micro conglomerates and feldspathic sandstones in the lower part, followed upwards by shales, siltstones and fine-grained sandstones, green or purple in colour, with strongly bioturbated quartzites in the uppermost part. Thickness changes from 100 to 800 metres and sometimes passes laterally to the Rabassa conglomerates.	Casas, J. M. Ordovician deformations in the Pyrenees: new insights into the significance of pre-Variscan ('sardic') tectonics. Geol. Mag. 147, 674-689; https://doi.org/10.1017/S0016756809990756 (2010).
e	Western Argentina	Gondwana rim<br>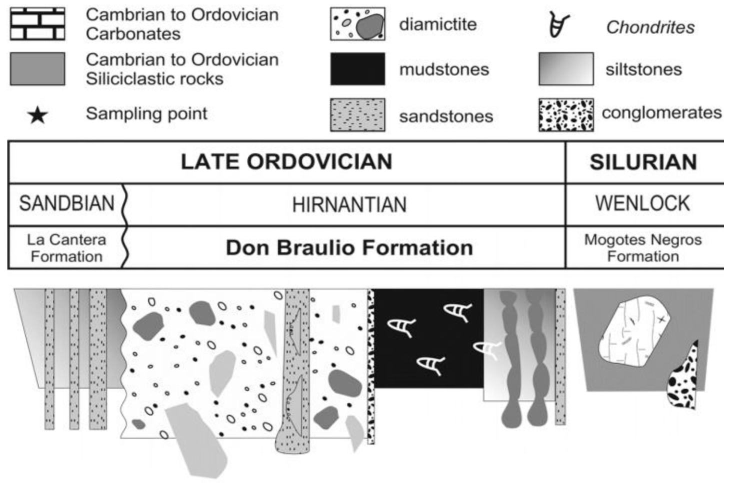	Katian<br>Hirnatian (Upper Ordovician)	Cambro-Ordovician Carbonate and Late-Ordovician conglomerates and diamictite.<br>Late Ordovician to Silurian_above unconformity, diamictite, and partly conglomerate, sandstone.<br>Lower member_ca. 10–15 m thick mud-supported diamictites that frequently contain faceted and striated clasts.<br>These diamictites alternate with channel-like deposits filled with sandstones and grain-supported conglomerates.<br>Upper member_conspicuous pavement–interpreted as a ravinement surface by Astini and Benedetto (1992)– with abundant macrofossils and rounded clasts.<br><br>The succession begins with 10–12 m-thick greenish bioturbated mudstones and silty sandstones. Some deposits contain carbonate cement and common macrofossils (bryozoans, brachiopods, trilobites, bivalves, crinoids, gastropods, and graptolites). The overlying 7–12 m of yellowish fine-grained siltstones rarely contain benthic shells but are rich in graptolites. A single oolitic ferruginous sandstone caps the sequence, showing a sudden shallowing-upward environment. Three ferruginous layers interbedded with the yellowish siltstones occur in other sections (e.g., the La Pola section located 2 km to the south).<br><br>The latter was interpreted to be shallow marine bars, probably representing a parasequence boundary (Astini, 1992).<br>Warm-water carbonates were deposited during the Cambrian and Lower Ordovician, whereas Middle Ordovician units were deposited at mid-latitude (30–35°) locations, including the Katian Sassito limestones (Ernst and Carrera, 2008). The Hirnantian glaciogenic rocks of the Don Braulio Formation represent the last step in the shifting trajectory of the Precordillera terrane (Astini, 1998; Benedetto et al., 2009; Benedetto et al., 2011).	Halpern, K., & Carrera, M. G. Post-glacial Hirnantian (Upper Ordovician) bryozoans from western Argentina: implications for survival and extinction patterns. Ameghiniana 51, 243-253; https://doi.org/10.5710/AMGH.20.03.2014.1840 (2014).

Table S2. Quantitative XRD analyses for major mineral composition of target samples

Sample name	Unit (Fm.)	Major mineral	Minor mineral	I/Ic(Cc)	I/Ic(Do)	I/Ic(Qz)	I/Ic(il)		Calcite	Dolomite	Quartz	Illite
S01+C	Upper carbonate unit (Hoedongri Fm.)	cal	qtz,tae,rut,dol	4.30	0.07	0.13	0.14		91.08	0.62	3.64	4.66
S02+C	Upper carbonate unit (Hoedongri Fm.)	cal	qtz,tae,rut,dol	4.48	0.06	0.14	0.13		90.93	0.57	3.99	4.52
S03+C	Upper carbonate unit (Hoedongri Fm.)	cal	qtz,tae,dol	4.18	0.07	0.13	0.13		91.31	0.62	3.63	4.44
S04+C	Upper carbonate unit (Hoedongri Fm.)	cal	dol,qtz	5.74	0.17	0.05	0.00		97.17	1.57	1.26	0.00
S05+C	Upper carbonate unit (Hoedongri Fm.)	cal	dol,qtz	5.03	0.18	0.04	0.00		97.22	1.61	1.17	0.00
S06+C	Upper carbonate unit (Hoedongri Fm.)	cal	dol	6.07	0.19	0.06	0.00		96.65	1.74	1.62	0.00
S07+C	Upper carbonate unit (Hoedongri Fm.)	cal	dol,qtz,rut	5.28	0.11	0.02	0.00		98.60	0.96	0.44	0.00
S08+C	Upper carbonate unit (Hoedongri Fm.)	cal	dol,qtz,tae	5.17	0.10	0.00	0.06		97.09	0.89	0.00	2.02
S09+C	Upper carbonate unit (Hoedongri Fm.)	cal	dol,qtz,tae,rut	3.57	0.09	0.16	0.02		94.25	0.79	4.44	0.52
S10+C	Upper carbonate unit (Hoedongri Fm.)	dol,cal	qtz,tae	2.84	6.41	0.00	0.00		41.72	58.28	0.00	0.00
S11+C	Upper carbonate unit (Hoedongri Fm.)	dol,cal	qtz,pyr	2.97	8.16	0.00	0.00		25.86	74.14	0.00	0.00
S12+C	Upper carbonate unit (Hoedongri Fm.)	dol,cal	qtz,tae,pyr	2.43	5.72	0.06	0.08		43.73	52.02	1.53	2.72
S13+C	Upper carbonate unit (Hoedongri Fm.)	dol,cal	tae,qtz,pyr	2.43	5.25	0.03	0.09		48.59	47.73	0.79	2.89
S14+C	PBSC unit (Haengmae Fm.)	dol,cal	qtz,tae,pyr	2.28	6.06	0.17	0.14		35.31	55.11	4.71	4.87
S15+C	PBSC unit (Haengmae Fm.)	cal,dol	qtz,tae,rut,pyr	5.12	1.04	0.11	0.13		83.07	9.45	3.03	4.45
S16+C	PBSC unit (Haengmae Fm.)	cal,dol	qtz,tae,rut,pyr	5.18	1.03	0.18	0.09		82.31	9.40	5.13	3.16
S17+C	PBSC unit (Haengmae Fm.)	dol,cal	qtz,tae,rut,clch,pyr	1.41	4.54	0.41	0.07		44.95	41.23	11.30	2.52
S18+C	PBSC unit (Haengmae Fm.)	dol,cal	qtz,tae,rut,clch,pyr	1.50	3.82	0.39	0.07		51.93	34.74	10.81	2.51
S19+C	PBSC unit (Haengmae Fm.)	dol,cal	qtz,tae,rut,clch,pyr	1.41	3.58	0.83	0.08		41.64	32.59	23.17	2.60
S20+C	PBSC unit (Haengmae Fm.)	cal,dol,qtz	tae,clch,rut,pyr	4.77	2.46	0.84	0.14		49.57	22.36	23.43	4.64
S21+C	PBSC unit (Haengmae Fm.)	cal,dol,qtz	clch,tae,rut,pyr	6.21	2.05	0.83	0.16		52.91	18.62	23.00	5.47
S22+C	PBSC unit (Haengmae Fm.)	cal,dol	clch,rut,pyr	2.39	2.27	0.97	0.30		42.27	20.66	26.98	10.10
S23+C	PBSC unit (Haengmae Fm.)	cal,dol	clch,rut,pyr	2.74	2.36	0.45	0.34		54.39	21.45	12.46	11.70
S24+C	PBSC unit (Haengmae Fm.)	cal,dol	qtz,tae,rut,clch,pyr	2.43	1.67	0.44	0.28		63.16	15.17	12.11	9.56
S25+C	PBSC unit (Haengmae Fm.)	cal,dol,qtz	tae	1.83	1.74	1.03	0.11		51.87	15.79	28.50	3.84
S26+C	PBSC unit (Haengmae Fm.)	cal,dol	qtz,tae,pyr,chcl	1.55	1.45	0.84	0.11		59.87	13.14	23.38	3.61
S27+C	PBSC unit (Haengmae Fm.)	cal,dol,qtz	qtz,tae,pyr,chcl,kln	1.66	1.88	1.03	0.15		49.21	17.13	28.53	5.12
S28+C	PBSC unit (Haengmae Fm.)	cal,dol,qtz	qtz,tae,pyr,chcl,kln	1.75	1.64	1.02	0.10		53.29	14.86	28.40	3.45
S29+C	PBSC unit (Haengmae Fm.)	dol,cal	qtz,tae	1.40	7.28	0.10	0.37		18.48	66.14	2.73	12.65
S30+C	PBSC unit (Haengmae Fm.)	cal	qtz,tae,dol	3.59	0.01	0.39	0.23		81.39	0.12	10.77	7.73
S31+C	PBSC unit (Haengmae Fm.)	cal	qtz,tae,hem,rut	3.82	0.00	0.33	0.22		83.52	0.00	9.10	7.38
S32+C	PBSC unit (Haengmae Fm.)	cal	qtz,tae,pyr	3.35	0.01	0.33	0.22		83.28	0.14	9.14	7.44
S33+C	PBSC unit (Haengmae Fm.)	cal,qtz	dol,rut	3.22	0.02	0.90	0.00		74.87	0.16	24.97	0.00
S34+C	PBSC unit (Haengmae Fm.)	cal,qtz	dol,tae,rut	4.05	0.01	1.20	0.21		59.50	0.11	33.30	7.09
S35+C	PBSC unit (Haengmae Fm.)	cal,qtz	tze,rut	5.17	0.02	1.00	0.26		63.19	0.19	27.80	8.82
S36+C	PBSC unit (Haengmae Fm.)	cal,qtz	dol,chcl,rut,sid	6.17	0.11	0.49	0.00		85.47	0.97	13.55	0.00
S37+C	PBSC unit (Haengmae Fm.)	cal	dol,qtz,tae,chcl	6.55	0.11	0.29	0.42		76.87	1.01	7.92	14.21
S38+C	PBSC unit (Haengmae Fm.)	cal,qtz	dol,tae,rut	4.70	0.11	0.45	0.46		70.85	1.04	12.48	15.64
S39+C	PBSC unit (Haengmae Fm.)	cal	ill,qtz,rut	2.89	0.00	0.37	0.34		78.42	0.00	10.15	11.43
S40+C	PBSC unit (Haengmae Fm.)	cal,qtz	tae	1.62	0.00	0.73	0.34		68.36	0.00	20.24	11.40
S41+C	PBSC unit (Haengmae Fm.)	cal,qtz	tae	1.86	0.00	0.26	0.33		92.81	0.00	7.16	0.03
S42+C	PBSC unit (Haengmae Fm.)	cal,qtz	dol,tae,rut	4.49	0.05	0.94	0.35		61.51	0.45	26.14	11.91
S43+C	PBSC unit (Haengmae Fm.)	cal,qtz	dol,tae,rut	5.48	0.02	0.75	0.32		68.10	0.18	20.85	10.87
S44+C	PBSC unit (Haengmae Fm.)	cal,qtz	dol,tae,rut	5.21	0.04	0.82	0.29		67.13	0.32	22.80	9.74
S45+C	PBSC unit (Haengmae Fm.)	cal,dol,qtz	tae,chcl	5.86	1.66	0.85	0.07		58.92	15.09	23.61	2.37
S46+C	PBSC unit (Haengmae Fm.)	cal,dol,qtz	tae,chcl	4.99	2.47	0.77	0.06		54.24	22.42	21.27	2.07
S47+C	PBSC unit (Haengmae Fm.)	cal,dol,qtz	tae,chcl	5.95	0.78	0.74	0.07		70.10	7.08	20.53	2.29
S48+C	Lower carbonate unit (Jeongseon Fm.)	cal	dol,qtz	5.41	0.54	0.21	0.00		89.38	4.88	5.73	0.00
S49+C	Lower carbonate unit (Jeongseon Fm.)	cal	dol,qtz	5.28	0.57	0.03	0.12		89.87	5.16	0.93	4.04
S50+C	Lower carbonate unit (Jeongseon Fm.)	cal,dol	qtz,tae	3.05	1.43	0.19	0.00		81.80	13.03	5.17	0.00
S51+C	Lower carbonate unit (Jeongseon Fm.)	cal,dol	qtz,tae,pyr,clch,rut	6.24	1.57	0.12	0.09		79.07	14.31	3.39	3.22
S52+C	Lower carbonate unit (Jeongseon Fm.)	cal.dol	qtz,tae,rut,pyr	5.62	1.35	0.12	0.08		81.66	12.28	3.33	2.73
S53+C	Lower carbonate unit (Jeongseon Fm.)	cal,dol	pyr	6.01	2.39	0.00	0.00		78.29	21.71	0.00	0.00
S54+C	Lower carbonate unit (Jeongseon Fm.)	cal,dol	pyr	5.91	2.28	0.00	0.00		79.28	20.72	0.00	0.00
S55+C	Lower carbonate unit (Jeongseon Fm.)	cal,dol	pyr	6.59	2.74	0.00	0.00		75.10	24.90	0.00	0.00
S56+C	Lower carbonate unit (Jeongseon Fm.)	cal	dol,qtz,tae	4.89	0.05	0.03	0.08		96.04	0.48	0.89	2.59
S57+C	Lower carbonate unit (Jeongseon Fm.)	cal	dol,qtz,tae	4.66	0.02	0.08	0.00		97.76	0.14	2.10	0.00
S58+C	Lower carbonate unit (Jeongseon Fm.)	cal	dol,qtz,tae	4.70	0.06	0.03	0.08		96.17	0.53	0.74	2.56
S59+C	Lower carbonate unit (Jeongseon Fm.)	cal	dol,taen	6.78	0.67	0.00	0.16		88.34	6.13	0.00	5.53
S60+C	Lower carbonate unit (Jeongseon Fm.)	cal	dol,taen	6.00	0.69	0.00	0.00		93.74	6.26	0.00	0.00
S61+C	Lower carbonate unit (Jeongseon Fm.)	cal	clch	3.59	0.00	0.00	0.00		100.00	0.00	0.00	0.00
S62+C	Lower carbonate unit (Jeongseon Fm.)	cal,qtz	clch	4.83	0.00	0.20	0.00		94.41	0.00	5.59	0.00
S63+C	Lower carbonate unit (Jeongseon Fm.)	cal	clch	4.00	0.00	0.20	0.00		94.42	0.00	5.58	0.00
S64+C	Lower carbonate unit (Jeongseon Fm.)	cal	dol,qtz,tae	3.36	0.15	0.11	0.10		92.09	1.40	3.12	3.38
S65+C	Lower carbonate unit (Jeongseon Fm.)	cal	dol,qtz,tae	3.57	0.19	0.14	0.00		94.49	1.72	3.79	0.00
S66+C	Lower carbonate unit (Jeongseon Fm.)	cal,qtz	tae	5.47	0.00	0.02	0.13		95.02	0.00	0.42	4.56

Table S3-1. Analytical conditions for LG-SIMS measurement of Oxygen isotopes of quartz.

Instrument	Cameca IMS1300-HR^3 large-geometry SIMS, KBSI
Primary ions	Gaussian Cs+ beam, ~3 nA, ~15 m diameter, with 10 m raster, +10 kV potential
Primary beam mass filter aperture	3000 μm
Secondary HV	−10000 V
Transfer optics magnification	× 200
Normal incidence electron gun	Round shape with 120 mm diameter, maximum emission current 2 mA
Entrance slit	70.3 μm
Contrast aperture	400 μm diameter
Field aperture	3000 μm
Energy slit	50 eV bandwidth, with 5 eV gap
Exit slit of multicollector	500 μm
Mass resolving power	5500 (50% peak height) on multi collector
Secondary ion intensity	~3×10^9 cps (^{16}O), ~6×10^6 cps (^{18}O)
Detector and amplifier	Multiple Faraday cups on L'2 ($10^{10}\Omega$) and on H'2 ($10^{11}\Omega$) position
Pre-sputtering	10 μm, 100 seconds
Beam centering	Automatic adjustment of secondary deflectors DTFA-X, DTFA-Y, DTCA-X and DTCA-Y

Table S3-2. LG-SIMS Oxygen isotope analyses data of quartz (including reference materials).

Sample Name	Ipr (nA)	16O/Coeff	1se(%)	18O/Coeff	1se(%)	17O/Coeff	1se(%)	18O/16O	1se(%)	17O/16O	1se(%)	δ18O		δ17O		big delta	corrected value	
		L'2		H'2		FC2		H'2/L'2		FC2/L'2								2s
UnilQ@1	2.5156	1.77E+09	0.3827	3575125	0.3789	679921.3	0.3754	0.00202	0.0115	0.00038	0.0233	7.3544	0.2291	3.3350	0.4655	-0.4893	10.2638	0.2291
UnilQ@2	2.5124	1.687E+09	0.4819	3405396	0.4709	649351.1	0.4842	0.00202	0.0186	0.00038	0.0222	6.7674	0.3728	5.3818	0.4430	1.8628	9.5030	0.3728
UnilQ@3	2.5034	1.672E+09	0.4592	3376639	0.4563	643256.7	0.4524	0.00202	0.0160	0.00038	0.0285	7.1185	0.3208	4.7937	0.5699	1.0921	9.6647	0.3208
S11HM8-1_Q2@01	2.4221	1.655E+09	0.4526	3389743	0.4486	638024.5	0.4556	0.00205	0.0122	0.00039	0.0289	21.2228	0.2434	6.6624	0.5774	-4.3735	22.0676	0.2434
S11HM8-1_Q2@02	2.4273	1.72E+09	0.4343	3518563	0.4233	661806.7	0.4249	0.00205	0.0158	0.00038	0.0234	20.3690	0.3169	5.1194	0.4676	-5.4725	21.1505	0.3169
S11HM8-1_Q2@03	2.4265	1.653E+09	0.4457	3384758	0.4349	635898.3	0.4391	0.00205	0.0163	0.00038	0.0317	21.2158	0.3262	4.7809	0.6340	-6.2514	21.9237	0.3262
S11HM8-1_Q2@04	2.4164	1.711E+09	0.4107	3500393	0.4047	658155	0.4129	0.00205	0.0114	0.00038	0.0371	20.3077	0.2285	4.6973	0.7417	-5.8627	20.9561	0.2285
S11HM8-1_Q2@05	2.4127	1.634E+09	0.4373	3346370	0.4284	627060.2	0.4313	0.00205	0.0111	0.00038	0.0327	21.5141	0.2227	2.4762	0.6547	-8.7111	22.0935	0.2227
S11HM8-1_Q3@01	2.4164	1.494E+09	0.3882	3061038	0.3929	575944.4	0.3956	0.00205	0.0132	0.00039	0.0343	21.6687	0.2636	6.7379	0.6867	-4.5298	22.1926	0.2636
S11HM8-1_Q3@02	2.4118	1.529E+09	0.4427	3130671	0.4415	590094.7	0.4337	0.00205	0.0114	0.00039	0.0293	21.2926	0.2286	8.1676	0.5854	-2.9046	21.7522	0.2286
S11HM8-1_Q3@03	2.4117	1.479E+09	0.5016	3028068	0.5011	571211.2	0.4925	0.00205	0.0092	0.00039	0.0315	20.9206	0.1843	8.6069	0.6307	-2.2718	21.3285	0.1843
S11HM8-1_Q3@04	2.4102	1.601E+09	0.4627	3276357	0.4584	616460.9	0.4496	0.00205	0.0149	0.00039	0.0336	20.7535	0.2984	5.8517	0.6724	-4.9401	21.1017	0.2984
S11HM8-1_Q3@05	2.4076	1.554E+09	0.4522	3184178	0.4437	599149.8	0.4527	0.00205	0.0137	0.00039	0.0302	21.7430	0.2745	6.8661	0.6040	-4.4402	22.0431	0.2745
S11JS8-9D_Q1@01	2.4057	1.817E+09	0.3884	3729168	0.3846	700391.1	0.3848	0.00205	0.0098	0.00039	0.0227	23.6116	0.1960	6.8369	0.4531	-5.4412	23.8565	0.1960
S11JS8-9D_Q1@02	2.4039	1.784E+09	0.5136	3664396	0.5038	687966.9	0.5125	0.00205	0.0158	0.00039	0.0201	24.5317	0.3163	7.3535	0.4029	-5.4030	24.7237	0.3163
S11JS8-9D_Q1@03	2.4001	1.764E+09	0.4212	3622228	0.4090	679567.2	0.4136	0.00205	0.0166	0.00039	0.0282	23.9088	0.3328	6.0311	0.5645	-6.4015	24.0586	0.3328
S11JS8-9D_Q1@04	2.3991	1.774E+09	0.4394	3643602	0.4309	684579.2	0.4243	0.00205	0.0130	0.00039	0.0314	24.5183	0.2596	8.1148	0.6274	-4.6347	24.6195	0.2596
S11JS8-9D_Q1@05	2.3989	1.747E+09	0.4216	3589600	0.4112	675372.6	0.4212	0.00205	0.0164	0.00039	0.0244	24.4230	0.3288	9.4118	0.4882	-3.2881	24.4857	0.3288
UnilQ@3	2.3938	1.637E+09	0.4385	3317905	0.4328	625669.4	0.4299	0.00203	0.0109	0.00038	0.0356	10.4852	0.2190	-2.0529	0.7121	-7.5052	10.4289	0.2190
UnilQ@4	2.3917	1.718E+09	0.4347	3478850	0.4256	656752.1	0.4096	0.00202	0.0130	0.00038	0.0388	9.8718	0.2601	-1.5347	0.7752	-6.6680	9.7778	0.2601
UnilQ@5	2.3903	1.666E+09	0.4237	3374893	0.4199	637495	0.4434	0.00203	0.0121	0.00038	0.0379	9.9855	0.2422	-0.8765	0.7583	-6.0690	9.8562	0.2422
UnilQ@6	2.4005	1.702E+09	0.4574	3445582	0.4532	652508.4	0.4599	0.00202	0.0124	0.00038	0.0397	9.4769	0.2472	1.1818	0.7944	-3.7461	9.3145	0.2472
S9HM10-2-test	2.3530	1.582E+09	0.4347	3244330	0.4338	610011.9	0.4310	0.00205	0.0094	0.00039	0.0209	22.7583	0.1874	7.1226	0.4186	-4.7117	22.3757	0.1874
S9HD12-6-Q@1	2.3697	1.728E+09	0.3979	3552189	0.3914	666031.3	0.3928	0.00206	0.0144	0.00039	0.0358	25.3002	0.2878	6.8032	0.7153	-6.3529	24.9091	0.2878
S9HM10-2-test2	2.3507	1.6E+09	0.3954	3281627	0.3918	613891.9	0.3682	0.00205	0.0115	0.00038	0.0464	22.7414	0.2303	2.0095	0.9276	-9.8160	22.3438	0.2303
S9HM10-2-test3	2.3582	1.72E+09	0.4108	3522549	0.4058	660175.6	0.4194	0.00205	0.0097	0.00038	0.0386	21.2268	0.1948	2.3409	0.7712	-8.6970	20.8268	0.1948
S9HD12-6-Q@02	2.3673	1.708E+09	0.3668	3513330	0.3623	659367.9	0.3826	0.00206	0.0106	0.00039	0.0282	25.8174	0.2115	8.2491	0.5646	-5.1760	25.4171	0.2115
S9HD12-6-Q@03	2.3654	1.718E+09	0.3512	3533569	0.3442	662391.2	0.3397	0.00206	0.0089	0.00039	0.0398	25.5496	0.1776	6.8264	0.7959	-6.4594	25.1513	0.1776
S9HD12-6-Q@04	2.3610	1.705E+09	0.3479	3504485	0.3427	659046.1	0.3461	0.00206	0.0118	0.00039	0.0201	25.1072	0.2357	9.6129	0.4014	-3.4428	24.7122	0.2357
S9HD12-6-Q@05	2.3572	1.65E+09	0.3572	3394790	0.3504	636666.4	0.3523	0.00206	0.0111	0.00039	0.0236	26.0009	0.2224	7.7236	0.4715	-5.7969	25.6114	0.2224
S9HD12-6-Q@06	2.3674	1.647E+09	0.2348	3388230	0.2290	635341	0.2420	0.00206	0.0103	0.00039	0.0273	26.1705	0.2064	7.7351	0.5462	-5.8736	25.7870	0.2064
S9HD12-6-Q@07	2.3648	1.672E+09	0.3238	3440112	0.3175	647262	0.3238	0.00206	0.0141	0.00039	0.0300	25.8278	0.2818	10.8245	0.5999	-2.6059	25.4534	0.2818
S9HD12-6-Q@08	2.3604	1.681E+09	0.3760	3443228	0.3686	650306.6	0.3767	0.00205	0.0143	0.00039	0.0261	21.3206	0.2855	10.1995	0.5227	-0.8872	20.9550	0.2855
S9HD12-6-Q@09	2.3671	1.695E+09	0.3838	3487398	0.3802	655891.2	0.3818	0.00206	0.0089	0.00039	0.0237	25.7815	0.1775	10.3685	0.4730	-3.0378	25.4283	0.1775
S9HD12-6-Q@10	2.3736	1.656E+09	0.3576	3409144	0.3551	640904	0.3457	0.00206	0.0128	0.00039	0.0243	26.4652	0.2559	10.6242	0.4867	-3.1377	26.1262	0.2559
S9HM10-2-Q1@01	2.3570	1.69E+09	0.4101	3460971	0.4070	651909.6	0.3914	0.00205	0.0119	0.00039	0.0308	21.0717	0.2375	7.2704	0.6162	-3.6869	20.7459	0.2375
S9HM10-2-Q1@02	2.3568	1.661E+09	0.4131	3402831	0.4112	638959.6	0.3999	0.00205	0.0079	0.00038	0.0307	21.7998	0.1575	4.8422	0.6135	-6.4937	21.4914	0.1575
S9HM10-2-Q1@03	2.3631	1.7E+09	0.4257	3480527	0.4195	653419.5	0.4271	0.00205	0.0121	0.00038	0.0259	20.9490	0.2423	3.7918	0.5187	-7.1017	20.6565	0.2423
S9HM10-2-Q1@04	2.3623	1.661E+09	0.3633	3403243	0.3535	636496.3	0.3658	0.00205	0.0177	0.00038	0.0392	21.8522	0.3541	0.8823	0.7834	-10.4809	21.5803	0.3541
S9HM10-2-Q1@05	2.3594	1.684E+09	0.3739	3448167	0.3698	642904.4	0.3364	0.00205	0.0127	0.00038	0.0557	21.2832	0.2537	-2.7356	1.1141	-13.8028	21.0298	0.2537
S9HM10-2-Q2@01	2.3491	1.841E+09	0.4326	3763067	0.4286	702248.9	0.4317	0.00204	0.0079	0.00038	0.0676	19.4340	0.1584	-3.6690	1.3511	-13.7747	19.2044	0.1584
S9HM10-2-Q2@02	2.3519	1.662E+09	0.3604	3409750	0.3521	631172.3	0.3007	0.00205	0.0141	0.00038	0.0770	23.1962	0.2812	-8.0372	1.5401	-20.0993	22.9877	0.2812
S9HM10-2-Q2@03	2.3511	1.642E+09	0.3592	3364117	0.3504	621770.3	0.3610	0.00205	0.0176	0.00038	0.0779	22.0537	0.3512	-10.7026	1.5577	-22.1705	21.8720	0.3512
S9HM10-2-Q2@04	2.3512	1.655E+09	0.3537	3391989	0.3516	625028	0.3875	0.00205	0.0085	0.00038	0.0746	22.3005	0.1710	-13.4705	1.4915	-25.0668	22.1425	0.1710
S9HM10-2-Q2@05	2.3481	1.6E+09	0.3694	3284029	0.3665	599277.9	0.3913	0.00205	0.0113	0.00037	0.0753	23.7777	0.2255	-21.5997	1.5060	-33.9641	23.6495	0.2255
S9JS10-10-Q@01	2.3392	1.659E+09	0.3647	3414501	0.3569	626077.2	0.3786	0.00206	0.0172	0.00038	0.0721	26.5425	0.3433	-14.2473	1.4413	-28.0494	26.4458	0.3433
S9JS10-10-Q@02	2.3405	1.656E+09	0.3450	3405031	0.3357	622665.5	0.3116	0.00206	0.0137	0.00038	0.0930	25.6787	0.2740	-17.6867	1.8592	-31.0397	25.6095	0.2740
S9JS10-10-Q@03	2.3418	1.599E+09	0.3548	3289851	0.3508	601130.8	0.3902	0.00206	0.0124	0.00038	0.1029	26.3126	0.2473	-17.8907	2.0570	-31.5733	26.2778	0.2473
S9JS10-10-Q@04	2.3352	1.7E+09	0.3234	3490709	0.3200	646864.9	0.3454	0.00205	0.0106	0.00038	0.0469	24.0106	0.2121	-6.2132	0.9372	-18.6987	24.0056	0.2121
S9JS10-10-Q@05	2.3465	1.63E+09	0.3792	3344689	0.3742	618479.2	0.3646	0.00205	0.0094	0.00038	0.0696	23.5278	0.1872	-8.7826	1.3930	-21.0171	23.5601	0.1872
UnilQ@7	2.3552	1.693E+09	0.3921	3425438	0.3834	643149.7	0.3877	0.00202	0.0154	0.00038	0.0424	9.0684	0.3087	-7.7737	0.8490	-12.4893	10.1509	0.3087
UnilQ@8	2.3530	1.725E+09	0.3534	3487920	0.3432	658348.1	0.3556	0.00202	0.0145	0.00038	0.0328	8.4146	0.2898	-3.1728	0.6556	-7.5484	9.5736	0.2898
UnilQ@9	2.3471	1.742E+09	0.3602	3523599	0.3522	661508.9	0.3144	0.00202	0.0124	0.00038	0.0606	8.4600	0.2474	-8.4496	1.2126	-12.8488	9.6857	0.2474
UnilQ@10	2.3424	1.709E+09	0.3553	3455804	0.3518	649960.3	0.3473	0.00202	0.0124	0.00038	0.0439	8.7318	0.2482	-6.4025	0.8780	-10.9431	10.0367	0.2482
O-Multi-FC-FC2-FC Qtz	2.7965	1.832E+09	0.5103	3676295	0.5125	698846.2	0.4973	0.00201	0.0116	0.00038	0.0447	0.7700	0.2300	-3.6600	0.8900	-4.0600		
O-Multi-FC-FC2-FC Qtz@1	2.8939	2.07E+09	0.3218	4145376	0.3215	789067.4	0.3144	0.00200	0.0087	0.00038	0.0276	-1.4000	0.1700	-4.5000	0.5500	-3.7800		
O-Multi-FC-FC2-FC Qtz@2	2.9324	2.093E+09	0.3043	4186647	0.3052	796342.8	0.3010	0.00200	0.0129	0.00038	0.0262	-2.5700	0.2600	-6.4000	0.5200	-5.0600		
O-Multi-FC-FC2-FC Qtz@3	2.9273	1.864E+09	0.3081	3741268	0.3078	710496.5	0.3079	0.00201	0.0111	0.00038	0.0203	1.1000	0.2200	-4.3300	0.4100	-4.9000		
O-Multi-FC-FC2-FC Qtz@3	2.9273	1.864E+09	0.3081	3741268	0.3078	710496.5	0.3079	0.00201	0.0111	0.00038	0.0203	1.1000	0.2200	-4.3300	0.4100	-4.9000	8.6700	
O-Multi-FC-FC2-FC Qtz@4	2.9208	1.944E+09	0.2999	3901078	0.2988	742401.1	0.2904	0.00201	0.0101	0.00038	0.0189	0.7600	0.2000	-2.5800	0.3800	-2.9700		
s15hd13-7w@1	2.6968	1.802E+09	0.3167	3658051	0.3126	691003.7	0.3066	0.00203	0.0128	0.00038	0.0262	12.2300	0.2600	1.4000	0.5200	-4.9600	20.9000	0.2600
S15HD13-7W@2	2.6941	1.882E+09	0.3038	3815987	0.3085	721269.5	0.3072	0.00203	0.0109	0.00038	0.0230	11.1400	0.2200	0.9100	0.4600	-4.8800	19.8100	0.2200
S15HD13-7W1@1	2.6783	1.877E+09	0.2767	3805687	0.2766	719454.9	0.2715	0.00203	0.0084	0.00038	0.0223	11.3000	0.1700	1.2600	0.4500	-4.6100	19.9700	0.1700
S15HD13-7W1@2	2.6599	1.817E+09	0.2539	3685774	0.2571	694476.5	0.2409	0.00203	0.0101	0.00038	0.0283	11.3700	0.2000	-1.9900	0.5700	-7.9000	20.0400	0.2000
S15HD13-7W1@3	2.6591	1.997E+09	0.3396	4046297	0.3363	766022.6	0.3231	0.00203	0.0127	0.00038	0.0263	10.3900	0.2500	1.7800	0.5300	-3.6300	19.0600	0.2500
S15HD13-7M1@1	2.6351	1.939E+09	0.2881	3940094	0.2889	747170	0.2871	0.00203	0.0064	0.00039	0.0203	13.1700	0.1300	6.2200	0.4100	-0.6300	21.8400	0.1300
S15HD13-7M1@2	2.6293	1.971E+09	0.3407	4005908	0.3463	759124.1	0.3375	0.00203	0.0112	0.00039	0.0203	13.3300	0.2200	5.6900	0.4100	-1.2500	22.0000	0.2200
S15HD13-7M1@3	2.6348	1.899E+09	0.2325	3860468	0.2350	731443.4	0.2259	0.00203	0.0099	0.00039	0.0208	13.8700	0.2000	6.0600	0.4200	-1.1600	22.5500	0.2000
O-Multi-FC-FC2-FC Qtz@4	2.9208	1.944E+09	0.2999	3901078	0.2988	742401.1	0.2904	0.00201	0.0101	0.00038	0.0189	0.7600	0.2000	-2.5800	0.3800	-2.9700	9.4300	0.2000
S15HD13-7M1@4	2.6377	1.912E+09	0.2926	3893628	0.2982	738266.3	0.2885	0.00204	0.0093	0.00039	0.0202	15.4500	0.1900	8.3600	0.4000	0.3300	24.1200	0.1900
S15HD13-7M1-5@01	2.6506	1.908E+09	0.2993	3882958	0.3004	735760.7	0.2929	0.00203	0.0081	0.00039	0.0162	14.7900	0.1600	7.0400	0.3200	-0.6500	23.4600	0.1600
S15HD13-7M2@01	2.6183	1.951E+09	0.3816	3963943	0.3850	747938.9	0.3760	0.00203	0.0128	0.00038	0.0215	13.0200	0.2600	1.0500	0.4300	-5.7200	21.6900	0.2600
S15HD13-7M2@02	2.6304	2.004E+09	0.2837	4068796	0.2874	767403.3	0.2848	0.00203	0.0102	0.00038	0.0286	12.4900	0.2000	0.1000	0.5700	-6.3900	21.1600	0.2000
S15HD13-7M2@03	2.6326	1.927E+09	0.3011	3912003	0.3039	737914.5	0.2985	0.00203	0.0102	0.00038	0.0189	12.5400	0.2000	0.2700	0.3800	-6.2500	21.2100	0.2000
S15HD13-7M2@04	2.6226	1.905E+09	0.3384	3871370	0.3426	731870.6	0.3304	0.00203	0.0093	0.00038	0.0227	13.3700	0.1900	3.3100	0.4500	-3.6400	22.0400	0.1900
S15HD13-7M2@05	2.6118	1.872E+09	0.2982	3801777	0.2963	715754.6	0.2891	0.00203	0.0159	0.00038	0.0310	12.5900	0.3200	-1.6000	0.6200	-8.1400	21.2600	0.3200
S15HD13-7M3@01	2.6090	1.925E+09	0.3065	3913697	0.3061	741404.7	0.3062	0.00203	0.0100	0.00039	0.0162	14.1400	0.2000	6.1400	0.3200	-1.2100	22.8100	0.2000
S15HD13-7M3@02	2.6023	1.976E+09	0.3505	4012039	0.3463	759786.6	0.3308	0.00203	0.0102	0.00038	0.0307	12.7500	0.2000	4.4500	0.6100	-2.1800	21.4200	0.2000
S15HD13-7M3@03	2.6019	1.907E+09	0.3323	3872106	0.3302	733575.4	0.3232	0.00203	0.0101	0.00038	0.0206	12.5900	0.2000	4.6800	0.4100	-1.8600	21.2600	0.2000
S15HD13-7M3@04	2.6070	1.868E+09	0.3150	3791879	0.3140	718495.8	0.3070	0.00203	0.0129	0.00038	0.0189	12.4200	0.2600	4.6800	0.3800	-1.7800	21.0900	0.2600
S15HD13-7M3@05	2.5999	1.876E+09	0.2782	3810333	0.2808	722730.6	0.2727	0.00203	0.0123	0.00039	0.0207	12.7300	0.2500	6.0100	0.4100	-0.6100	21.4000	0.2500
S15HD6-4@01	2.5514	1.87E+09	0.3108	3811740	0.3127	723343.6	0.3089	0.00204	0.0097	0.00039	0.0369	16.3300	0.1900	10.0700	0.7400	1.5800	25.0000	0.1900
S15HD6-4@02	2.5436	1.837E+09	0.3061	3741836	0.3092	709932.1	0.3036	0.00204	0.0133	0.00039	0.0214	16.0300	0.2700	9.5700	0.4300	1.2300	24.7000	0.2700
S15HD6-4@03	2.5542	1.891E+09	0.3220	3850325	0.3258	730144.8	0.3228	0.00204	0.0110	0.00039	0.0417	15.4700	0.2200	8.5000	0.8300	0.4500	24.1400	0.2200
S15HD6-4@04	2.5774	1.872E+09	0.2619	3815843	0.2662	723524.8	0.2679	0.00204	0.0119	0.00039	0.0200	16.7700	0.2400	9.6700	0.4000	0.9500	25.4400	0.2400
S15HD6-4@05	2.5678	1.868E+09	0.3137	3806610	0.3208	721388.9	0.3198	0.00204	0.0119	0.00039	0.0237	16.0300	0.2400	8.4000	0.4700	0.0600	24.7000	0.2400
S15HD12-6_1@01	2.5607	1.891E+09	0.3247	3854911	0.3276	730643.4	0.3276	0.00204	0.0114	0.00039	0.0142	16.5200	0.2300	9.0300	0.2800	0.4300	25.1900	0.2300
S15HD12-6_1@02	2.5610	1.936E+09	0.3216	3940246	0.3221	747230.6	0.3194	0.00204	0.0131	0.00039	0.0210	14.9400	0.2600	8.0200	0.4200	0.2500	23.6100	0.2600
S15HD12-6_1@03	2.5645	1.887E+09	0.2981	3848164	0.2972	729677.6	0.2905	0.00204	0.0099	0.00039	0.0207	16.8700	0.2000	9.8100	0.4100	1.0400	25.5400	0.2000
S15HD12-6_2@01	2.5671	1.914E+09	0.3054	3902355	0.3046	739519.1	0.3049	0.00204	0.0119	0.00039	0.0217	16.9100	0.2400	9.2500	0.4300	0.4600	25.5800	0.2400
S15HD12-6_2@02	2.5709	1.936E+09	0.3167	3948912	0.3185	749153.4	0.3132	0.00204	0.0104	0.00039	0.0189	17.2700	0.2100	10.7100	0.3800	1.7300	25.9400	0.2100
S15HD12-6_2@03	2.5737	1.899E+09	0.2906	3875095	0.2933	734721.2	0.2900	0.00204	0.0132	0.00039	0.0214	17.6800	0.2600	10.5200	0.4300	1.3300	26.3500	0.2600
S15HD6-2_1@01	2.5719	2.103E+09	0.3109	4284858	0.3154	811908.8	0.2985	0.00204	0.0088	0.00039	0.0242	16.0700	0.1800	8.3100	0.4800	-0.0500	24.7400	0.1800
S15HD6-2_1@02	2.5755	1.894E+09	0.3110	3856521	0.3145	730609.4	0.3063	0.00204	0.0119	0.00039	0.0225	15.5900	0.2400	7.6400	0.4500	-0.4700	24.2600	0.2400
S15HD6-2_1@03	2.5702	1.925E+09	0.2635	3922182	0.2688	743345.8	0.2626	0.00204	0.0103	0.00039	0.0277	15.8500	0.2100	8.3000	0.5500	0.0600	24.5200	0.2100
S15HD6-2_1@04	2.5624	1.889E+09	0.2799	3854439	0.2800	731228.1	0.2808	0.00204	0.0098	0.00039	0.0221	17.4600	0.2000	10.8900	0.4400	1.8100	26.1300	0.2000
S15HD6-2_1@05	2.5575	1.923E+09	0.3356	3923658	0.3422	744074.4	0.3337	0.00204	0.0118	0.00039	0.0134	17.6100	0.2400	10.6600	0.2700	1.5000	26.2800	0.2400
S15HD924BRul@01	2.5603	1.862E+09	0.2448	3763934	0.2499	716467.2	0.2429	0.00202	0.0122	0.00038	0.0226	8.0300	0.2400	4.9000	0.4500	0.7300	16.7000	0.2400
S15HD924BRul@02	2.5510	1.901E+09	0.2446	3818387	0.2452	728677.3	0.2459	0.00201	0.0126	0.00038	0.0203	1.7400	0.2500	1.1700	0.4100	0.2600	10.4100	0.2500
S15HD924BRul@03	2.5446	1.923E+09	0.3414	3879760	0.3407	740009.9	0.3412	0.00202	0.0122	0.00038	0.0215	6.0600	0.2400	4.9600	0.4300	1.8100	14.7300	0.2400
S15HD13-8@01	2.5593	1.861E+09	0.3144	3783095	0.3165	715243.1	0.2993	0.00203	0.0095	0.00038	0.0253	13.6900	0.1900	3.7200	0.5100	-3.4000	22.3600	0.1900
S15HD13-8@02	2.5584	1.823E+09	0.3020	3719566	0.2991	703537.4	0.2958	0.00204	0.0112	0.00039	0.0240	17.2600	0.2200	7.6800	0.4800	-1.3000	25.9300	0.2200
S15HD13-8@03	2.5607	1.854E+09	0.2961	3783356	0.2994	714824.5	0.2938	0.00204	0.0105	0.00039	0.0213	17.8600	0.2100	7.1800	0.4300	-2.1100	26.5300	0.2100
S15HD6-2_2@01	2.5432	1.885E+09	0.2863	3845472	0.2882	728175	0.2855	0.00204	0.0134	0.00039	0.0183	17.4400	0.2700	9.0000	0.3700	-0.0700	26.1100	0.2700
S15HD6-2_2@02	2.5373	1.894E+09	0.3068	3861446	0.3060	731906.7	0.3121	0.00204	0.0103	0.00039	0.0168	16.5800	0.2100	9.1100	0.3400	0.4900	25.2500	0.2100
S15HD6-2_2@03	2.5375	1.878E+09	0.3141	3833781	0.3159	726233.2	0.3079	0.00204	0.0111	0.00039	0.0250	18.2100	0.2200	10.1500	0.5000	0.6800	26.8800	0.2200
S15HD12-6_3@01	2.5358	1.95E+09	0.3207	3978454	0.3226	752886.8	0.3187	0.00204	0.0096	0.00039	0.0207	17.6700	0.1900	8.6000	0.4100	-0.5900	26.3400	0.1900
S15HD12-6_3@02	2.5352	1.94E+09	0.3167	3961937	0.3196	750034.1	0.3170	0.00204	0.0129	0.00039	0.0215	18.3000	0.2600	9.5900	0.4300	0.0800	26.9700	0.2600
S15HD12-6_3@03	2.5325	1.94E+09	0.3399	3956807	0.3423	748999.5	0.3467	0.00204	0.0102	0.00039	0.0245	17.0100	0.2000	8.2200	0.4900	-0.6200	25.6800	0.2000
S15HD13-7M4@01	2.5543	1.917E+09	0.3160	3899453	0.3155	739494.3	0.3162	0.00203	0.0122	0.00039	0.0226	14.3600	0.2400	7.4400	0.4500	-0.0300	23.0300	0.2400
S15HD13-7M4@02	2.5529	1.903E+09	0.2754	3870241	0.2778	733763.1	0.2754	0.00203	0.0114	0.00039	0.0177	14.0900	0.2300	6.9100	0.3500	-0.4200	22.7600	0.2300
S15HD13-7M4@03	2.5516	1.937E+09	0.3454	3935800	0.3483	746226.4	0.3466	0.00203	0.0087	0.00039	0.0194	13.4600	0.1700	6.3300	0.3900	-0.6700	22.1300	0.1700
S15HD13-7M4@04	2.5487	1.912E+09	0.3167	3892615	0.3202	737767.7	0.3216	0.00204	0.0103	0.00039	0.0195	15.1600	0.2100	7.6500	0.3900	-0.2400	23.8300	0.2100
S15HD13-7M4@05	2.5422	1.841E+09	0.2898	3747217	0.2880	710477.3	0.2876	0.00204	0.0108	0.00039	0.0212	14.9100	0.2200	7.7800	0.4200	0.0200	23.5800	0.2200
S15HD13-7W1@04	2.5450	1.989E+09	0.3691	4034506	0.3670	765898.4	0.3660	0.00203	0.0106	0.00039	0.0206	11.8200	0.2100	5.9600	0.4100	-0.1900	20.4900	0.2100
S15HD13-7W1@05	2.5564	1.915E+09	0.3440	3889763	0.3437	737977.5	0.3539	0.00203	0.0127	0.00039	0.0181	12.7500	0.2500	6.2700	0.3600	-0.3600	21.4200	0.2500
S15HD13-8@04	2.5713	2.778E+09	0.3483	5641986	0.3383	1067537	0.3467	0.00203	0.0124	0.00038	0.0167	12.8400	0.2500	3.6600	0.3300	-3.0200	21.5100	0.2500
nbs18@	2.5366	1.922E+09	0.4133	3849125	0.4197	735671	0.4043	0.00200	0.0119	0.00038	0.0193	-1.1600	0.2400	-0.1900	0.3900	0.4100		
nbs18@1	2.5314	1.919E+09	0.2533	3841977	0.2572	734250.3	0.2426	0.00200	0.0104	0.00038	0.0198	-1.8000	0.2100	-0.9000	0.4000	0.0300	11.0800	
S17JS13-7W@1	2.5354	1.928E+09	0.2429	3896513	0.2459	736841.1	0.2343	0.00202	0.0147	0.00038	0.0285	8.0100	0.2900	-1.7000	0.5700	-5.8700	19.0900	0.2900
S17JS13-7W@2	2.5337	1.91E+09	0.3255	3863939	0.3292	732142.8	0.3231	0.00202	0.0133	0.00038	0.0215	8.9800	0.2700	1.2500	0.4300	-3.4200	20.0600	0.2700
S17JS13-7D@01	2.5608	1.929E+09	0.2321	3917267	0.2354	743338.4	0.2273	0.00203	0.0101	0.00039	0.0224	12.9800	0.2000	6.7100	0.4500	-0.0500	24.0600	0.2000
S17JS13-7D@02	2.5657	2.026E+09	0.1461	4120450	0.1504	783037.9	0.1419	0.00203	0.0103	0.00039	0.0225	14.2100	0.2100	9.4000	0.4500	2.0100	25.2900	0.2100
S17JS13-7D@03	2.5675	1.833E+09	0.2700	3733119	0.2680	709501.1	0.2648	0.00204	0.0118	0.00039	0.0183	15.9300	0.2400	11.2100	0.3700	2.9200	27.0100	0.2400
S17JS13-7D@04	2.5618	2.039E+09	0.1510	4148283	0.1547	787650	0.1532	0.00203	0.0100	0.00039	0.0128	14.6500	0.2000	8.9700	0.2600	1.3500	25.7300	0.2000
S17JS13-7D@05	2.5575	1.972E+09	0.1484	4012494	0.1554	761271.9	0.1538	0.00203	0.0144	0.00039	0.0233	14.5600	0.2900	8.0900	0.4700	0.5200	25.6400	0.2900
S17JS13-7D@07	2.5593	2.712E+09	0.3559	5499566	0.3499	1043175	0.3407	0.00203	0.0109	0.00038	0.0238	11.4000	0.2200	4.7300	0.4800	-1.2000	22.4800	0.2200
S17JS13-7D@06	2.5457	2.037E+09	0.2358	4130197	0.2412	784656	0.2285	0.00203	0.0127	0.00039	0.0260	11.2600	0.2500	6.1600	0.5200	0.3100	22.3300	0.2500
S17JS13-7D@08	2.5558	2.367E+09	0.3203	4800305	0.3256	908628.9	0.3206	0.00203	0.0097	0.00038	0.0192	11.5300	0.1900	2.7500	0.3800	-3.2500	22.6000	0.1900
S17JS924BRimbr1-1@01	2.5617	1.856E+09	0.2448	3760778	0.2513	706034.3	0.2500	0.00203	0.0104	0.00038	0.0340	10.5400	0.2100	-6.4300	0.6800	-11.9100	21.6200	0.2100
S17JS924BRimbr1-1@02	2.5605	1.906E+09	0.2992	3850597	0.3033	724277	0.2834	0.00202	0.0114	0.00038	0.0312	7.4300	0.2300	-7.5900	0.6200	-11.4500	18.5100	0.2300
S17JS924BRimbr1-1@03	2.5575	1.79E+09	0.3087	3630780	0.3060	686671.8	0.2960	0.00203	0.0107	0.00038	0.0270	11.5900	0.2100	1.9600	0.5400	-4.0700	22.6600	0.2100
S17JS924BRimbr2-1@01	2.5551	1.777E+09	0.2760	3598724	0.2777	682090.4	0.2746	0.00203	0.0087	0.00038	0.0234	10.1800	0.1700	2.7400	0.4700	-2.5500	21.2600	0.1700
S17JS924BRimbr2-1@02	2.5532	1.836E+09	0.2759	3718134	0.2780	701326.7	0.2640	0.00203	0.0126	0.00038	0.0397	10.0500	0.2500	-2.2100	0.7900	-7.4400	21.1300	0.2500
S17JS924BRimbr2-1@03	2.5561	1.774E+09	0.3016	3596800	0.2951	681739.8	0.2909	0.00203	0.0156	0.00038	0.0258	11.2600	0.3100	3.8300	0.5200	-2.0200	22.3400	0.3100

Sample Name	Ipr (nA)	16O/Coeff	1se(%)	18O/Coeff	1se(%)	17O/Coeff	1se(%)	18O/16O	1se(%)	17O/16O	1se(%)	δ18O		δ17O		big delta	corrected value	
S17JS924BRimbr3-1@01	2.5513	1.835E+09	0.3148	3706349	0.3183	692479.1	0.3193	0.00202	0.0108	0.00038	0.0378	7.4300	0.2200	-14.2400	0.7600	-18.1100	18.5000	0.2200
S17JS924BRimbr3-1@02	2.5525	1.763E+09	0.2996	3574302	0.2956	676091.2	0.2735	0.00203	0.0118	0.00038	0.0446	11.1400	0.2400	1.6800	0.8900	-4.1200	22.2200	0.2400
S17JS924BRimbr3-1@03	2.5507	1.841E+09	0.2602	3737683	0.2652	706582.1	0.2597	0.00203	0.0113	0.00038	0.0247	12.6600	0.2300	2.5800	0.4900	-4.0000	23.7400	0.2300
S17JS924BRimbr4@01	2.5386	1.815E+09	0.2923	3677403	0.2920	698176.3	0.2770	0.00203	0.0117	0.00038	0.0222	10.5500	0.2300	4.8100	0.4400	-0.6800	21.6300	0.2300
S17JS924BRimbr5@01	2.5550	1.861E+09	0.3492	3758825	0.3471	701831.4	0.3407	0.00202	0.0112	0.00038	0.0288	7.0900	0.2200	-15.2100	0.5800	-18.8900	18.1700	0.2200
S17JS924BRimbr6@01	2.5406	1.855E+09	0.2674	3766937	0.2690	714471.4	0.2623	0.00203	0.0129	0.00039	0.0298	12.9700	0.2600	6.2100	0.6000	-0.5300	24.0500	0.2600
S17JS924BRimbr7-1@01	2.5343	1.843E+09	0.3141	3728147	0.3150	702692.8	0.3191	0.00202	0.0129	0.00038	0.0326	8.5500	0.2600	-4.4500	0.6500	-8.8900	19.6300	0.2600
S17JS924BRimbr7-1@02	2.5295	1.843E+09	0.3033	3727697	0.3039	697997.9	0.2872	0.00202	0.0136	0.00038	0.0392	8.4600	0.2700	-11.0500	0.7800	-15.4500	19.5400	0.2700
S17JS924BRimbr7-1@03	2.5273	1.805E+09	0.2984	3647506	0.3018	682213.8	0.2777	0.00202	0.0107	0.00038	0.0411	7.7600	0.2100	-12.8500	0.8200	-16.8900	18.8300	0.2100
S17JS8-6D@01	2.5478	1.878E+09	0.3116	3779366	0.3204	707720.3	0.3118	0.00201	0.0120	0.00038	0.0266	3.8300	0.2400	-15.5400	0.5300	-17.5300	14.9100	0.2400
S17JS8-6D@02	2.5401	1.786E+09	0.2505	3601933	0.2532	679633.1	0.2415	0.00202	0.0128	0.00038	0.0359	5.8900	0.2600	-6.0000	0.7200	-9.0600	16.9700	0.2600
S17JS8-6D@03	2.5374	1.897E+09	0.1781	3827552	0.1778	719844.7	0.1652	0.00202	0.0120	0.00038	0.0342	6.4100	0.2400	-8.7300	0.6800	-12.0700	17.4900	0.2400
S17JS8-6D@04	2.5269	2.444E+09	0.4151	4911932	0.4114	929319.2	0.4047	0.00201	0.0092	0.00038	0.0305	2.4800	0.1800	-6.6900	0.6100	-7.9800	13.5500	0.1800
S17JS8-6D@05	2.5254	2.271E+09	0.4160	4571807	0.4099	864440.8	0.4065	0.00201	0.0107	0.00038	0.0270	3.9100	0.2100	-5.8800	0.5400	-7.9200	14.9900	0.2100
S17JS8-6D@06	2.5223	2.538E+09	0.3743	5105689	0.3693	965942.3	0.3723	0.00201	0.0095	0.00038	0.0269	3.4400	0.1900	-5.7800	0.5400	-7.5700	14.5200	0.1900
S17JS8-6D@07	2.5202	1.79E+09	0.2482	3602165	0.2544	679103.9	0.2458	0.00201	0.0133	0.00038	0.0335	3.8600	0.2700	-8.8400	0.6700	-10.8500	14.9400	0.2700
S17JS8-6D@08	2.5115	1.921E+09	0.2099	3879843	0.2102	737887	0.2089	0.00202	0.0115	0.00038	0.0315	7.3400	0.2300	3.3400	0.6300	-0.4800	18.4200	0.2300
S17JS8-6W1-1@03	2.5221	1.803E+09	0.2926	3665570	0.2895	690482.2	0.2831	0.00203	0.0113	0.00038	0.0335	14.1100	0.2300	0.4500	0.6700	-6.8900	25.1900	0.2300
S17JS8-6W1-1@02	2.5108	1.787E+09	0.2628	3633779	0.2644	683118.1	0.2635	0.00203	0.0080	0.00038	0.0294	14.2000	0.1600	-1.4800	0.5900	-8.8600	25.2800	0.1600
S17JS8-6W1-1@01	2.5194	1.781E+09	0.3068	3619410	0.3129	679527.3	0.3064	0.00203	0.0121	0.00038	0.0217	13.2200	0.2400	-3.7500	0.4300	-10.6200	24.2900	0.2400
S17JS8-6W2-1@03	2.5216	1.973E+09	0.2987	3997067	0.2971	744423.7	0.2747	0.00203	0.0134	0.00038	0.0527	10.5200	0.2700	-14.3500	1.0500	-19.8200	21.6000	0.2700
S17JS8-6W2-1@02	2.5314	1.799E+09	0.2836	3649405	0.2848	683335.4	0.2696	0.00203	0.0153	0.00038	0.0312	11.7600	0.3100	-7.8200	0.6200	-13.9400	22.8400	0.3100
S17JS8-6W2-1@01	2.5303	1.87E+09	0.2954	3793507	0.2962	708824.1	0.2933	0.00203	0.0093	0.00038	0.0279	11.8100	0.1900	-9.8700	0.5600	-16.0100	22.8800	0.1900
S17JS8-6W3-1@03	2.5195	986654300	0.0130	1632552	0.1303	211855.1	0.3281	0.00165	0.1362	0.00021	0.3353	-174.8300	2.7200	-439.1900	6.7100	-348.2800	-163.7500	2.7200
S17JS8-6W3-1@02	2.5220	759691900	0.0307	1162681	0.1158	155870.4	0.2352	0.00153	0.1240	0.00021	0.2412	-236.7500	2.4800	-464.1200	4.8200	-341.0100	-225.6700	2.4800
S17JS8-6W3-1@01	2.5219	650952700	0.0468	1030606	0.1461	142702.8	0.3564	0.00158	0.1809	0.00022	0.3892	-210.4300	3.6200	-427.4300	7.7800	-318.0000	-199.3500	3.6200
S17JS8-6W4-1@03	2.4750	29534910	0.1720	56960.23	0.6691	10834.53	1.2426	0.00193	0.6257	0.00037	1.2270	-38.2400	12.5100	-41.9000	24.5400	-22.0200	-27.1600	12.5100
S17JS8-6W4-1@02	2.4656	34785560	0.0445	66714.17	0.6208	12983.42	1.0276	0.00192	0.6117	0.00037	1.0305	-43.5600	12.2300	-25.1700	20.6100	-2.5100	-32.4800	12.2300
S17JS8-6W4-1@01	2.4766	81686120	0.1067	159215.9	0.2102	29988.93	0.4568	0.00195	0.2113	0.00037	0.4716	-27.9600	4.2300	-41.1300	9.4300	-26.5900	-16.8800	4.2300
S17JS8-6W5@01	2.4788	1.929E+09	0.3108	3921400	0.3152	740028.3	0.3099	0.00203	0.0126	0.00038	0.0166	13.5500	0.2500	1.7200	0.3300	-5.3200	24.6300	0.2500
S17JS8-6W6@01	2.4666	1.782E+09	0.3048	3619233	0.3034	682758.7	0.3016	0.00203	0.0128	0.00038	0.0203	12.7400	0.2600	0.5600	0.4100	-6.0600	23.8200	0.2600
S17JS10-10@08	2.4826	1.82E+09	0.2851	3666592	0.2940	698070	0.2885	0.00201	0.0123	0.00038	0.0217	4.5800	0.2500	1.6600	0.4300	-0.7300	15.6600	0.2500
S17JS10-10@07	2.4798	1.815E+09	0.3159	3669104	0.3198	697792.2	0.3105	0.00202	0.0134	0.00038	0.0251	8.2900	0.2700	4.2700	0.5000	-0.0400	19.3700	0.2700
S17JS10-10@06	2.4607	1.797E+09	0.3117	3651305	0.3130	692310.7	0.3126	0.00203	0.0112	0.00039	0.0241	13.4700	0.2200	6.3700	0.4800	-0.6300	24.5400	0.2200
S17JS10-10@05	2.4551	1.783E+09	0.2845	3606894	0.2857	685438.1	0.2770	0.00202	0.0117	0.00038	0.0270	8.9100	0.2300	4.1100	0.5400	-0.5200	19.9800	0.2300
S17JS10-10@04	2.4599	1.761E+09	0.2804	3544095	0.2803	675554.5	0.2668	0.00201	0.0119	0.00038	0.0301	3.5800	0.2400	1.8600	0.6000	0.0000	14.6600	0.2400
S17JS10-10@03	2.4589	1.83E+09	0.3173	3719572	0.3160	704731.8	0.3126	0.00203	0.0138	0.00039	0.0223	13.6900	0.2800	5.8500	0.4500	-1.2700	24.7700	0.2800
S17JS10-10@02	2.4555	1.802E+09	0.3260	3657232	0.3282	692808.5	0.3274	0.00203	0.0077	0.00038	0.0259	12.0300	0.1500	4.0300	0.5200	-2.2200	23.1100	0.1500
S17JS10-10@01	2.4557	1.795E+09	0.2642	3634006	0.2695	689254.8	0.2586	0.00202	0.0109	0.00038	0.0199	9.8500	0.2200	3.1100	0.4000	-2.0100	20.9300	0.2200
S17JS13-7W@09	2.3688	1.835E+09	0.2980	3712260	0.2995	704346.4	0.2940	0.00202	0.0146	0.00038	0.0284	8.6900	0.2900	2.3100	0.5700	-2.2100	19.7700	0.2900
S17JS13-7W@08	2.3856	1.799E+09	0.3165	3638942	0.3182	690427.6	0.3087	0.00202	0.0154	0.00038	0.0323	8.5700	0.3100	2.1800	0.6500	-2.2700	19.6500	0.3100
S17JS13-7W@07	2.4209	1.846E+09	0.2900	3735912	0.2936	707944.7	0.2762	0.00202	0.0130	0.00038	0.0284	9.0600	0.2600	1.4300	0.5700	-3.2800	20.1400	0.2600
S17JS13-7W@06	2.4777	1.878E+09	0.2750	3802039	0.2734	721725.1	0.2775	0.00202	0.0110	0.00038	0.0250	9.6900	0.2200	3.7800	0.5000	-1.2600	20.7700	0.2200
S17JS13-7W@05	2.4875	1.926E+09	0.3125	3893416	0.3137	738316	0.3142	0.00202	0.0144	0.00038	0.0258	8.1800	0.2900	1.2600	0.5200	-3.0000	19.2600	0.2900
S17JS13-7W@04	2.4867	1.892E+09	0.3099	3827109	0.3168	725375.9	0.3044	0.00202	0.0131	0.00038	0.0220	8.7700	0.2600	1.3400	0.4400	-3.2200	19.8500	0.2600
S17JS13-7W@03	2.4942	1.884E+09	0.2761	3805282	0.2852	721445.8	0.2741	0.00202	0.0169	0.00038	0.0191	7.3300	0.3400	0.2000	0.3800	-3.6100	18.4100	0.3400
S17JS13-7W@02	2.4914	1.92E+09	0.3339	3880299	0.3331	735739.6	0.3320	0.00202	0.0073	0.00038	0.0213	7.6700	0.1500	0.6200	0.4300	-3.3600	18.7400	0.1500
S17JS13-7W@01	2.4985	1.895E+09	0.3219	3830757	0.3243	725760.4	0.3239	0.00202	0.0120	0.00038	0.0203	8.0300	0.2400	0.1800	0.4100	-4.0000	19.1100	0.2400
nbs@01	2.5607	1.878E+09	0.2879	3763553	0.2848	717304.2	0.2685	0.00200	0.0098	0.00038	0.0256	-0.8500	0.2000	-2.6700	0.5100	-2.2300	10.4100	0.2000
nbs@2	2.5526	1.877E+09	0.2934	3760272	0.2919	717571.1	0.2858	0.00200	0.0115	0.00038	0.0250	-0.7700	0.2300	-1.3600	0.5000	-0.9600		
S17JS13-7W@10	2.5492	1.863E+09	0.2345	3771914	0.2361	712005.4	0.2261	0.00202	0.0110	0.00038	0.0348	9.5400	0.2200	-1.9700	0.7000	-6.9300	19.9500	0.2200
S17JS13-7W@11	2.5298	1.901E+09	0.2892	3849307	0.2936	727901.7	0.2860	0.00203	0.0128	0.00038	0.0219	10.0700	0.2600	0.3200	0.4400	-4.9200	20.4800	0.2600
S17JS8-6D@9	2.5269	1.765E+09	0.2273	3552380	0.2261	672029.8	0.2331	0.00201	0.0124	0.00038	0.0330	3.7200	0.2500	-5.5700	0.6600	-7.5000	14.1300	0.2500
S17JS8-6D@10	2.5238	2.487E+09	0.3229	5016977	0.3208	950449.2	0.3181	0.00202	0.0087	0.00038	0.0212	5.9800	0.1700	-1.9100	0.4200	-5.0100	16.3900	0.1700
S17JS8-6W7@01	2.5322	1.759E+09	0.2354	3575383	0.2354	670880.9	0.2352	0.00203	0.0139	0.00038	0.0347	13.4300	0.2800	-4.1100	0.6900	-11.0900	23.8400	0.2800
S17JS8-6W8@01	2.5143	1.734E+09	0.2408	3526063	0.2384	663602.8	0.2380	0.00203	0.0138	0.00038	0.0274	13.9100	0.2800	-0.6600	0.5500	-7.8900	24.3200	0.2800
S17JS8-6W8@02	2.5125	1.706E+09	0.2347	3478888	0.2374	657443.3	0.2371	0.00204	0.0134	0.00039	0.0538	16.8800	0.2700	6.4300	1.0800	-2.3500	27.2900	0.2700
S17JS8-6W8@03	2.5183	1.772E+09	0.2319	3598773	0.2299	674815.2	0.2360	0.00203	0.0162	0.00038	0.0366	13.0000	0.3200	-5.2100	0.7300	-11.9600	23.4100	0.3200
S17JS10-10@09	2.4968	1.829E+09	0.3124	3682151	0.3122	698840.3	0.3060	0.00201	0.0118	0.00038	0.0207	4.1000	0.2400	-1.9600	0.4100	-4.0900	14.5000	0.2400
S17JS10-10@10	2.4761	1.796E+09	0.2547	3654775	0.2590	689809.7	0.2575	0.00203	0.0098	0.00038	0.0231	14.6700	0.2000	2.9700	0.4600	-4.6600	25.0800	0.2000
S17JS10-10@11	2.4572	1.791E+09	0.2746	3645968	0.2761	689738.8	0.2779	0.00204	0.0142	0.00039	0.0145	15.1800	0.2800	5.8000	0.2900	-2.1000	25.5900	0.2800
S17JS12-2D@01	2.4421	1.761E+09	0.2565	3580132	0.2626	674971.5	0.2605	0.00203	0.0146	0.00038	0.0218	13.9800	0.2900	1.1700	0.4400	-6.0900	24.3800	0.2900
S17JS12-2D@02	2.4327	1.87E+09	0.2996	3800216	0.2985	717407.5	0.2920	0.00203	0.0107	0.00038	0.0197	13.4400	0.2100	1.9600	0.3900	-5.0200	23.8500	0.2100
S17JS12-2D@05	2.4239	1.759E+09	0.2417	3575340	0.2452	674163.1	0.2445	0.00203	0.0129	0.00038	0.0245	13.5700	0.2600	0.9100	0.4900	-6.1400	23.9800	0.2600
S17JS12-2D@04	2.4241	1.754E+09	0.2832	3567302	0.2864	673088	0.2822	0.00203	0.0115	0.00038	0.0239	14.1200	0.2300	2.1200	0.4800	-5.2300	24.5300	0.2300
S17JS12-2D@03	2.4295	1.733E+09	0.2485	3524198	0.2506	665836.8	0.2440	0.00203	0.0120	0.00038	0.0204	13.9900	0.2400	3.3100	0.4100	-3.9600	24.4000	0.2400
S17JS8-7W@01	2.3874	1.785E+09	0.2677	3623089	0.2711	684625.3	0.2687	0.00203	0.0124	0.00038	0.0186	12.0400	0.2500	1.5400	0.3700	-4.7200	22.4500	0.2500
S17JS8-7W@02	2.3838	1.756E+09	0.2650	3567586	0.2638	674011.2	0.2603	0.00203	0.0126	0.00038	0.0324	13.4100	0.2500	2.7100	0.6500	-4.2600	23.8200	0.2500
S17JS8-7W@03	2.3867	1.749E+09	0.2524	3554406	0.2587	672312.2	0.2533	0.00203	0.0124	0.00038	0.0190	13.5900	0.2500	4.0600	0.3800	-3.0000	24.0000	0.2500
S17JS8-7W@04	2.3957	1.767E+09	0.2671	3585514	0.2673	676865.4	0.2687	0.00203	0.0106	0.00038	0.0191	11.7800	0.2100	0.3100	0.3800	-5.8200	22.1900	0.2100
S17JS8-7W@05	2.3966	1.737E+09	0.2611	3530004	0.2641	667677.2	0.2553	0.00203	0.0125	0.00038	0.0210	13.7500	0.2500	4.2000	0.4200	-2.9500	24.1600	0.2500
S17JS8-7W@06	2.3944	1.729E+09	0.2451	3518810	0.2393	665511	0.2494	0.00204	0.0106	0.00038	0.0266	15.0000	0.2100	5.3500	0.5300	-2.4500	25.4100	0.2100
S17JS8-7W@07	2.3999	1.747E+09	0.2557	3547328	0.2583	670255.3	0.2459	0.00203	0.0133	0.00038	0.0262	12.7400	0.2700	2.1600	0.5200	-4.4700	23.1500	0.2700
S17JS8-7W@08	2.3947	1.757E+09	0.2674	3563474	0.2703	673876.9	0.2651	0.00203	0.0113	0.00038	0.0285	11.5100	0.2300	1.7800	0.5700	-4.2000	21.9100	0.2300
S17JS8-7W@09	2.3879	1.745E+09	0.2844	3546249	0.2872	670483.8	0.2839	0.00203	0.0131	0.00038	0.0242	13.6600	0.2600	3.7100	0.4800	-3.4000	24.0700	0.2600
S17JS8-7W@10	2.3953	1.766E+09	0.2877	3585306	0.2903	676402.2	0.2885	0.00203	0.0163	0.00038	0.0345	12.7300	0.3300	0.6100	0.6900	-6.0000	23.1400	0.3300
S17JS8-7W@11	2.3908	1.7E+09	0.2535	3460862	0.2556	654452	0.2581	0.00204	0.0137	0.00038	0.0185	14.9900	0.2700	5.1900	0.3700	-2.6000	25.4000	0.2700
S17JS12-2D@06	2.3918	1.695E+09	0.2517	3451293	0.2490	652743.5	0.2467	0.00204	0.0137	0.00039	0.0147	15.6800	0.2700	6.0400	0.2900	-2.1200	26.0900	0.2700
S17JS924BRimbr8	2.3885	1.713E+09	0.2263	3478804	0.2271	657994	0.2249	0.00203	0.0138	0.00038	0.0296	12.8600	0.2800	3.3100	0.5900	-3.3700	23.2700	0.2800
S17JS924BRimbr9	2.3843	1.724E+09	0.2685	3496734	0.2678	661518.4	0.2642	0.00203	0.0159	0.00038	0.0251	11.7700	0.3200	2.4300	0.5000	-3.6800	22.1800	0.3200
S17JS924BRimbr10	2.3814	1.824E+09	0.2649	3696967	0.2665	699419.9	0.2561	0.00203	0.0092	0.00038	0.0269	10.7800	0.1800	1.4900	0.5400	-4.1100	21.1900	0.1800
S17JS10-10@12	2.3897	1.711E+09	0.2578	3477002	0.2574	656161.9	0.2559	0.00203	0.0095	0.00038	0.0338	13.4600	0.1900	1.6400	0.6800	-5.3600	23.8700	0.1900
S16HM10-8R@01	2.5840	1.833E+09	0.2240	3706146	0.2213	698175.4	0.2160	0.00202	0.0112	0.00038	0.0242	8.3600	0.2200	-5.1600	0.4800	-9.5100	18.7700	0.2200
S16HM13-7@01	2.5309	2.439E+09	0.1490	4938019	0.1465	938911.2	0.1487	0.00202	0.0094	0.00039	0.0180	9.8700	0.1900	5.6100	0.3600	0.4800	20.2800	0.1900
S16HM13-7@2	2.5155	1.811E+09	0.2635	3673485	0.2588	695474	0.2627	0.00203	0.0104	0.00038	0.0256	11.7700	0.2100	3.1800	0.5100	-2.9400	22.1800	0.2100
S16HM13-7@3	2.5224	1.781E+09	0.2671	3589757	0.2749	672639.1	0.2662	0.00202	0.0117	0.00038	0.0352	4.9100	0.2300	-13.8500	0.7000	-16.4000	15.3200	0.2300
S16HM13-7_2@01	2.7035	2.626E+09	0.3951	5333489	0.3916	1002027	0.4000	0.00203	0.0064	0.00038	0.0261	12.7100	0.1300	-3.5800	0.5200	-10.1800	23.1200	0.1300
S16HM13-7_2@02	2.6831	2.623E+09	0.3680	5342231	0.3626	1008228	0.3565	0.00204	0.0091	0.00038	0.0207	15.5500	0.1800	3.7700	0.4100	-4.3100	25.9600	0.1800
S16HM13-7_2@03	2.6702	2.663E+09	0.3572	5415317	0.3518	1018472	0.3518	0.00203	0.0101	0.00038	0.0221	14.0900	0.2000	-1.1500	0.4400	-8.4800	24.5000	0.2000
S16HM13-7_2@04	2.6722	2.615E+09	0.3279	5288073	0.3186	998788.5	0.3226	0.00202	0.0113	0.00038	0.0236	8.4700	0.2300	-2.4500	0.4700	-6.8600	18.8800	0.2300
S16HM13-7_2@05	2.6468	2.753E+09	0.3276	5576720	0.3281	1056827	0.3323	0.00203	0.0102	0.00038	0.0225	10.2000	0.2000	2.6000	0.4500	-2.7100	20.6100	0.2000
S16HM13-7_2@06	2.6473	1.878E+09	0.2241	3805929	0.2220	720761.9	0.2223	0.00203	0.0121	0.00038	0.0194	10.9100	0.2400	2.6200	0.3900	-3.0500	21.3100	0.2400
S16HM13-7_2@07	2.6449	1.874E+09	0.2126	3811063	0.2154	722585.8	0.2103	0.00203	0.0141	0.00039	0.0206	14.0700	0.2800	6.9500	0.4100	-0.3700	24.4800	0.2800
S16HM13-7R_1@01	2.6479	1.96E+09	0.2421	3965509	0.2408	749851.9	0.2434	0.00202	0.0110	0.00038	0.0238	9.2300	0.2200	-0.5500	0.4800	-5.3500	19.6400	0.2200
S16HM13-7R_1@02	2.6563	1.953E+09	0.2240	3935224	0.2210	738663.9	0.2158	0.00202	0.0143	0.00038	0.0402	5.0500	0.2900	-11.9800	0.8000	-14.6100	15.4600	0.2900
S16HM13-7R_1@03	2.6569	1.941E+09	0.2492	3921726	0.2470	740888.5	0.2417	0.00202	0.0120	0.00038	0.0253	7.8300	0.2400	-2.8600	0.5100	-6.9300	18.2300	0.2400
S16HM13-7R_1@04	2.6599	1.947E+09	0.2109	3926547	0.2042	739357.2	0.2020	0.00202	0.0156	0.00038	0.0363	5.6700	0.3100	-8.2600	0.7300	-11.2100	16.0800	0.3100
S16HM13-7R_1@05	2.6574	1.961E+09	0.2856	3951790	0.2843	741871.2	0.2750	0.00202	0.0116	0.00038	0.0294	5.1400	0.2300	-11.7600	0.5900	-14.4400	15.5500	0.2300
S16HM13-7R_2@01	2.6520	1.957E+09	0.2331	3958794	0.2372	749300.3	0.2263	0.00202	0.0135	0.00038	0.0221	8.9800	0.2700	0.1700	0.4400	-4.5000	19.3900	0.2700
S16HM13-7R_2@02	2.6505	2.037E+09	0.2976	4118814	0.2992	779336.9	0.2841	0.00202	0.0101	0.00038	0.0315	8.5100	0.2000	-0.6200	0.6300	-5.0400	18.9100	0.2000
S16HM13-7R_2@03	2.6481	1.935E+09	0.2325	3915961	0.2325	740836.6	0.2366	0.00202	0.0146	0.00038	0.0283	9.1800	0.2900	-0.1200	0.5700	-4.9000	19.5900	0.2900
S16HM13-7R_2@04	2.6552	2.062E+09	0.2974	4178270	0.2994	793954.2	0.2930	0.00203	0.0085	0.00039	0.0229	10.7700	0.1700	5.8900	0.4600	0.2800	21.1800	0.1700
S16HM13-7R_2@05	2.6583	2.249E+09	0.4309	4534013	0.4336	861354.8	0.4357	0.00202	0.0094	0.00038	0.0214	5.5200	0.1900	0.4200	0.4300	-2.4500	15.9200	0.1900
S16HM13-7R_3@01	2.6627	2.001E+09	0.2799	4039509	0.2797	764184.4	0.2655	0.00202	0.0092	0.00038	0.0222	6.7200	0.1800	-2.5800	0.4400	-6.0800	17.1300	0.1800
S16HM13-7R_3@02	2.6671	1.996E+09	0.2626	4031397	0.2697	763364.1	0.2585	0.00202	0.0143	0.00038	0.0210	7.1300	0.2900	-1.2500	0.4200	-4.9500	17.5400	0.2900
S16HM13-7R_3@03	2.6634	1.998E+09	0.2455	4033394	0.2485	763464.7	0.2447	0.00202	0.0147	0.00038	0.0258	6.8700	0.2900	-1.8700	0.5200	-5.4400	17.2800	0.2900
S16HM13-7R_3@04	2.6481	2.003E+09	0.2489	4041019	0.2514	766288.4	0.2441	0.00202	0.0120	0.00038	0.0206	6.2900	0.2400	-0.6400	0.4100	-3.9100	16.7000	0.2400
S16HM13-7R_3@05	2.5977	1.914E+09	0.3345	3863364	0.3407	731604.7	0.3412	0.00202	0.0121	0.00038	0.0296	6.5400	0.2400	-1.7500	0.5900	-5.1500	16.9500	0.2400
S16HM10-8R_2@01	2.5941	1.898E+09	0.1930	3835025	0.1945	725194.1	0.1990	0.00202	0.0119	0.00038	0.0216	7.7000	0.2400	-2.0400	0.4300	-6.0500	18.1100	0.2400
S16HM10-8R_2@02	2.6187	1.88E+09	0.2529	3797752	0.2517	714228.6	0.2550	0.00202	0.0110	0.00038	0.0252	7.3000	0.2200	-7.8800	0.5000	-11.6800	17.7100	0.2200
S16HM10-8R_2@03	2.6288	1.835E+09	0.2255	3705820	0.2302	696005.9	0.2345	0.00202	0.0154	0.00038	0.0426	7.1400	0.3100	-9.3700	0.8500	-13.0800	17.5500	0.3100
S16HM10-8R_1@01	2.6085	1.923E+09	0.2528	3892330	0.2500	735204.6	0.2489	0.00202	0.0152	0.00038	0.0195	9.4200	0.3000	-1.4600	0.3900	-6.3600	19.8300	0.3000
S16HM10-8R_1@02	2.5891	1.867E+09	0.2086	3776264	0.2107	710846.1	0.1908	0.00202	0.0098	0.00038	0.0421	8.6000	0.2000	-5.6700	0.8400	-10.1400	19.0100	0.2000
S16HM10-8R_3@01	2.5812	1.844E+09	0.2447	3735894	0.2447	706008.8	0.2307	0.00203	0.0115	0.00038	0.0384	10.2500	0.2300	-0.1300	0.7700	-5.4600	20.6600	0.2300
S16HM10-8R_3@02	2.5840	1.885E+09	0.2510	3813070	0.2479	720624.2	0.2330	0.00202	0.0111	0.00038	0.0264	8.7800	0.2200	-1.5400	0.5300	-6.1100	19.1900	0.2200
S16HM10-8R_3@03	2.5836	1.856E+09	0.2525	3765447	0.2474	715708.8	0.2502	0.00203	0.0117	0.00039	0.0252	11.9900	0.2300	7.3700	0.5000	1.1400	22.3900	0.2300
S16HM10-8R_4@01	2.5816	1.854E+09	0.1831	3751594	0.1879	709881.7	0.1886	0.00202	0.0154	0.00038	0.0250	9.0200	0.3100	-0.0800	0.5000	-4.7700	19.4300	0.3100
S16HM10-8R_4@02	2.5447	1.798E+09	0.2213	3651931	0.2241	693680.4	0.2188	0.00203	0.0116	0.00039	0.0257	12.8200	0.2300	7.5500	0.5100	0.8800	23.2300	0.2300
S16HM924qfvHMD_1@01	2.5705	1.892E+09	0.2386	3833372	0.2411	725874.2	0.2409	0.00203	0.0094	0.00038	0.0187	10.3900	0.1900	2.0000	0.3700	-3.4100	20.8000	0.1900
S16HM924qfvHMD_1@02	2.5784	1.89E+09	0.2203	3826009	0.2193	725229.2	0.2159	0.00202	0.0097	0.00038	0.0192	9.5700	0.1900	2.2200	0.3800	-2.7600	19.9800	0.1900
S16HM924qfvHMD_1@03	2.5723	1.892E+09	0.2299	3827937	0.2306	725530.3	0.2279	0.00202	0.0095	0.00038	0.0251	9.2400	0.1900	1.8000	0.5000	-3.0100	19.6500	0.1900
S16HM924qfvHMD_1@04	2.5801	1.901E+09	0.2285	3846059	0.2291	728217.5	0.2249	0.00202	0.0125	0.00038	0.0244	9.1700	0.2500	0.7000	0.4900	-4.0700	19.5800	0.2500
S16HM924qfvHMD_1@05	2.5763	1.874E+09	0.2258	3793470	0.2264	718676.1	0.2176	0.00202	0.0086	0.00038	0.0262	9.5400	0.1700	1.6600	0.5200	-3.3100	19.9500	0.1700
S16HM924qfvHMD_1@06	2.5823	1.881E+09	0.2314	3806803	0.2312	720731.7	0.2290	0.00202	0.0094	0.00038	0.0239	9.3500	0.1900	0.8100	0.4800	-4.0500	19.7600	0.1900
S16HM924qfvHMD_1@07	2.5759	1.892E+09	0.2148	3834071	0.2190	726890	0.2168	0.00203	0.0108	0.00038	0.0199	10.5500	0.2200	3.3800	0.4000	-2.1100	20.9600	0.2200
S16HM923qfvMT_1@01	2.5739	1.925E+09	0.2486	3877083	0.2530	736699.5	0.2471	0.00201	0.0110	0.00038	0.0235	4.4100	0.2200	-0.4800	0.4700	-2.7700	14.8200	0.2200
S16HM923qfvMT_1@02	2.5652	1.927E+09	0.2554	3869882	0.2600	735555.6	0.2549	0.00201	0.0106	0.00038	0.0185	1.5300	0.2100	-3.0400	0.3700	-3.8400	11.9400	0.2100
S16HM923qfvMT_1@03	2.5571	1.876E+09	0.2246	3774772	0.2228	718575	0.2152	0.00201	0.0130	0.00038	0.0210	3.2600	0.2600	0.2100	0.4200	-1.4900	13.6700	0.2600
S16HM923qfvMT_1@04	2.5603	1.9E+09	0.2254	3828571	0.2269	726647.9	0.2218	0.00202	0.0075	0.00038	0.0272	4.9200	0.1500	-1.1200	0.5400	-3.6800	15.3200	0.1500
S16HM923qfvMT_1@05	2.5501	1.933E+09	0.2051	3906253	0.2053	739793.4	0.1990	0.00202	0.0108	0.00038	0.0245	7.6900	0.2200	-0.5200	0.4900	-4.5200	18.1000	0.2200
S16HM923qfvMT_1@06	2.5359	1.913E+09	0.2555	3865634	0.2579	734428.4	0.2567	0.00202	0.0140	0.00038	0.0164	7.7800	0.2800	2.7400	0.3300	-1.3000	18.1800	0.2800
S16HM923qfvMT_1@07	2.5321	1.89E+09	0.2257	3814815	0.2264	724275.9	0.2253	0.00202	0.0121	0.00038	0.0184	6.5700	0.2400	0.8600	0.3700	-2.5600	16.9800	0.2400
S16HM10-2matyel_1@01	2.5277	1.848E+09	0.2722	3741411	0.2746	708477.4	0.2645	0.00202	0.0140	0.00038	0.0294	9.5600	0.2800	1.2000	0.5900	-3.7700	19.9700	0.2800
S16HM10-2matyel_1@02	2.5171	1.805E+09	0.1975	3656687	0.2039	691543.8	0.2015	0.00203	0.0135	0.00038	0.0225	10.0300	0.2700	0.3700	0.4500	-4.8400	20.4400	0.2700
S16HM10-2matyel_1@03	2.5057	1.791E+09	0.2112	3629040	0.2065	686030.5	0.2149	0.00203	0.0132	0.00038	0.0212	10.7100	0.2600	0.6300	0.4200	-4.9400	21.1200	0.2600
S16HM10-2matyel_1@04	2.4819	1.786E+09	0.2301	3621964	0.2280	686551.6	0.2275	0.00203	0.0098	0.00038	0.0216	11.2500	0.2000	3.8800	0.4300	-1.9700	21.6600	0.2000
S16HM10-2matyel_1@05	2.4885	1.74E+09	0.1871	3538916	0.1905	671006.9	0.1853	0.00203	0.0158	0.00039	0.0240	14.5600	0.3200	7.4700	0.4800	-0.1000	24.9700	0.3200

Sample Name	Ipr (nA)	16O/Coeff	1se(%)	18O/Coeff	1se(%)	17O/Coeff	1se(%)	18O/16O	1se(%)	17O/16O	1se(%)	δ18O		δ17O		big delta	corrected value	
S16HM10-2matyel_2@01	2.4697	1.778E+09	0.2160	3605346	0.2202	682898.2	0.2163	0.00203	0.0098	0.00038	0.0261	11.2400	0.2000	3.1400	0.5200	-2.7100	21.6500	0.2000
S16HM10-2matyel_2@02	2.4667	1.804E+09	0.2206	3656694	0.2203	691591.2	0.2160	0.00203	0.0148	0.00038	0.0278	10.9100	0.3000	1.3100	0.5600	-4.3600	21.3200	0.3000
S16HM10-2matyel_2@03	2.4721	1.767E+09	0.2240	3583302	0.2198	678204.3	0.2242	0.00203	0.0090	0.00038	0.0259	11.1400	0.1800	2.2600	0.5200	-3.5300	21.5500	0.1800
S16HM10-2matyel_2@04	2.4634	1.763E+09	0.2243	3573749	0.2223	674310.8	0.2156	0.00203	0.0131	0.00038	0.0240	10.7300	0.2600	-1.2200	0.4800	-6.8000	21.1400	0.2600
S16HM10-2matyel_2@05	2.4792	1.758E+09	0.2204	3564913	0.2227	674447.1	0.2240	0.00203	0.0117	0.00038	0.0182	11.2800	0.2300	2.0000	0.3600	-3.8700	21.6900	0.2300
S16HM10-2matyel_3@01	2.4792	1.822E+09	0.1940	3693867	0.1941	698614.6	0.1805	0.00203	0.0085	0.00038	0.0353	11.1900	0.1700	1.5800	0.7100	-4.2400	21.6000	0.1700
S16HM10-2matyel_3@02	2.4796	1.771E+09	0.2112	3589835	0.2062	679867.4	0.2068	0.00203	0.0130	0.00038	0.0255	10.8600	0.2600	2.6200	0.5100	-3.0300	21.2700	0.2600
S16HM10-2matyel_3@03	2.4572	1.823E+09	0.2608	3693104	0.2599	698365.2	0.2628	0.00203	0.0115	0.00038	0.0243	10.5000	0.2300	0.7400	0.4900	-4.7200	20.9100	0.2300
S16HM10-2matyel_3@04	2.4473	1.789E+09	0.2168	3628229	0.2123	687006.9	0.2108	0.00203	0.0131	0.00038	0.0257	11.4900	0.2600	3.0500	0.5100	-2.9200	21.9000	0.2600
S16HM10-2matyel_3@05	2.4432	1.826E+09	0.2712	3700384	0.2724	700058.8	0.2668	0.00203	0.0103	0.00038	0.0220	10.5800	0.2100	1.2800	0.4400	-4.2200	20.9900	0.2100
S16HM10-2matyel_4@01	2.4708	1.772E+09	0.1768	3587921	0.1798	673366.6	0.1805	0.00203	0.0129	0.00038	0.0209	9.9300	0.2600	-7.3500	0.4200	-12.5200	20.3400	0.2600
S16HM10-2matyel_4@02	2.4549	1.742E+09	0.2185	3531199	0.2174	663877	0.2057	0.00203	0.0105	0.00038	0.0307	10.8300	0.2100	-4.7300	0.6100	-10.3600	21.2400	0.2100
S16HM10-2matyel_4@03	2.4579	1.757E+09	0.2557	3559966	0.2586	669059.7	0.2546	0.00203	0.0157	0.00038	0.0258	10.4000	0.3100	-5.4900	0.5200	-10.9000	20.8100	0.3100
S16HM10-2matyel_4@04	2.4627	1.757E+09	0.2334	3549955	0.2353	663581.6	0.2449	0.00202	0.0124	0.00038	0.0404	7.8300	0.2500	-13.3700	0.8100	-17.4500	18.2400	0.2500
S16HM10-2matyel_4@05	2.4618	1.715E+09	0.2326	3474401	0.2357	652740.7	0.2358	0.00203	0.0097	0.00038	0.0310	10.0200	0.1900	-6.2300	0.6200	-11.4400	20.4300	0.1900
S16HM924BRum_1@01	2.4523	1.77E+09	0.2364	3557231	0.2362	649026.6	0.2094	0.00201	0.0177	0.00037	0.0702	2.1500	0.3500	-42.4000	1.4000	-43.5200	12.5500	0.3500
S16HM924BRum_1@02	2.4505	1.749E+09	0.2131	3519830	0.2167	644925.2	0.2274	0.00201	0.0146	0.00037	0.0679	3.5700	0.2900	-36.9900	1.3600	-38.8500	13.9800	0.2900
S16HM924BRum_1@03	2.4430	1.721E+09	0.2224	3463768	0.2185	637670.5	0.2195	0.00201	0.0148	0.00037	0.0713	3.9600	0.3000	-32.0400	1.4300	-34.0900	14.3600	0.3000
S16HM924BRum_1@04	2.4421	1.727E+09	0.2314	3481306	0.2286	643411.4	0.2106	0.00202	0.0138	0.00037	0.0397	5.4400	0.2800	-26.8000	0.7900	-29.6300	15.8500	0.2800
S16HM924BRum_1@05	2.4551	1.706E+09	0.2310	3447669	0.2300	640900.2	0.2049	0.00202	0.0121	0.00038	0.0481	7.8400	0.2400	-18.8100	0.9600	-22.8800	18.2400	0.2400
S16HM924BRum_1@06	2.4579	1.759E+09	0.2339	3552493	0.2339	657561.3	0.2273	0.00202	0.0090	0.00037	0.0464	7.0700	0.1800	-23.7500	0.9300	-27.4300	17.4800	0.1800
S16HM924BRum_1@07	2.4603	1.692E+09	0.2121	3424638	0.2199	638253.3	0.2183	0.00202	0.0143	0.00038	0.0360	9.3000	0.2900	-14.8700	0.7200	-19.7100	19.7100	0.2900
S16HM924BRum_2@01	2.4287	1.746E+09	0.2101	3541574	0.2061	670438.3	0.1934	0.00203	0.0147	0.00038	0.0315	11.8500	0.2900	3.1700	0.6300	-2.9900	22.2600	0.2900
S16HM924BRum_2@02	2.4370	1.748E+09	0.2107	3547648	0.2104	671177	0.2142	0.00203	0.0112	0.00038	0.0238	12.0000	0.2200	2.7000	0.4800	-3.5400	22.4100	0.2200
S16HM924BRum_2@03	2.4371	1.749E+09	0.2301	3543159	0.2377	669398.7	0.2375	0.00203	0.0121	0.00038	0.0226	10.5400	0.2400	-0.1300	0.4500	-5.6100	20.9500	0.2400
S16HM924BRum_2@04	2.4419	1.73E+09	0.2134	3505822	0.2171	663387.4	0.2120	0.00203	0.0126	0.00038	0.0276	10.3500	0.2500	1.2600	0.5500	-4.1200	20.7600	0.2500
S16HM924BRum_2@05	2.4414	1.743E+09	0.2102	3531516	0.2118	666671.7	0.2140	0.00203	0.0126	0.00038	0.0211	10.2900	0.2500	-1.1700	0.4200	-6.5200	20.7000	0.2500
S16HM924BRum_2@06	2.4434	1.712E+09	0.2436	3472628	0.2413	656150.1	0.2416	0.00203	0.0118	0.00038	0.0240	11.3800	0.2400	0.8100	0.4800	-5.1000	21.7800	0.2400
S16HM924BRum_2@07	2.4377	1.691E+09	0.1857	3419668	0.1898	643987.1	0.1923	0.00202	0.0136	0.00038	0.0301	8.5000	0.2700	-5.3600	0.6000	-9.7800	18.9100	0.2700
S16HM924BRum_3@01	2.4213	1.861E+09	0.2584	3771842	0.2585	713579.4	0.2592	0.00203	0.0090	0.00038	0.0174	10.6400	0.1800	1.3300	0.3500	-4.2000	21.0400	0.1800
S16HM924BRum_3@02	2.4087	1.836E+09	0.2542	3718340	0.2504	703012.9	0.2427	0.00203	0.0119	0.00038	0.0335	10.0400	0.2400	0.1100	0.6700	-5.1100	20.4500	0.2400
S16HM924BRum_3@03	2.4039	1.828E+09	0.2254	3710026	0.2252	704071.6	0.2197	0.00203	0.0097	0.00039	0.0221	12.2100	0.1900	6.0200	0.4400	-0.3300	22.6200	0.1900
S16HM924BRum_3@04	2.4164	1.841E+09	0.2576	3734590	0.2580	709059.8	0.2630	0.00203	0.0128	0.00039	0.0233	11.8900	0.2600	6.1600	0.4700	-0.0200	22.3000	0.2600
S16HM924BRum_3@05	2.4126	1.796E+09	0.2100	3651571	0.2094	693678.4	0.2098	0.00203	0.0126	0.00039	0.0256	13.7800	0.2500	8.5900	0.5100	1.4300	24.1900	0.2500
S16HM924BRum_3@06	2.4177	1.741E+09	0.1920	3542083	0.1904	673921.3	0.1890	0.00203	0.0122	0.00039	0.0297	14.7500	0.2400	11.1300	0.5900	3.4600	25.1600	0.2400
S16HM924BRum_3@07	2.4152	1.721E+09	0.1994	3502156	0.1960	665214.7	0.2012	0.00204	0.0131	0.00039	0.0248	14.9900	0.2600	9.6800	0.5000	1.8800	25.4000	0.2600
S16HM924BRum_4@01	2.4330	1.782E+09	0.2370	3615003	0.2350	684778.4	0.2354	0.00203	0.0131	0.00038	0.0169	11.7200	0.2600	3.6800	0.3400	-2.4100	22.1300	0.2600
S16HM924BRum_4@02	2.4324	1.751E+09	0.2155	3554657	0.2161	673372.5	0.2115	0.00203	0.0106	0.00038	0.0253	12.2400	0.2100	4.2400	0.5100	-2.1200	22.6500	0.2100
S16HM924BRum_4@03	2.4341	1.759E+09	0.2130	3567209	0.2124	674733.7	0.2038	0.00203	0.0113	0.00038	0.0239	11.3700	0.2300	1.8700	0.4800	-4.0400	21.7800	0.2300
S16HM924BRum_4@04	2.4266	1.738E+09	0.1860	3527524	0.1862	667736.5	0.1819	0.00203	0.0108	0.00038	0.0240	11.9800	0.2200	3.2300	0.4800	-2.9900	22.3900	0.2200
S16HM924BRum_4@05	2.4121	1.725E+09	0.1771	3502523	0.1760	662377.3	0.1726	0.00203	0.0145	0.00038	0.0221	12.5800	0.2900	2.8900	0.4400	-3.6600	22.9900	0.2900

Table S4-1. Analytical conditions for LA-MC-ICP-MS measurement of Strontium isotopes in carbonate minerals(calcite and dolomite).

MC-ICP-MS	
Instrument	Nu Plasma II, Nu instrumnets
RF power	1300 W
Reflected power	< 1 W
Mixed gas and flow rate	Ar, ~ 0.9 L/min
Auxiliary gas and flow rate	Ar, 0.8 L/min
Cool gas and flow rate	Ar, 13 L/min
Sampler & Skimmer cone	Ni (1 mm orifice)
Skimmer cone	Ni (0.7 mm orifice)
Data acquisition mode	Time resolved analysis
Integration times	0.4 secs
Collectors	2 Faraday cups and 2 ion counters
Measured isotopes	^{88}Sr, ^{87}Sr, ^{86}Sr, ^{84}Sr
Laser ablation system	
Laser	$NWA193^{UC}$, ESI machines (193 nm ArF excimer laser)
Ablation mode	Single hole drilling
Spot size	100~120 μm
Fluence	3.8~4.0 J/cm^2
Dwell time	30 secs
Cell	Two volume 2 cell (10 × 10 cm)

Table S4-2. LA-MC-ICP-MS Strontium isotope analyses data of carbonate minerals(calcite and dolomite).

Sample	Sr Beam	Sr87_86	2SE	Sr86_88	2SE	Sr84_86	2SE	Rb/Sr	1/Rb	1/Sr	(1/Sr)*1000	Sr_ppm	Rb_ppm
S11HD98C_1	2.61	0.7090	0.0003	0.1194	0.0000	0.0644	0.0003	0.0001	22.2222	0.0027	2.7270	366.70	0.05
S11HD98C_2	2.62	0.7091	0.0003	0.1194	0.0000	0.0640	0.0003	0.0014	1.8692	0.0027	2.6998	370.40	0.54
S11HD98C_3	2.60	0.7090	0.0002	0.1194	0.0000	0.0643	0.0004	0.0003	9.9010	0.0027	2.7167	368.10	0.10
S11HD98C_4	2.50	0.7091	0.0003	0.1194	0.0000	0.0647	0.0004	0.0003	8.7719	0.0028	2.8058	356.40	0.11
S11HD98C_5	2.53	0.7089	0.0003	0.1194	0.0000	0.0646	0.0003	0.0002	11.5607	0.0028	2.7647	361.70	0.09
HD-B-C1	3.23	0.7099	0.0003	0.1193	0.0000	0.0572	0.0003	0.0117	0.0866	0.0010	1.0127	987.50	11.55
HD-B-C2	3.29	0.7099	0.0002	0.1193	0.0000	0.0572	0.0003	0.0107	0.0931	0.0010	0.9996	1000.43	10.74
HD-B-C3	2.46	0.7093	0.0003	0.1193	0.0000	0.0586	0.0003	0.0007	1.8699	0.0013	1.3333	750.03	0.53
HD-B-C4	3.01	0.7106	0.0003	0.1193	0.0000	0.0572	0.0003	0.0167	0.0653	0.0011	1.0927	915.20	15.32
HD-B-C5	2.88	0.7092	0.0002	0.1193	0.0000	0.0579	0.0003	0.0010	1.1628	0.0011	1.1418	875.82	0.86
HD-A-C1	3.48	0.7091	0.0002	0.1194	0.0000	0.0574	0.0003	0.0050	0.1891	0.0009	0.9430	1060.39	5.29
HD-A-C2	3.94	0.7089	0.0002	0.1194	0.0000	0.0573	0.0002	0.0024	0.3509	0.0008	0.8348	1197.94	2.85
HD-A-C3	3.67	0.7089	0.0002	0.1194	0.0000	0.0575	0.0003	0.0021	0.4250	0.0009	0.8949	1117.41	2.35
HD-A-C4	3.75	0.7091	0.0002	0.1194	0.0000	0.0573	0.0002	0.0054	0.1612	0.0009	0.8769	1140.33	6.20
HD-A-C5	3.81	0.7094	0.0002	0.1194	0.0000	0.0572	0.0002	0.0071	0.1217	0.0009	0.8614	1160.90	8.21
S5HD9-6-C1	2.84	0.7094	0.0003	0.1193	0.0000	0.0601	0.0003	0.0001	24.2131	0.0016	1.6145	619.40	0.04
S5HD9-6-C2	2.76	0.7096	0.0003	0.1193	0.0000	0.0603	0.0003	0.0002	6.9930	0.0017	1.6628	601.40	0.14
S5HD9-6-C3	2.89	0.7096	0.0003	0.1193	0.0000	0.0601	0.0004	0.0002	8.1967	0.0016	1.5883	629.60	0.12
S5HD9-6-C4	3.94	0.7094	0.0003	0.1193	0.0000	0.0591	0.0002	0.0002	5.7143	0.0012	1.1641	859.00	0.18
S5HD9-6-C5	4.03	0.7093	0.0003	0.1193	0.0000	0.0588	0.0003	0.0016	0.7143	0.0011	1.1364	880.00	1.40
S2HD62C_1	3.35	0.7094	0.0003	0.1194	0.0000	0.0595	0.0003	0.0002	9.1408	0.0015	1.5188	658.40	0.11
S2HD62C_2	3.64	0.7094	0.0002	0.1194	0.0000	0.0591	0.0003	0.0001	12.4844	0.0014	1.3959	716.40	0.08
S2HD62C_3	3.40	0.7091	0.0002	0.1194	0.0000	0.0596	0.0003	0.0001	23.7530	0.0015	1.4952	668.80	0.04
S2HD62C_4	7.88	0.7090	0.0002	0.1194	0.0000	0.0578	0.0001	0.0004	1.6313	0.0006	0.6439	1553.00	0.61
S2HD62C_5	3.31	0.7095	0.0003	0.1194	0.0000	0.0596	0.0003	0.0031	0.4975	0.0015	1.5321	652.70	2.01
S5HM9-5-C1	3.00	0.7096	0.0003	0.1193	0.0000	0.0590	0.0004	0.0015	1.0101	0.0015	1.4837	674.00	0.99
S5HM9-5-C2	2.80	0.7093	0.0003	0.1193	0.0000	0.0599	0.0004	0.0001	12.1359	0.0016	1.5848	631.00	0.08
S5HM9-5-C3	2.98	0.7092	0.0003	0.1193	0.0000	0.0593	0.0004	0.0004	3.4965	0.0015	1.4881	672.00	0.29
S5HM9-5-C4	1.21	0.7099	0.0005	0.1192	0.0000	0.0648	0.0008	0.0001	47.6190	0.0037	3.6765	272.00	0.02
S5HM9-5-C5	2.86	0.7093	0.0003	0.1193	0.0000	0.0594	0.0003	0.0000	93.4579	0.0016	1.5504	645.00	0.01
S5HM9-3-C1	1.44	0.7099	0.0004	0.1192	0.0000	0.0630	0.0008	0.0000	142.8571	0.0031	3.1250	320.00	0.01
S5HM9-3-C2	2.54	0.7095	0.0003	0.1193	0.0000	0.0594	0.0004	0.0000	35.5872	0.0018	1.7637	567.00	0.03
S5HM9-3-C3	1.86	0.7100	0.0004	0.1192	0.0000	0.0608	0.0005	0.0051	0.4695	0.0024	2.4108	414.80	2.13
S5HM9-3-C4	2.20	0.7102	0.0004	0.1192	0.0000	0.0597	0.0004	0.0036	0.5602	0.0020	2.0400	490.20	1.79
S5HM9-3-C5	1.71	0.7103	0.0004	0.1192	0.0000	0.0612	0.0006	0.0020	1.2837	0.0026	2.6199	381.70	0.78
S5HM9-3-C1	24.71	0.7085	0.0002	0.1194	0.0000	0.0565	0.0001	0.0009	0.2137	0.0002	0.1833	5455.00	4.68
S5HM9-3-C2	24.54	0.7085	0.0002	0.1194	0.0000	0.0565	0.0001	0.0018	0.1035	0.0002	0.1845	5421.00	9.66
S5HM9-3-C3	23.61	0.7085	0.0002	0.1194	0.0000	0.0565	0.0001	0.0016	0.1181	0.0002	0.1916	5219.00	8.47
S11HM81C_1	3.28	0.7092	0.0002	0.1194	0.0000	0.0609	0.0003	0.0017	1.0526	0.0018	1.8308	546.20	0.95
S11HM81C_2	1.66	0.7096	0.0004	0.1194	0.0000	0.0667	0.0006	0.0000	133.3333	0.0036	3.6036	277.50	0.01
S11HM81C_3	3.05	0.7092	0.0003	0.1194	0.0000	0.0611	0.0003	0.0000	79.3651	0.0020	1.9550	511.50	0.01
S11HM81C_4	3.47	0.7093	0.0002	0.1194	0.0000	0.0607	0.0003	0.0000	102.0408	0.0017	1.7085	585.30	0.01
S11HM81C_5	1.18	0.7099	0.0005	0.1194	0.0000	0.0700	0.0007	0.0000	129.8701	0.0050	5.0100	199.60	0.01
HM1-A-C1	1.06	0.7108	0.0005	0.1192	0.0000	0.0591	0.0009	0.0107	0.2907	0.0031	3.1102	321.53	3.44
HM1-A-C3	0.73	0.7100	0.0008	0.1192	0.0001	0.0628	0.0013	0.0075	0.6087	0.0045	4.5367	220.42	1.64
HM1-A-C4	1.05	0.7101	0.0005	0.1192	0.0000	0.0599	0.0010	0.0092	0.3432	0.0031	3.1418	318.29	2.91
HM-C-C1	2.62	0.7094	0.0002	0.1194	0.0000	0.0500	0.0004	0.0000	74.1025	0.0014	1.3734	728.14	0.01
HM-C-C2	3.46	0.7096	0.0002	0.1194	0.0000	0.0497	0.0003	0.0000	64.3522	0.0010	1.0380	963.44	0.02
HM-C-C3	3.51	0.7095	0.0002	0.1194	0.0000	0.0498	0.0003	0.0000	74.1025	0.0010	1.0238	976.74	0.01
HM-C-C4	2.04	0.7097	0.0003	0.1194	0.0000	0.0524	0.0005	0.0000	163.0256	0.0018	1.7584	568.70	0.01
HM-C-C5	3.18	0.7090	0.0002	0.1194	0.0000	0.0499	0.0003	0.0001	21.6406	0.0011	1.1324	883.11	0.05
HM1-B-C2	0.84	0.7103	0.0007	0.1192	0.0000	0.0613	0.0011	0.0016	2.4552	0.0039	3.9381	253.93	0.41
S10HM1A-C1	1.35	0.7097	0.0005	0.1192	0.0000	0.0657	0.0007	0.0003	13.8889	0.0035	3.5137	284.60	0.07
S10HM1A-C2	1.66	0.7098	0.0004	0.1192	0.0000	0.0622	0.0007	0.0013	2.2422	0.0028	2.8490	351.00	0.45
S10HM1A-C3	1.79	0.7098	0.0004	0.1192	0.0000	0.0622	0.0005	0.0013	2.0492	0.0026	2.6399	378.80	0.49
S10HM1A-C4	1.89	0.7099	0.0003	0.1192	0.0000	0.0613	0.0006	0.0026	0.9690	0.0025	2.5000	400.00	1.03
S10HM1A-C5	1.60	0.7098	0.0005	0.1192	0.0000	0.0620	0.0006	0.0027	1.0977	0.0030	2.9586	338.00	0.91
S2HM73C_1	3.78	0.7093	0.0002	0.1194	0.0000	0.0586	0.0003	0.0000	113.6364	0.0014	1.3548	738.10	0.01
S2HM73C_2	3.94	0.7093	0.0002	0.1194	0.0000	0.0585	0.0002	0.0001	15.8479	0.0013	1.3006	768.90	0.06
S2HM73C_3	4.13	0.7091	0.0002	0.1194	0.0000	0.0585	0.0002	0.0000	78.1250	0.0012	1.2405	806.10	0.01
S2HM73C_4	5.80	0.7094	0.0002	0.1194	0.0000	0.0579	0.0002	0.0002	4.0650	0.0009	0.8838	1131.50	0.25
S2HM73C_5	4.95	0.7092	0.0002	0.1194	0.0000	0.0579	0.0002	0.0001	8.3264	0.0010	1.0359	965.30	0.12
S2HM72C_1	3.54	0.7091	0.0002	0.1194	0.0000	0.0586	0.0003	0.0001	13.4771	0.0014	1.4472	691.00	0.07
S2HM72C_2	4.87	0.7090	0.0002	0.1194	0.0000	0.0579	0.0002	0.0002	6.1387	0.0011	1.0504	952.00	0.16
S2HM72C_3	4.94	0.7090	0.0002	0.1194	0.0000	0.0580	0.0002	0.0003	3.2648	0.0010	1.0341	967.00	0.31
S2HM72C_4	4.52	0.7089	0.0002	0.1194	0.0000	0.0582	0.0002	0.0004	2.9011	0.0011	1.1289	885.80	0.34
S2HM72C_5	4.90	0.7088	0.0002	0.1194	0.0000	0.0581	0.0002	0.0004	2.7174	0.0010	1.0411	960.50	0.37
S11HM85C_1	1.90	0.7096	0.0004	0.1194	0.0000	0.0628	0.0006	0.0058	0.5030	0.0029	2.9240	342.00	1.99
S11HM85C_2	3.34	0.7094	0.0002	0.1194	0.0000	0.0600	0.0003	0.0020	0.8065	0.0017	1.6526	605.10	1.24
S11HM85C_3	2.00	0.7097	0.0003	0.1194	0.0000	0.0616	0.0005	0.0008	3.3898	0.0028	2.7533	363.20	0.30
S11HM85C_4	2.12	0.7098	0.0003	0.1194	0.0000	0.0630	0.0004	0.0009	2.9499	0.0026	2.5833	387.10	0.34
S11HM85C_5	2.11	0.7095	0.0003	0.1194	0.0000	0.0627	0.0005	0.0040	0.6494	0.0026	2.5813	387.40	1.54
HM3-A-C1	5.88	0.7086	0.0001	0.1194	0.0000	0.0563	0.0002	0.0000	41.7380	0.0006	0.5583	1791.02	0.02
HM3-A-C2	6.57	0.7086	0.0001	0.1194	0.0000	0.0564	0.0002	0.0000	24.3472	0.0005	0.4988	2004.98	0.04
HM3-A-C3	2.86	0.7088	0.0003	0.1194	0.0000	0.0571	0.0003	0.0001	9.2384	0.0011	1.1495	869.94	0.11
HM3-A-C4	3.90	0.7088	0.0002	0.1194	0.0000	0.0568	0.0002	0.0001	7.9772	0.0008	0.8422	1187.35	0.13
HM3-A-C5	4.13	0.7088	0.0002	0.1194	0.0000	0.0566	0.0002	0.0000	45.8299	0.0008	0.7950	1257.89	0.02
HM3-B-C1	4.93	0.7091	0.0002	0.1193	0.0000	0.0563	0.0002	0.0004	1.5687	0.0007	0.6646	1504.77	0.64
HM3-B-C2	2.78	0.7092	0.0002	0.1193	0.0000	0.0572	0.0003	0.0002	6.6213	0.0012	1.1814	846.43	0.15
HM3-B-C3	5.94	0.7089	0.0001	0.1194	0.0000	0.0561	0.0002	0.0003	1.9641	0.0006	0.5524	1810.42	0.51
S10HM3A-C1	6.05	0.7090	0.0002	0.1193	0.0000	0.0578	0.0002	0.0001	8.3333	0.0008	0.7813	1280.00	0.12
S10HM3A-C3	6.18	0.7090	0.0002	0.1193	0.0000	0.0579	0.0002	0.0019	0.4132	0.0008	0.7651	1307.00	2.42
S10HM3A-C4	3.74	0.7093	0.0003	0.1193	0.0000	0.0593	0.0004	0.0011	1.1905	0.0013	1.2658	790.00	0.84
S10HM3A-C5	2.23	0.7101	0.0004	0.1192	0.0000	0.0607	0.0004	0.0083	0.2545	0.0021	2.1169	472.40	3.93
S10HM3B-FC1	7.66	0.7086	0.0002	0.1194	0.0000	0.0581	0.0001	0.0000	32.2581	0.0006	0.6158	1624.00	0.03
S10HM3B-FC2	2.54	0.7095	0.0003	0.1193	0.0000	0.0606	0.0004	0.0000	125.0000	0.0019	1.8580	538.20	0.01
S10HM3B-FC3	4.26	0.7090	0.0002	0.1193	0.0000	0.0587	0.0002	0.0000	36.7647	0.0011	1.1086	902.00	0.03
S10HM3B-FC4	9.42	0.7087	0.0002	0.1194	0.0000	0.0577	0.0001	0.0000	26.5252	0.0005	0.5013	1995.00	0.04
S10HM3B-FC5	10.47	0.7086	0.0002	0.1194	0.0000	0.0575	0.0001	0.0000	14.0252	0.0005	0.4509	2218.00	0.07
S9HM102C_2	1.80	0.7104	0.0003	0.1194	0.0000	0.0787	0.0005	0.0000	96.1538	0.0047	4.6664	214.30	0.01
S9HM102C_3	3.22	0.7101	0.0002	0.1194	0.0000	0.0698	0.0005	0.0000	227.2727	0.0026	2.6042	384.00	0.00

Sample	Sr Beam	Sr87_86	2SE	Sr86_88	2SE	Sr84_86	2SE	Rb/Sr	1/Rb	1/Sr	(1/Sr)*1000	Sr_ppm	Rb_ppm
S9HM102C_4	2.57	0.7101	0.0002	0.1194	0.0000	0.0718	0.0005	0.0000	175.4386	0.0033	3.2573	307.00	0.01
S9HM102C_5	8.23	0.7094	0.0001	0.1194	0.0000	0.0609	0.0001	0.0000	138.8889	0.0010	1.0173	983.00	0.01
S5HM9-4-C1	3.47	0.7088	0.0002	0.1193	0.0000	0.0588	0.0003	0.0008	1.5385	0.0013	1.3038	767.00	0.65
S5HM9-4-C2	1.15	0.7098	0.0005	0.1192	0.0000	0.0655	0.0010	0.0000	92.5926	0.0040	3.9526	253.00	0.01
S2JS710C_1	3.69	0.7090	0.0002	0.1194	0.0000	0.0585	0.0002	0.0001	12.4844	0.0014	1.3835	722.80	0.08
S2JS710C_2	3.54	0.7092	0.0002	0.1194	0.0000	0.0587	0.0003	0.0001	11.7371	0.0014	1.4409	694.00	0.09
S2JS710C_3	3.88	0.7091	0.0002	0.1194	0.0000	0.0587	0.0002	0.0001	12.4069	0.0013	1.3177	758.90	0.08
S2JS710C_4	3.80	0.7092	0.0002	0.1194	0.0000	0.0587	0.0003	0.0001	13.4590	0.0013	1.3455	743.20	0.07
S2JS710C_5	4.01	0.7090	0.0003	0.1194	0.0000	0.0586	0.0003	0.0002	8.1235	0.0013	1.2755	784.00	0.12
S9JS1010C_1	23.45	0.7088	0.0001	0.1194	0.0000	0.0582	0.0000	0.0001	3.1546	0.0004	0.3597	2780.00	0.32
S9JS1010C_2	22.90	0.7088	0.0001	0.1194	0.0000	0.0582	0.0000	0.0001	2.9412	0.0004	0.3679	2718.00	0.34
S9JS1010C_3	21.05	0.7088	0.0001	0.1194	0.0000	0.0583	0.0000	0.0001	4.7619	0.0004	0.4000	2500.00	0.21
S9JS1010C_4	22.51	0.7088	0.0001	0.1194	0.0000	0.0582	0.0000	0.0003	1.2642	0.0004	0.3737	2676.00	0.79
S9JS1010C_5	24.68	0.7088	0.0001	0.1194	0.0000	0.0582	0.0000	0.0001	5.2632	0.0003	0.3406	2936.00	0.19
S11JS87WC_1	3.84	0.7090	0.0003	0.1194	0.0000	0.0605	0.0002	0.0010	1.7212	0.0018	1.8018	555.00	0.58
S11JS87WC_2	3.39	0.7093	0.0003	0.1194	0.0000	0.0614	0.0003	0.0002	10.0000	0.0020	2.0325	492.00	0.10
S11JS87WC_3	5.09	0.7090	0.0002	0.1194	0.0000	0.0599	0.0002	0.0001	14.3472	0.0013	1.3475	742.10	0.07
S11JS87WC_4	3.90	0.7090	0.0002	0.1194	0.0000	0.0613	0.0002	0.0001	21.9780	0.0018	1.7538	570.20	0.05
S11JS87WC_5	1.83	0.7096	0.0003	0.1194	0.0000	0.0672	0.0005	0.0000	91.7431	0.0037	3.7313	268.00	0.01
S11JS89C_1	3.82	0.7099	0.0003	0.1194	0.0000	0.0598	0.0002	0.0050	0.3377	0.0017	1.6935	590.50	2.96
S11JS89C_2	4.12	0.7099	0.0002	0.1194	0.0000	0.0599	0.0002	0.0032	0.4926	0.0016	1.5605	640.80	2.03
S11JS89C_3	4.21	0.7099	0.0002	0.1194	0.0000	0.0597	0.0002	0.0050	0.3057	0.0015	1.5218	657.10	3.27
S11JS89C_4	4.31	0.7101	0.0003	0.1194	0.0000	0.0599	0.0002	0.0057	0.2601	0.0015	1.4819	674.80	3.84
S11JS89C_5	4.53	0.7099	0.0002	0.1194	0.0000	0.0599	0.0002	0.0034	0.4082	0.0014	1.4029	712.80	2.45
S9JS122C_1	15.10	0.7087	0.0001	0.1194	0.0000	0.0594	0.0001	0.0002	2.4038	0.0006	0.5624	1778.00	0.42
S9JS122C_2	12.39	0.7088	0.0001	0.1194	0.0000	0.0596	0.0001	0.0001	7.5188	0.0007	0.6849	1460.00	0.13
S9JS122C_3	8.68	0.7088	0.0001	0.1194	0.0000	0.0604	0.0001	0.0002	6.4516	0.0010	0.9766	1024.00	0.16
S9JS122C_4	11.31	0.7087	0.0001	0.1194	0.0000	0.0601	0.0001	0.0001	8.4317	0.0007	0.7485	1336.00	0.12
S9JS122C_5	34.38	0.7085	0.0001	0.1194	0.0000	0.0577	0.0000	0.0006	0.4274	0.0002	0.2460	4065.00	2.34
S5JS9-2-C1	3.26	0.7100	0.0002	0.1193	0.0000	0.0587	0.0003	0.0057	0.2433	0.0014	1.3965	716.10	4.11
S5JS9-2-C2	3.37	0.7100	0.0003	0.1193	0.0000	0.0585	0.0003	0.0043	0.3155	0.0013	1.3495	741.00	3.17
S5JS9-2-C3	3.18	0.7101	0.0003	0.1193	0.0000	0.0588	0.0003	0.0110	0.1300	0.0014	1.4280	700.30	7.69
S5JS9-2-C4	3.63	0.7099	0.0003	0.1193	0.0000	0.0585	0.0002	0.0042	0.2967	0.0013	1.2516	799.00	3.37
S5JS9-2-C5	3.34	0.7100	0.0003	0.1193	0.0000	0.0587	0.0003	0.0056	0.2415	0.0014	1.3607	734.90	4.14
JS-A-C1	2.43	0.7100	0.0002	0.1193	0.0000	0.0588	0.0004	0.0042	0.3224	0.0014	1.3502	740.63	3.10
JS-A-C2	2.53	0.7097	0.0002	0.1193	0.0000	0.0590	0.0004	0.0024	0.5373	0.0013	1.2967	771.19	1.86
JS-A-C3	2.51	0.7097	0.0003	0.1193	0.0000	0.0590	0.0004	0.0014	0.9059	0.0013	1.3087	764.14	1.10
JS-A-C4	2.60	0.7095	0.0002	0.1193	0.0000	0.0593	0.0004	0.0008	1.6815	0.0013	1.2621	792.35	0.59
JS-A-C5	2.66	0.7095	0.0002	0.1193	0.0000	0.0589	0.0003	0.0001	19.1584	0.0012	1.2355	809.40	0.05
JS-B-C1	2.02	0.7097	0.0003	0.1193	0.0000	0.0584	0.0004	0.0017	0.9425	0.0016	1.6296	613.66	1.06
JS-B-C2	2.07	0.7106	0.0003	0.1193	0.0000	0.0586	0.0004	0.0111	0.1425	0.0016	1.5870	630.12	7.02
JS-B-C3	2.30	0.7097	0.0003	0.1193	0.0000	0.0583	0.0004	0.0031	0.4665	0.0014	1.4296	699.48	2.14
JS-B-C4	2.27	0.7101	0.0003	0.1193	0.0000	0.0583	0.0004	0.0044	0.3306	0.0014	1.4479	690.66	3.02
JS-B-C5	2.26	0.7098	0.0002	0.1193	0.0000	0.0585	0.0004	0.0010	1.4428	0.0015	1.4528	688.31	0.69
S5HD9-6-D1	1.61	0.7092	0.0004	0.1193	0.0000	0.0606	0.0006	0.0053	0.5397	0.0028	2.8425	351.80	1.85
S5HD9-6-D2	1.43	0.7095	0.0005	0.1193	0.0000	0.0611	0.0007	0.0049	0.6452	0.0032	3.1878	313.70	1.55
S5HD9-6-D4	1.77	0.7094	0.0004	0.1193	0.0000	0.0603	0.0005	0.0043	0.5952	0.0026	2.5867	386.60	1.68
S5HD9-6-D5	1.45	0.7092	0.0005	0.1193	0.0000	0.0608	0.0007	0.0081	0.3888	0.0032	3.1546	317.00	2.57
S2HD62D_1	0.73	0.7101	0.0008	0.1194	0.0000	0.0622	0.0012	0.0248	0.2825	0.0070	6.9979	142.90	3.54
S2HD62D_2	0.58	0.7104	0.0009	0.1194	0.0000	0.0656	0.0019	0.0290	0.3040	0.0088	8.8106	113.50	3.29
S2HD62D_3	0.47	0.7101	0.0011	0.1194	0.0000	0.0676	0.0022	0.0109	0.9804	0.0107	10.6860	93.58	1.02
S2HD62D_4	1.28	0.7099	0.0005	0.1194	0.0000	0.0614	0.0006	0.0260	0.1527	0.0040	3.9761	251.50	6.55
S2HD62D_5	0.75	0.7103	0.0007	0.1194	0.0000	0.0644	0.0013	0.0346	0.1961	0.0068	6.7889	147.30	5.10
HM1-A-DA1	2.64	0.7091	0.0002	0.1194	0.0000	0.0560	0.0004	0.0106	0.1176	0.0012	1.2445	803.52	8.50
HM1-A-DA2	2.68	0.7096	0.0002	0.1194	0.0000	0.0557	0.0004	0.0096	0.1281	0.0012	1.2239	817.04	7.81
HM1-A-DA3	2.57	0.7095	0.0003	0.1194	0.0000	0.0557	0.0004	0.0085	0.1511	0.0013	1.2772	782.95	6.62
HM1-A-DA4	3.05	0.7089	0.0002	0.1194	0.0000	0.0562	0.0003	0.0027	0.3975	0.0011	1.0774	928.14	2.52
HM1-A-DA5	3.16	0.7088	0.0002	0.1194	0.0000	0.0561	0.0003	0.0030	0.3422	0.0010	1.0405	961.05	2.92
HM-C-DA1-4	1.53	0.7107	0.0006	0.1194	0.0000	0.0467	0.0007	0.0028	0.8403	0.0023	2.3467	426.13	1.19
HM-C-DA1-5	2.12	0.7105	0.0009	0.1194	0.0000	0.0474	0.0014	0.0005	3.0954	0.0017	1.6934	590.51	0.32
HM1-B-DA1	3.17	0.7110	0.0003	0.1194	0.0000	0.0557	0.0003	0.0240	0.0431	0.0010	1.0348	966.34	23.19
HM1-B-DA2	2.55	0.7097	0.0003	0.1193	0.0000	0.0559	0.0004	0.0089	0.1446	0.0013	1.2879	776.48	6.91
HM1-B-DA4	3.32	0.7095	0.0002	0.1194	0.0000	0.0556	0.0002	0.0093	0.1062	0.0010	0.9880	1012.19	9.41
HM1-B-DA5	1.72	0.7104	0.0005	0.1193	0.0000	0.0556	0.0005	0.0095	0.2015	0.0019	1.9137	522.55	4.96
HM3-B-DA1	3.23	0.7136	0.0005	0.1194	0.0000	0.0556	0.0003	0.0617	0.0165	0.0010	1.0163	983.98	60.75
HM3-B-DA2	3.38	0.7098	0.0002	0.1194	0.0000	0.0558	0.0003	0.0129	0.0753	0.0010	0.9705	1030.41	13.28
HM3-B-DA3	4.39	0.7087	0.0002	0.1194	0.0000	0.0561	0.0002	0.0017	0.4312	0.0007	0.7475	1337.83	2.32
S10HM3A-DA1	6.25	0.7091	0.0003	0.1194	0.0000	0.0569	0.0002	0.0231	0.0327	0.0008	0.7559	1323.00	30.60
S10HM3A-DA2	6.31	0.7091	0.0002	0.1194	0.0000	0.0570	0.0002	0.0194	0.0386	0.0007	0.7491	1335.00	25.90
S10HM3A-DA3	6.71	0.7090	0.0002	0.1194	0.0000	0.0568	0.0001	0.0170	0.0413	0.0007	0.7037	1421.00	24.20
S10HM3A-DA4	6.93	0.7086	0.0002	0.1194	0.0000	0.0570	0.0001	0.0003	2.4155	0.0007	0.6817	1467.00	0.41
S10HM3A-DA5	5.55	0.7091	0.0002	0.1193	0.0000	0.0565	0.0001	0.0246	0.0346	0.0009	0.8518	1174.00	28.90
S10HM1B-DA1	1.70	0.7133	0.0007	0.1193	0.0000	0.0581	0.0006	0.1788	0.0155	0.0028	2.7724	360.70	64.50
S10HM1B-DA2	1.71	0.7139	0.0007	0.1193	0.0000	0.0574	0.0005	0.2094	0.0132	0.0028	2.7586	362.50	75.90
S10HM1B-DA3	1.87	0.7118	0.0006	0.1193	0.0000	0.0579	0.0005	0.1186	0.0213	0.0025	2.5284	395.50	46.90
S10HM1B-DA4	1.53	0.7149	0.0009	0.1193	0.0000	0.0582	0.0006	0.2355	0.0131	0.0031	3.0788	324.80	76.50
S10HM1B-DA5	2.17	0.7096	0.0004	0.1193	0.0000	0.0585	0.0005	0.0280	0.0776	0.0022	2.1725	460.30	12.89
S9HM102DA_1	35.11	0.7085	0.0001	0.1194	0.0000	0.0569	0.0000	0.0016	0.1484	0.0002	0.2388	4187.00	6.74
S9HM102DA_2	28.22	0.7087	0.0001	0.1194	0.0000	0.0569	0.0000	0.0064	0.0463	0.0003	0.2972	3365.00	21.60
S9HM102DA_3	30.90	0.7085	0.0001	0.1194	0.0000	0.0570	0.0000	0.0025	0.1070	0.0003	0.2716	3682.00	9.35
S9HM102DA_4	38.30	0.7085	0.0001	0.1194	0.0000	0.0569	0.0000	0.0003	0.6614	0.0002	0.2192	4562.00	1.51
S9HM102DA_5	37.36	0.7085	0.0001	0.1194	0.0000	0.0569	0.0000	0.0009	0.2584	0.0002	0.2248	4448.00	3.87
S5HM9-3-D1	3.04	0.7092	0.0003	0.1193	0.0000	0.0571	0.0003	0.0061	0.2421	0.0015	1.4667	681.80	4.13
S5HM9-3-D2	4.03	0.7095	0.0003	0.1193	0.0000	0.0567	0.0003	0.0158	0.0699	0.0011	1.1055	904.60	14.30
S5HM9-3-D3	5.46	0.7089	0.0002	0.1194	0.0000	0.0566	0.0002	0.0047	0.1745	0.0008	0.8157	1226.00	5.73
S5HM9-3-D4	4.84	0.7087	0.0003	0.1194	0.0000	0.0565	0.0003	0.0038	0.2445	0.0009	0.9208	1086.00	4.09
S5HM9-3-D5	3.41	0.7095	0.0003	0.1194	0.0000	0.0569	0.0003	0.0167	0.0784	0.0013	1.3053	766.10	12.76
S11HM81D_1	1.68	0.7090	0.0005	0.1194	0.0000	0.0584	0.0006	0.0146	0.2342	0.0034	3.4130	293.00	4.27
S11HM81D_2	7.21	0.7089	0.0002	0.1194	0.0000	0.0570	0.0001	0.0123	0.0645	0.0008	0.7924	1262.00	15.50
S11HM81D_3	8.45	0.7088	0.0002	0.1194	0.0000	0.0569	0.0001	0.0021	0.3279	0.0007	0.6725	1487.00	3.05
S11HM81D_4	1.05	0.7094	0.0006	0.1194	0.0000	0.0587	0.0009	0.0016	3.4247	0.0054	5.3677	186.30	0.29
S11HM81D_5	1.60	0.7088	0.0004	0.1194	0.0000	0.0585	0.0006	0.0010	3.4722	0.0035	3.5162	284.40	0.29
HM1-A-D1	2.26	0.7088	0.0002	0.1193	0.0000	0.0568	0.0003	0.0025	0.5715	0.0015	1.4554	687.08	1.75

Sample	Sr Beam	Sr87_86	2SE	Sr86_88	2SE	Sr84_86	2SE	Rb/Sr	1/Rb	1/Sr	(1/Sr)*1000	Sr_ppm	Rb_ppm
HM1-A-D2	2.72	0.7092	0.0002	0.1193	0.0000	0.0563	0.0003	0.0039	0.3116	0.0012	1.2091	827.03	3.21
HM1-A-D3	2.34	0.7089	0.0002	0.1193	0.0000	0.0566	0.0004	0.0002	5.7008	0.0014	1.4017	713.41	0.18
HM1-A-D4	5.07	0.7087	0.0001	0.1194	0.0000	0.0560	0.0001	0.0005	1.3058	0.0006	0.6476	1544.15	0.77
HM1-A-D5	2.97	0.7091	0.0002	0.1194	0.0000	0.0563	0.0003	0.0045	0.2440	0.0011	1.1069	903.45	4.10
HM-C-D1	3.26	0.7089	0.0002	0.1194	0.0000	0.0480	0.0003	0.0004	3.1391	0.0011	1.1012	908.11	0.32
HM-C-D2	3.16	0.7088	0.0002	0.1194	0.0000	0.0485	0.0003	0.0003	3.8877	0.0011	1.1372	879.38	0.26
HM-C-D3	3.01	0.7088	0.0002	0.1194	0.0000	0.0484	0.0003	0.0003	3.4249	0.0012	1.1935	837.89	0.29
HM-C-D4	3.04	0.7088	0.0002	0.1194	0.0000	0.0487	0.0004	0.0002	4.9602	0.0012	1.1815	846.40	0.20
HM-C-D5	3.30	0.7088	0.0002	0.1194	0.0000	0.0485	0.0003	0.0003	4.2528	0.0011	1.0884	918.75	0.24
HM1-B-D1	1.11	0.7122	0.0005	0.1193	0.0000	0.0566	0.0007	0.0426	0.0692	0.0029	2.9469	339.34	14.44
HM1-B-D2	1.22	0.7109	0.0005	0.1193	0.0000	0.0557	0.0007	0.0201	0.1333	0.0027	2.6834	372.66	7.50
HM1-B-D3	2.82	0.7092	0.0002	0.1194	0.0000	0.0558	0.0003	0.0045	0.2586	0.0012	1.1639	859.19	3.87
S10HM1A-D1	2.00	0.7104	0.0004	0.1192	0.0000	0.0549	0.0005	0.0497	0.0474	0.0024	2.3590	423.90	21.08
S10HM1A-D2	1.52	0.7094	0.0004	0.1193	0.0000	0.0583	0.0007	0.0232	0.1339	0.0031	3.1075	321.80	7.47
S10HM1A-D3	3.05	0.7085	0.0003	0.1193	0.0000	0.0573	0.0003	0.0004	3.9526	0.0016	1.5504	645.00	0.25
S10HM1A-D4	2.58	0.7090	0.0003	0.1193	0.0000	0.0578	0.0004	0.0080	0.2278	0.0018	1.8295	546.60	4.39
S10HM1A-D5	2.25	0.7090	0.0003	0.1193	0.0000	0.0581	0.0004	0.0030	0.7042	0.0021	2.1013	475.90	1.42
S2HM72D_1	1.70	0.7089	0.0004	0.1194	0.0000	0.0586	0.0006	0.0019	1.5649	0.0030	2.9895	334.50	0.64
S2HM72D_2	1.97	0.7087	0.0003	0.1194	0.0000	0.0588	0.0005	0.0002	10.3520	0.0026	2.5840	387.00	0.10
S2HM72D_3	1.85	0.7089	0.0004	0.1194	0.0000	0.0587	0.0005	0.0009	3.1447	0.0027	2.7480	363.90	0.32
S2HM72D_4	1.70	0.7090	0.0004	0.1194	0.0000	0.0590	0.0005	0.0026	1.1351	0.0030	2.9771	335.90	0.88
S2HM72D_5	1.72	0.7090	0.0004	0.1194	0.0000	0.0583	0.0005	0.0018	1.6667	0.0029	2.9334	340.90	0.60
S11HM85D_1	4.75	0.7087	0.0002	0.1194	0.0000	0.0579	0.0002	0.0008	1.3263	0.0011	1.1109	900.20	0.75
S11HM85D_2	3.80	0.7088	0.0002	0.1194	0.0000	0.0584	0.0002	0.0032	0.4292	0.0014	1.3829	723.10	2.33
S11HM85D_3	5.09	0.7088	0.0002	0.1194	0.0000	0.0573	0.0002	0.0025	0.4098	0.0010	1.0260	974.70	2.44
S11HM85D_4	4.26	0.7090	0.0002	0.1194	0.0000	0.0575	0.0002	0.0032	0.3759	0.0012	1.2192	820.20	2.66
S11HM85D_5	5.20	0.7090	0.0002	0.1194	0.0000	0.0573	0.0002	0.0038	0.2597	0.0010	0.9952	1004.80	3.85
HM3-A-D1	1.45	0.7085	0.0003	0.1194	0.0000	0.0569	0.0006	0.0020	1.1402	0.0023	2.2732	439.91	0.88
HM3-A-D2	1.39	0.7093	0.0004	0.1194	0.0000	0.0563	0.0007	0.0099	0.2390	0.0024	2.3635	423.10	4.18
HM3-A-D3	1.52	0.7092	0.0003	0.1193	0.0000	0.0562	0.0006	0.0054	0.3968	0.0022	2.1562	463.77	2.52
HM3-A-D4	1.70	0.7091	0.0004	0.1194	0.0000	0.0563	0.0006	0.0049	0.3942	0.0019	1.9311	517.85	2.54
HM3-A-D5	1.66	0.7090	0.0004	0.1193	0.0000	0.0557	0.0006	0.0008	2.5573	0.0020	1.9828	504.33	0.39
HM3-B-D1	2.55	0.7108	0.0003	0.1193	0.0000	0.0553	0.0004	0.0176	0.0733	0.0013	1.2888	775.90	13.65
HM3-B-D2	2.17	0.7118	0.0004	0.1193	0.0000	0.0559	0.0004	0.0376	0.0403	0.0015	1.5160	659.63	24.81
HM3-B-D3	2.72	0.7099	0.0002	0.1194	0.0000	0.0562	0.0003	0.0130	0.0931	0.0012	1.2083	827.62	10.74
S10HM3A-D1	6.84	0.7084	0.0002	0.1194	0.0000	0.0567	0.0001	0.0000	14.2045	0.0007	0.6916	1446.00	0.07
S10HM3A-D2	6.10	0.7085	0.0002	0.1194	0.0000	0.0571	0.0002	0.0001	13.7363	0.0008	0.7748	1290.60	0.07
S10HM3A-D3	8.31	0.7085	0.0002	0.1194	0.0000	0.0568	0.0001	0.0001	10.4275	0.0006	0.5692	1757.00	0.10
S10HM3A-D4	6.79	0.7086	0.0002	0.1194	0.0000	0.0569	0.0001	0.0002	4.1322	0.0007	0.6962	1436.30	0.24
S10HM3A-D5	3.96	0.7085	0.0003	0.1194	0.0000	0.0579	0.0002	0.0003	4.5872	0.0012	1.1922	838.80	0.22
S9HM102D_1	44.61	0.7084	0.0001	0.1194	0.0000	0.0569	0.0000	0.0000	6.0096	0.0002	0.1887	5300.00	0.17
S9HM102D_3	28.10	0.7085	0.0001	0.1194	0.0000	0.0574	0.0001	0.0000	14.6628	0.0003	0.2994	3340.00	0.07
S9HM102D_4	34.89	0.7085	0.0001	0.1194	0.0000	0.0569	0.0000	0.0001	3.9526	0.0002	0.2414	4143.00	0.25
S9HM102D_5	35.24	0.7085	0.0001	0.1194	0.0000	0.0569	0.0000	0.0001	3.4965	0.0002	0.2392	4180.00	0.29
S5HM9-4-D1	10.11	0.7086	0.0002	0.1194	0.0000	0.0566	0.0001	0.0035	0.1279	0.0004	0.4456	2244.00	7.82
S5HM9-4-D2	10.12	0.7086	0.0002	0.1194	0.0000	0.0566	0.0001	0.0022	0.2049	0.0004	0.4446	2249.00	4.88
S5HM9-4-D3	10.42	0.7087	0.0002	0.1194	0.0000	0.0567	0.0001	0.0032	0.1340	0.0004	0.4320	2315.00	7.46
S5HM9-4-D4	10.60	0.7086	0.0002	0.1194	0.0000	0.0564	0.0001	0.0038	0.1129	0.0004	0.4243	2357.00	8.86
S5HM9-4-D5	10.63	0.7087	0.0002	0.1194	0.0000	0.0565	0.0001	0.0045	0.0943	0.0004	0.4227	2366.00	10.61
S9JS1010D_1	9.47	0.7094	0.0001	0.1194	0.0000	0.0582	0.0001	0.0194	0.0455	0.0009	0.8834	1132.00	21.98
S9JS1010D_2	9.07	0.7092	0.0001	0.1194	0.0000	0.0585	0.0001	0.0189	0.0487	0.0009	0.9225	1084.00	20.52
S9JS1010D_3	10.53	0.7090	0.0001	0.1194	0.0000	0.0585	0.0001	0.0091	0.0875	0.0008	0.7949	1258.00	11.43
S9JS1010D_4	11.11	0.7090	0.0001	0.1194	0.0000	0.0584	0.0001	0.0083	0.0912	0.0008	0.7530	1328.00	10.96
S9JS1010D_5	10.99	0.7088	0.0001	0.1194	0.0000	0.0584	0.0001	0.0091	0.0839	0.0008	0.7610	1314.00	11.92
S11JS87WD_1	2.44	0.7087	0.0003	0.1194	0.0000	0.0596	0.0004	0.0000	57.8035	0.0027	2.7211	367.50	0.02
S11JS87WD_2	2.27	0.7086	0.0003	0.1194	0.0000	0.0599	0.0004	0.0001	21.5054	0.0029	2.9146	343.10	0.05
S11JS87WD_3	2.35	0.7086	0.0003	0.1194	0.0000	0.0597	0.0004	0.0001	32.2581	0.0028	2.8027	356.80	0.03
S11JS87WD_4	2.40	0.7087	0.0003	0.1194	0.0000	0.0596	0.0004	0.0019	1.4493	0.0027	2.7360	365.50	0.69
S11JS87WD_5	2.53	0.7086	0.0003	0.1194	0.0000	0.0599	0.0004	0.0000	114.9425	0.0026	2.5900	386.10	0.01
S11JS89D_1	0.78	0.7102	0.0007	0.1194	0.0000	0.0670	0.0011	0.0166	0.4753	0.0079	7.9114	126.40	2.10
S11JS89D_2	1.20	0.7103	0.0006	0.1194	0.0000	0.0632	0.0008	0.0094	0.5464	0.0051	5.1467	194.30	1.83
S11JS89D_3	1.35	0.7106	0.0005	0.1194	0.0000	0.0628	0.0006	0.0236	0.1931	0.0045	4.5496	219.80	5.18
S11JS89D_4	1.39	0.7103	0.0005	0.1194	0.0000	0.0630	0.0006	0.0211	0.2096	0.0044	4.4131	226.60	4.77
S11JS89D_5	0.84	0.7103	0.0007	0.1194	0.0000	0.0642	0.0011	0.0462	0.1570	0.0073	7.2516	137.90	6.37

Table S4-3. LA-MC-ICP-MS Strontium isotope analyses data of reference materials.

Reference	Sr Beam Int.	87Sr/86Sr ratio	87Sr/86Sr 2SE	86Sr/88Sr	86Sr/88Sr 2SE	84Sr/86Sr	84Sr/86Sr 2SE	Sr_ppm	Rb_ppm
C_KRNC1_1	3.94	0.70876	0.00017	0.1194	0.0000	0.0599	0.0002	601.90	0.0021
C_KRNC1_2	3.66	0.70850	0.00014	0.1194	0.0000	0.0598	0.0002	563.90	0.0027
C_KRNC1_3	3.66	0.70858	0.00017	0.1194	0.0000	0.0597	0.0003	567.30	0.0064
C_KRNC1_4	3.59	0.70852	0.00017	0.1194	0.0000	0.0597	0.0003	558.30	0.0035
C_KRNC1_5	3.57	0.70862	0.00014	0.1194	0.0000	0.0595	0.0003	556.30	0.0025
C_KRNC1_6	3.59	0.70864	0.00015	0.1194	0.0000	0.0598	0.0003	554.10	0.0064
C_KRNC1_7	6.04	0.70862	0.00013	0.1194	0.0000	0.0623	0.0001	742.30	0.0073
C_KRNC1_8	6.18	0.70861	0.00011	0.1194	0.0000	0.0624	0.0001	729.10	0.0053
C_KRNC1_9	6.33	0.70866	0.00013	0.1194	0.0000	0.0623	0.0001	730.40	0.0050
C_KRNC1_10	6.29	0.70856	0.00011	0.1194	0.0000	0.0622	0.0002	728.90	0.0066
C_KRNC1_11	6.14	0.70855	0.00013	0.1194	0.0000	0.0623	0.0002	723.80	0.0077
C_KRNC1_12	6.17	0.70866	0.00011	0.1194	0.0000	0.0626	0.0002	733.50	0.0061
C_KRNC1_13	6.07	0.70855	0.00012	0.1194	0.0000	0.0624	0.0002	722.00	0.0050
C_KRNC1_14	5.93	0.70862	0.00011	0.1194	0.0000	0.0623	0.0002	702.10	0.0048
C_KRNC1_15	5.95	0.70857	0.00012	0.1194	0.0000	0.0621	0.0002	698.10	0.0072
C_KRNC1_16	5.71	0.70860	0.00013	0.1194	0.0000	0.0621	0.0002	676.10	0.0023
C_KRNC1_17	5.59	0.70858	0.00013	0.1194	0.0000	0.0624	0.0002	665.30	0.0027
C_KRNC1_18	5.70	0.70869	0.00011	0.1194	0.0000	0.0622	0.0002	681.00	0.0030
C_KRNC1_19	5.43	0.70849	0.00011	0.1194	0.0000	0.0620	0.0002	648.40	0.0035
C_KRNC1_20	5.28	0.70860	0.00013	0.1194	0.0000	0.0617	0.0002	628.00	0.0070
C_KRNC1_21	5.37	0.70870	0.00012	0.1194	0.0000	0.0621	0.0002	635.90	0.4400
C_KRNC1_1	4.07	0.70859	0.00024	0.1194	0.0000	0.0608	0.0002	561.70	0.3280
C_KRNC1_2	3.12	0.70864	0.00021	0.1194	0.0000	0.0609	0.0003	431.70	0.2190
C_KRNC1_3	4.25	0.70857	0.00019	0.1194	0.0000	0.0606	0.0002	610.60	0.0137
C_KRNC1_4	3.90	0.70860	0.00019	0.1194	0.0000	0.0606	0.0002	576.20	0.0258
C_KRNC1_5	3.88	0.70862	0.00022	0.1194	0.0000	0.0605	0.0002	596.40	0.0103
C_KRNC1_6	3.82	0.70863	0.00031	0.1194	0.0000	0.0606	0.0003	605.40	0.0164
C_KRNC1_7	3.63	0.70860	0.00022	0.1194	0.0000	0.0609	0.0003	600.60	0.0037
C_KRNC1_8	3.57	0.70861	0.00030	0.1194	0.0000	0.0606	0.0003	609.30	0.0069
C_KRNC1_9	3.46	0.70862	0.00023	0.1194	0.0000	0.0610	0.0003	618.10	0.0061
C_KRNC1_10	3.54	0.70857	0.00021	0.1194	0.0000	0.0607	0.0003	654.10	0.0028
C_KRNC1_11	3.08	0.70862	0.00023	0.1194	0.0000	0.0606	0.0003	599.00	0.0041
C_KRNC1_1	2.78	0.70861	0.00029	0.1194	0.0000	0.0599	0.0003	574.80	0.0142
C_KRNC1_2	2.78	0.70858	0.00031	0.1194	0.0000	0.0601	0.0003	556.20	0.0021
C_KRNC1_3	2.84	0.70857	0.00028	0.1194	0.0000	0.0604	0.0004	556.50	0.0049
C_KRNC1_4	2.82	0.70865	0.00027	0.1194	0.0000	0.0609	0.0003	538.70	0.0028
C_KRNC1_5	2.82	0.70872	0.00025	0.1194	0.0000	0.0606	0.0003	533.90	0.0070
C_KRNC1_6	2.80	0.70845	0.00026	0.1194	0.0000	0.0608	0.0003	537.90	-0.0001
C_KRNC1_7	2.81	0.70859	0.00024	0.1194	0.0000	0.0609	0.0003	548.20	0.0048
C_KRNC1_8	2.75	0.70866	0.00028	0.1194	0.0000	0.0604	0.0004	542.20	0.0033
C_KRNC1_9	2.80	0.70857	0.00029	0.1194	0.0000	0.0605	0.0003	551.30	0.0011
C_KRNC1_10	2.85	0.70857	0.00029	0.1194	0.0000	0.0600	0.0003	556.30	0.0017
C_KRNC1_11	2.88	0.70866	0.00023	0.1194	0.0000	0.0607	0.0003	561.60	0.0062
C_KRNC1_12	2.73	0.70862	0.00028	0.1194	0.0000	0.0604	0.0004	536.80	0.0030
C_KRNC1_13	2.72	0.70856	0.00029	0.1194	0.0000	0.0604	0.0004	538.30	0.0081
C_KRNC1_1	2.50	0.70854	0.00033	0.1193	0.0000	0.0603	0.0004	527.90	0.0045
C_KRNC1_2	2.51	0.70864	0.00032	0.1193	0.0000	0.0604	0.0004	531.50	0.0050
C_KRNC1_3	2.50	0.70862	0.00031	0.1193	0.0000	0.0603	0.0004	528.60	0.0051
C_KRNC1_4	2.61	0.70847	0.00030	0.1193	0.0000	0.0603	0.0004	551.10	0.0072
C_KRNC1_5	2.66	0.70868	0.00033	0.1193	0.0000	0.0600	0.0004	562.90	0.0028
C_KRNC1_6	2.58	0.70849	0.00031	0.1193	0.0000	0.0607	0.0004	547.00	0.0022
C_KRNC1_7	2.55	0.70884	0.00030	0.1193	0.0000	0.0607	0.0003	540.90	0.0032
C_KRNC1_8	2.54	0.70864	0.00027	0.1193	0.0000	0.0601	0.0004	538.20	0.0097
C_KRNC1_9	2.55	0.70856	0.00030	0.1193	0.0000	0.0606	0.0004	540.60	0.0136
C_KRNC1_10	2.59	0.70859	0.00025	0.1193	0.0000	0.0602	0.0003	549.70	0.0086
C_KRNC1_11	2.61	0.70860	0.00028	0.1193	0.0000	0.0600	0.0003	553.10	0.0013
C_KRNC1_12	2.53	0.70855	0.00030	0.1193	0.0000	0.0602	0.0003	537.20	0.0007
C_KRNC1_13	2.52	0.70870	0.00029	0.1193	0.0000	0.0601	0.0003	533.90	0.0050
C_KRNC1_14	2.39	0.70869	0.00029	0.1193	0.0000	0.0603	0.0004	506.40	0.0031
C_KRNC1_15	2.34	0.70840	0.00033	0.1193	0.0000	0.0601	0.0004	495.80	0.0106
C_KRNC1_1	2.33	0.70864	0.00031	0.1193	0.0000	0.0600	0.0004	506.10	0.0046
C_KRNC1_2	2.36	0.70855	0.00035	0.1193	0.0000	0.0602	0.0004	512.00	0.0146
C_KRNC1_3	2.41	0.70863	0.00032	0.1193	0.0000	0.0600	0.0004	525.40	0.0042
C_KRNC1_4	2.30	0.70860	0.00036	0.1193	0.0000	0.0597	0.0004	505.30	0.0034
C_KRNC1_5	2.31	0.70859	0.00032	0.1193	0.0000	0.0599	0.0004	510.10	0.0084
C_KRNC1_6	2.21	0.70860	0.00038	0.1193	0.0000	0.0599	0.0005	490.10	0.0056
C_KRNC1_7	2.30	0.70866	0.00036	0.1193	0.0000	0.0598	0.0004	511.70	0.0100
C_KRNC1_8	2.27	0.70854	0.00033	0.1193	0.0000	0.0600	0.0004	507.30	0.0072
C_KRNC1_9	2.25	0.70864	0.00031	0.1193	0.0000	0.0595	0.0004	506.30	0.0065
C_KRNC1_10	2.21	0.70858	0.00036	0.1193	0.0000	0.0596	0.0005	497.50	0.0028
C_KRNC1_11	2.21	0.70865	0.00036	0.1193	0.0000	0.0597	0.0005	501.40	0.0058
C_KRNC1_12	2.23	0.70857	0.00035	0.1193	0.0000	0.0595	0.0004	507.20	0.0076
C_KRNC1_13	2.23	0.70859	0.00029	0.1193	0.0000	0.0592	0.0005	511.40	0.0109
C_KRNC1_14	2.26	0.70862	0.00041	0.1193	0.0000	0.0596	0.0005	520.90	0.0058
C_UKC1_1	3.94	0.70745	0.00017	0.1194	0.0000	0.0592	0.0002	607.60	0.0047
C_UKC1_2	3.88	0.70743	0.00018	0.1194	0.0000	0.0593	0.0002	603.00	0.0052
C_UKC1_3	3.85	0.70753	0.00016	0.1194	0.0000	0.0593	0.0002	593.20	0.0066
C_UKC1_4	6.79	0.70744	0.00012	0.1194	0.0000	0.0614	0.0002	797.60	0.0062
C_UKC1_5	6.50	0.70750	0.00012	0.1194	0.0000	0.0615	0.0001	754.10	0.0044
C_UKC1_6	6.36	0.70750	0.00010	0.1194	0.0000	0.0614	0.0002	756.70	0.0043
C_UKC1_7	6.40	0.70750	0.00012	0.1194	0.0000	0.0616	0.0001	756.70	0.0043
C_UKC1_8	6.48	0.70743	0.00012	0.1194	0.0000	0.0614	0.0002	760.30	0.0021
C_UKC1_9	6.53	0.70753	0.00012	0.1194	0.0000	0.0612	0.0002	778.70	0.0024
C_UKC1_10	6.75	0.70740	0.00013	0.1194	0.0000	0.0611	0.0001	806.20	0.0043
C_UKC1_11	5.89	0.70744	0.00013	0.1194	0.0000	0.0610	0.0001	697.00	0.0295
C_UKC1_1	5.16	0.70760	0.00017	0.1194	0.0000	0.0603	0.0002	716.90	0.0051
C_UKC1_2	4.55	0.70757	0.00021	0.1194	0.0000	0.0607	0.0002	674.40	0.0040
C_UKC1_3	4.74	0.70750	0.00021	0.1194	0.0000	0.0602	0.0002	753.40	0.0062

Reference	Sr Beam Int.	87Sr/86Sr ratio	87Sr/86Sr 2SE	86Sr/88Sr	86Sr/88Sr 2SE	84Sr/86Sr	84Sr/86Sr 2SE	Sr_ppm	Rb_ppm
C_UKC1_4	3.74	0.70761	0.00026	0.1194	0.0000	0.0609	0.0002	640.10	0.0063
C_UKC1_5	4.27	0.70744	0.00022	0.1194	0.0000	0.0602	0.0002	794.10	0.0044
C_UKC1_1	3.55	0.70745	0.00023	0.1194	0.0000	0.0594	0.0003	730.20	0.0060
C_UKC1_2	3.42	0.70749	0.00025	0.1194	0.0000	0.0600	0.0003	667.70	0.0060
C_UKC1_3	3.47	0.70735	0.00025	0.1194	0.0000	0.0602	0.0003	657.20	0.0041
C_UKC1_4	3.50	0.70754	0.00024	0.1194	0.0000	0.0601	0.0003	683.10	0.0034
C_UKC1_5	3.31	0.70754	0.00024	0.1194	0.0000	0.0598	0.0003	651.30	0.0018
C_UKC1_6	3.25	0.70766	0.00026	0.1194	0.0000	0.0598	0.0003	633.10	0.0049
C_UKC1_7	3.11	0.70752	0.00027	0.1194	0.0000	0.0601	0.0003	618.00	0.0054
C_UKC1_1	2.46	0.70740	0.00027	0.1193	0.0000	0.0602	0.0004	520.30	0.0078
C_UKC1_2	3.04	0.70759	0.00028	0.1193	0.0000	0.0600	0.0004	642.30	0.0019
C_UKC1_3	2.91	0.70743	0.00025	0.1193	0.0000	0.0599	0.0003	616.10	0.0057
C_UKC1_4	2.96	0.70751	0.00030	0.1193	0.0000	0.0597	0.0003	626.60	0.0039
C_UKC1_5	2.82	0.70748	0.00031	0.1193	0.0000	0.0599	0.0003	598.40	0.0022
C_UKC1_6	2.73	0.70748	0.00027	0.1193	0.0000	0.0595	0.0003	577.70	0.0056
C_UKC1_7	2.57	0.70740	0.00030	0.1193	0.0000	0.0603	0.0004	545.10	0.0018
C_UKC1_1	2.61	0.70784	0.00036	0.1193	0.0000	0.0596	0.0003	567.10	0.0047
C_UKC1_2	2.79	0.70728	0.00030	0.1193	0.0000	0.0595	0.0004	613.00	0.0012
C_UKC1_3	2.83	0.70734	0.00029	0.1193	0.0000	0.0593	0.0004	626.10	0.0062
C_UKC1_4	2.83	0.70745	0.00028	0.1193	0.0000	0.0591	0.0004	633.80	0.0029
C_UKC1_5	2.80	0.70747	0.00031	0.1193	0.0000	0.0592	0.0004	633.30	0.0023
C_UKC1_6	2.83	0.70758	0.00034	0.1193	0.0000	0.0587	0.0003	645.80	0.0054
DGC_1	1.92	0.71018	0.00030	0.1194	0.0000	0.0633	0.0004	295.50	0.0410
DGC_2	1.86	0.70994	0.00031	0.1194	0.0000	0.0636	0.0006	289.90	0.0151
DGC_3	1.93	0.71031	0.00033	0.1194	0.0000	0.0641	0.0004	295.60	0.3000
DGC_4	3.16	0.71019	0.00018	0.1194	0.0000	0.0689	0.0003	369.20	0.0083
DGC_5	3.14	0.71016	0.00017	0.1194	0.0000	0.0692	0.0003	364.60	0.0057
DGC_6	3.14	0.71010	0.00020	0.1194	0.0000	0.0693	0.0003	373.20	0.0062
DGC_7	3.22	0.71022	0.00018	0.1194	0.0000	0.0687	0.0003	379.60	0.0091
DGC_8	3.11	0.71024	0.00018	0.1194	0.0000	0.0684	0.0003	365.30	0.4390
DGC_9	3.17	0.71032	0.00019	0.1194	0.0000	0.0680	0.0003	378.40	0.5350
DGC_10	3.18	0.71026	0.00019	0.1194	0.0000	0.0686	0.0003	379.30	0.0068
DGC_11	3.16	0.71033	0.00018	0.1194	0.0000	0.0684	0.0003	374.10	0.0164
DGC_1	2.80	0.70989	0.00024	0.1194	0.0000	0.0660	0.0004	390.50	0.0056
DGC_2	2.57	0.71009	0.00025	0.1194	0.0000	0.0654	0.0004	382.40	0.0079
DGC_3	2.43	0.71014	0.00031	0.1194	0.0000	0.0656	0.0004	388.50	0.0059
DGC_4	2.27	0.71007	0.00035	0.1194	0.0000	0.0655	0.0004	390.90	0.0108
DGC_5	2.14	0.71011	0.00029	0.1194	0.0000	0.0656	0.0004	399.80	0.0980
DGC_1	1.74	0.70984	0.00033	0.1194	0.0000	0.0641	0.0005	356.60	0.0100
DGC_2	1.83	0.71020	0.00037	0.1194	0.0000	0.0657	0.0005	356.10	0.0080
DGC_3	1.75	0.70989	0.00036	0.1194	0.0000	0.0658	0.0005	332.00	0.0060
DGC_4	1.69	0.71008	0.00038	0.1194	0.0000	0.0659	0.0006	331.20	0.0030
DGC_5	1.60	0.70994	0.00045	0.1194	0.0000	0.0650	0.0006	314.80	0.0034
DGC_6	1.68	0.71011	0.00041	0.1194	0.0000	0.0649	0.0006	326.60	0.2770
DGC_7	1.68	0.71012	0.00039	0.1194	0.0000	0.0650	0.0006	334.60	0.3770
DGC_1	1.54	0.71055	0.00043	0.1192	0.0000	0.0642	0.0006	324.90	0.0199
DGC_2	1.64	0.71075	0.00038	0.1192	0.0000	0.0645	0.0007	346.50	0.0065
DGC_3	1.54	0.71058	0.00037	0.1192	0.0000	0.0643	0.0006	326.20	0.0070
DGC_4	1.60	0.71044	0.00036	0.1192	0.0000	0.0648	0.0006	338.60	0.0030
DGC_5	1.50	0.71075	0.00041	0.1192	0.0000	0.0640	0.0007	317.10	0.0118
DGC_6	1.51	0.71071	0.00050	0.1192	0.0000	0.0646	0.0006	319.40	0.1090
DGC_7	1.48	0.71060	0.00049	0.1192	0.0000	0.0645	0.0006	314.40	0.0159
DGC_1	1.37	0.71037	0.00051	0.1192	0.0000	0.0645	0.0006	298.40	0.3030
DGC_2	1.49	0.71055	0.00043	0.1192	0.0000	0.0648	0.0006	326.80	0.0630
DGC_3	1.47	0.71067	0.00046	0.1192	0.0000	0.0640	0.0008	325.50	0.2450
DGC_4	1.42	0.71069	0.00047	0.1192	0.0000	0.0640	0.0007	316.30	0.1900
DGC_5	1.49	0.71037	0.00045	0.1192	0.0000	0.0633	0.0007	335.70	0.0072
DGC_6	1.45	0.71061	0.00053	0.1192	0.0000	0.0633	0.0007	331.70	0.0103
C_KRNC1_1	3.3	0.708970	0.000210	0.1193	0.0000	0.0538	0.0003		
C_KRNC1_2	4.0	0.708500	0.000160	0.1193	0.0000	0.0543	0.0002		
C_KRNC1_3	4.0	0.708610	0.000180	0.1193	0.0000	0.0542	0.0002		
C_KRNC1_4	4.0	0.708570	0.000160	0.1193	0.0000	0.0543	0.0003		
C_KRNC1_5	3.9	0.708520	0.000180	0.1193	0.0000	0.0545	0.0003		
C_KRNC1_6	3.9	0.708620	0.000160	0.1193	0.0000	0.0546	0.0002		
C_KRNC1_7	3.9	0.708450	0.000170	0.1193	0.0000	0.0546	0.0002		
C_KRNC1_8	3.9	0.708650	0.000180	0.1193	0.0000	0.0541	0.0002		
C_KRNC1_9	3.9	0.708660	0.000200	0.1193	0.0000	0.0540	0.0003		
C_KRNC1_10	3.9	0.708660	0.000170	0.1193	0.0000	0.0539	0.0002		
C_KRNC1_11	3.9	0.708570	0.000170	0.1193	0.0000	0.0540	0.0002		
C_KRNC1_12	4.0	0.708660	0.000190	0.1193	0.0000	0.0536	0.0002		
C_KRNC1_13	3.9	0.708620	0.000170	0.1193	0.0000	0.0538	0.0003		
C_KRNC1_14	3.8	0.708700	0.000180	0.1193	0.0000	0.0535	0.0002		
C_KRNC1_15	3.9	0.708550	0.000190	0.1193	0.0000	0.0537	0.0003		
C_KRNC1_16	3.9	0.708630	0.000180	0.1193	0.0000	0.0535	0.0002		
C_KRNC1_17	3.9	0.708430	0.000180	0.1193	0.0000	0.0534	0.0002		
C_KRNC1_18	3.6	0.708650	0.000170	0.1193	0.0000	0.0531	0.0003		
C_KRNC1_19	3.7	0.708560	0.000190	0.1193	0.0000	0.0537	0.0003		
C_KRNC1_20	3.8	0.708580	0.000180	0.1193	0.0000	0.0534	0.0003		
C_KRNC1_21	3.6	0.708730	0.000190	0.1193	0.0000	0.0532	0.0003		
C_KRNC1_22	3.7	0.708650	0.000190	0.1193	0.0000	0.0534	0.0003		
C_KRNC1_23	3.6	0.708530	0.000180	0.1193	0.0000	0.0533	0.0003		
C_KRNC1_24	3.6	0.708520	0.000200	0.1193	0.0000	0.0530	0.0003		
C_KRNC1_25	3.6	0.708670	0.000180	0.1193	0.0000	0.0534	0.0003		
C_KRNC1_1	3.6	0.708670	0.000180	0.1194	0.0000	0.0507	0.0003		
C_KRNC1_2	3.6	0.708600	0.000160	0.1194	0.0000	0.0511	0.0003		
C_KRNC1_3	3.5	0.708610	0.000160	0.1194	0.0000	0.0517	0.0003		
C_KRNC1_4	3.5	0.708700	0.000160	0.1194	0.0000	0.0513	0.0003		
C_KRNC1_5	3.5	0.708370	0.000170	0.1194	0.0000	0.0508	0.0003		
C_KRNC1_6	3.5	0.708660	0.000160	0.1194	0.0000	0.0507	0.0003		
C_KRNC1_7	3.4	0.708580	0.000170	0.1194	0.0000	0.0511	0.0003		

Reference	Sr Beam Int.	87Sr/86Sr ratio	87Sr/86Sr 2SE	86Sr/88Sr	86Sr/88Sr 2SE	84Sr/86Sr	84Sr/86Sr 2SE	Sr_ppm	Rb_ppm
C_KRNC1_8	3.3	0.708700	0.000160	0.1194	0.0000	0.0489	0.0003		
C_KRNC1_9	3.1	0.708570	0.000180	0.1194	0.0000	0.0481	0.0003		
C_KRNC1_10	3.2	0.708630	0.000190	0.1194	0.0000	0.0486	0.0003		
C_KRNC1_11	3.0	0.708410	0.000200	0.1194	0.0000	0.0487	0.0004		
C_KRNC1_12	3.0	0.708900	0.000170	0.1194	0.0000	0.0487	0.0003		
C_KRNC1_13	2.9	0.708460	0.000170	0.1194	0.0000	0.0479	0.0004		
C_KRNC1_14	3.1	0.708400	0.000200	0.1194	0.0000	0.0483	0.0003		
C_KRNC1_15	3.0	0.708800	0.000200	0.1194	0.0000	0.0481	0.0003		
C_KRNC1_16	3.0	0.708520	0.000190	0.1194	0.0000	0.0482	0.0003		
C_KRNC1_17	3.2	0.708650	0.000190	0.1194	0.0000	0.0477	0.0003		
C_KRNC1_18	2.9	0.708750	0.000200	0.1194	0.0000	0.0481	0.0003		
C_KRNC1_19	2.9	0.708450	0.000180	0.1194	0.0000	0.0477	0.0003		
C_KRNC1_20	2.6	0.708580	0.000240	0.1194	0.0000	0.0471	0.0004		
C_KRNC1_21	2.6	0.708670	0.000230	0.1194	0.0000	0.0469	0.0004		
C_KRNC1_22	2.5	0.708700	0.000240	0.1194	0.0000	0.0469	0.0004		
C_KRNC1_23	2.4	0.708540	0.000240	0.1194	0.0000	0.0460	0.0004		
C_KRNC1_24	2.4	0.708650	0.000230	0.1194	0.0000	0.0451	0.0005		
C_KRNC1_25	2.4	0.708550	0.000220	0.1194	0.0000	0.0454	0.0004		
C_KRNC1_1	2.7	0.708550	0.000240	0.1193	0.0000	0.0580	0.0003		
C_KRNC1_2	2.8	0.708610	0.000220	0.1193	0.0000	0.0580	0.0003		
C_KRNC1_3	2.8	0.708600	0.000230	0.1193	0.0000	0.0582	0.0003		
C_KRNC1_4	2.6	0.708640	0.000210	0.1193	0.0000	0.0583	0.0004		
C_KRNC1_5	2.7	0.708600	0.000230	0.1193	0.0000	0.0581	0.0003		
C_KRNC1_6	2.6	0.708650	0.000230	0.1193	0.0000	0.0584	0.0003		
C_KRNC1_7	2.6	0.708650	0.000260	0.1193	0.0000	0.0580	0.0003		
C_KRNC1_8	2.6	0.708580	0.000240	0.1193	0.0000	0.0581	0.0004		
C_KRNC1_9	2.6	0.708490	0.000250	0.1193	0.0000	0.0582	0.0003		
C_KRNC1_10	2.6	0.708620	0.000210	0.1193	0.0000	0.0581	0.0004		
C_KRNC1_11	2.5	0.708540	0.000260	0.1193	0.0000	0.0579	0.0004		
C_KRNC1_12	2.5	0.708700	0.000240	0.1193	0.0000	0.0582	0.0003		
C_KRNC1_13	2.5	0.708550	0.000270	0.1193	0.0000	0.0582	0.0004		
C_KRNC1_14	2.5	0.708590	0.000230	0.1193	0.0000	0.0583	0.0004		
C_KRNC1_15	2.5	0.708620	0.000230	0.1193	0.0000	0.0577	0.0003		
C_KRNC1_16	2.5	0.708640	0.000260	0.1193	0.0000	0.0581	0.0003		
C_KRNC1_17	2.5	0.708620	0.000260	0.1193	0.0000	0.0582	0.0004		
C_KRNC1_18	2.5	0.708520	0.000270	0.1194	0.0000	0.0585	0.0004		
C_KRNC1_19	2.5	0.708700	0.000280	0.1193	0.0000	0.0580	0.0003		
C_KRNC1_20	2.5	0.708630	0.000220	0.1193	0.0000	0.0580	0.0004		
C_KRNC1_21	2.4	0.708470	0.000260	0.1193	0.0000	0.0579	0.0004		
C_KRNC1_22	2.4	0.708690	0.000240	0.1193	0.0000	0.0579	0.0004		
C_KRNC1_23	2.4	0.708580	0.000240	0.1193	0.0000	0.0578	0.0004		
C_KRNC1_24	2.5	0.708600	0.000220	0.1193	0.0000	0.0584	0.0004		
C_KRNC1_25	2.4	0.708510	0.000280	0.1193	0.0000	0.0581	0.0004		
C_KRNC1_26	2.5	0.708720	0.000250	0.1193	0.0000	0.0579	0.0004		
C_KRNC1_27	2.5	0.708610	0.000220	0.1193	0.0000	0.0580	0.0003		
C_KRNC1_28	2.5	0.708570	0.000240	0.1193	0.0000	0.0580	0.0003		
C_UKC1_1	4.6	0.707400	0.000160	0.1193	0.0000	0.0541	0.0002		
C_UKC1_2	4.7	0.707490	0.000130	0.1193	0.0000	0.0539	0.0002		
C_UKC1_3	4.5	0.707290	0.000170	0.1193	0.0000	0.0542	0.0002		
C_UKC1_4	4.3	0.707470	0.000150	0.1193	0.0000	0.0537	0.0002		
C_UKC1_5	4.3	0.707470	0.000150	0.1193	0.0000	0.0536	0.0002		
C_UKC1_6	4.4	0.707440	0.000180	0.1193	0.0000	0.0535	0.0002		
C_UKC1_7	4.2	0.707380	0.000140	0.1193	0.0000	0.0531	0.0002		
C_UKC1_8	4.1	0.707490	0.000150	0.1193	0.0000	0.0528	0.0002		
C_UKC1_9	4.1	0.707390	0.000150	0.1193	0.0000	0.0530	0.0002		
C_UKC1_10	4.0	0.707420	0.000150	0.1193	0.0000	0.0527	0.0003		
C_UKC1_11	4.0	0.707510	0.000160	0.1193	0.0000	0.0531	0.0003		
C_UKC1_12	3.8	0.707300	0.000170	0.1193	0.0000	0.0531	0.0003		
C_UKC1_1	4.1	0.707470	0.000160	0.1194	0.0000	0.0510	0.0002		
C_UKC1_2	4.2	0.707430	0.000150	0.1194	0.0000	0.0509	0.0002		
C_UKC1_3	4.2	0.707350	0.000140	0.1194	0.0000	0.0505	0.0002		
C_UKC1_4	3.9	0.707630	0.000160	0.1194	0.0000	0.0482	0.0003		
C_UKC1_5	3.6	0.707310	0.000160	0.1194	0.0000	0.0486	0.0003		
C_UKC1_6	3.8	0.707760	0.000140	0.1194	0.0000	0.0485	0.0003		
C_UKC1_7	3.4	0.707560	0.000160	0.1194	0.0000	0.0482	0.0003		
C_UKC1_8	3.5	0.707440	0.000150	0.1194	0.0000	0.0475	0.0003		
C_UKC1_9	3.6	0.707690	0.000170	0.1194	0.0000	0.0481	0.0003		
C_UKC1_10	3.1	0.707700	0.000190	0.1194	0.0000	0.0481	0.0003		
C_UKC1_11	3.2	0.707440	0.000210	0.1194	0.0000	0.0470	0.0003		
C_UKC1_12	3.5	0.707610	0.000180	0.1194	0.0000	0.0466	0.0003		
C_UKC1_13	3.4	0.707580	0.000170	0.1194	0.0000	0.0462	0.0003		
C_UKC1_14	3.4	0.707660	0.000160	0.1194	0.0000	0.0457	0.0003		
C_UKC1_1	3.7	0.707550	0.000170	0.1193	0.0000	0.0576	0.0003		
C_UKC1_2	3.7	0.707390	0.000160	0.1193	0.0000	0.0579	0.0002		
C_UKC1_3	3.6	0.707450	0.000170	0.1193	0.0000	0.0580	0.0002		
C_UKC1_4	3.6	0.707420	0.000160	0.1193	0.0000	0.0579	0.0002		
C_UKC1_5	3.5	0.707700	0.000160	0.1193	0.0000	0.0577	0.0003		
C_UKC1_6	3.4	0.707660	0.000180	0.1193	0.0000	0.0579	0.0002		
C_UKC1_7	3.3	0.707420	0.000180	0.1193	0.0000	0.0577	0.0003		
C_UKC1_8	3.5	0.707270	0.000170	0.1193	0.0000	0.0577	0.0003		
C_UKC1_9	3.3	0.707310	0.000200	0.1194	0.0000	0.0580	0.0003		
C_UKC1_10	3.2	0.707460	0.000220	0.1193	0.0000	0.0574	0.0003		
C_UKC1_11	3.1	0.707450	0.000210	0.1193	0.0000	0.0574	0.0003		
C_UKC1_12	3.4	0.707580	0.000200	0.1193	0.0000	0.0578	0.0002		
C_UKC1_13	3.3	0.707400	0.000190	0.1193	0.0000	0.0577	0.0003		
DGC_1	1.4	0.710260	0.000440	0.1192	0.0000	0.0584	0.0007		
DGC_2	1.4	0.710920	0.000400	0.1192	0.0000	0.0581	0.0006		
DGC_3	1.4	0.710590	0.000440	0.1192	0.0000	0.0582	0.0007		
DGC_4	1.4	0.710260	0.000490	0.1192	0.0000	0.0580	0.0007		
DGC_5	1.8	0.709960	0.000380	0.1193	0.0000	0.0559	0.0005		

Reference	Sr Beam Int.	87Sr/86Sr ratio	87Sr/86Sr 2SE	86Sr/88Sr	86Sr/88Sr 2SE	84Sr/86Sr	84Sr/86Sr 2SE	Sr_ppm	Rb_ppm
DGC_6	1.4	0.710350	0.000410	0.1192	0.0000	0.0575	0.0007		
DGC_7	1.4	0.710290	0.000400	0.1192	0.0000	0.0573	0.0008		
DGC_8	1.3	0.710510	0.000460	0.1192	0.0000	0.0570	0.0007		
DGC_9	1.4	0.710610	0.000400	0.1193	0.0000	0.0574	0.0007		
DGC_10	1.3	0.710290	0.000390	0.1192	0.0000	0.0573	0.0006		
DGC_11	1.3	0.710490	0.000470	0.1192	0.0000	0.0576	0.0008		
DGC_12	1.2	0.710480	0.000410	0.1192	0.0000	0.0573	0.0008		
DGC_1	1.3	0.710500	0.000410	0.1194	0.0000	0.0552	0.0007		
DGC_2	1.4	0.711400	0.000410	0.1194	0.0000	0.0541	0.0008		
DGC_3	1.3	0.711300	0.000440	0.1194	0.0000	0.0542	0.0007		
DGC_4	1.2	0.710540	0.000440	0.1194	0.0000	0.0516	0.0009		
DGC_5	1.1	0.710060	0.000420	0.1194	0.0000	0.0521	0.0009		
DGC_6	1.2	0.710200	0.000500	0.1194	0.0000	0.0526	0.0008		
DGC_7	1.1	0.710030	0.000530	0.1194	0.0000	0.0520	0.0010		
DGC_8	1.1	0.709940	0.000500	0.1194	0.0000	0.0507	0.0010		
DGC_9	1.0	0.709870	0.000480	0.1194	0.0000	0.0514	0.0010		
DGC_10	1.0	0.709940	0.000650	0.1194	0.0000	0.0507	0.0010		
DGC_11	0.9	0.709980	0.000610	0.1194	0.0000	0.0493	0.0013		
DGC_12	1.0	0.710080	0.000570	0.1194	0.0000	0.0494	0.0012		
DGC_13	0.9	0.710960	0.000620	0.1194	0.0000	0.0489	0.0012		
DGC_14	0.9	0.710330	0.000670	0.1194	0.0000	0.0473	0.0011		
DGC_1	1.2	0.710240	0.000480	0.1193	0.0000	0.0617	0.0008		
DGC_2	1.1	0.709950	0.000510	0.1193	0.0000	0.0612	0.0008		
DGC_3	1.1	0.710160	0.000470	0.1192	0.0000	0.0604	0.0008		
DGC_4	1.2	0.710410	0.000460	0.1193	0.0000	0.0610	0.0008		
DGC_5	1.1	0.710330	0.000470	0.1192	0.0000	0.0608	0.0008		
DGC_6	1.1	0.710390	0.000510	0.1192	0.0000	0.0605	0.0010		
DGC_7	1.1	0.710500	0.000490	0.1193	0.0000	0.0607	0.0009		
DGC_8	1.0	0.709910	0.000550	0.1193	0.0000	0.0618	0.0008		
DGC_9	1.1	0.710200	0.000510	0.1193	0.0000	0.0611	0.0008		
DGC_10	1.1	0.710370	0.000500	0.1193	0.0000	0.0614	0.0009		
DGC_11	1.1	0.709980	0.000540	0.1193	0.0000	0.0613	0.0008		
DGC_12	1.1	0.710330	0.000500	0.1192	0.0000	0.0605	0.0008		
DGC_13	1.1	0.710330	0.000590	0.1192	0.0000	0.0609	0.0009		
DGC_14	1.1	0.710320	0.000520	0.1193	0.0000	0.0616	0.0008		
G_NIST610_1	2.3	1.247700	0.003600	0.1085	0.0001	-0.2210	0.0027		
G_NIST610_2	2.5	1.258300	0.003600	0.1089	0.0001	-0.2117	0.0026		
G_NIST610_3	2.6	1.272700	0.003800	0.1089	0.0001	-0.2099	0.0034		
G_NIST610_4	2.5	1.271500	0.003600	0.1093	0.0001	-0.1997	0.0030		
G_NIST610_5	2.7	1.284200	0.003100	0.1094	0.0001	-0.1975	0.0026		
G_NIST610_6	2.7	1.299700	0.003100	0.1096	0.0001	-0.1929	0.0032		
G_NIST610_7	2.7	1.310900	0.002300	0.1097	0.0001	-0.1916	0.0033		
G_NIST610_8	2.4	1.344900	0.004800	0.1094	0.0001	-0.1999	0.0029		
G_NIST610_9	2.5	1.341600	0.004000	0.1096	0.0001	-0.1948	0.0023		
G_NIST610_10	2.3	1.338200	0.006000	0.1097	0.0001	-0.1910	0.0024		
G_NIST610_11	2.3	1.325600	0.005900	0.1101	0.0001	-0.1802	0.0026		
G_NIST610_12	2.1	1.322100	0.005800	0.1101	0.0001	-0.1792	0.0023		
G_NIST610_1	1.8	0.939800	0.003500	0.1175	0.0001	-0.2675	0.0054		
G_NIST610_2	2.1	0.958800	0.003100	0.1178	0.0000	-0.2355	0.0042		
G_NIST610_3	2.0	0.973800	0.002200	0.1178	0.0000	-0.2391	0.0044		
G_NIST610_4	1.9	1.078000	0.003200	0.1180	0.0000	-0.2222	0.0044		
G_NIST610_5	1.8	1.097100	0.003000	0.1180	0.0000	-0.2214	0.0041		
G_NIST610_6	2.0	1.086300	0.003100	0.1181	0.0000	-0.2148	0.0037		
G_NIST610_7	2.1	1.106200	0.002100	0.1180	0.0000	-0.2247	0.0043		
G_NIST610_8	2.2	1.120600	0.002500	0.1180	0.0000	-0.2250	0.0040		
G_NIST610_9	2.0	1.143500	0.005200	0.1181	0.0000	-0.2116	0.0052		
G_NIST610_10	1.9	1.172100	0.005700	0.1181	0.0000	-0.2095	0.0052		
G_NIST610_11	2.0	1.186900	0.005300	0.1181	0.0000	-0.2083	0.0050		
G_NIST610_12	1.9	1.230900	0.005500	0.1181	0.0000	-0.2082	0.0051		
G_NIST610_13	1.9	1.268900	0.006500	0.1181	0.0000	-0.2167	0.0051		
G_NIST610_14	1.9	1.299000	0.005200	0.1181	0.0000	-0.2187	0.0054		
G_NIST610_1	1.0	0.829700	0.001800	0.1140	0.0000	-0.0722	0.0013		
G_NIST610_2	1.2	0.836700	0.001700	0.1142	0.0000	-0.0672	0.0010		
G_NIST610_3	1.2	0.836500	0.001500	0.1143	0.0000	-0.0647	0.0011		
G_NIST610_4	1.1	0.833800	0.002000	0.1144	0.0000	-0.0625	0.0012		
G_NIST610_5	1.2	0.831400	0.001700	0.1146	0.0000	-0.0569	0.0013		
G_NIST610_6	1.4	0.831800	0.001500	0.1147	0.0000	-0.0542	0.0011		
G_NIST610_7	1.4	0.831100	0.001400	0.1148	0.0000	-0.0531	0.0011		
G_NIST610_8	1.2	0.833200	0.001600	0.1148	0.0000	-0.0517	0.0011		
G_NIST610_9	2.0	0.830530	0.000930	0.1153	0.0000	-0.0402	0.0012		
G_NIST610_10	2.0	0.831150	0.000930	0.1153	0.0000	-0.0396	0.0010		
G_NIST610_11	2.0	0.833570	0.000900	0.1153	0.0000	-0.0410	0.0010		
G_NIST610_12	2.0	0.830970	0.000820	0.1152	0.0000	-0.0422	0.0010		
G_NIST610_13	1.7	0.835300	0.001100	0.1151	0.0000	-0.0448	0.0011		
G_NIST610_14	1.6	0.832300	0.001200	0.1151	0.0000	-0.0464	0.0011		

Table S5-1. Zircon U-Pb isotope age data of the PBSC unit (SHRIMP and LA-MC-ICP-MS).

Reference	Sample name	Age	SE
Unpublished data (In this study)	IG28-3	450.3	3.9
	IG37-1	447.0	10.0
	IG25-1	441.5	5.6
	IG10-1	435.7	5.0
	IG38-1	434.4	8.0
	IG28-1	429.9	6.1
	IG15-1	428.0	24.0
	IG29-1	426.0	12.0
	NJ11-2	440.0	8.6
	NJ4-2	436.5	3.5
	NJ8-1	448.1	5.1
	NJ3-2	447.6	2.8
Kim et al. (2020)	SAMPLE1_12.1	454.8	7.6
	SAMPLE1_34.1	453.7	14.0
	SAMPLE1_37.1	449.7	19.8
	SAMPLE2_71.1	434.0	8.1
	SAMPLE2_93.1	424.7	11.3
Jang (2017)	HM-1084-57	455.6	8.2
	HM-1084-87	453.7	4.2
	HM-1084-67	453.0	4.8
	HM-1084-68	452.6	4.2
	HM-1084-66	451.9	5.0
	HM-1084-79	451.7	3.4
	HM-1084-63	451.3	4.7
	HM-1084-83	451.3	9.7
	HM-1084-70	450.4	6.0
	HM-1084-52	449.3	3.7
	HM-1084-95	448.4	5.8
	HM-1084-72	448.2	5.7
	HM-1084-53	447.6	5.6
	HM-1084-54	447.6	3.3
	HM-1084-100	447.1	5.2
	HM-1084-96	446.7	5.2
	HM-1084-93	445.3	4.0
	HM-1084-91	444.5	4.0

Table S5-2. LA-MC-ICP-MS zircon U-Pb isotope age data of PBSC unit from NJ and BD site.

	Non Corr. Ratio									Anderson Corr. Ratio									Non Corr. Age						Anderson Corr. Age										
	207_235	2SE	206_238	2SE	Error Correlation	238_206	2SE	207_206	2SE	207_235	2SE	206_238	2SE	Error Correlation	238_206	2SE	207_206	2SE	207_235	2SE	206_238	2SE	207_206	2SE	207_235	2SE	206_238	2SE	207_206	2SE	Approx_ U_PPM	Approx_ Th_PPM	Approx_ Pb_PPM	Th/U	FinalDisc Percent
IG24-1	10.49	0.22	0.46	0.01	0.95	2.18	0.04	0.17	0.00	10.46	0.30	0.45	0.01	0.85	2.20	0.05	0.16	0.00	2477.00	19.00	2430.00	39.00	2513.80	6.00	2472.00	29.00	2406.00	45.00	2472.00	34.00	29.74	21.90	29.02	0.71	3.40
NJ1-7	9.22	0.47	0.46	0.02	0.99	2.18	0.10	0.15	0.00	8.68	0.61	0.45	0.02	0.98	2.28	0.11	0.14	0.00	2323.00	42.00	2413.00	95.00	2296.00	11.00	2273.00	63.00	2390.00	99.00	2206.00	33.00	13.08	20.60	27.50	1.49	-3.70
NJ1-2	8.82	0.25	0.44	0.01	0.96	2.30	0.06	0.15	0.00	8.70	0.32	0.43	0.01	0.94	2.30	0.06	0.14	0.00	2320.00	25.00	2322.00	48.00	2300.20	7.10	2312.00	34.00	2314.00	51.00	2249.00	24.00	25.30	41.60	52.40	1.63	-1.50
NJ1-4	8.65	0.45	0.43	0.02	0.97	2.30	0.12	0.15	0.00	8.08	0.62	0.43	0.02	0.95	2.40	0.12	0.14	0.00	2301.00	47.00	2303.00	97.00	2305.00	14.00	2230.00	72.00	2280.00	100.00	2178.00	52.00	11.88	14.10	17.11	1.33	0.00
NJ1-3	8.78	0.90	0.43	0.04	0.99	2.31	0.23	0.15	0.00	8.40	1.00	0.43	0.04	0.99	2.43	0.19	0.14	0.00	2288.00	82.00	2290.00	180.00	2307.00	19.00	2250.00	100.00	2280.00	180.00	2272.00	43.00	12.45	15.10	20.03	1.22	2.90
NJ1-5	8.62	0.42	0.43	0.02	0.97	2.33	0.12	0.15	0.00	8.14	0.62	0.42	0.02	0.94	2.39	0.13	0.14	0.00	2289.00	46.00	2289.00	97.00	2296.00	13.00	2232.00	76.00	2260.00	100.00	2185.00	62.00	17.06	28.30	34.00	1.59	-0.30
NJ1-6	8.39	0.40	0.42	0.02	0.97	2.38	0.11	0.15	0.00	8.04	0.55	0.42	0.02	0.95	2.45	0.10	0.14	0.00	2262.00	41.00	2258.00	84.00	2288.00	13.00	2237.00	57.00	2242.00	88.00	2224.00	38.00	19.67	31.70	37.69	1.65	1.50
NJ1-1	7.98	0.21	0.40	0.01	0.95	2.51	0.06	0.15	0.00	7.60	0.32	0.40	0.01	0.94	2.53	0.07	0.14	0.00	2227.00	24.00	2166.00	46.00	2290.80	9.70	2186.00	38.00	2151.00	50.00	2176.00	37.00	22.66	29.80	33.80	1.27	5.20
IG6-3	5.92	0.08	0.37	0.01	0.91	2.68	0.04	0.12	0.00	5.93	0.08	0.37	0.01	0.91	2.68	0.04	0.12	0.00	1965.00	12.00	2046.00	25.00	1891.90	5.50	1965.00	12.00	2049.00	26.00	1888.20	6.30	72.00	19.10	21.86	0.24	-8.10
IG39-1	5.80	0.06	0.37	0.00	0.83	2.74	0.03	0.11	0.00	5.80	0.06	0.37	0.00	0.83	2.74	0.03	0.11	0.00	1944.70	8.90	2008.00	16.00	1879.60	7.20	1944.70	8.90	2008.00	16.00	1879.60	7.20	78.60	32.10	37.80	0.39	-6.80
IG6-2	5.76	0.09	0.36	0.01	0.94	2.76	0.04	0.12	0.00	5.77	0.10	0.36	0.01	0.91	2.77	0.04	0.12	0.00	1938.00	14.00	1994.00	28.00	1893.20	5.80	1940.00	15.00	1994.00	28.00	1885.40	7.90	62.00	19.10	20.20	0.29	-5.60
IG2-2	5.78	0.07	0.36	0.00	0.92	2.76	0.03	0.12	0.00	5.78	0.07	0.36	0.00	0.92	2.77	0.03	0.12	0.00	1942.00	11.00	1990.00	19.00	1889.30	4.30	1942.00	11.00	1990.00	19.00	1889.30	4.30	97.00	19.90	23.10	0.19	-5.60
IG3-3	5.66	0.11	0.35	0.01	0.90	2.82	0.04	0.12	0.00	5.67	0.11	0.35	0.01	0.90	2.81	0.04	0.12	0.00	1924.00	16.00	1957.00	26.00	1888.70	9.00	1925.00	16.00	1957.00	27.00	1887.80	8.90	51.00	20.90	21.03	0.39	-4.30
IG4-2	5.56	0.09	0.35	0.01	0.91	2.84	0.04	0.12	0.00	5.57	0.10	0.35	0.01	0.82	2.84	0.04	0.11	0.00	1911.00	12.00	1944.00	24.00	1883.20	8.60	1911.00	16.00	1945.00	24.00	1872.00	13.00	50.10	17.20	18.12	0.32	-3.40
IG9-1	5.42	0.07	0.34	0.00	0.89	2.93	0.03	0.12	0.00	5.37	0.09	0.34	0.00	0.88	2.94	0.03	0.11	0.00	1887.00	11.00	1892.00	18.00	1890.40	5.40	1880.00	15.00	1889.00	19.00	1865.00	11.00	72.70	14.00	13.83	0.17	-0.01
IG2-4	5.39	0.12	0.34	0.01	0.97	2.95	0.06	0.12	0.00	5.23	0.19	0.34	0.01	0.94	2.98	0.07	0.11	0.00	1881.00	19.00	1882.00	33.00	1892.10	4.70	1851.00	33.00	1876.00	36.00	1830.00	33.00	192.00	34.20	58.70	0.16	0.40
IG6-1	5.38	0.08	0.34	0.00	0.85	2.96	0.04	0.12	0.00	5.31	0.12	0.34	0.01	0.84	2.96	0.05	0.11	0.00	1882.00	12.00	1881.00	21.00	1887.40	7.60	1865.00	21.00	1878.00	25.00	1855.00	19.00	56.00	19.50	18.60	0.29	0.30
IG3-2	5.35	0.08	0.34	0.00	0.90	2.96	0.04	0.12	0.00	5.38	0.10	0.34	0.01	0.82	2.95	0.04	0.11	0.00	1882.00	13.00	1874.00	23.00	1889.90	6.50	1880.00	17.00	1876.00	24.00	1883.00	17.00	77.70	41.90	42.90	0.54	1.20
IG14-1	5.29	0.09	0.34	0.01	0.95	2.96	0.05	0.12	0.00	5.27	0.11	0.34	0.01	0.88	2.95	0.04	0.11	0.00	1870.00	14.00	1874.00	25.00	1878.00	4.40	1861.00	18.00	1878.00	26.00	1855.00	14.00	137.10	20.00	27.80	0.14	0.70
IG3-5	5.29	0.14	0.33	0.01	0.98	2.99	0.09	0.11	0.00	5.16	0.21	0.33	0.01	0.95	3.04	0.10	0.11	0.00	1867.00	24.00	1862.00	45.00	1873.30	5.60	1834.00	38.00	1844.00	50.00	1794.00	37.00	111.00	25.60	38.20	0.23	1.00
IG4-1	5.33	0.12	0.34	0.01	0.90	2.98	0.06	0.12	0.00	5.23	0.19	0.33	0.01	0.86	3.02	0.07	0.11	0.00	1874.00	18.00	1862.00	33.00	1894.40	7.70	1849.00	33.00	1853.00	36.00	1839.00	39.00	48.10	17.00	15.98	0.31	1.70
IG11-1	5.23	0.08	0.33	0.00	0.94	3.01	0.04	0.11	0.00	5.11	0.14	0.33	0.01	0.89	3.01	0.05	0.11	0.00	1859.00	14.00	1860.00	23.00	1869.10	5.60	1831.00	25.00	1850.00	26.00	1801.00	32.00	86.90	55.50	55.00	0.64	0.80
IG4-5	5.28	0.08	0.33	0.00	0.89	3.00	0.04	0.11	0.00	5.22	0.11	0.33	0.00	0.79	2.99	0.04	0.11	0.00	1865.00	13.00	1856.00	21.00	1876.80	8.10	1859.00	21.00	1858.00	21.00	1864.00	28.00	112.00	39.90	38.92	0.35	0.90
IG2-5	5.28	0.05	0.33	0.00	0.78	3.00	0.03	0.12	0.00	5.23	0.08	0.33	0.00	0.75	2.99	0.03	0.11	0.00	1864.40	8.30	1855.00	16.00	1889.30	7.50	1856.00	14.00	1860.00	17.00	1868.00	13.00	100.10	16.70	19.52	0.16	2.10
IG2-1	5.20	0.10	0.33	0.01	0.97	3.00	0.06	0.11	0.00	5.07	0.15	0.33	0.01	0.99	3.06	0.07	0.11	0.00	1847.00	18.00	1854.00	32.00	1859.00	5.10	1820.00	27.00	1848.00	33.00	1790.00	23.00	115.00	19.40	23.80	0.18	-0.30
IG4-3	5.27	0.09	0.33	0.01	0.86	3.00	0.06	0.12	0.00	5.24	0.23	0.33	0.01	0.66	2.98	0.07	0.11	0.00	1864.00	14.00	1853.00	32.00	1884.00	13.00	1869.00	47.00	1859.00	35.00	1880.00	80.00	82.70	24.70	24.58	0.30	0.60
IG4-4	5.21	0.08	0.33	0.01	0.86	3.02	0.05	0.11	0.00	5.22	0.15	0.33	0.01	0.77	3.02	0.05	0.11	0.00	1855.00	14.00	1843.00	27.00	1880.00	10.00	1852.00	25.00	1841.00	30.00	1859.00	29.00	71.30	25.50	24.13	0.36	1.80
IG3-1	5.20	0.09	0.33	0.01	0.90	3.02	0.05	0.12	0.00	5.08	0.20	0.33	0.01	0.83	3.06	0.06	0.11	0.00	1852.00	14.00	1840.00	28.00	1887.10	9.20	1827.00	34.00	1830.00	34.00	1812.00	48.00	80.40	42.70	41.30	0.53	2.10
IG2-3	5.04	0.14	0.33	0.01	0.99	3.04	0.09	0.11	0.00	4.86	0.21	0.33	0.01	0.99	3.10	0.11	0.11	0.00	1822.00	25.00	1830.00	47.00	1819.40	5.40	1792.00	37.00	1824.00	50.00	1755.00	28.00	206.00	25.60	44.40	0.11	-0.80
IG3-4	5.20	0.06	0.33	0.00	0.93	3.05	0.03	0.12	0.00	5.14	0.09	0.33	0.00	0.85	3.06	0.03	0.11	0.00	1852.50	9.60	1827.00	15.00	1887.50	4.40	1841.00	16.00	1826.00	16.00	1847.00	17.00	153.60	35.60	33.70	0.22	3.06
NJ10-1	5.12	0.05	0.33	0.00	0.97	3.05	0.03	0.11	0.00	5.07	0.08	0.33	0.00	0.81	3.07	0.03	0.11	0.00	1839.40	7.80	1827.00	14.00	1855.40	2.90	1828.00	16.00	1823.00	15.00	1834.00	21.00	316.70	52.50	45.20	0.17	1.63
IG21-1	5.61	0.24	0.33	0.01	0.98	3.06	0.12	0.12	0.00	5.02	0.34	0.32	0.01	0.98	3.21	0.16	0.11	0.00	1902.00	39.00	1822.00	63.00	2014.20	8.90	1798.00	63.00	1800.00	67.00	1785.00	61.00	70.10	38.30	57.60	0.48	9.60
IG18-1	4.32	0.19	0.28	0.01	0.97	3.60	0.14	0.11	0.00	3.67	0.28	0.27	0.01	0.94	3.82	0.18	0.10	0.00	1692.00	36.00	1582.00	57.00	1861.10	8.40	1520.00	64.00	1549.00	61.00	1542.00	70.00	82.30	24.70	19.50	0.29	15.10
NJ10-2	4.11	0.07	0.27	0.00	0.98	3.76	0.06	0.11	0.00	3.40	0.14	0.26	0.00	0.92	3.83	0.07	0.09	0.00	1659.00	14.00	1521.00	22.00	1832.10	4.60	1499.00	35.00	1503.00	25.00	1515.00	38.00	244.00	144.00	76.90	0.56	17.30
NJ5-1	3.27	0.09	0.22	0.01	0.96	4.46	0.12	0.11	0.00	2.52	0.11	0.22	0.01	0.92	4.63	0.13	0.08	0.00	1480.00	21.00	1303.00	31.00	1733.10	8.40	1274.00	33.00	1270.00	32.00	1281.00	35.00	86.00	45.90	15.21	0.41	25.20
NJ6-1	2.35	0.02	0.21	0.00	0.88	4.72	0.03	0.08	0.00	2.35	0.02	0.21	0.00	0.89	4.71	0.03	0.08	0.00	1226.70	5.00	1239.40	6.70	1200.20	4.00	1226.10	5.10	1240.20	6.80	1200.40	4.20	412.60	4.15	2.21	0.02	-3.18
NJ9-1	4.24	0.32	0.21	0.01	1.00	4.85	0.33	0.15	0.00	2.09	0.22	0.19	0.01	0.94	5.94	0.46	0.08	0.00	1646.00	67.00	1210.00	76.00	2324.00	19.00	1112.00	76.00	1097.00	72.00	1151.00	74.00	498.00	422.00	266.00	0.83	48.10
NJ6-2	2.19	0.03	0.20	0.00	0.91	5.00	0.04	0.08	0.00	2.19	0.03	0.20	0.00	0.91	5.00	0.04	0.08	0.00	1176.80	7.90	1175.20	9.10	1175.30	5.90	1176.80	7.90	1175.20	9.10	1175.30	5.90	630.00	8.90	4.87	0.01	-0.35
IG33-1	2.91	0.16	0.18	0.01	0.98	5.46	0.28	0.12	0.00	1.76	0.14	0.17	0.01	0.96	5.99	0.42	0.07	0.00	1380.00	42.00	1089.00	50.00	1884.00	13.00	1049.00	55.00	1034.00	52.00	1053.00	64.00	165.00	45.10	23.90	0.30	42.80
IG20-1	2.64	0.14	0.18	0.01	0.97	5.51	0.26	0.11	0.00	1.74	0.13	0.17	0.01	0.91	6.03	0.32	0.07	0.00	1305.00	41.00	1069.00	48.00	1738.00	13.00	1011.00	51.00	1029.00	47.00	1033.00	63.00	93.70	42.90	24.70	0.45	38.70
NJ13-1	1.79	0.02	0.17	0.00	0.97	5.94	0.07	0.08	0.00	1.68	0.03	0.17	0.00	0.96	5.98	0.07	0.07	0.00	1040.80	8.80	1002.00	10.00	1124.80	6.00	999.00	11.00	997.00	10.00	1005.00	12.00	576.50	33.30	9.82	0.06	10.92
IG19-1	0.79	0.03	0.09	0.00	0.58	10.70	0.22	0.06	0.00	0.74	0.02	0.09	0.00	0.85	10.72	0.23	0.06	0.00	592.00	14.00	576.00	11.00	681.00	36.00	560.00	13.00	576.00	12.00	539.00	23.00	37.50	20.10	7.32	0.51	8.80
IG19-3	0.74	0.05	0.09	0.00	0.48	11.04	0.44	0.06	0.00	0.69	0.05	0.09	0.00	0.80	11.22	0.46	0.06	0.00	559.00	27.00	559.00	21.00	605.00	64.00	532.00	28.00	555.00	22.00	512.00	32.00	37.20	25.30	7.52	0.65	3.00
IG35-2	0.67	0.02	0.09	0.00	0.71	11.53	0.28	0.06	0.00	0.65	0.02	0.09	0.00	0.99	11.30	0.21	0.06	0.00	528.70	7.30	535.00	13.00	470.00	15.00	519.50	9.70	535.00	13.00	410.00	26.00	179.70	256.00	82.70	1.40	-18.10
IG13-1	0.66	0.02	0.08	0.00	0.61	11.82	0.21	0.06	0.00	0.65	0.02	0.08	0.00	0.59	11.87	0.21	0.06	0.00	518.00	11.00	523.60	8.70	495.00	30.00	505.00	13.00	522.70	8.70	466.00	27.00	140.90	37.40	12.27	0.26	-7.10
IG23-2	0.68	0.02	0.08	0.00	0.55	11.89	0.20	0.06	0.00	0.63	0.02	0.08	0.00	0.75	11.97	0.21	0.06	0.00	523.00	10.00	520.50	8.40	526.00	25.00	493.00	13.00	517.20	8.80	441.00	24.00	60.30	28.70	8.75	0.47	-4.80
IG30-1	0.65	0.01	0.08	0.00	0.86	11.93	0.17	0.06	0.00	0.64	0.01	0.08	0.00	0.87	11.95	0.17	0.06	0.00	504.80	6.30	518.80	7.10	451.80	8.10	504.20	7.20	518.40	7.20	447.90	8.70	305.00	77.50	23.80	0.25	-14.80
IG34-1	0.66	0.03	0.08	0.00	0.71	12.02	0.27	0.06	0.00	0.60	0.02	0.08	0.00	0.81	12.02	0.25	0.05	0.00	517.00	17.00	515.00	11.00	519.00	48.00	477.00	15.00	512.00	12.00	315.00	49.00	43.90	26.00	8.01	0.44	-13.00
IG27-1	0.64	0.01	0.08	0.00	0.57	12.23	0.14	0.06	0.00	0.60	0.02	0.08	0.00	0.72	12.26	0.15	0.05	0.00	501.70	7.80	506.60	5.50	476.00	19.00	477.00	11.00	505.40	5.60	334.00	42.00	117.70	73.50	21.38	0.62	-9.30
IG7-2	0.63	0.02	0.08	0.00	0.89	12.30	0.24	0.06	0.00	0.61	0.02	0.08	0.00	0.92	12.44	0.26	0.06	0.00	495.00	10.00	503.80	9.40	464.00	18.00	480.00	13.00	502.60	9.60	433.00	20.00	135.10	67.50	20.90	0.49	-10.40
IG17-1	0.65	0.04	0.08	0.00	0.95	12.33	0.71	0.06	0.00	0.63	0.04	0.08	0.00	0.98	12.87	0.84	0.06	0.00	504.00	22.00	502.00	28.00	526.00	25.00	489.00	26.00	501.00	29.00	455.00	21.00	233.00	45.70	18.30	0.20	4.10
IG7-3	0.62	0.02	0.08	0.00	0.93	12.41	0.46	0.06	0.00	0.60	0.03	0.08	0.00	0.96	12.77	0.58	0.05	0.00	489.00	15.00	499.00	18.00	461.00	22.00	473.00	19.00	498.00	18.00	412.00	19.00	176.00	47.00	17.00	0.25	-11.00
IG8-3	0.63	0.01	0.08	0.00	0.73	12.45	0.09	0.06	0.00	0.60	0.01	0.08	0.00	0.77	12.49	0.10	0.05	0.00	496.50	4.70	498.10	3.60	499.00	11.00	475.80	7.50	496.90	3.70	371.00	34.00	246.00	162.00	48.40	0.65	-1.40
IG22-3	0.63	0.09	0.08	0.01	0.95	12.53	1.11	0.06	0.00	0.59	0.08	0.08	0.01	0.97	13.40	1.00	0.05	0.00	481.00	51.00	493.00	42.00	550.00	56.00	457.00	47.00	491.00	42.00	329.00	60.00	10.10	7.00	1.94	1.18	-5.00
NJ14-1	0.63	0.03	0.08	0.00	0.87	12.69	0.58	0.06	0.00	0.59	0.04	0.08	0.00	0.93	12.89	0.62	0.05	0.00	494.00	21.00	491.00	21.00	523.00	26.00	465.00	24.00	486.00	22.00	358.00	51.00	68.60	45.80	8.02	0.63	6.00
IG31-1	0.62	0.01	0.08	0.00	0.69	12.65	0.15	0.06	0.00	0.58	0.02	0.08	0.00	0.77	12.70	0.15	0.05	0.00	486.20	6.70	490.50	5.60	476.00	14.00	462.60	9.60	488.80	5.80	348.00	38.00	147.90	136.30	37.00	0.92	-6.20
IG19-2	0.66	0.02	0.08	0.00	0.45	12.66	0.26	0.06	0.00	0.57	0.02	0.08	0.00	0.70	12.81	0.29	0.05	0.00	516.00	13.00	490.20	9.70	628.00	36.00	457.00	15.00	486.00	10.00	319.00	58.00	49.70	42.00	10.09	0.84	14.20
IG8-1	0.61	0.01	0.08	0.00	0.75	12.77	0.12	0.06	0.00	0.58	0.01	0.08	0.00	0.70	12.80	0.13	0.05	0.00	485.70	4.70	485.90	4.30	483.80	9.10	466.10	8.70	485.30	4.50	344.00	42.00	464.00	318.00	82.50	0.69	-1.40
IG23-1	0.61	0.02	0.08	0.00	0.62	12.79	0.18	0.06	0.00	0.56	0.02	0.08	0.00	0.73	12.92	0.19	0.05	0.00	483.00	11.00	485.40	6.80	474.00	28.00	448.00	12.00	481.60	6.80	280.00	44.00	82.90	47.00	13.69	0.57	-10.10
IG15-2	0.63	0.06	0.08	0.01	0.86	12.80	0.95	0.06	0.00	0.58	0.06	0.08	0.01	0.94	13.80	1.00	0.05	0.00	482.00	40.00	483.00	34.00	691.00	83.00	451.00	34.00	480.00	35.00	368.00	47.00	10.88	5.70	1.66	0.62	13.00
IG22-2	0.60	0.05	0.08	0.01	0.57	12.67	0.80	0.06	0.00	0.57	0.04	0.08	0.01	0.91	13.22	0.75	0.05	0.00	479.00	35.00	483.00	28.00	595.00	64.00	452.00	27.00	485.00	30.00	370.00	42.00	9.91	8.00	1.90	0.91	1.00
NJ4-1	0.61	0.01	0.08	0.00	0.46	12.86	0.09	0.06	0.00	0.56	0.01	0.08	0.00	0.59	12.94	0.10	0.05	0.00	482.50	5.70	482.80	3.40	485.00	18.00	447.50	7.40	480.10	3.50	283.00	36.00	127.30	62.20	15.31	0.48	-4.60
IG28-2	0.60	0.02	0.08	0.00	0.51	12.89	0.25	0.06	0.00	0.56	0.02	0.08	0.00	0.80	13.05	0.25	0.05	0.00	477.00	13.00	481.80	9.10	474.00	36.00	452.00	11.00	477.80	8.90	326.00	38.00	49.60	21.60	5.55	0.42	-8.00
NJ3-1	0.60	0.01	0.08	0.00	0.62	12.91	0.11	0.06	0.00	0.60	0.01	0.08	0.00	0.55	12.91	0.13	0.06	0.00	481.00	5.80	481.00	4.10	473.00	15.00	477.00	8.10	479.20	4.60	463.00	20.00	571.80	176.10	40.77	0.30	-1.90
IG22-1	0.65	0.09	0.08	0.01	0.70	12.87	1.24	0.06	0.01	0.60	0.09	0.08	0.01	0.86	13.30	1.30	0.05	0.00	488.00	53.00	480.00	44.00	710.00	100.00	459.00	50.00	468.00	41.00	367.00	58.00	9.01	6.40	1.70	0.66	2.00
IG10-2	0.60	0.02	0.08	0.00	0.56	12.99	0.19	0.06	0.00	0.55	0.02	0.08	0.00	0.63	13.09	0.18	0.05	0.00	474.40	9.90	478.40	6.40	466.00	31.00	443.00	12.00	475.60	6.50	282.00	57.00	107.90	83.60	22.07	0.77	-1.40
NJ12-1	0.60	0.00	0.08	0.00	0.77	13.02	0.06	0.06	0.00	0.58	0.01	0.08	0.00	0.66	13.07	0.06	0.06	0.00	475.60	2.20	476.90	2.00	467.70	6.30	468.00	4.80	475.60	2.10	453.20	8.90	1527.00	563.00	122.90	0.36	-1.70
IG8-2	0.59	0.03	0.08	0.00	0.98	13.16	0.55	0.06	0.00	0.57	0.03	0.08	0.00	0.98	13.64	0.57	0.05	0.00	471.00	15.00	472.00	19.00	464.00	11.00	450.00	20.00	470.00	19.00	333.00	38.00	234.00	131.00	44.80	0.57	-1.20
IG35-1	0.59	0.01	0.08	0.00	0.42	13.19	0.10	0.06	0.00	0.55	0.01	0.08	0.00	0.66	13.26	0.11	0.05	0.00	468.90	6.30	471.00	3.30	469.00	17.00	443.40	8.50	468.90	3.50	304.00	38.00	164.30	256.20	63.10	1.55	-3.00
IG26-1	0.56	0.02	0.08	0.00	0.69	13.29	0.17	0.05	0.01	0.54	0.02	0.07	0.00	0.91	13.64	0.48	0.05	0.00	460.80	8.80	467.60	5.70	461.00	13.00	438.00	11.00	464.30	4.80	279.00	55.00	416.00	131.90	33.20	0.32	-2.10
IG13-2	0.59	0.01	0.08	0.00	0.63	13.31	0.13	0.06	0.00	0.54	0.01	0.07	0.00	0.71	13.39	0.14	0.05	0.00	467.50	4.60	467.00	4.40	482.00	11.00	438.10	9.30	464.70	4.70	305.00	48.00	281.00	83.60	21.03	0.30	2.40
IG32-1	0.58	0.01	0.08	0.00	0.63	13.33	0.11	0.06	0.00	0.54	0.01	0.07	0.00	0.72	13.41	0.12	0.05	0.00	463.10	4.30	466.20	3.70	460.00	13.00	436.20	7.90	463.80	3.80	285.00	38.00	239.00	245.00	60.40	1.02	-2.80
IG35-3	0.58	0.01	0.07	0.00	0.39	13.36	0.17	0.06	0.00	0.54	0.01	0.07	0.00	0.68	13.44	0.18	0.05	0.00	462.70	7.20	466.10	5.90	492.00	27.00	435.80	8.60	463.60	6.10	295.00	40.00	61.30	29.00	7.19	0.46	1.20
IG5-1	0.58	0.01	0.07	0.00	0.88	13.34	0.15	0.06	0.01	0.54	0.01	0.07	0.00	0.89	13.47	0.16	0.05	0.00	449.00	15.00	465.80	5.10	469.00	12.00	436.60	7.60	463.80	5.20	288.00	34.00	272.00	70.70	17.27	0.26	0.80
IG7-1	0.58	0.01	0.07	0.00	0.86	13.35	0.18	0.06	0.00	0.56	0.02	0.07	0.00	0.81	13.43	0.19	0.05	0.00	466.90	6.10	465.50	5.90	482.00	12.00	450.00	14.00	463.70	6.30	344.00	69.00	476.90	161.60	37.22	0.34	3.20
IG8-4	0.58	0.01	0.07	0.00	0.73	13.47	0.15	0.06	0.00	0.53	0.01	0.07	0.00	0.76	13.51	0.15	0.05	0.00	461.40	5.80	461.70	5.10	464.00	12.00	433.10	7.80	459.80	5.10	279.00	33.00	230.60	275.60	66.60	1.20	0.60
IG36-1	0.57	0.00	0.07	0.00	0.59	13.47	0.07	0.06	0.00	0.53	0.01	0.07	0.00	0.69	13.53	0.08	0.05	0.00	459.90	3.10	461.50	2.40	469.10	7.70	431.20	7.40	459.70	2.50	284.00	40.00	421.90	263.30	61.85	0.62	0.80
IG12-1	0.57	0.01	0.07	0.00	0.54	13.48	0.17	0.06	0.00	0.53	0.01	0.07	0.00	0.65	13.54	0.18	0.05	0.00	459.00	7.50	461.10	5.60	476.00	21.00	431.40	9.40	458.80	5.90	296.00	40.00	98.80	28.10	7.23	0.29	0.20
IG5-2	0.58	0.01	0.07	0.00	0.69	13.52	0.17	0.06	0.00	0.52	0.01	0.07	0.00	0.64	13.60	0.18	0.05	0.00	460.70	6.60	460.00	5.70	474.00	19.00	424.90	8.20	455.90	6.00	273.00	42.00	200.00	42.70	10.37	0.21	1.10
IG28-3	0.56	0.01	0.07	0.00	0.96	13.82	0.12	0.06	0.00	0.51	0.02	0.07	0.00	0.86	14.11	0.40	0.05	0.00	448.90	5.80	450.30	3.90	462.80	9.70	420.60	9.80	447.90	4.00	258.00	54.00	499.00	134.40	31.36	0.27	2.00
NJ8-1	0.56	0.01	0.07	0.00	0.94	13.89	0.16	0.06	0.00	0.53	0.01	0.07	0.00	0.92	13.96	0.18	0.05	0.00	448.80	4.70	448.10	5.10	454.10	4.10	430.10	9.40	446.80	5.40	332.00	38.00	1841.00	783.00	157.10	0.44	1.40
NJ3-2	0.55	0.01	0.07	0.00	0.71	13.91	0.09	0.06	0.00	0.54	0.01	0.07	0.00	0.66	13.95	0.10	0.06	0.00	447.40	3.20	447.60	2.80	440.10	7.40	441.20	4.50	446.30	2.90	421.60	9.60	553.00	200.20	39.50	0.35	-0.90
IG37-1	0.58	0.02	0.07	0.00	0.71	13.91	0.33	0.06	0.00	0.52	0.02	0.07	0.00	0.88	14.09	0.34	0.05	0.00	469.00	13.00	447.00	10.00	577.00	35.00	423.00	13.00	445.00	10.00	314.00	39.00	39.86	19.50	4.57	0.46	15.00
IG25-1	0.55	0.01	0.07	0.00	0.48	14.10	0.18	0.06	0.00	0.50	0.01	0.07	0.00	0.65	14.20	0.18	0.05	0.00	445.90	8.70	441.50	5.60	476.00	27.00	409.70	9.80	439.40	5.60	228.00	42.00	68.70	55.00	12.18	0.80	4.30
NJ11-2	1.43	0.04	0.07	0.00	0.91	14.14	0.28	0.15	0.00	0.44	0.01	0.06	0.00	0.88	16.20	0.34	0.05	0.00	899.00	17.00	440.00	8.60	2299.00	20.00	365.00	10.00	387.70	7.40	217.00	42.00	700.00	609.00	182.80	0.86	80.85
NJ4-2	0.55	0.01	0.07	0.00	0.58	14.27	0.12	0.06	0.00	0.49	0.01	0.07	0.00	0.71	14.39	0.13	0.05	0.00	444.70	4.80	436.50	3.50	476.00	15.00	406.90	7.10	433.70	3.60	248.00	34.00	162.70	84.40	18.08	0.52	6.00

	207_235	2SE	206_238	2SE	Error Correlation	238_206	2SE	207_206	2SE	207_235	2SE	206_238	2SE	Error Correlation	238_206	2SE	207_206	2SE	207_235	2SE	206_238	2SE	207_206	2SE	207_235	2SE	206_238	2SE	207_206	2SE	Approx_ U_PPM	Approx_ Th_PPM	Approx_ Pb_PPM	Th/U	FinalDisc Percent
IG10-1	0.54	0.01	0.07	0.00	0.45	14.30	0.17	0.06	0.00	0.49	0.01	0.07	0.00	0.68	14.44	0.18	0.05	0.00	439.10	6.60	435.70	5.00	463.00	24.00	403.30	9.20	432.80	5.20	234.00	45.00	104.40	71.90	15.98	0.68	3.20
IG38-1	0.55	0.02	0.07	0.00	0.60	14.35	0.27	0.06	0.00	0.49	0.02	0.07	0.00	0.79	14.46	0.28	0.05	0.00	441.00	13.00	434.40	8.00	480.00	38.00	407.00	11.00	431.90	8.10	290.00	32.00	30.50	14.40	3.18	0.39	-12.00
IG28-1	0.54	0.02	0.07	0.00	0.47	14.49	0.21	0.06	0.00	0.47	0.01	0.07	0.00	0.58	14.64	0.23	0.05	0.00	436.00	12.00	429.90	6.10	488.00	35.00	391.90	9.70	423.90	6.30	220.00	47.00	58.20	25.90	5.65	0.43	4.60
IG15-1	0.53	0.04	0.07	0.00	0.80	14.53	0.87	0.06	0.00	0.51	0.04	0.07	0.00	0.87	14.80	0.89	0.05	0.00	429.00	27.00	428.00	24.00	527.00	60.00	405.00	27.00	426.00	25.00	247.00	44.00	17.68	16.00	3.86	0.81	-27.00
IG29-1	0.64	0.03	0.07	0.00	0.68	14.68	0.41	0.07	0.00	0.49	0.02	0.07	0.00	0.89	14.91	0.45	0.05	0.00	503.00	17.00	426.00	12.00	878.00	37.00	402.00	12.00	418.00	12.00	313.00	41.00	38.70	17.60	5.68	0.42	48.60
NJ11-1	1.01	0.02	0.06	0.00	0.86	16.76	0.28	0.12	0.00	0.38	0.01	0.05	0.00	0.84	18.54	0.30	0.05	0.00	710.00	11.00	373.60	5.90	2015.00	21.00	326.80	6.80	339.70	5.40	250.00	33.00	868.00	1040.00	187.00	1.14	81.39
IG1-1	0.43	0.07	0.06	0.00	0.61	16.92	1.17	0.05	0.01	0.43	0.04	0.06	0.00	0.85	18.00	1.40	0.05	0.00	352.00	44.00	369.00	25.00	646.00	83.00	354.00	29.00	368.00	25.00	268.00	36.00	9.53	5.40	1.06	0.73	19.00
IG16-1	0.48	0.03	0.06	0.00	0.96	17.04	1.07	0.06	0.00	0.41	0.03	0.06	0.00	0.97	18.10	1.20	0.05	0.00	400.00	21.00	367.00	22.00	593.00	26.00	344.00	24.00	363.00	22.00	199.00	51.00	220.00	63.60	18.50	0.30	35.00
IG1-5	0.40	0.01	0.05	0.00	0.29	18.42	0.21	0.05	0.00	0.36	0.01	0.05	0.00	0.67	18.56	0.22	0.05	0.00	342.10	6.30	340.80	3.80	385.00	26.00	315.40	5.40	338.50	3.90	162.00	27.00	72.50	38.80	7.42	0.54	-2.00
IG1-2	0.36	0.01	0.05	0.00	0.56	20.45	0.17	0.05	0.00	0.33	0.01	0.05	0.00	0.57	20.57	0.17	0.05	0.00	310.80	4.60	307.80	2.40	328.00	20.00	289.60	4.10	306.50	2.50	158.00	27.00	163.80	129.20	20.19	0.79	3.40
IG1-4	0.34	0.01	0.05	0.00	0.51	21.03	0.27	0.05	0.00	0.31	0.01	0.05	0.00	0.71	21.19	0.26	0.05	0.00	299.80	7.60	299.40	3.70	337.00	32.00	275.20	5.10	297.80	3.70	106.00	20.00	74.30	64.90	9.71	0.87	-36.00
IG1-3	0.34	0.01	0.05	0.00	0.47	21.12	0.26	0.05	0.00	0.31	0.01	0.05	0.00	0.66	21.26	0.27	0.05	0.00	297.60	7.80	298.60	3.50	326.00	34.00	270.10	4.80	296.80	3.60	94.00	21.00	64.20	45.40	6.38	0.70	-28.00
NJ2-1	0.13	0.01	0.02	0.00	0.38	51.81	1.15	0.05	0.00	0.12	0.00	0.02	0.00	0.81	51.90	1.10	0.05	0.00	125.70	7.70	123.20	2.70	254.00	56.00	118.40	2.40	123.40	2.60	3.60	3.30	150.60	179.00	12.28	1.18	18.00
NJ2-5	0.12	0.00	0.02	0.00	0.03	54.41	0.50	0.05	0.00	0.12	0.00	0.02	0.00	0.75	54.59	0.51	0.05	0.00	118.10	2.70	117.40	1.00	191.00	26.00	112.40	1.30	117.20	1.10	8.80	3.50	232.00	158.50	10.18	0.67	-82.00
NJ2-4	0.12	0.00	0.02	0.00	0.43	56.50	0.70	0.05	0.00	0.11	0.00	0.02	0.00	0.86	56.70	0.74	0.05	0.00	113.00	2.10	113.10	1.40	162.00	24.00	108.50	1.40	112.60	1.40	15.00	4.90	402.00	489.00	27.80	1.20	-100.00
NJ2-3	0.11	0.00	0.02	0.00	0.39	58.93	0.59	0.05	0.00	0.11	0.00	0.02	0.00	0.72	58.78	0.56	0.05	0.00	107.20	3.20	108.50	1.10	191.00	32.00	104.60	1.30	108.30	1.10	16.00	4.70	190.50	173.30	9.43	0.90	12.00
NJ2-2	0.11	0.00	0.02	0.00	0.36	63.41	0.60	0.05	0.00	0.10	0.00	0.02	0.00	0.83	63.63	0.61	0.05	0.00	101.80	3.00	100.86	0.96	253.00	36.00	97.51	0.97	100.38	0.94	11.20	4.70	245.40	278.80	13.66	1.12	19.00

Table S5-3. SHRIMP zircon U-Pb isotope age data of PBSC unit from Pyeongan-ri and Haengmae-dong (Kim et al., 2020).

Spot	204Pb /206Pb	±%	207Pb /206Pb	±%	208Pb /206Pb	±%	206Pb /238U	±%	% 206Pb$_c$	ppm U	ppm Th	4-corr ppm 206Pb*	4-corr ppm 208Pb*	232Th /238U	±%	(1) 206Pb /238U Age		(2) 206Pb /238U Age		(3) 206Pb /238U Age		(1) 207Pb /206Pb Age		preferred age		(1) 208Pb /232Th Age	
SAMPLE1_1.1	0.00	21.30	0.12	1.48	0.19	1.16	0.86	3.25	0.56	211.21	131.00	60.16	10.81	0.64	0.60	1846.09	28.16	1846.43	31.95	1851.35	30.66	1843.43	32.77	1843.43	32.77	1780.18	45.40
SAMPLE3_60.1	0.00	24.26	0.28	2.16	0.01	5.93	1.73	1.56	0.21	70.38	0.64	40.23	0.03	0.01	3.91	3288.45	74.33	3209.39	138.17	3290.03	74.52	3368.94	34.11	3368.94	34.11	1193.72	1869.13
SAMPLE1_72.1	0.00	22.36	0.21	1.17	0.16	1.28	0.54	3.11	0.28	303.69	128.79	62.78	9.46	0.44	1.03	1390.11	17.80	1230.78	17.01	1379.83	18.85	2901.24	19.66	2901.24	19.66	1591.93	37.08
SAMPLE1_89.1	0.00	41.21	0.19	0.77	0.14	1.61	1.25	1.42	0.12	75.44	36.33	35.29	4.86	0.50	0.71	2802.63	38.82	2819.91	57.20	2802.16	40.95	2763.59	13.35	2763.59	13.35	2811.98	68.54
SAMPLE1_49.1	0.00	19.70	0.20	0.92	0.34	1.30	1.32	2.28	0.75	48.96	57.62	21.41	6.98	1.22	0.71	2652.68	45.44	2616.77	60.14	2664.81	52.46	2751.32	19.54	2751.32	19.54	2562.68	67.33
SAMPLE3_61.1	0.00	19.78	0.19	1.08	0.17	1.00	1.29	3.40	0.22	151.53	87.84	70.51	11.49	0.60	0.56	2790.71	38.44	2812.75	56.84	2793.00	41.00	2740.01	18.38	2740.01	18.38	2753.41	55.52
SAMPLE2_78.1	0.00	26.11	0.19	0.58	0.10	1.40	1.25	1.91	0.16	119.98	38.79	49.31	4.61	0.33	1.11	2520.29	31.54	2466.51	39.51	2520.42	32.73	2694.08	10.26	2694.08	10.26	2516.45	61.34
SAMPLE2_11.1	0.00	25.09	0.18	0.56	0.20	0.96	1.27	2.30	0.16	170.99	118.49	76.12	14.93	0.72	0.55	2691.61	37.52	2696.47	52.07	2694.04	40.58	2678.77	9.92	2678.77	9.92	2659.40	52.12
SAMPLE2_70.1	0.00	40.83	0.17	0.44	0.08	1.10	1.16	1.92	0.04	220.33	64.12	96.43	7.99	0.30	0.50	2654.49	30.23	2675.42	41.94	2655.18	31.20	2596.04	7.54	2596.04	7.54	2631.98	46.66
SAMPLE1_4.1	0.00	80.18	0.17	2.35	0.13	1.32	1.11	2.55	0.02	172.72	79.40	67.79	9.19	0.47	0.67	2426.02	38.50	2384.67	48.61	2424.57	40.58	2577.28	39.41	2577.28	39.41	2454.57	56.10
SAMPLE1_13.1	0.00	50.71	0.17	0.97	0.11	1.28	1.12	2.70	0.04	200.01	70.57	81.34	8.56	0.36	0.64	2498.51	66.46	2479.61	84.82	2495.91	69.14	2562.39	16.39	2562.39	16.39	2566.05	85.39
SAMPLE1_60.1	0.00	37.50	0.17	0.89	0.27	1.32	1.17	1.47	0.18	74.94	72.99	32.44	8.60	1.01	0.69	2630.50	42.38	2654.33	58.50	2645.32	47.51	2561.67	16.19	2561.67	16.19	2497.63	59.61
SAMPLE2_63.1	0.00	31.62	0.17	0.68	0.11	1.49	1.26	0.67	0.14	116.74	43.11	49.74	5.19	0.38	0.68	2596.75	56.38	2611.64	76.18	2598.64	58.76	2551.83	12.11	2551.83	12.11	2549.34	78.52
SAMPLE2_28.1	0.00	22.36	0.17	0.61	0.17	1.09	1.18	2.83	0.22	158.40	94.45	64.94	11.03	0.62	0.58	2515.43	35.84	2506.01	46.32	2518.03	38.44	2546.71	11.32	2546.71	11.32	2476.33	52.09
SAMPLE2_92.1	0.00	24.83	0.17	0.54	0.19	0.90	0.98	2.25	-0.14	212.65	150.56	78.42	15.61	0.73	0.46	2302.49	27.78	2247.19	32.85	2309.84	30.12	2535.08	9.56	2535.08	9.56	2212.92	37.92
SAMPLE2_94.1	0.00	45.23	0.17	0.68	0.22	1.09	1.14	0.65	0.07	114.31	85.91	44.54	9.66	0.78	0.52	2411.47	31.22	2378.93	38.57	2413.42	34.16	2533.29	11.81	2533.29	11.81	2388.91	45.10
SAMPLE1_21.1	0.00	57.74	0.17	0.74	0.11	1.60	0.55	1.92	-0.05	464.99	158.94	219.25	24.97	0.35	1.69	2820.67	65.67	2950.69	109.13	2804.46	68.29	2532.56	12.69	2532.56	12.69	3265.52	112.86
SAMPLE3_89.1	0.00	33.13	0.16	1.18	0.20	3.65	1.12	1.08	-0.40	40.39	29.01	16.52	3.48	0.74	0.80	2510.05	42.20	2504.50	54.70	2507.67	46.07	2528.64	22.60	2528.64	22.60	2539.17	107.74
SAMPLE2_43.1	0.00	19.25	0.17	0.75	0.16	1.40	1.21	1.70	0.48	95.26	50.32	39.84	5.80	0.55	0.68	2556.99	38.69	2566.73	51.42	2563.37	41.20	2526.08	15.34	2526.08	15.34	2447.68	65.74
SAMPLE2_31.1	0.00	22.36	0.17	1.04	0.15	1.28	1.00	2.59	0.22	189.66	98.53	64.73	9.40	0.54	0.57	2156.75	30.91	2083.21	35.14	2163.41	32.90	2517.19	18.37	2517.19	18.37	2045.68	45.63
SAMPLE3_62.1	0.00	25.00	0.17	0.61	0.20	1.02	0.99	3.08	0.18	165.32	104.68	54.71	10.61	0.65	0.55	2100.70	30.26	2020.47	33.74	2095.73	32.75	2516.76	11.13	2516.76	11.13	2167.00	43.59
SAMPLE3_51.1	0.00	37.80	0.17	0.66	0.22	1.97	1.01	2.75	0.09	122.37	107.56	39.82	8.77	0.91	2.23	2070.87	135.59	1987.46	149.75	2103.80	150.18	2516.48	11.68	2516.48	11.68	1760.67	138.61
SAMPLE1_66.1	0.00	70.71	0.17	1.18	0.29	1.67	1.12	2.42	-0.09	37.80	36.11	14.96	4.35	0.99	0.76	2442.91	41.59	2422.74	52.39	2430.55	46.90	2516.08	20.53	2516.08	20.53	2552.25	67.05
SAMPLE1_8.1	0.00	100.00	0.17	0.98	0.17	1.77	1.08	0.91	0.03	70.55	39.12	27.62	4.59	0.57	0.80	2420.75	40.45	2395.24	50.40	2416.56	43.22	2515.58	16.66	2515.58	16.66	2487.64	66.13
SAMPLE1_36.1	0.00	30.00	0.17	0.76	0.26	1.13	0.97	3.09	0.20	105.82	96.48	39.00	9.91	0.94	0.61	2301.23	34.86	2251.07	41.38	2312.86	38.97	2513.36	13.92	2513.36	13.92	2193.91	47.42
SAMPLE3_74.1	0.00	106.07	0.17	0.73	0.20	1.23	1.12	0.69	0.02	106.43	71.87	44.08	8.72	0.70	0.57	2536.82	33.36	2545.41	44.09	2534.59	36.12	2508.82	12.41	2508.82	12.41	2565.98	50.53
SAMPLE1_94.1	0.00	61.24	0.17	0.59	0.13	1.16	0.88	3.49	0.03	222.20	96.15	90.50	11.77	0.45	0.53	2501.60	30.31	2499.52	39.27	2497.43	31.87	2508.64	9.98	2508.64	9.98	2588.52	47.81
SAMPLE1_46.1	0.00	41.60	0.17	0.63	0.12	1.30	1.14	2.82	0.07	156.73	62.98	63.83	7.45	0.42	0.64	2501.49	35.97	2499.65	46.58	2501.32	37.66	2507.73	10.91	2507.73	10.91	2505.24	55.07
SAMPLE3_99.1	0.00	44.72	0.17	1.38	0.10	1.43	0.97	4.49	0.06	156.61	54.89	62.92	6.25	0.36	0.59	2473.40	73.06	2463.79	93.33	2475.52	75.97	2507.14	23.42	2507.14	23.42	2418.37	89.79
SAMPLE1_87.1	0.00	150.00	0.16	3.39	0.08	2.11	0.87	2.25	-0.01	103.18	29.41	40.84	3.42	0.29	0.77	2443.16	56.73	2426.01	73.03	2442.51	58.55	2505.47	56.95	2505.47	56.95	2463.99	83.65
SAMPLE3_88.1	0.00	21.43	0.17	0.89	0.02	7.07	0.39	2.87	0.11	954.14	123.35	145.08	2.37	0.13	12.80	1050.62	12.71	957.75	12.14	1060.49	12.96	2504.37	15.32	2504.37	15.32	428.93	64.17
SAMPLE3_70.1	0.00	28.22	0.17	0.57	0.17	1.02	0.99	1.71	0.12	167.38	95.16	64.97	10.65	0.59	0.48	2403.59	29.01	2377.23	35.90	2405.19	31.02	2503.65	10.13	2503.65	10.13	2378.81	42.73
SAMPLE3_98.1	0.00	65.47	0.16	0.53	0.15	0.90	1.01	0.52	-0.02	282.05	148.23	97.93	15.01	0.54	0.89	2188.32	26.02	2122.41	29.82	2189.81	27.69	2502.16	9.03	2502.16	9.03	2163.79	38.86
SAMPLE3_31.1	0.00	35.36	0.16	0.92	0.14	1.00	1.12	3.32	0.06	201.12	95.44	78.90	10.92	0.49	0.55	2424.96	33.58	2404.64	42.09	2424.72	35.47	2500.35	15.64	2500.35	15.64	2429.63	46.98
SAMPLE1_9.1	0.00	39.06	0.17	0.79	0.12	2.95	1.11	2.25	0.13	101.48	38.56	41.55	4.79	0.39	0.76	2512.74	38.97	2516.63	50.89	2508.08	40.84	2499.69	14.17	2499.69	14.17	2624.04	91.49
SAMPLE2_86.1	0.00	38.41	0.16	3.46	0.16	2.12	0.64	2.86	-0.10	294.86	147.14	67.71	10.93	0.52	1.28	1527.14	38.28	1423.18	39.29	1521.96	40.81	2487.19	58.07	2487.19	58.07	1610.38	58.67
SAMPLE1_32.1	0.00	22.43	0.17	1.12	0.25	2.83	1.02	2.18	0.82	56.54	49.17	22.71	5.30	0.90	0.78	2472.89	43.54	2469.15	55.79	2490.85	48.78	2486.06	26.20	2486.06	26.20	2294.88	88.88
SAMPLE2_26.1	0.00	22.94	0.16	0.65	0.26	0.97	1.20	1.69	0.25	131.65	113.32	52.53	13.27	0.89	0.56	2459.26	35.59	2452.12	45.34	2456.82	39.58	2484.80	12.37	2484.80	12.37	2483.51	50.02
SAMPLE2_25.1	0.00	21.83	0.17	0.88	0.15	1.65	1.21	0.85	0.52	73.61	36.95	30.91	4.37	0.52	0.76	2565.30	40.73	2592.01	55.04	2568.53	43.28	2480.85	18.67	2480.85	18.67	2507.15	77.66
SAMPLE2_68.1	0.00	300.00	0.16	0.60	0.16	1.82	1.11	0.57	0.00	141.89	76.76	59.37	9.47	0.56	0.52	2558.35	31.42	2582.81	42.32	2555.41	33.52	2480.37	10.17	2480.37	10.17	2607.20	58.96
SAMPLE3_6.1	0.00	21.32	0.17	0.76	0.28	1.09	1.16	2.66	0.39	95.22	89.41	40.78	11.04	0.97	0.60	2607.47	39.09	2649.85	54.31	2607.27	43.89	2480.28	15.18	2480.28	15.18	2609.33	56.97
SAMPLE2_60.1	0.00	25.01	0.17	1.15	0.20	1.90	1.12	2.12	0.67	44.73	29.04	18.33	3.40	0.67	0.85	2514.26	45.19	2525.81	59.36	2516.73	49.00	2475.41	25.62	2475.41	25.62	2480.72	87.06
SAMPLE1_22.1	0.00	18.58	0.17	1.74	0.14	2.02	1.02	2.83	0.95	56.49	25.16	21.62	2.52	0.46	0.84	2375.31	40.09	2352.04	49.73	2387.02	42.41	2466.98	35.45	2466.98	35.45	2140.34	96.40
SAMPLE2_98.1	0.00	27.16	0.16	0.72	0.16	1.46	1.08	0.66	0.22	114.11	61.41	42.02	6.41	0.56	0.57	2299.71	30.19	2260.68	36.11	2304.27	32.22	2466.47	13.50	2466.47	13.50	2225.77	50.69
SAMPLE2_44.1	0.00	18.84	0.16	0.54	0.20	1.53	1.12	0.59	0.29	232.65	153.11	97.08	18.53	0.68	0.51	2552.40	34.75	2582.24	46.85	2551.71	37.68	2456.26	10.60	2456.26	10.60	2561.61	58.03
SAMPLE2_57.1	0.00	22.95	0.16	0.90	0.35	1.17	1.16	0.95	0.49	67.33	80.97	27.44	9.45	1.24	0.63	2503.16	40.01	2525.39	52.82	2507.19	46.71	2426.93	19.14	2426.93	19.14	2475.62	57.47
SAMPLE3_96.1	0.00	122.47	0.16	0.55	0.21	0.88	0.51	3.62	-0.01	398.13	224.37	75.41	15.97	0.58	0.41	1284.52	15.77	1191.79	15.48	1265.30	17.08	2414.88	9.43	2414.88	9.43	1545.21	24.77
SAMPLE1_5.1	0.00	27.74	0.16	0.90	0.09	2.07	1.23	3.23	0.32	78.59	24.64	31.25	2.71	0.32	0.87	2452.19	58.03	2463.58	74.77	2456.13	60.24	2410.63	17.77	2410.63	17.77	2338.29	96.61
SAMPLE2_81.1	0.00	53.88	0.16	0.72	0.14	1.38	0.77	2.55	-0.06	142.49	72.59	40.97	5.77	0.53	0.59	1861.32	23.93	1780.14	25.65	1870.13	25.44	2408.20	12.57	2408.20	12.57	1717.69	35.72
SAMPLE3_43.1	0.00	35.36	0.15	0.55	0.19	0.98	0.75	1.54	0.07	269.55	189.96	74.81	14.43	0.73	0.48	1804.89	25.24	1723.66	26.78	1818.89	27.55	2386.55	9.61	2386.55	9.61	1645.09	31.02
SAMPLE2_40.1	0.00	24.58	0.16	1.59	0.26	1.86	1.06	3.11	0.28	120.71	106.10	45.84	11.71	0.91	0.59	2359.86	35.46	2353.36	44.43	2361.14	39.76	2386.28	28.46	2386.28	28.46	2347.70	60.44
SAMPLE1_97.1	0.00	41.60	0.15	0.51	0.17	3.23	0.98	4.40	-0.03	275.28	158.74	96.27	16.21	0.60	0.42	2201.77	108.24	2165.64	126.67	2203.17	115.89	2375.77	8.73	2375.77	8.73	2181.13	137.64
SAMPLE2_69.1	0.00	29.14	0.15	0.75	0.12	3.11	0.57	2.66	0.07	525.09	200.15	120.33	14.10	0.39	3.12	1524.47	17.91	1435.38	18.22	1524.20	19.04	2373.76	12.99	2373.76	12.99	1530.24	68.02
SAMPLE3_80.1	0.00	18.50	0.15	0.91	0.18	0.93	0.28	2.74	0.26	800.52	519.40	86.60	14.91	0.67	0.37	764.67	12.94	702.71	12.24	776.25	14.18	2297.99	16.77	2297.99	16.77	637.64	13.54
SAMPLE1_62.1	0.00	21.82	0.15	1.34	0.17	1.20	0.89	3.26	0.30	181.21	110.94	59.69	9.93	0.63	0.58	2092.46	48.76	2058.52	56.12	2104.78	52.58	2278.89	24.58	2278.89	24.58	1924.92	58.28
SAMPLE3_41.1	0.00	21.82	0.15	0.81	0.17	1.38	1.03	2.97	0.41	89.32	53.01	31.66	5.12	0.61	0.64	2227.18	34.10	2218.41	40.97	2238.18	36.70	2269.17	17.34	2269.17	17.34	2070.42	53.23
SAMPLE3_12.1	0.00	18.90	0.14	0.95	0.18	1.58	0.93	4.96	0.75	76.13	43.38	27.11	4.57	0.59	1.41	2235.64	51.14	2243.76	62.25	2234.76	55.22	2196.65	23.64	2196.65	23.64	2248.61	84.42
SAMPLE3_18.1	0.00	35.36	0.14	0.94	0.17	1.54	0.89	3.45	0.20	88.80	53.29	28.92	4.98	0.62	0.67	2072.03	32.71	2050.18	37.80	2076.83	35.30	2196.60	18.28	2196.60	18.28	2006.23	51.03
SAMPLE2_5.1	0.00	26.94	0.14	0.99	0.19	1.58	1.00	2.11	0.39	96.97	60.39	35.01	6.43	0.64	0.72	2261.85	36.54	2276.06	45.01	2261.28	39.59	2195.50	21.28	2195.50	21.28	2269.52	62.72
SAMPLE3_29.1	0.00	25.27	0.14	1.61	0.13	2.06	0.99	2.65	0.17	165.83	74.07	52.14	6.64	0.46	1.41	2010.60	28.97	1983.94	33.28	2015.15	30.70	2173.30	28.86	2173.30	28.86	1926.11	57.11
SAMPLE2_73.1	0.00	33.33	0.13	0.58	0.12	1.11	0.88	1.97	-0.08	214.54	82.89	73.19	9.07	0.40	0.56	2155.79	25.87	2153.30	30.78	2147.85	27.12	2168.86	10.56	2168.86	10.56	2329.24	43.08
SAMPLE2_15.1	0.00	36.38	0.13	0.55	0.14	0.97	0.42	2.82	-0.06	596.15	262.84	98.06	13.98	0.46	0.94	1129.49	21.81	1064.75	21.51	1127.36	23.14	2143.83	9.91	2143.83	9.91	1165.59	28.62
SAMPLE1_70.1	0.00	24.92	0.13	0.79	0.11	2.92	0.84	3.09	0.29	145.79	61.56	40.51	4.29	0.44	0.56	1806.55	51.11	1763.96	55.67	1821.71	53.90	2129.23	16.41	2129.23	16.41	1513.38	68.29
SAMPLE1_18.1	0.00	29.98	0.14	1.99	0.09	1.67	0.16	11.57	0.47	1987.70	435.70	363.61	30.21	0.23	2.17	1244.54	20.63	1180.37	20.84	1237.14	21.32	2126.60	39.83	2126.60	39.83	1506.48	75.47
SAMPLE1_75.1	0.00	80.18	0.13	0.54	0.14	2.10	0.84	0.50	-0.01	287.60	144.54	90.81	13.08	0.52	0.44	2017.95	40.92	2000.84	46.92	2022.43	43.54	2122.61	9.61	2122.61	9.61	1944.63	59.22
SAMPLE1_11.1	0.00	37.80	0.13	1.01	0.24	3.80	0.78	2.47	0.20	120.84	94.37	44.41	10.79	0.81	1.31	2296.18	36.76	2336.35	46.59	2283.57	41.62	2113.07	19.99	2113.07	19.99	2427.65	103.08
SAMPLE2_19.1	0.00	25.00	0.13	0.98	0.20	1.49	0.96	2.31	0.44	83.81	55.64	28.53	5.63	0.69	0.67	2151.88	34.14	2159.50	40.87	2150.87	37.27	2111.28	22.06	2111.28	22.06	2164.29	57.56
SAMPLE2_91.1	0.00	18.88	0.14	0.66	0.23	0.95	0.77	3.21	0.80	202.97	148.85	64.41	14.16	0.76	1.33	2026.59	26.40	2015.86	30.35	2025.33	29.24	2092.04	22.02	2092.04	22.02	2040.29	53.37
SAMPLE2_76.1	0.00	94.87	0.13	0.96	0.12	1.05	0.85	3.08	-0.01	226.57	94.15	67.50	8.46	0.43	0.47	1919.36	23.11	1895.34	26.06	1918.76	24.34	2083.21	16.84	2083.21	16.84	1931.09	33.43
SAMPLE1_2.1	0.00	18.68	0.13	1.38	0.17	2.80	0.93	1.65	0.37	214.33	124.61	66.13	11.00	0.60	0.59	1978.40	40.93	1963.44	46.81	1984.09	44.28	2074.65	26.37	2074.65	26.37	1899.37	70.03
SAMPLE3_81.1	0.00	33.13	0.13	0.66	0.14	1.15	0.90	0.60	0.11	215.15	104.10	71.13	9.89	0.50	0.50	2099.09	25.89	2103.75	30.64	2102.69	27.50	2072.65	12.44	2072.65	12.44	2037.50	38.90
SAMPLE1_55.1	0.00	35.36	0.13	0.68	0.13	1.21	0.88	2.08	0.10	196.41	84.49	59.75	7.83	0.44	0.58	1954.45	28.30	1937.85	32.15	1952.67	29.90	2064.15	12.72	2064.15	12.72	1988.34	42.32
SAMPLE1_30.1	0.00	28.22	0.13	0.87	0.18	1.38	0.88	0.81	0.26	140.60	87.42	47.26	8.32	0.64	0.64	2128.93	46.94	2142.64	56.17	2135.65	50.79	2053.18	18.18	2053.18	18.18	2040.74	61.31
SAMPLE2_88.1	0.00	22.94	0.13	0.63	0.17	1.02	0.88	2.61	0.21	200.82	121.55	59.84	9.92	0.63	0.46	1919.58	23.66	1902.18	26.72	1931.56	25.56	2039.36	12.83	2039.36	12.83	1761.38	32.59
SAMPLE3_47.1	0.00	33.34	0.13	1.42	0.13	2.70	0.88	4.08	0.53	38.74	14.30	11.81	1.42	0.38	1.02	1957.53	36.54	1946.16	41.69	1950.10	38.47	2032.90	34.21	2032.90	34.21	2122.27	99.90
SAMPLE2_12.1	0.00	36.38	0.13	1.26	0.07	1.69	0.88	2.23	0.10	175.75	43.10	54.36	3.87	0.25	0.68	1982.38	28.90	1976.56	33.37	1983.86	29.80	2020.10	22.92	2020.10	22.92	1931.85	51.06
SAMPLE1_65.1	0.00	27.71	0.13	2.68	0.11	5.66	0.80	0.69	0.67	163.65	57.45	51.99	5.02	0.36	2.80	2028.73	30.88	2031.21	36.66	2034.92	32.70	2013.37	55.25	2013.37	55.25	1881.95	142.50
SAMPLE3_53.1	0.00	27.74	0.13	1.43	0.23	2.04	0.18	11.21	0.78	365.11	150.95	80.70	17.47	0.43	2.16	1476.00	55.33	1427.12	57.49	1422.15	59.62	2011.46	38.50	2011.46	38.50	2455.21	130.42
SAMPLE3_3.1	0.00	35.36	0.13	1.88	1.17	1.43	0.93	1.49	0.82	22.08	87.94	6.98	8.20	4.11	0.77	2019.87	45.00	2023.69	52.34	2035.61	97.51	1995.99	51.60	1995.99	51.60	2001.50	59.11
SAMPLE2_16.1	0.00	20.71	0.13	1.06	0.13	1.91	0.94	1.82	0.73	79.14	35.13	26.97	3.14	0.46	0.77	2154.11	34.73	2186.81	42.34	2166.36	36.80	1975.91	28.09	1975.91	28.09	1924.02	75.51
SAMPLE2_3.1	0.00	28.87	0.12	0.67	0.08	1.51	0.89	3.20	0.14	251.79	67.13	80.34	6.03	0.28	0.64	2036.22	63.63	2046.09	74.61	2039.52	65.76	1975.23	13.15	1975.23	13.15	1932.44	77.11
SAMPLE3_79.1	0.00	90.45	0.12	0.53	0.08	1.16	0.62	4.01	0.01	476.51	146.82	128.89	10.74	0.32	0.45	1764.66	20.85	1739.23	22.88	1771.46	21.65	1972.65	9.47	1972.65	9.47	1585.63	28.15
SAMPLE3_78.1	0.00	212.13	0.12	1.47	0.17	2.31	0.31	5.27	-0.01	173.56	72.33	33.39	5.78	0.43	3.33	1302.70	87.47	1253.02	89.14	1279.04	92.97	1967.57	26.37	1967.57	26.37	1726.51	139.98
SAMPLE3_58.1	0.00	22.37	0.13	1.17	0.14	2.05	0.88	1.75	0.78	60.58	28.42	19.43	2.51	0.48	0.80	2045.18	34.59	2058.79	40.72	2053.68	36.84	1961.70	31.97	1961.70	31.97	1896.79	78.26
SAMPLE3_69.1	0.00	24.41	0.12	0.66	0.22	2.40	0.42	4.70	0.16	673.88	435.50	183.45	39.51	0.67	1.86	1774.69	93.75	1752.49	103.15	1759.98	102.56	1955.10	12.87	1955.10	12.87	1949.59	125.95
SAMPLE3_9.1	0.00	20.86	0.12	0.94	0.18	1.51	0.85	1.89	0.56	96.27	56.43	30.54	5.15	0.61	0.65	2026.00	31.52	2037.98	36.96	2030.84	34.11	1950.87	23.51	1950.87	23.51	1959.80	54.83
SAMPLE3_34.1	0.00	23.01	0.12	1.49	0.05	1.93	0.95	0.63	0.22	187.51	29.22	57.78	2.48	0.16	0.75	1976.20	28.54	1980.06	33.20	1978.93	29.12	1950.72	27.93	1950.72	27.93	1828.66	67.28
SAMPLE1_61.1	0.00	26.11	0.12	0.62	0.10	1.22	0.83	0.51	0.14	290.08	97.70	81.57	7.84	0.35	0.57	1825.61	26.18	1809.46	29.16	1829.46	27.32	1950.28	12.13	1950.28	12.13	1733.10	38.22
SAMPLE3_20.1	0.00	27.74	0.12	0.69	0.05	1.90	0.89	1.97	0.16	198.20	33.32	61.49	2.83	0.17	0.72	1987.62	28.53	1994.23	33.13	1990.73	29.14	1944.43	13.76	1944.43	13.76	1831.91	60.12
SAMPLE2_4.1	0.00	53.03	0.12	0.96	0.09	3.73	0.86	3.32	0.09	126.92	33.14	40.93	3.48	0.27	0.81	2054.67	32.18	2074.91	38.22	2048.84	33.36	1931.01	18.28	1931.01	18.28	2240.14	93.47
SAMPLE3_90.1	0.00	300.00	0.12	0.79	0.32	1.03	0.72	2.19	0.00	186.76	197.09	48.92	15.93	1.09	0.45	1715.84	22.03	1692.55	24.10	1711.40	25.66	1918.51	14.12	1918.51	14.12	1746.26	30.89
SAMPLE3_71.1	0.00	32.93	0.12	0.77	0.07	1.88	0.85	0.59	0.15	152.41	33.08	46.45	3.02	0.22	0.67	1957.29	25.69	1963.22	29.70	1957.23	26.42	1917.10	15.51	1917.10	15.51	1959.81	56.97
SAMPLE2_13.1	0.00	29.85	0.12	0.90	0.17	1.41	0.60	0.73	0.24	207.77	84.37	44.10	7.38	0.42	0.61	1423.33	21.74	1382.46	22.65	1398.56	23.14	1908.74	19.09	1908.74	19.09	1882.88	44.94
SAMPLE2_72.1	0.00	29.42	0.12	0.74	0.12	1.35	0.72	3.78	-0.16	163.53	64.94	46.29	5.73	0.41	0.52	1836.12	23.01	1826.89	25.82	1833.09	24.18	1907.21	14.60	1907.21	14.60	1897.05	40.19
SAMPLE2_74.1	0.00	51.45	0.12	0.96	0.13	1.66	0.63	3.37	-0.09	145.16	57.35	42.20	5.78	0.41	0.61	1879.30	25.00	1875.70	28.38	1865.62	26.35	1905.83	18.28	1905.83	18.28	2155.40	50.46
SAMPLE1_90.1	0.00	24.54	0.12	1.22	0.07	1.16	0.37	5.62	0.23	982.71	258.06	153.63	9.82	0.27	0.45	1077.88	45.03	1031.22	44.95	1086.13	46.67	1905.71	23.51	1905.71	23.51	840.92	42.09
SAMPLE2_75.1	0.00	43.30	0.12	0.56	0.10	1.09	0.75	2.29	-0.04	322.10	109.89	92.72	9.72	0.35	0.46	1863.02	22.12	1858.19	24.99	1861.30	23.09	1899.29	10.36	1899.29	10.36	1903.70	33.60
SAMPLE3_28.1	0.00	21.33	0.12	1.09	0.18	1.72	0.87	2.08	0.73	72.46	39.41	23.15	3.76	0.56	0.73	2038.26	33.33	2060.71	39.45	2037.78	35.94	1897.86	29.77	1897.86	29.77	2045.28	67.44
SAMPLE3_85.1	0.00	40.45	0.12	0.65	0.18	0.97	0.80	1.56	-0.07	278.72	174.23	83.49	15.48	0.65	0.44	1928.53	30.34	1933.06	34.84	1929.88	32.86	1896.67	12.08	1896.67	12.08	1911.52	38.96
SAMPLE2_10.1	0.00	27.74	0.12	0.78	0.09	1.60	0.82	1.74	0.21	171.92	51.84	49.75	4.54	0.31	0.64	1871.62	27.50	1868.47	31.15	1871.13	28.62	1895.11	16.32	1895.11	16.32	1884.76	50.72
SAMPLE2_67.1	0.00	31.80	0.12	0.65	0.07	1.56	0.58	2.91	0.11	329.13	73.67	70.64	4.67	0.23	0.52	1437.74	17.84	1398.80	18.64	1439.31	18.38	1894.52	12.78	1894.52	12.78	1382.75	32.96
SAMPLE2_77.1	0.00	21.11	0.12	0.54	0.15	0.89	0.51	1.64	0.17	444.80	203.09	75.64	11.08	0.47	0.95	1164.38	17.53	1118.89	17.68	1162.48	18.72	1892.53	10.98	1892.53	10.98	1194.95	25.15
SAMPLE1_85.1	0.00	33.75	0.11	0.66	0.12	1.18	0.78	2.24	-0.10	235.54	96.06	68.41	8.75	0.42	0.49	1877.60	23.03	1875.65	26.14	1873.58	24.25	1892.01	12.52	1892.01	12.52	1956.47	37.12
SAMPLE1_53.1	0.00	47.43	0.12	0.63	0.11	1.21	0.86	2.55	0.04	254.98	88.95	71.74	7.64	0.36	0.56	1826.42	26.15	1818.03	29.30	1825.37	27.34	1891.89	11.76	1891.89	11.76	1850.61	38.23
SAMPLE2_53.1	0.00	44.72	0.12	0.75	0.09	1.52	0.79	2.85	0.07	213.47	64.46	64.02	5.93	0.31	0.63	1930.39	28.04	1935.98	32.25	1928.68	29.15	1891.14	14.20	1891.14	14.20	1976.44	46.70
SAMPLE1_86.1	0.00	44.72	0.12	0.42	0.02	1.79	0.79	1.74	0.02	637.10	45.27	182.19	3.33	0.07	0.58	1852.49	21.79	1847.98	24.58	1854.67	21.98	1886.81	7.70	1886.81	7.70	1595.24	39.98
SAMPLE2_14.1	0.00	100.00	0.12	0.81	0.09	1.69	0.83	0.70	0.02	189.38	58.57	57.22	5.25	0.32	0.65	1943.03	28.55	1951.26	32.98	1943.67	29.69	1886.03	14.82	1886.03	14.82	1926.02	46.28
SAMPLE1_31.1	0.00	50.00	0.11	0.73	0.20	1.05	0.64	0.92	-0.05	407.89	248.70	126.88	25.45	0.63	0.57	1992.20	50.47	2008.45	59.03	1977.17	54.70	1885.27	13.47	1885.27	13.47	2186.17	66.13
SAMPLE1_67.1	0.00	212.13	0.12	0.63	0.11	1.18	0.75	1.70	0.00	259.67	97.14	74.05	8.46	0.39	0.49	1847.92	22.57	1843.31	25.46	1846.65	23.69	1883.22	11.37	1883.22	11.37	1875.31	34.00
SAMPLE2_79.1	0.00	23.57	0.12	0.52	0.07	1.25	0.77	2.00	0.12	368.52	79.94	105.20	6.62	0.22	0.49	1849.62	21.87	1845.34	24.66	1851.30	22.48	1882.26	10.33	1882.26	10.33	1785.97	37.45
SAMPLE3_91.1	0.00	106.07	0.12	0.80	0.03	1.82	0.77	1.27	0.01	461.95	41.33	128.72	3.42	0.09	0.60	1811.10	21.16	1802.37	23.70	1811.39	21.39	1880.51	14.42	1880.51	14.42	1783.65	40.77
SAMPLE1_69.1	0.00	25.09	0.12	0.61	0.06	1.60	0.76	1.66	0.15	271.27	50.41	78.26	4.10	0.19	0.57	1866.66	22.59	1864.84	25.59	1869.16	23.13	1880.35	12.22	1880.35	12.22	1755.74	46.25
SAMPLE3_19.1	0.00	37.80	0.12	0.68	0.07	1.58	0.82	3.23	0.08	227.04	55.65	64.40	4.46	0.25	0.63	1839.32	26.52	1834.06	29.85	1842.51	27.35	1879.95	13.04	1879.95	13.04	1732.98	42.89
SAMPLE1_54.1	0.00	25.07	0.12	0.77	0.11	2.80	0.77	3.59	0.46	179.94	63.19	52.42	5.07	0.36	0.63	1882.53	27.84	1882.95	31.61	1889.03	29.21	1879.42	21.53	1879.42	21.53	1732.48	71.93
SAMPLE2_23.1	0.00	30.15	0.12	0.70	0.18	1.06	0.81	1.86	0.13	239.43	144.36	71.19	12.82	0.62	0.53	1916.14	27.43	1921.39	31.46	1916.60	29.70	1878.67	13.86	1878.67	13.86	1910.20	38.23
SAMPLE3_27.1	0.00	31.62	0.12	0.71	0.15	1.16	0.88	2.48	0.13	175.03	85.50	47.24	6.89	0.50	0.55	1761.21	25.70	1747.25	28.43	1762.41	27.41	1878.14	13.96	1878.14	13.96	1741.88	36.64
SAMPLE3_22.1	0.00	70.71	0.12	1.01	0.15	1.64	0.76	3.28	0.05	98.00	48.10	27.00	4.12	0.51	0.67	1793.32	28.12	1782.91	31.36	1789.99	30.03	1877.73	18.87	1877.73	18.87	1846.55	45.49
SAMPLE2_100.1	0.00	40.45	0.12	0.56	0.03	2.04	0.77	1.12	0.05	315.29	29.76	89.34	2.41	0.10	1.26	1837.67	29.42	1832.55	33.09	1838.71	29.77	1877.32	10.54	1877.32	10.54	1746.62	57.93
SAMPLE1_57.1	0.00	37.80	0.12	0.52	0.07	1.15	0.79	1.47	0.04	462.26	111.31	133.07	9.57	0.25	0.56	1863.22	26.07	1861.37	29.50	1863.55	26.87	1877.20	9.65	1877.20	9.65	1851.92	37.54
SAMPLE1_51.1	0.00	28.87	0.12	0.79	0.11	1.46	0.86	2.26	0.19	148.58	58.46	41.11	4.59	0.41	0.62	1799.83	27.09	1790.50	30.23	1804.82	28.51	1874.98	16.34	1874.98	16.34	1698.62	42.48
SAMPLE3_82.1	0.00	48.04	0.11	0.60	0.07	1.35	0.82	1.52	0.04	292.13	75.63	85.54	6.40	0.27	0.51	1890.97	22.70	1893.26	25.90	1893.11	23.45	1874.16	11.23	1874.16	11.23	1823.31	36.45
SAMPLE2_83.1	0.00	31.11	0.11	0.72	0.16	1.13	0.78	1.94	-0.14	160.18	88.70	44.94	7.58	0.57	0.92	1821.89	30.58	1815.27	34.28	1820.49	32.83	1874.06	14.22	1874.06	14.22	1841.70	43.78
SAMPLE3_75.1	0.00	83.21	0.11	0.73	0.27	0.92	0.76	1.82	-0.02	201.36	176.28	57.06	15.50	0.90	0.44	1837.81	23.02	1833.13	25.93	1831.48	25.99	1874.02	13.28	1874.02	13.28	1891.91	32.00
SAMPLE3_32.1	0.00	35.36	0.12	1.20	0.41	1.30	0.72	4.39	0.30	73.37	100.03	20.23	8.18	1.41	0.62	1794.23	29.84	1784.57	33.29	1799.91	36.57	1872.58	26.61	1872.58	26.61	1765.16	41.59
SAMPLE1_45.1	0.00	134.16	0.11	0.86	0.19	1.30	0.80	2.21	0.01	140.83	88.30	41.87	7.86	0.65	0.61	1916.12	30.82	1922.25	35.40	1916.32	33.47	1872.33	15.71	1872.33	15.71	1913.67	42.99

Spot	(2) 208Pb /232Th Age		(3) 207Pb /206Pb Age		% Dis-cor-dant	7corr 208Pb* /232Th	±%	Total 238U /206Pb	±%	Total 207Pb /206Pb	±%	(1) 238U /206Pb*	±%	(1) 207Pb* /206Pb*	±%	(1) 207Pb* /235U	±%	(1) 206Pb* /238U	±%	err corr	(3) 238U /206Pb*	±%	(3) 207Pb* /206Pb*	±%	(3) 207Pb* /235U	±%	(3) 206Pb* /238U	±%	err. corr.
SAMPLE1_1.1	1784.83	79.45	1888.61	30.53	-0.17	0.09	4.65	3.00	1.75	0.12	1.48	3.02	1.75	0.11	1.81	5.15	2.52	0.33	1.75	0.70	3.01	1.90	0.12	1.70	5.30	2.30	0.33	1.90	0.69
SAMPLE3_60.1	#NUM!	#NUM!	3371.87	33.94	3.05	#######	#######	1.50	2.89	0.28	2.16	1.50	2.89	0.28	2.19	25.79	3.62	0.67	2.89	0.80	1.50	2.89	0.28	2.18	25.86	3.61	0.67	2.89	0.80
SAMPLE1_72.1	-1929.26	104.18	2843.01	21.06	57.66	-0.09	-5.15	4.14	1.42	0.21	1.17	4.16	1.42	0.21	1.21	6.95	1.87	0.24	1.42	0.76	4.19	1.52	0.20	1.29	6.65	1.89	0.24	1.52	0.73
SAMPLE1_89.1	3169.93	404.82	2762.02	15.26	-1.74	0.17	13.80	1.83	1.71	0.19	0.77	1.84	1.71	0.19	0.81	14.45	1.89	0.54	1.71	0.90	1.84	1.80	0.19	0.93	14.44	1.83	0.54	1.80	0.87
SAMPLE1_49.1	2258.33	152.10	2793.27	27.04	4.37	0.12	7.12	1.95	2.08	0.20	0.92	1.96	2.09	0.19	1.19	13.41	2.40	0.51	2.09	0.87	1.95	2.40	0.20	1.65	13.84	2.73	0.51	2.40	0.80
SAMPLE3_61.1	3131.96	343.56	2747.79	19.39	-2.28	0.17	11.84	1.84	1.70	0.19	1.08	1.85	1.70	0.19	1.12	14.17	2.03	0.54	1.70	0.84	1.84	1.81	0.19	1.18	14.26	1.96	0.54	1.81	0.81
SAMPLE2_78.1	844.06	295.92	2694.59	11.45	7.79	0.04	35.80	2.09	1.51	0.19	0.58	2.09	1.51	0.18	0.62	12.17	1.63	0.48	1.51	0.92	2.09	1.57	0.18	0.69	12.18	1.60	0.48	1.57	0.90
SAMPLE2_11.1	2728.53	222.72	2687.67	13.29	-0.59	0.14	8.73	1.93	1.70	0.18	0.56	1.93	1.70	0.18	0.60	13.06	1.81	0.52	1.70	0.94	1.93	1.84	0.18	0.80	13.15	1.73	0.52	1.84	0.90
SAMPLE2_70.1	3321.65	391.88	2598.76	8.58	-2.75	0.18	12.79	1.96	1.39	0.17	0.44	1.96	1.39	0.17	0.45	12.22	1.46	0.51	1.39	0.95	1.96	1.43	0.17	0.51	12.24	1.43	0.51	1.43	0.94
SAMPLE1_4.1	1587.84	318.41	2571.02	40.10	7.04	0.08	20.85	2.19	1.90	0.17	2.35	2.19	1.90	0.17	2.36	10.84	3.03	0.46	1.90	0.63	2.19	2.01	0.17	2.40	10.79	3.00	0.46	2.01	0.60
SAMPLE1_13.1	2051.53	554.56	2551.35	18.62	3.01	0.11	28.43	2.11	3.21	0.17	0.97	2.11	3.21	0.17	0.98	11.13	3.35	0.47	3.21	0.96	2.11	3.34	0.17	1.11	11.04	3.26	0.47	3.34	0.94
SAMPLE1_60.1	2734.75	179.62	2620.19	22.64	-3.27	0.14	7.02	1.98	1.96	0.17	0.89	1.98	1.96	0.17	0.97	11.84	2.19	0.50	1.96	0.90	1.97	2.19	0.18	1.36	12.35	2.26	0.51	2.19	0.81
SAMPLE2_63.1	2935.14	534.69	2559.60	14.31	-2.14	0.16	19.57	2.01	2.64	0.17	0.68	2.02	2.64	0.17	0.72	11.59	2.73	0.50	2.64	0.96	2.01	2.75	0.17	0.86	11.65	2.64	0.50	2.75	0.95
SAMPLE2_28.1	2325.06	186.56	2557.72	14.00	1.48	0.12	8.49	2.09	1.72	0.17	0.61	2.10	1.72	0.17	0.68	11.11	1.85	0.48	1.72	0.93	2.09	1.84	0.17	0.84	11.20	1.77	0.48	1.84	0.89
SAMPLE2_92.1	1471.94	86.69	2568.43	12.41	10.90	0.08	6.11	2.33	1.43	0.17	0.54	2.33	1.43	0.17	0.57	9.93	1.54	0.43	1.43	0.93	2.32	1.55	0.17	0.74	10.17	1.49	0.43	1.55	0.88
SAMPLE2_94.1	1977.58	112.73	2541.93	15.97	5.76	0.10	5.98	2.20	1.55	0.17	0.68	2.20	1.55	0.17	0.70	10.48	1.70	0.45	1.55	0.91	2.20	1.70	0.17	0.95	10.54	1.68	0.45	1.70	0.84
SAMPLE1_21.1	6660.43	1100.00	2467.33	17.49	-14.07	0.39	19.39	1.82	2.87	0.17	0.74	1.82	2.87	0.17	0.76	12.68	2.97	0.55	2.87	0.97	1.83	3.00	0.16	1.04	12.11	2.92	0.55	3.00	0.94
SAMPLE3_89.1	2465.73	209.86	2518.34	30.72	0.89	0.13	9.04	2.11	2.03	0.16	1.18	2.10	2.03	0.17	1.35	10.97	2.44	0.48	2.03	0.83	2.10	2.22	0.17	1.83	10.89	2.53	0.48	2.22	0.71
SAMPLE2_43.1	2624.19	250.24	2552.85	16.11	-1.48	0.14	10.17	2.04	1.83	0.17	0.75	2.05	1.83	0.17	0.91	11.20	2.05	0.49	1.83	0.89	2.05	1.95	0.17	0.96	11.41	1.92	0.49	1.95	0.88
SAMPLE2_31.1	706.77	122.92	2549.52	19.16	16.82	0.04	17.70	2.51	1.69	0.17	1.04	2.52	1.69	0.17	1.09	9.09	2.01	0.40	1.69	0.84	2.51	1.79	0.17	1.14	9.30	1.94	0.40	1.79	0.81
SAMPLE3_62.1	991.29	78.76	2491.46	14.76	19.34	0.05	8.14	2.59	1.69	0.17	0.61	2.60	1.69	0.17	0.66	8.81	1.81	0.39	1.69	0.93	2.60	1.83	0.16	0.88	8.66	1.76	0.38	1.83	0.88
SAMPLE3_51.1	872.94	230.24	2672.94	39.83	20.67	0.04	26.95	2.64	7.66	0.17	0.66	2.64	7.66	0.17	0.70	8.66	7.69	0.38	7.66	1.00	2.59	8.37	0.18	2.41	9.69	7.37	0.39	8.37	0.96
SAMPLE1_66.1	2353.40	137.18	2459.79	31.79	3.49	0.12	6.18	2.17	2.04	0.17	1.18	2.17	2.05	0.17	1.22	10.54	2.38	0.46	2.05	0.86	2.18	2.32	0.16	1.88	10.13	2.71	0.46	2.32	0.73
SAMPLE1_8.1	2052.21	202.24	2496.64	21.01	4.52	0.11	10.36	2.19	2.00	0.17	0.98	2.19	2.00	0.17	0.99	10.42	2.24	0.46	2.00	0.90	2.20	2.14	0.16	1.25	10.28	2.19	0.45	2.14	0.83
SAMPLE1_36.1	1675.30	89.80	2566.49	19.26	10.03	0.09	5.59	2.33	1.80	0.17	0.76	2.33	1.80	0.17	0.83	9.79	1.98	0.43	1.80	0.91	2.32	2.01	0.17	1.15	10.17	2.00	0.43	2.01	0.83
SAMPLE3_74.1	2686.65	167.78	2499.14	16.73	-1.35	0.14	6.67	2.07	1.59	0.17	0.73	2.07	1.59	0.17	0.74	10.98	1.75	0.48	1.59	0.91	2.08	1.72	0.16	0.99	10.90	1.72	0.48	1.72	0.83
SAMPLE1_94.1	2543.21	212.83	2490.26	12.25	0.34	0.13	8.91	2.11	1.46	0.17	0.59	2.11	1.46	0.17	0.59	10.79	1.58	0.47	1.46	0.93	2.11	1.54	0.16	0.73	10.65	1.54	0.47	1.54	0.89
SAMPLE1_46.1	2461.81	270.25	2507.01	12.99	0.30	0.13	11.66	2.11	1.73	0.17	0.63	2.11	1.73	0.17	0.65	10.79	1.85	0.47	1.73	0.94	2.11	1.82	0.16	0.77	10.78	1.80	0.47	1.82	0.91
SAMPLE3_99.1	2156.78	611.98	2516.46	24.92	1.62	0.11	29.92	2.14	3.56	0.17	1.38	2.14	3.56	0.16	1.39	10.64	3.82	0.47	3.56	0.93	2.14	3.70	0.17	1.48	10.71	3.71	0.47	3.70	0.92
SAMPLE1_87.1	1890.10	756.68	2502.55	57.41	2.99	0.10	41.93	2.17	2.79	0.16	3.39	2.17	2.79	0.16	3.39	10.47	4.39	0.46	2.79	0.64	2.17	2.88	0.16	3.41	10.45	4.35	0.46	2.88	0.62
SAMPLE3_88.1	-6665.41	1326.96	2593.47	23.40	62.72	-0.28	-16.81	5.64	1.31	0.17	0.89	5.65	1.31	0.16	0.91	4.02	1.60	0.18	1.31	0.82	5.59	1.33	0.17	1.40	4.28	2.04	0.18	1.33	0.73
SAMPLE3_70.1	1939.29	133.12	2510.88	12.56	4.79	0.10	7.20	2.21	1.45	0.17	0.57	2.21	1.45	0.16	0.60	10.26	1.57	0.45	1.45	0.92	2.21	1.55	0.17	0.75	10.31	1.51	0.45	1.55	0.88
SAMPLE3_98.1	984.37	92.35	2509.45	12.14	14.77	0.05	9.61	2.47	1.40	0.16	0.53	2.47	1.40	0.16	0.54	9.17	1.50	0.40	1.40	0.93	2.47	1.49	0.17	0.72	9.21	1.49	0.40	1.49	0.88
SAMPLE3_31.1	2023.61	199.21	2499.23	17.04	3.62	0.11	10.35	2.19	1.66	0.16	0.92	2.19	1.66	0.16	0.93	10.35	1.90	0.46	1.66	0.87	2.19	1.76	0.16	1.01	10.34	1.85	0.46	1.76	0.84
SAMPLE1_9.1	2720.42	326.12	2479.06	18.74	-0.63	0.14	12.81	2.10	1.87	0.17	0.79	2.10	1.87	0.16	0.84	10.79	2.05	0.48	1.87	0.91	2.10	1.97	0.16	1.11	10.64	2.00	0.48	1.97	0.84
SAMPLE2_86.1	-228.45	168.95	2451.55	60.90	43.23	-0.01	-73.54	3.74	2.82	0.16	3.46	3.74	2.82	0.16	3.45	6.01	4.45	0.27	2.82	0.63	3.76	3.01	0.16	3.60	5.86	4.50	0.27	3.01	0.60
SAMPLE1_32.1	2253.92	169.66	2563.97	32.86	0.64	0.12	7.95	2.12	2.11	0.17	1.12	2.14	2.12	0.16	1.55	10.50	2.63	0.47	2.12	0.81	2.12	2.36	0.17	1.96	11.10	2.81	0.47	2.36	0.72
SAMPLE2_26.1	2405.37	123.95	2473.73	17.89	1.24	0.13	5.47	2.15	1.74	0.16	0.65	2.15	1.74	0.16	0.73	10.42	1.89	0.46	1.74	0.92	2.16	1.94	0.16	1.06	10.34	1.87	0.46	1.94	0.85
SAMPLE2_25.1	3010.23	289.52	2494.88	18.85	-4.13	0.16	10.35	2.04	1.92	0.17	0.88	2.05	1.92	0.16	1.11	10.94	2.22	0.49	1.92	0.87	2.04	2.04	0.16	1.12	11.05	2.07	0.49	2.04	0.85
SAMPLE2_68.1	3032.73	205.25	2467.38	15.43	-3.81	0.16	7.29	2.05	1.49	0.16	0.60	2.05	1.49	0.16	0.60	10.91	1.61	0.49	1.49	0.93	2.06	1.59	0.16	0.91	10.81	1.57	0.49	1.59	0.83
SAMPLE3_6.1	3037.97	168.99	2479.41	21.14	-6.24	0.16	5.99	2.00	1.82	0.17	0.76	2.01	1.82	0.16	0.90	11.16	2.03	0.50	1.82	0.90	2.01	2.05	0.16	1.25	11.15	2.06	0.50	2.05	0.81
SAMPLE2_60.1	2648.89	236.59	2486.36	25.81	-1.90	0.14	9.53	2.08	2.16	0.17	1.15	2.10	2.17	0.16	1.52	10.65	2.65	0.48	2.17	0.82	2.09	2.35	0.16	1.53	10.73	2.44	0.48	2.35	0.80
SAMPLE1_22.1	1642.27	278.61	2520.73	31.41	4.44	0.08	17.66	2.22	2.01	0.17	1.74	2.24	2.02	0.16	2.10	9.89	2.91	0.45	2.02	0.69	2.23	2.13	0.17	1.87	10.28	2.65	0.45	2.13	0.71
SAMPLE2_98.1	1543.22	129.44	2488.28	15.53	8.03	0.08	8.71	2.33	1.56	0.16	0.72	2.33	1.56	0.16	0.80	9.52	1.75	0.43	1.56	0.89	2.33	1.66	0.16	0.92	9.66	1.68	0.43	1.66	0.85
SAMPLE2_44.1	2988.18	186.31	2453.19	16.32	-4.74	0.16	6.71	2.05	1.65	0.16	0.54	2.06	1.65	0.16	0.63	10.72	1.76	0.49	1.65	0.93	2.06	1.79	0.16	0.96	10.70	1.71	0.49	1.79	0.85
SAMPLE2_57.1	2649.41	120.55	2445.41	32.26	-3.79	0.14	4.85	2.10	1.93	0.16	0.90	2.11	1.93	0.16	1.13	10.29	2.23	0.47	1.93	0.86	2.10	2.25	0.16	1.91	10.42	2.86	0.48	2.25	0.75
SAMPLE3_96.1	165.77	23.75	2242.95	14.44	51.48	0.01	14.39	4.54	1.35	0.16	0.55	4.54	1.35	0.16	0.56	4.75	1.46	0.22	1.35	0.93	4.61	1.49	0.14	0.84	4.22	1.48	0.22	1.49	0.84
SAMPLE1_5.1	2677.27	524.86	2429.22	18.32	-2.07	0.14	20.93	2.15	2.84	0.16	0.90	2.16	2.84	0.16	1.05	9.94	3.03	0.46	2.84	0.94	2.16	2.95	0.16	1.08	10.07	2.91	0.46	2.95	0.93
SAMPLE2_81.1	266.35	65.31	2459.96	14.53	26.09	0.01	24.68	2.99	1.48	0.16	0.72	2.99	1.48	0.16	0.74	7.18	1.66	0.33	1.48	0.89	2.97	1.57	0.16	0.86	7.44	1.61	0.34	1.57	0.85
SAMPLE3_43.1	613.40	41.39	2471.00	13.29	27.88	0.03	6.85	3.09	1.60	0.15	0.55	3.09	1.60	0.15	0.56	6.84	1.70	0.32	1.60	0.94	3.07	1.74	0.16	0.79	7.26	1.65	0.33	1.74	0.89
SAMPLE2_40.1	2279.23	130.31	2392.64	34.91	1.32	0.12	6.05	2.26	1.79	0.16	1.59	2.26	1.79	0.15	1.67	9.36	2.45	0.44	1.79	0.73	2.26	2.01	0.15	2.05	9.40	2.66	0.44	2.01	0.65
SAMPLE1_97.1	1605.34	356.83	2383.20	31.34	8.64	0.08	23.12	2.46	5.80	0.15	0.51	2.46	5.80	0.15	0.51	8.57	5.83	0.41	5.80	1.00	2.45	6.21	0.15	1.84	8.61	5.54	0.41	6.21	0.96
SAMPLE2_69.1	-531.74	87.32	2371.76	22.35	40.07	-0.03	-16.21	3.75	1.32	0.15	0.75	3.75	1.32	0.15	0.76	5.61	1.52	0.27	1.32	0.87	3.75	1.40	0.15	1.31	5.60	1.85	0.27	1.40	0.71
SAMPLE3_80.1	-116.84	15.26	2454.58	16.32	70.59	-0.01	-13.03	7.92	1.79	0.15	0.91	7.94	1.79	0.15	0.98	2.53	2.04	0.13	1.79	0.88	7.81	1.94	0.16	0.97	2.82	1.97	0.13	1.94	0.88
SAMPLE1_62.1	1422.93	144.49	2349.75	25.79	9.57	0.07	10.52	2.60	2.73	0.15	1.34	2.61	2.73	0.14	1.43	7.63	3.08	0.38	2.73	0.89	2.59	2.93	0.15	1.51	8.00	2.91	0.39	2.93	0.87
SAMPLE3_41.1	1935.73	126.05	2329.84	18.40	2.19	0.10	6.83	2.41	1.81	0.15	0.81	2.42	1.81	0.14	1.01	8.16	2.07	0.41	1.81	0.87	2.41	1.94	0.15	1.07	8.50	1.94	0.42	1.94	0.85
SAMPLE3_12.1	2375.95	200.07	2191.43	26.66	-2.10	0.12	8.93	2.39	2.70	0.14	0.95	2.41	2.71	0.14	1.36	7.86	3.03	0.41	2.71	0.89	2.41	2.92	0.14	1.53	7.83	2.87	0.41	2.92	0.86
SAMPLE3_18.1	1682.39	100.56	2226.50	21.42	6.63	0.09	6.23	2.63	1.84	0.14	0.94	2.64	1.85	0.14	1.05	7.19	2.12	0.38	1.85	0.87	2.63	1.99	0.14	1.24	7.33	2.04	0.38	1.99	0.81
SAMPLE2_5.1	2473.77	144.06	2192.14	23.83	-3.58	0.13	6.19	2.37	1.91	0.14	0.99	2.38	1.92	0.14	1.22	7.97	2.27	0.42	1.92	0.84	2.38	2.08	0.14	1.37	7.95	2.14	0.42	2.08	0.79
SAMPLE3_29.1	1396.52	138.48	2202.78	30.59	8.71	0.07	10.26	2.73	1.68	0.14	1.61	2.73	1.68	0.14	1.66	6.85	2.36	0.37	1.68	0.71	2.72	1.77	0.14	1.76	6.98	2.36	0.37	1.77	0.67
SAMPLE2_73.1	2272.53	127.62	2118.48	13.15	0.71	0.12	5.94	2.52	1.41	0.13	0.58	2.52	1.41	0.14	0.61	7.41	1.54	0.40	1.41	0.92	2.53	1.48	0.13	0.75	7.17	1.50	0.40	1.48	0.87
SAMPLE2_15.1	-27.06	25.37	2119.86	14.56	51.46	0.00	-93.69	5.23	2.10	0.13	0.55	5.22	2.10	0.13	0.57	3.52	2.18	0.19	2.10	0.97	5.23	2.24	0.13	0.83	3.47	2.17	0.19	2.24	0.93
SAMPLE1_70.1	625.28	134.75	2236.67	21.53	17.36	0.03	21.89	3.08	3.24	0.13	0.79	3.09	3.24	0.13	0.94	5.90	3.38	0.32	3.24	0.96	3.06	3.40	0.14	1.24	6.34	3.23	0.33	3.40	0.93
SAMPLE1_18.1	-944.47	157.13	2047.46	39.53	45.51	-0.05	-16.25	4.67	1.82	0.14	1.99	4.70	1.82	0.13	2.27	3.88	2.91	0.21	1.82	0.63	4.73	1.89	0.13	2.24	3.68	2.86	0.21	1.89	0.63
SAMPLE1_75.1	1645.35	126.59	2152.58	19.00	5.74	0.08	8.01	2.72	2.36	0.13	0.54	2.72	2.36	0.13	0.55	6.68	2.42	0.37	2.36	0.97	2.71	2.51	0.13	1.09	6.81	2.33	0.37	2.51	0.90
SAMPLE1_11.1	2881.83	165.07	2033.46	47.04	-10.31	0.15	6.15	2.33	1.90	0.13	1.01	2.34	1.90	0.13	1.14	7.74	2.22	0.43	1.90	0.86	2.35	2.16	0.13	2.66	7.35	3.30	0.43	2.16	0.60
SAMPLE2_19.1	2265.25	111.91	2104.74	24.71	-2.26	0.12	5.22	2.51	1.86	0.13	0.98	2.52	1.87	0.13	1.26	7.16	2.25	0.40	1.87	0.83	2.52	2.04	0.13	1.41	7.13	2.12	0.40	2.04	0.77
SAMPLE2_91.1	1912.90	68.92	2083.32	23.62	3.64	0.10	3.78	2.69	1.51	0.14	0.66	2.71	1.52	0.13	1.25	6.60	1.97	0.37	1.52	0.77	2.71	1.68	0.13	1.34	6.56	1.95	0.37	1.68	0.74
SAMPLE2_76.1	1429.71	89.13	2078.88	18.32	9.09	0.07	6.46	2.88	1.39	0.13	0.96	2.88	1.39	0.13	0.96	6.16	1.69	0.35	1.39	0.82	2.88	1.47	0.13	1.04	6.15	1.66	0.35	1.47	0.78
SAMPLE1_2.1	1675.26	125.58	2114.41	32.92	5.39	0.09	7.81	2.77	2.40	0.13	1.38	2.78	2.40	0.13	1.50	6.35	2.83	0.36	2.40	0.85	2.77	2.59	0.13	1.88	6.52	2.74	0.36	2.59	0.75
SAMPLE3_81.1	2121.80	100.24	2096.76	14.86	-1.50	0.11	4.98	2.60	1.44	0.13	0.66	2.60	1.45	0.13	0.71	6.80	1.61	0.38	1.45	0.90	2.59	1.53	0.13	0.85	6.91	1.55	0.39	1.53	0.85
SAMPLE1_55.1	1654.37	96.96	2051.18	15.74	6.16	0.09	6.10	2.82	1.68	0.13	0.68	2.82	1.68	0.13	0.72	6.23	1.83	0.35	1.68	0.92	2.83	1.77	0.13	0.89	6.18	1.77	0.35	1.77	0.87
SAMPLE1_30.1	2234.13	148.58	2097.83	22.80	-4.33	0.12	7.03	2.55	2.59	0.13	0.87	2.56	2.59	0.13	1.03	6.84	2.79	0.39	2.59	0.93	2.55	2.79	0.13	1.30	7.04	2.62	0.39	2.79	0.89
SAMPLE2_88.1	1512.16	59.03	2125.57	14.88	6.79	0.08	4.05	2.88	1.42	0.13	0.63	2.88	1.43	0.13	0.73	6.01	1.60	0.35	1.43	0.89	2.86	1.53	0.13	0.85	6.36	1.52	0.35	1.53	0.84
SAMPLE3_47.1	1858.37	169.63	1976.90	32.16	4.30	0.10	9.55	2.80	2.16	0.13	1.42	2.82	2.16	0.13	1.93	6.13	2.90	0.35	2.16	0.75	2.83	2.29	0.12	1.81	5.91	2.62	0.35	2.29	0.74
SAMPLE2_12.1	1726.12	204.48	2030.92	23.15	2.17	0.09	12.36	2.77	1.69	0.13	1.26	2.78	1.69	0.12	1.29	6.17	2.13	0.36	1.69	0.79	2.78	1.75	0.13	1.31	6.22	2.08	0.36	1.75	0.78
SAMPLE1_65.1	1943.48	265.80	2057.34	51.49	-0.89	0.10	14.34	2.69	1.76	0.13	2.68	2.70	1.77	0.12	3.12	6.32	3.58	0.37	1.77	0.49	2.69	1.87	0.13	2.92	6.50	3.40	0.37	1.87	0.52
SAMPLE3_53.1	1519.50	123.27	1349.65	64.14	29.74	0.08	8.42	3.86	4.19	0.13	1.43	3.89	4.19	0.12	2.17	4.39	4.72	0.26	4.19	0.89	4.05	4.67	0.09	3.32	2.94	4.84	0.25	4.67	0.76
SAMPLE3_3.1	2009.77	61.64	2106.35	3035.46	-1.39	0.10	3.22	2.70	2.58	0.13	1.88	2.72	2.60	0.12	2.90	6.23	3.89	0.37	2.60	0.67	2.69	5.59	0.13	172.97	6.69	175.98	0.37	5.59	0.55
SAMPLE2_16.1	2565.88	170.11	2059.23	23.16	-10.62	0.14	7.06	2.50	1.89	0.13	1.06	2.52	1.90	0.12	1.58	6.64	2.47	0.40	1.90	0.77	2.50	2.00	0.13	1.31	7.00	2.13	0.40	2.00	0.80
SAMPLE2_3.1	2251.05	367.82	1999.39	15.44	-3.60	0.12	17.27	2.69	3.64	0.12	0.67	2.69	3.64	0.12	0.74	6.21	3.72	0.37	3.64	0.98	2.69	3.76	0.12	0.87	6.31	3.61	0.37	3.76	0.97
SAMPLE3_79.1	877.44	75.56	2028.31	10.82	12.04	0.04	8.80	3.18	1.35	0.12	0.53	3.18	1.35	0.12	0.53	5.26	1.45	0.31	1.35	0.93	3.16	1.40	0.12	0.61	5.45	1.42	0.32	1.40	0.91
SAMPLE3_78.1	777.35	124.90	1675.72	57.24	37.26	0.04	16.38	4.47	7.42	0.12	1.47	4.47	7.42	0.12	1.48	3.73	7.56	0.22	7.42	0.98	4.56	8.01	0.10	3.10	3.11	7.61	0.22	8.01	0.92
SAMPLE3_58.1	2147.41	141.23	2023.26	26.30	-4.97	0.11	6.93	2.66	1.97	0.13	1.17	2.68	1.97	0.12	1.79	6.20	2.67	0.37	1.97	0.74	2.67	2.09	0.12	1.48	6.45	2.27	0.38	2.09	0.77
SAMPLE3_69.1	1662.91	178.68	1824.57	50.52	10.55	0.09	11.19	3.15	6.04	0.12	0.66	3.16	6.04	0.12	0.72	5.24	6.09	0.32	6.04	0.99	3.19	6.66	0.11	2.79	4.83	5.95	0.31	6.66	0.91
SAMPLE3_9.1	2135.45	100.75	1986.78	23.50	-4.49	0.11	4.97	2.69	1.81	0.12	0.94	2.71	1.81	0.12	1.32	6.09	2.24	0.37	1.81	0.81	2.70	1.96	0.12	1.32	6.23	2.03	0.37	1.96	0.78
SAMPLE3_34.1	2040.76	333.75	1971.55	27.06	-1.52	0.11	17.19	2.78	1.68	0.12	1.49	2.79	1.68	0.12	1.56	5.92	2.29	0.36	1.68	0.73	2.78	1.71	0.12	1.52	6.00	2.23	0.36	1.71	0.73
SAMPLE1_61.1	1324.12	94.34	1981.60	13.53	7.34	0.07	7.36	3.05	1.65	0.12	0.62	3.05	1.65	0.12	0.68	5.40	1.78	0.33	1.65	0.92	3.05	1.72	0.12	0.76	5.51	1.72	0.33	1.72	0.90
SAMPLE3_20.1	2168.20	253.37	1968.10	13.53	-2.58	0.11	12.32	2.76	1.67	0.12	0.69	2.77	1.67	0.12	0.77	5.94	1.84	0.36	1.67	0.91	2.76	1.70	0.12	0.76	6.03	1.78	0.36	1.70	0.91
SAMPLE2_4.1	2891.91	233.12	1886.25	23.89	-7.48	0.15	8.65	2.66	1.83	0.12	0.96	2.66	1.83	0.12	1.02	6.12	2.09	0.38	1.83	0.87	2.67	1.90	0.12	1.33	5.95	2.05	0.37	1.90	0.78
SAMPLE3_90.1	1562.93	35.86	1878.47	32.11	12.02	0.08	2.38	3.28	1.46	0.12	0.79	3.28	1.46	0.12	0.79	4.94	1.66	0.30	1.46	0.88	3.29	1.71	0.11	1.78	4.82	2.38	0.30	1.71	0.67
SAMPLE3_71.1	2190.96	179.32	1916.58	15.89	-2.43	0.11	8.64	2.81	1.52	0.12	0.77	2.82	1.52	0.12	0.86	5.74	1.75	0.35	1.52	0.87	2.82	1.57	0.12	0.89	5.74	1.69	0.35	1.57	0.85
SAMPLE2_13.1	1078.55	51.08	1616.03	25.88	28.30	0.05	4.86	4.04	1.70	0.12	0.90	4.05	1.70	0.12	1.06	3.98	2.01	0.25	1.70	0.85	4.13	1.84	0.10	1.39	3.33	2.01	0.24	1.84	0.74
SAMPLE2_72.1	1701.75	79.18	1881.40	16.64	4.28	0.09	4.85	3.04	1.44	0.12	0.74	3.03	1.44	0.12	0.81	5.31	1.65	0.33	1.44	0.87	3.04	1.52	0.12	0.92	5.22	1.59	0.33	1.52	0.82
SAMPLE2_74.1	2079.47	98.37	1787.27	22.62	1.60	0.11	4.98	2.96	1.53	0.12	0.96	2.95	1.53	0.12	1.02	5.44	1.84	0.34	1.53	0.83	2.98	1.63	0.11	1.24	5.06	1.82	0.34	1.63	0.75
SAMPLE1_90.1	-597.44	82.82	2013.02	22.04	47.09	-0.03	-13.66	5.48	4.54	0.12	1.22	5.49	4.54	0.12	1.31	2.93	4.72	0.18	4.54	0.96	5.45	4.67	0.12	1.24	3.13	4.61	0.18	4.67	0.96
SAMPLE2_75.1	1784.44	85.28	1884.81	12.65	2.20	0.09	4.99	2.99	1.37	0.12	0.56	2.98	1.37	0.12	0.58	5.37	1.48	0.34	1.37	0.92	2.99	1.43	0.12	0.70	5.32	1.45	0.33	1.43	0.88
SAMPLE3_28.1	2396.57	120.24	1894.17	27.44	-8.63	0.13	5.32	2.67	1.90	0.12	1.09	2.69	1.91	0.12	1.66	5.96	2.53	0.37	1.91	0.76	2.69	2.06	0.12	1.53	5.94	2.21	0.37	2.06	0.75
SAMPLE3_85.1	1973.04	75.60	1907.63	17.62	-1.94	0.10	4.02	2.87	1.82	0.12	0.65	2.87	1.82	0.12	0.67	5.58	1.94	0.35	1.82	0.94	2.87	1.97	0.12	0.98	5.62	1.86	0.35	1.97	0.87
SAMPLE2_10.1	1796.55	123.83	1891.02	17.23	1.43	0.09	7.20	2.96	1.69	0.12	0.78	2.97	1.69	0.12	0.91	5.39	1.92	0.34	1.69	0.88	2.97	1.76	0.12	0.96	5.37	1.84	0.34	1.76	0.86
SAMPLE2_67.1	-66.82	69.15	1910.97	13.32	26.86	0.00	-103.31	4.00	1.38	0.12	0.65	4.00	1.38	0.12	0.71	3.99	1.56	0.25	1.38	0.89	4.00	1.42	0.12	0.74	4.04	1.51	0.25	1.42	0.87
SAMPLE2_77.1	405.15	22.31	1868.06	15.77	41.98	0.02	5.56	5.04	1.65	0.12	0.54	5.05	1.65	0.12	0.61	3.16	1.76	0.20	1.65	0.94	5.06	1.76	0.11	0.87	3.11	1.77	0.20	1.76	0.88
SAMPLE1_85.1	1916.41	80.05	1857.97	15.41	0.88	0.10	4.38	2.96	1.41	0.11	0.66	2.96	1.41	0.12	0.70	5.40	1.58	0.34	1.41	0.90	2.96	1.49	0.11	0.85	5.28	1.53	0.34	1.49	0.84
SAMPLE1_53.1	1648.56	93.08	1882.86	14.57	3.97	0.08	5.88	3.05	1.64	0.12	0.63	3.05	1.64	0.12	0.65	5.23	1.77	0.33	1.64	0.93	3.06	1.72	0.12	0.81	5.20	1.72	0.33	1.72	0.89
SAMPLE2_53.1	2132.59	135.36	1877.16	16.47	-2.40	0.11	6.69	2.86	1.68	0.12	0.75	2.86	1.68	0.12	0.79	5.57	1.86	0.35	1.68	0.91	2.87	1.75	0.11	0.91	5.52	1.81	0.35	1.75	0.87
SAMPLE1_86.1	1048.05	368.64	1905.16	7.83	2.09	0.05	36.09	3.00	1.35	0.12	0.42	3.00	1.35	0.12	0.43	5.30	1.42	0.33	1.35	0.95	3.00	1.36	0.12	0.44	5.36	1.41	0.33	1.36	0.95
SAMPLE2_14.1	2150.72	139.68	1891.25	17.47	-3.50	0.11	6.85	2.84	1.70	0.12	0.81	2.84	1.70	0.12	0.82	5.60	1.89	0.35	1.70	0.90	2.84	1.77	0.12	0.97	5.62	1.85	0.35	1.77	0.86
SAMPLE1_31.1	2410.18	138.42	1759.31	24.44	-6.60	0.13	6.09	2.76	2.94	0.11	0.73	2.76	2.95	0.12	0.75	5.76	3.04	0.36	2.95	0.97	2.79	3.21	0.11	1.34	5.33	2.89	0.36	3.21	0.91
SAMPLE1_67.1	1771.69	80.59	1872.29	14.41	2.16	0.09	4.75	3.01	1.41	0.12	0.63	3.01	1.41	0.12	0.63	5.27	1.54	0.33	1.41	0.91	3.01	1.48	0.11	0.80	5.24	1.51	0.33	1.48	0.86
SAMPLE2_79.1	1619.42	124.93	1896.52	10.86	1.99	0.08	8.03	3.01	1.36	0.12	0.52	3.01	1.36	0.12	0.57	5.28	1.48	0.33	1.36	0.92	3.01	1.40	0.12	0.60	5.32	1.43	0.33	1.40	0.91
SAMPLE3_91.1	950.57	309.91	1883.08	14.65	4.23	0.05	33.38	3.08	1.34	0.12	0.80	3.08	1.34	0.12	0.80	5.15	1.56	0.32	1.34	0.86	3.08	1.35	0.12	0.81	5.15	1.55	0.32	1.35	0.85
SAMPLE1_69.1	1672.62	157.19	1901.30	12.19	0.84	0.09	9.79	2.97	1.39	0.12	0.61	2.98	1.39	0.12	0.68	5.33	1.55	0.34	1.39	0.90	2.97	1.43	0.12	0.68	5.40	1.50	0.34	1.43	0.89
SAMPLE3_19.1	1551.36	135.04	1907.00	14.12	2.48	0.08	9.04	3.03	1.66	0.12	0.68	3.03	1.66	0.12	0.72	5.24	1.81	0.33	1.66	0.92	3.02	1.71	0.12	0.79	5.33	1.76	0.33	1.71	0.90
SAMPLE1_54.1	1742.64	117.63	1932.85	20.60	-0.19	0.09	7.05	2.94	1.70	0.12	0.77	2.95	1.71	0.11	1.19	5.38	2.08	0.34	1.71	0.82	2.94	1.78	0.12	1.15	5.56	1.84	0.34	1.78	0.80
SAMPLE2_23.1	1983.74	71.92	1882.44	18.70	-2.31	0.10	3.81	2.89	1.65	0.12	0.70	2.89	1.65	0.11	0.77	5.48	1.82	0.35	1.65	0.91	2.89	1.79	0.12	1.04	5.50	1.76	0.35	1.79	0.83
SAMPLE3_27.1	1502.93	64.70	1888.83	17.40	7.11	0.08	4.47	3.18	1.67	0.12	0.71	3.18	1.67	0.11	0.77	4.98	1.84	0.31	1.67	0.91	3.18	1.78	0.12	0.97	5.01	1.77	0.31	1.78	0.85
SAMPLE3_22.1	1669.38	80.37	1848.16	24.08	5.15	0.09	5.02	3.12	1.80	0.12	1.01	3.12	1.80	0.11	1.05	5.08	2.08	0.32	1.80	0.86	3.12	1.92	0.11	1.33	4.99	2.04	0.32	1.92	0.78
SAMPLE2_100.1	1284.89	364.09	1886.17	10.98	2.43	0.07	29.25	3.03	1.84	0.12	0.56	3.03	1.84	0.11	0.59	5.22	1.93	0.33	1.84	0.95	3.03	1.86	0.12	0.61	5.25	1.91	0.33	1.86	0.95
SAMPLE1_57.1	1787.06	133.73	1880.03	11.17	0.86	0.09	7.82	2.98	1.61	0.12	0.52	2.98	1.61	0.11	0.54	5.31	1.70	0.34	1.61	0.95	2.98	1.66	0.12	0.62	5.32	1.66	0.34	1.66	0.93
SAMPLE1_51.1	1498.87	87.35	1917.94	17.66	4.59	0.08	6.05	3.10	1.72	0.12	0.79	3.10	1.72	0.11	0.91	5.09	1.95	0.32	1.72	0.89	3.10	1.81	0.12	0.98	5.23	1.85	0.32	1.81	0.86
SAMPLE3_82.1	1898.06	119.14	1892.00	12.59	-1.03	0.10	6.58	2.93	1.39	0.11	0.60	2.93	1.39	0.11	0.62	5.39	1.52	0.34	1.39	0.91	2.93	1.43	0.12	0.70	5.45	1.48	0.34	1.43	0.89
SAMPLE2_83.1	1741.54	74.92	1861.82	20.52	3.19	0.09	4.49	3.07	1.93	0.11	0.72	3.06	1.93	0.11	0.79	5.16	2.08	0.33	1.93	0.93	3.06	2.07	0.11	1.14	5.12	2.06	0.33	2.07	0.85
SAMPLE3_75.1	1847.25	45.05	1818.39	23.49	2.22	0.10	2.55	3.03	1.44	0.11	0.73	3.03	1.44	0.11	0.74	5.21	1.62	0.33	1.44	0.89	3.04	1.63	0.11	1.29	5.04	1.81	0.33	1.63	0.72
SAMPLE3_32.1	1705.90	47.94	1921.50	75.21	4.79	0.09	2.93	3.11	1.90	0.12	1.20	3.12	1.91	0.11	1.48	5.07	2.41	0.32	1.91	0.79	3.10	2.33	0.12	4.20	5.23	5.16	0.32	2.33	0.60
SAMPLE1_45.1	1996.15	80.41	1873.96	22.88	-2.70	0.10	4.23	2.89	1.86	0.11	0.86	2.89	1.86	0.11	0.87	5.47	2.05	0.35	1.86	0.91	2.89	2.02	0.11	1.27	5.47	2.03	0.35	2.02	0.80

Spot	204Pb/206Pb	±%	207Pb/206Pb	±%	208Pb/206Pb	±%	206Pb/238U	±%	% 206Pb$_c$	ppm U	ppm Th	4-corr ppm 206Pb*	4-corr ppm 208Pb*	232Th/238U	±%
SAMPLE1_92.1	0.00	44.72	0.11	0.67	0.10	1.30	0.79	1.79	-0.06	215.76	74.28	63.36	6.57	0.36	0.51
SAMPLE1_96.1	0.00	41.60	0.11	0.51	0.16	3.88	0.75	1.43	0.03	476.19	251.45	120.79	19.91	0.55	2.31
SAMPLE2_27.1	0.00	21.94	0.12	0.62	0.05	1.64	0.78	1.86	0.20	297.08	48.68	84.16	4.13	0.17	0.65
SAMPLE1_40.1	0.00	50.00	0.11	0.70	0.08	1.57	0.79	2.47	0.05	225.73	55.48	67.13	5.07	0.25	0.64
SAMPLE2_52.1	0.00	35.36	0.12	0.70	0.19	1.03	0.83	1.41	0.10	232.79	146.98	69.94	13.38	0.65	0.52
SAMPLE2_55.1	0.00	33.33	0.12	0.68	0.17	1.07	0.84	0.61	0.11	223.47	125.82	65.44	10.77	0.58	0.53
SAMPLE1_27.1	0.00	48.25	0.12	0.56	0.04	3.65	0.77	4.39	0.09	473.55	67.48	128.75	5.56	0.15	0.60
SAMPLE2_9.1	0.00	22.94	0.12	0.71	0.15	1.16	0.85	1.14	0.25	209.45	106.59	61.09	9.03	0.53	0.54
SAMPLE3_86.1	0.00	50.00	0.11	1.19	0.19	1.08	0.75	2.23	0.06	209.17	127.48	60.42	11.42	0.63	0.47
SAMPLE1_68.1	0.00	32.73	0.11	0.58	0.08	1.24	0.81	2.46	0.08	255.58	72.16	70.67	5.84	0.29	0.50
SAMPLE2_21.1	0.00	21.49	0.12	0.85	0.17	1.32	0.83	2.41	0.41	148.20	83.33	42.28	7.08	0.58	0.59
SAMPLE3_54.1	0.00	21.12	0.12	0.58	0.16	0.91	0.83	2.57	0.24	304.50	158.60	89.29	13.85	0.54	0.50
SAMPLE1_6.1	0.00	40.83	0.11	0.66	0.07	1.53	0.82	2.48	0.06	302.25	69.45	86.12	5.71	0.24	0.65
SAMPLE3_5.1	0.00	20.85	0.12	0.70	0.12	1.26	0.78	2.14	0.29	202.91	86.67	57.59	6.82	0.44	0.92
SAMPLE3_11.1	0.00	28.87	0.12	0.68	0.15	1.11	0.76	1.77	0.14	224.69	119.88	59.18	8.79	0.55	0.54
SAMPLE3_42.1	0.00	21.82	0.12	0.50	0.07	2.43	0.84	2.77	0.12	381.23	84.09	106.67	6.87	0.23	0.54
SAMPLE2_58.1	0.00	27.74	0.12	0.58	0.09	1.16	0.83	1.64	0.11	315.38	102.13	90.61	8.46	0.33	0.54
SAMPLE2_20.1	0.00	20.56	0.12	0.75	0.11	1.40	0.87	1.63	0.34	181.20	66.61	56.17	6.01	0.38	0.60
SAMPLE1_80.1	0.00	23.64	0.12	0.59	0.06	1.44	0.83	1.36	0.13	335.72	71.06	97.26	5.97	0.22	0.51
SAMPLE1_71.1	0.00	65.47	0.11	2.55	0.08	2.51	0.70	1.26	-0.02	389.67	122.31	95.24	7.50	0.32	3.01
SAMPLE3_16.1	0.00	30.15	0.12	0.67	0.08	1.48	0.75	3.59	0.11	235.92	57.42	64.92	4.75	0.25	0.61
SAMPLE3_7.1	0.00	20.85	0.12	0.73	0.12	1.33	0.79	2.52	0.31	193.42	75.13	56.15	6.41	0.40	0.60
SAMPLE1_39.1	0.00	20.00	0.12	0.56	0.06	1.35	0.77	2.73	0.19	390.97	87.76	104.08	6.19	0.23	1.24
SAMPLE2_34.1	0.00	19.25	0.12	0.62	0.07	1.37	0.89	1.15	0.25	297.49	72.16	84.81	5.92	0.25	0.59
SAMPLE3_50.1	0.00	25.00	0.11	0.53	0.27	0.68	0.72	3.12	0.10	451.07	392.15	133.28	35.68	0.90	0.85
SAMPLE1_63.1	0.00	35.36	0.11	0.58	0.11	2.35	0.89	1.13	0.06	396.43	142.38	122.76	13.34	0.37	1.22
SAMPLE3_45.1	0.00	33.33	0.11	0.60	0.07	1.46	0.78	2.35	0.08	252.36	56.83	68.67	4.41	0.23	0.58
SAMPLE2_82.1	0.00	31.45	0.11	0.50	0.09	1.03	0.72	2.02	0.06	399.53	122.63	107.02	9.49	0.32	0.45
SAMPLE3_17.1	0.00	23.87	0.11	0.52	0.11	2.28	0.81	2.51	0.11	367.31	130.71	105.70	11.69	0.37	2.28
SAMPLE2_42.1	0.00	33.34	0.12	0.99	0.16	1.61	0.82	0.75	0.24	107.58	54.95	31.29	4.76	0.53	0.66
SAMPLE1_20.1	0.00	18.25	0.12	1.05	0.03	4.71	0.61	4.58	0.33	635.50	58.70	141.16	2.95	0.10	2.64
SAMPLE3_44.1	0.00	30.15	0.12	0.73	0.14	1.22	0.69	3.35	0.14	201.16	98.17	48.61	6.70	0.50	0.54
SAMPLE3_83.1	0.00	40.83	0.11	0.59	0.22	2.21	0.70	2.71	-0.05	403.85	283.80	101.09	22.75	0.73	0.74
SAMPLE2_6.1	0.00	25.82	0.12	0.75	0.18	1.14	0.85	1.16	0.22	240.13	140.49	70.27	12.18	0.60	0.54
SAMPLE3_48.1	0.00	18.90	0.12	0.68	0.08	1.45	0.89	0.90	0.33	185.48	49.45	54.20	4.20	0.28	0.61
SAMPLE2_17.1	0.00	34.19	0.11	1.33	0.19	0.93	0.82	1.07	0.08	334.86	210.82	98.34	18.26	0.65	0.50
SAMPLE1_88.1	0.00	35.36	0.11	0.54	0.09	1.13	0.80	1.75	0.06	359.51	107.96	103.75	9.12	0.31	0.97
SAMPLE2_95.1	0.00	68.83	0.11	0.61	0.19	1.64	0.74	2.86	0.02	294.59	188.09	82.36	15.72	0.66	1.82
SAMPLE3_73.1	0.00	106.07	0.11	0.63	0.11	1.18	0.67	2.97	0.01	341.06	167.43	83.15	9.19	0.51	0.45
SAMPLE2_66.1	0.00	58.84	0.11	0.59	0.18	0.98	0.77	1.37	0.03	281.10	177.53	79.12	14.51	0.65	0.42
SAMPLE2_35.1	0.00	24.26	0.12	0.74	0.17	2.20	0.81	2.33	0.23	207.82	115.78	61.91	10.48	0.58	0.54
SAMPLE3_67.1	0.00	90.45	0.11	0.99	0.30	1.19	0.78	0.72	0.03	100.44	99.64	30.92	9.37	1.02	0.52
SAMPLE1_48.1	0.00	25.36	0.12	0.67	0.16	1.05	0.81	1.25	0.17	259.29	142.14	76.10	12.39	0.57	1.13
SAMPLE1_100.1	0.00	35.61	0.11	0.87	0.23	1.19	0.77	0.65	0.16	133.52	105.27	37.90	8.72	0.81	0.52
SAMPLE3_38.1	0.00	26.73	0.12	0.82	0.24	1.10	0.75	3.43	0.24	153.62	120.00	43.93	10.22	0.81	0.56
SAMPLE1_44.1	0.00	20.14	0.12	0.66	0.08	1.41	0.79	3.07	0.26	268.33	74.08	74.26	5.62	0.29	0.61
SAMPLE3_72.1	0.00	32.54	0.11	0.61	0.21	0.86	0.77	0.91	0.09	282.21	201.89	78.57	16.61	0.74	0.41
SAMPLE3_95.1	0.00	27.50	0.11	0.56	0.16	0.88	0.60	0.89	-0.11	433.83	243.70	93.53	15.76	0.58	0.39
SAMPLE2_41.1	0.00	18.26	0.12	0.78	0.20	1.12	0.82	1.84	0.47	199.98	130.62	60.59	11.75	0.67	0.55
SAMPLE1_25.1	0.00	25.82	0.12	0.81	0.09	1.65	0.80	3.15	0.25	160.28	48.32	43.57	3.75	0.31	0.67
SAMPLE2_51.1	0.00	30.15	0.12	1.09	0.30	1.32	0.82	2.16	0.34	90.43	89.52	26.45	7.73	1.02	0.61
SAMPLE1_59.1	0.00	18.29	0.12	0.69	0.17	1.07	0.77	2.38	0.35	249.35	134.30	66.28	10.55	0.56	0.54
SAMPLE3_52.1	0.00	28.87	0.12	0.91	0.45	1.56	0.54	2.75	0.27	184.61	204.45	42.26	18.90	1.14	0.51
SAMPLE1_77.1	0.00	44.23	0.11	0.70	0.09	1.64	0.75	2.44	-0.06	231.31	72.77	64.57	5.78	0.32	0.53
SAMPLE1_58.1	0.00	21.01	0.11	0.49	0.10	0.94	0.85	1.74	0.12	498.87	159.51	143.99	13.82	0.33	0.52
SAMPLE3_1.1	0.00	20.00	0.12	0.64	0.20	1.04	0.82	2.35	0.26	260.75	175.84	76.16	14.68	0.70	0.50
SAMPLE1_50.1	0.00	31.45	0.11	0.89	0.34	1.03	0.75	1.88	0.20	131.61	149.66	34.82	11.84	1.17	0.54
SAMPLE1_81.1	0.00	44.72	0.11	0.67	0.08	2.93	0.77	1.10	0.06	190.36	52.32	50.83	4.20	0.28	0.54
SAMPLE1_15.1	0.00	25.60	0.12	0.67	0.19	0.99	0.85	4.33	0.35	259.74	165.91	77.69	14.37	0.66	0.92
SAMPLE2_65.1	0.00	20.18	0.12	0.84	0.11	1.59	0.85	1.90	0.45	133.97	47.63	37.84	3.89	0.37	0.65
SAMPLE1_10.1	0.00	27.98	0.11	0.75	0.10	1.49	0.82	1.56	0.18	228.75	72.42	66.70	6.21	0.33	0.74
SAMPLE2_47.1	0.00	18.47	0.12	0.68	0.19	1.02	0.81	2.54	0.35	239.40	152.05	69.85	12.57	0.66	0.53
SAMPLE3_100.1	0.00	25.82	0.11	0.59	0.17	0.94	0.77	1.81	0.14	317.91	172.95	94.63	15.51	0.56	0.73
SAMPLE1_29.1	0.00	150.00	0.11	0.56	0.02	2.22	0.75	1.92	0.00	456.53	47.73	120.53	2.59	0.11	1.36
SAMPLE1_74.1	0.00	37.21	0.11	0.86	0.15	1.39	0.78	1.55	0.14	138.01	71.10	39.10	5.93	0.53	0.55
SAMPLE3_57.1	0.00	27.74	0.11	0.81	0.15	1.30	0.80	2.10	0.22	150.81	76.74	44.92	6.80	0.53	0.58
SAMPLE2_24.1	0.00	21.06	0.12	0.62	0.25	0.81	0.86	2.61	0.30	292.95	246.48	84.62	21.22	0.87	0.89
SAMPLE3_77.1	0.00	46.85	0.11	0.90	0.18	1.35	0.82	1.86	-0.10	135.78	83.51	38.68	7.04	0.64	0.54
SAMPLE3_15.1	0.00	31.62	0.11	0.76	0.15	1.22	0.73	2.31	0.14	195.37	96.47	55.07	8.39	0.51	1.04
SAMPLE2_62.1	0.00	19.25	0.12	0.67	0.17	2.15	0.85	2.57	0.31	227.47	128.20	66.00	10.71	0.58	0.52
SAMPLE2_18.1	0.00	35.36	0.11	0.77	0.18	1.06	0.83	1.48	0.10	247.03	148.97	72.48	12.96	0.62	0.53
SAMPLE3_46.1	0.00	37.80	0.11	0.99	0.13	1.71	0.67	2.60	0.18	121.19	54.02	31.12	4.09	0.46	0.65
SAMPLE2_8.1	0.00	30.15	0.11	0.93	0.20	1.35	0.84	1.77	0.25	132.41	86.08	38.45	7.38	0.67	1.08
SAMPLE2_7.1	0.00	22.94	0.11	0.66	0.09	1.35	0.84	2.36	0.20	267.39	77.56	78.24	6.63	0.30	0.59
SAMPLE3_64.1	0.00	18.29	0.12	0.79	0.09	1.62	0.78	2.08	0.48	148.93	45.27	43.45	3.70	0.31	0.64
SAMPLE3_21.1	0.00	25.82	0.11	0.81	0.13	1.43	0.80	2.67	0.25	151.83	63.87	43.26	5.44	0.43	0.62
SAMPLE2_33.1	0.00	25.01	0.12	1.39	0.40	3.38	0.85	2.46	0.86	50.51	64.09	14.74	5.71	1.31	1.31
SAMPLE1_52.1	0.00	22.81	0.12	0.88	0.19	1.30	0.80	3.47	0.38	151.61	92.63	44.26	8.11	0.63	0.60
SAMPLE3_68.1	0.00	37.80	0.11	0.70	0.09	1.43	0.72	3.05	0.09	225.94	66.26	66.84	6.08	0.30	3.70
SAMPLE3_33.1	0.00	21.33	0.11	0.47	0.11	1.57	0.42	3.03	0.17	881.67	359.74	126.80	13.57	0.42	0.46
SAMPLE2_61.1	0.00	25.82	0.11	0.76	0.06	1.85	0.83	3.89	0.22	191.92	42.51	54.76	3.18	0.23	0.68
SAMPLE3_94.1	0.00	37.80	0.11	0.72	0.19	1.05	0.83	0.56	0.09	216.90	147.57	64.09	12.45	0.70	1.16
SAMPLE3_76.1	0.00	68.83	0.11	0.59	0.26	0.76	0.64	2.09	0.02	394.86	353.85	88.83	23.29	0.93	0.39
SAMPLE2_90.1	0.00	24.75	0.11	0.68	0.07	2.85	0.68	1.78	0.19	267.74	84.14	67.79	4.71	0.32	0.87
SAMPLE2_39.1	0.00	22.94	0.11	0.73	0.18	1.10	0.79	1.05	0.26	221.83	137.92	63.75	11.24	0.64	0.52
SAMPLE3_37.1	0.00	57.74	0.11	1.31	0.22	1.78	0.73	2.48	0.13	56.10	41.43	14.74	3.29	0.76	0.70
SAMPLE3_13.1	0.00	22.94	0.11	0.81	0.10	1.60	0.71	2.97	0.32	170.92	53.99	48.14	4.58	0.33	0.64
SAMPLE3_2.1	0.00	20.85	0.11	1.21	0.07	1.38	0.82	1.91	0.20	317.41	79.50	87.78	5.57	0.26	0.57
SAMPLE1_95.1	0.00	94.87	0.11	0.52	0.09	1.07	0.62	2.01	0.01	416.61	126.85	90.33	7.84	0.31	0.42
SAMPLE2_32.1	0.00	19.61	0.12	0.80	0.12	1.44	0.90	1.35	0.44	166.89	66.16	48.20	5.46	0.41	0.61
SAMPLE3_26.1	0.00	23.57	0.11	0.96	0.11	1.03	0.60	4.12	0.13	443.96	170.10	98.83	10.89	0.40	0.51
SAMPLE2_38.1	0.00	23.57	0.11	0.75	0.16	1.17	0.86	1.33	0.26	171.25	91.31	48.21	7.70	0.55	0.56
SAMPLE1_76.1	0.00	39.39	0.11	1.95	0.13	1.60	0.84	0.68	0.14	155.90	68.90	45.90	5.70	0.46	0.58
SAMPLE1_43.1	0.00	27.16	0.11	1.18	0.17	1.02	0.69	2.75	0.14	446.29	245.12	124.29	20.83	0.57	0.60
SAMPLE1_23.1	0.00	25.00	0.11	1.69	0.11	1.58	0.61	0.60	0.27	205.54	79.75	46.67	4.79	0.40	1.87
SAMPLE2_97.1	0.00	29.28	0.11	0.52	0.03	1.72	0.49	1.22	0.08	561.08	154.47	104.09	3.29	0.28	2.23
SAMPLE3_63.1	0.00	18.26	0.12	1.13	0.24	1.54	0.85	0.87	1.08	72.47	57.17	22.10	4.90	0.81	0.67
SAMPLE1_35.1	0.00	20.85	0.11	1.41	0.09	1.38	0.55	4.38	0.25	341.29	111.65	69.32	5.71	0.34	0.97
SAMPLE3_87.1	0.00	35.36	0.11	1.23	0.08	1.22	0.57	2.31	0.06	461.23	192.13	88.97	7.12	0.43	1.20
SAMPLE3_93.1	0.00	29.71	0.11	0.60	0.09	3.11	0.55	2.26	0.10	422.13	175.85	86.79	7.44	0.43	1.85
SAMPLE1_93.1	0.00	300.00	0.11	0.51	0.30	0.61	0.53	2.17	0.00	557.69	554.31	108.24	32.59	1.03	1.13
SAMPLE2_36.1	0.00	25.82	0.11	0.78	0.19	1.14	0.83	2.40	0.23	194.21	124.38	57.42	10.81	0.66	0.55
SAMPLE1_41.1	0.00	22.36	0.12	0.99	0.16	1.57	0.73	3.70	0.51	125.58	69.14	36.10	5.50	0.57	0.66
SAMPLE3_55.1	0.00	20.16	0.12	0.83	0.14	1.41	0.83	1.91	0.54	147.27	67.69	42.30	5.39	0.47	0.60
SAMPLE3_8.1	0.00	22.95	0.12	1.04	0.13	1.83	0.79	1.62	0.55	91.82	37.06	26.87	3.23	0.42	0.73
SAMPLE2_30.1	0.00	24.26	0.11	0.92	0.13	1.59	0.80	2.24	0.37	146.68	63.55	42.38	5.32	0.45	0.65
SAMPLE2_37.1	0.00	20.41	0.11	0.78	0.05	2.14	0.50	2.26	0.37	304.80	83.79	61.69	2.64	0.28	0.58
SAMPLE1_98.1	0.00	45.75	0.11	0.89	0.20	1.24	0.68	2.86	-0.10	129.80	85.71	34.17	7.14	0.68	1.08
SAMPLE2_89.1	0.00	42.43	0.11	1.16	0.43	1.20	0.68	0.81	0.20	80.04	109.78	18.38	7.90	1.42	0.52
SAMPLE1_33.1	0.00	86.60	0.11	0.79	0.08	1.64	0.44	5.47	0.02	336.93	61.75	58.97	4.99	0.19	2.53
SAMPLE2_59.1	0.00	20.00	0.12	1.02	0.14	1.72	0.87	2.24	0.69	98.65	44.93	28.22	3.63	0.47	0.69
SAMPLE1_14.1	0.00	17.87	0.11	1.54	0.13	2.53	0.83	4.36	0.52	186.75	79.57	55.86	6.65	0.44	0.63
SAMPLE2_46.1	0.00	20.85	0.11	1.38	0.16	3.46	0.81	3.53	0.34	194.66	103.01	56.10	8.73	0.55	0.56
SAMPLE1_83.1	0.00	21.57	0.11	0.61	0.12	2.17	0.50	2.13	0.40	419.15	175.73	81.22	9.24	0.43	1.09
SAMPLE3_24.1	0.00	21.82	0.11	0.60	0.13	1.94	0.48	0.87	0.18	446.78	185.35	71.97	9.40	0.43	1.01
SAMPLE3_59.1	0.00	18.58	0.12	1.37	0.20	2.06	0.81	2.41	1.56	46.92	27.97	13.53	2.25	0.62	0.82
SAMPLE3_4.1	0.00	18.90	0.12	1.04	0.17	1.63	0.80	2.85	0.81	94.70	51.17	27.28	4.15	0.56	0.68
SAMPLE2_54.1	0.00	21.94	0.11	0.98	0.17	1.53	0.65	1.48	0.52	147.33	82.17	27.82	4.41	0.58	0.60
SAMPLE1_78.1	0.00	45.23	0.11	0.98	0.12	1.73	0.71	2.62	0.10	140.21	60.66	37.89	4.66	0.45	0.56
SAMPLE3_35.1	0.00	20.86	0.12	1.27	0.42	1.34	0.85	1.97	1.01	57.99	81.31	17.36	7.08	1.45	0.64
SAMPLE2_45.1	0.00	30.16	0.12	1.74	0.19	2.58	0.84	2.30	0.90	37.09	21.97	10.64	1.88	0.61	0.97
SAMPLE1_64.1	0.00	46.70	0.11	2.70	0.09	4.99	0.51	1.95	0.40	478.57	278.67	77.19	6.27	0.60	2.84
SAMPLE3_39.1	0.00	22.95	0.12	1.49	0.19	2.21	0.76	1.08	1.16	41.64	23.98	11.76	1.99	0.59	0.86
SAMPLE1_84.1	0.00	20.56	0.11	1.15	0.07	2.21	0.38	1.45	0.17	711.96	182.79	122.21	8.62	0.27	0.49
SAMPLE1_73.1	0.00	24.26	0.11	1.10	0.11	3.82	0.79	1.51	0.55	91.90	32.69	26.14	2.57	0.37	0.73
SAMPLE3_36.1	0.00	22.94	0.11	0.69	0.06	1.73	0.50	1.81	0.23	329.58	70.45	57.51	3.12	0.22	0.56
SAMPLE1_3.1	0.00	35.36	0.11	1.68	0.16	4.72	0.59	2.31	0.59	72.66	37.31	21.85	3.36	0.53	0.92

Spot	(1) 206Pb/238U Age		(2) 206Pb/238U Age		(3) 206Pb/238U Age		(1) 207Pb/206Pb Age		preferred age		(1) 208Pb/232Th Age		(2) 208Pb/232Th Age		(3) 207Pb/206Pb Age		% Dis-cor-dant	7corr 208Pb*/232Th	±%	Total 238U/206Pb	±%
SAMPLE1_92.1	1895.58	23.30	1898.79	26.61	1895.33	24.32	1872.18	12.52	1872.18	12.52	1901.51	37.57	1979.88	96.73	1870.06	14.73	-1.44	0.10	5.13	2.93	1.42
SAMPLE1_96.1	1667.92	19.53	1645.85	21.22	1664.93	21.56	1871.87	9.29	1871.87	9.29	1711.58	77.28	1366.06	72.18	1843.45	31.93	12.36	0.07	5.46	3.39	1.33
SAMPLE2_27.1	1837.28	25.96	1832.83	29.22	1837.43	26.54	1871.79	13.00	1871.79	13.00	1829.79	55.58	1600.92	191.51	1873.08	12.79	2.12	0.08	12.44	3.03	1.62
SAMPLE1_40.1	1916.51	27.71	1922.85	31.82	1915.08	28.60	1871.23	13.20	1871.23	13.20	1964.08	46.77	2180.42	157.24	1859.30	15.10	-2.80	0.11	7.61	2.89	1.67
SAMPLE2_52.1	1933.47	27.57	1942.41	31.80	1931.64	29.98	1870.69	13.62	1870.69	13.62	1956.07	38.36	2075.57	71.89	1855.54	19.39	-3.89	0.11	3.64	2.86	1.65
SAMPLE2_55.1	1891.09	27.04	1893.93	30.84	1894.39	29.10	1870.26	13.34	1870.26	13.34	1844.90	36.96	1887.45	71.96	1897.75	17.56	-1.29	0.10	3.99	2.93	1.65
SAMPLE1_27.1	1772.64	24.87	1760.87	27.58	1772.54	25.35	1870.24	11.85	1870.24	11.85	1778.45	79.70	1080.23	195.29	1869.34	13.79	5.97	0.05	18.57	3.16	1.60
SAMPLE2_9.1	1884.42	27.07	1886.41	30.82	1888.15	28.95	1869.68	15.29	1869.68	15.29	1826.36	39.64	1859.43	77.98	1900.78	17.64	-0.91	0.10	4.39	2.94	1.66
SAMPLE3_86.1	1868.82	23.33	1868.85	26.59	1864.37	25.32	1868.58	21.96	1868.58	21.96	1925.60	34.72	1926.05	67.82	1830.14	25.45	-0.02	0.10	3.69	2.97	1.44
SAMPLE1_68.1	1798.89	27.66	1790.32	30.86	1800.67	28.67	1868.12	11.21	1868.12	11.21	1748.05	39.03	1492.68	112.24	1883.67	12.82	4.25	0.08	7.80	3.10	1.76
SAMPLE2_21.1	1848.57	36.92	1846.11	41.66	1849.84	39.86	1867.38	19.92	1867.38	19.92	1830.91	51.95	1794.28	91.44	1878.24	23.01	1.16	0.09	5.33	3.00	2.30
SAMPLE3_54.1	1893.31	26.29	1896.87	30.00	1894.16	28.17	1867.19	12.68	1867.19	12.68	1880.40	36.78	1938.06	72.97	1874.33	15.28	-1.61	0.10	3.95	2.92	1.60
SAMPLE1_6.1	1846.56	27.03	1843.90	30.51	1848.57	27.83	1867.03	12.57	1867.03	12.57	1775.21	43.09	1677.52	146.65	1884.19	13.78	1.26	0.09	9.11	3.01	1.68
SAMPLE3_5.1	1840.38	26.52	1836.94	29.89	1847.86	28.05	1867.01	15.38	1867.01	15.38	1700.70	41.72	1632.63	83.46	1929.92	17.00	1.64	0.08	5.32	3.02	1.66
SAMPLE3_11.1	1723.92	24.93	1707.61	27.40	1733.07	26.73	1866.33	13.53	1866.33	13.53	1590.65	32.81	1334.69	55.06	1947.25	16.38	8.69	0.07	4.26	3.26	1.65
SAMPLE3_42.1	1817.71	25.18	1811.59	28.22	1819.19	25.92	1866.19	9.86	1866.19	9.86	1762.73	52.37	1528.99	135.42	1879.11	13.25	2.98	0.08	9.20	3.07	1.59
SAMPLE2_58.1	1859.97	26.00	1859.21	29.43	1862.92	27.09	1865.75	11.37	1865.75	11.37	1786.45	37.27	1766.61	103.06	1890.71	13.01	0.36	0.09	6.09	2.99	1.61
SAMPLE2_20.1	1986.29	28.68	2004.43	33.57	1988.43	30.11	1865.72	16.95	1865.72	16.95	1938.97	50.32	2355.96	126.82	1882.87	17.19	-7.51	0.12	5.70	2.76	1.68
SAMPLE1_80.1	1873.45	42.47	1874.66	48.22	1875.05	43.63	1864.39	11.56	1864.39	11.56	1811.41	56.05	1859.60	243.48	1877.94	12.84	-0.56	0.10	13.70	2.96	2.61
SAMPLE1_71.1	1614.11	19.63	1588.55	21.71	1625.06	20.48	1864.32	45.94	1864.32	45.94	1337.57	53.90	652.51	150.42	1966.20	45.75	15.16	0.03	23.43	3.52	1.37
SAMPLE3_16.1	1791.53	25.67	1782.60	28.62	1791.71	26.49	1864.23	13.05	1864.23	13.05	1785.81	43.37	1477.91	123.17	1865.75	14.22	4.47	0.08	8.64	3.12	1.64
SAMPLE3_7.1	1876.72	27.40	1878.47	31.16	1878.54	28.86	1863.73	16.30	1863.73	16.30	1839.13	45.43	1876.90	98.48	1879.10	17.09	-0.80	0.10	5.49	2.95	1.68
SAMPLE1_39.1	1740.16	24.57	1725.84	27.09	1745.97	25.29	1863.18	11.59	1863.18	11.59	1530.48	41.95	989.68	116.39	1914.82	12.35	7.53	0.05	12.05	3.22	1.61
SAMPLE2_34.1	1847.47	26.08	1845.46	29.45	1849.72	26.93	1862.91	13.17	1862.91	13.17	1771.70	45.90	1701.90	133.98	1882.20	13.18	0.95	0.09	8.21	3.01	1.62
SAMPLE3_50.1	1905.75	67.66	1911.68	77.44	1900.08	76.14	1862.85	10.23	1862.85	10.23	1954.95	79.18	2012.26	126.15	1814.35	30.56	-2.66	0.10	6.59	2.90	4.10
SAMPLE1_63.1	1984.52	27.86	2002.80	32.60	1983.39	29.23	1862.68	10.98	1862.68	10.98	2010.10	60.17	2438.49	132.61	1853.49	19.28	-7.60	0.13	5.77	2.77	1.63
SAMPLE3_45.1	1774.00	24.99	1763.39	27.75	1776.61	25.71	1862.03	11.56	1862.03	11.56	1679.87	39.40	1282.61	124.15	1885.18	12.54	5.41	0.07	9.99	3.15	1.61
SAMPLE2_82.1	1749.79	20.76	1736.61	22.95	1752.66	21.59	1861.87	9.57	1861.87	9.57	1674.98	29.46	1314.79	74.34	1887.56	11.05	6.87	0.07	5.84	3.20	1.35
SAMPLE3_17.1	1862.53	25.83	1862.62	29.26	1859.83	27.17	1861.83	10.18	1861.83	10.18	1923.39	66.66	1925.58	108.65	1838.48	22.28	-0.04	0.10	5.92	2.98	1.60
SAMPLE2_42.1	1879.75	29.44	1882.19	33.53	1880.59	31.52	1861.62	21.37	1861.62	21.37	1866.86	49.54	1906.99	90.23	1868.69	23.88	-1.12	0.10	4.96	2.95	1.80
SAMPLE1_20.1	1482.63	23.19	1449.05	24.47	1487.00	23.52	1860.94	21.18	1860.94	21.18	1102.32	93.42	-2120.82	346.75	1905.83	20.37	22.73	-0.10	-15.51	3.85	1.75
SAMPLE3_44.1	1598.09	23.39	1571.74	25.12	1605.37	24.95	1860.71	14.59	1860.71	14.59	1483.27	32.12	1036.07	48.54	1930.07	16.95	15.92	0.05	4.81	3.55	1.65
SAMPLE3_83.1	1648.58	42.45	1626.16	45.96	1640.75	46.82	1860.56	11.05	1860.56	11.05	1732.00	62.05	1470.00	72.88	1783.50	36.34	12.90	0.08	5.14	3.43	2.92
SAMPLE2_6.1	1889.88	27.46	1893.89	31.35	1891.65	29.69	1860.42	15.87	1860.42	15.87	1866.30	39.74	1923.91	72.45	1875.25	19.60	-1.83	0.10	3.95	2.93	1.68
SAMPLE3_48.1	1887.66	26.90	1891.39	30.68	1889.47	27.88	1860.17	15.39	1860.17	15.39	1832.39	51.44	1950.06	136.20	1875.39	14.87	-1.71	0.10	7.33	2.93	1.64
SAMPLE2_17.1	1895.78	26.55	1900.80	30.49	1898.21	28.84	1859.05	24.48	1859.05	24.48	1865.60	34.96	1932.76	75.41	1879.49	26.58	-2.28	0.10	4.09	2.92	1.62
SAMPLE1_88.1	1867.23	22.10	1868.37	25.09	1868.94	22.96	1858.65	10.21	1858.65	10.21	1820.98	36.34	1852.85	97.55	1873.25	12.70	-0.53	0.10	5.51	2.97	1.36
SAMPLE2_95.1	1816.35	21.88	1811.04	24.56	1817.46	24.03	1858.55	11.14	1858.55	11.14	1802.89	48.63	1733.28	61.57	1868.29	28.65	2.61	0.09	3.71	3.07	1.38
SAMPLE3_73.1	1610.48	19.80	1585.28	21.32	1636.28	21.02	1858.49	11.50	1858.49	11.50	1201.99	22.06	770.25	40.54	2087.32	12.50	15.07	0.04	5.36	3.52	1.39
SAMPLE2_66.1	1827.12	21.92	1823.20	24.65	1832.19	23.82	1857.96	10.94	1857.96	10.94	1765.05	29.64	1712.90	50.13	1901.42	16.01	1.91	0.09	3.05	3.05	1.38
SAMPLE2_35.1	1919.36	27.62	1927.98	31.77	1917.50	29.87	1857.86	15.68	1857.86	15.68	1945.69	54.05	2075.70	87.12	1842.18	24.56	-3.83	0.11	4.42	2.88	1.66
SAMPLE3_67.1	1974.39	81.54	1991.75	95.16	1968.71	93.31	1857.35	18.42	1857.35	18.42	2017.25	95.45	2165.18	155.20	1810.04	43.23	-7.32	0.11	7.56	2.79	4.79
SAMPLE1_48.1	1894.74	27.20	1899.89	31.09	1895.96	29.27	1856.97	13.70	1856.97	13.70	1877.13	42.07	1956.24	76.72	1867.31	20.16	-2.35	0.10	4.11	2.92	1.66
SAMPLE1_100.1	1840.52	25.35	1838.48	28.63	1846.01	28.23	1856.32	17.83	1856.32	17.83	1787.62	36.32	1765.91	53.90	1903.15	24.06	0.98	0.09	3.19	3.02	1.58
SAMPLE3_38.1	1852.56	27.52	1852.30	31.15	1854.40	30.64	1854.55	17.46	1854.55	17.46	1834.61	38.49	1831.81	57.67	1870.40	24.00	0.12	0.09	3.29	3.00	1.71
SAMPLE1_44.1	1800.29	26.07	1793.58	29.14	1805.61	27.03	1854.48	14.21	1854.48	14.21	1644.17	43.67	1439.34	112.34	1900.84	14.02	3.35	0.07	8.09	3.10	1.66
SAMPLE3_72.1	1809.77	21.81	1804.22	24.44	1813.00	24.01	1854.21	11.80	1854.21	11.80	1775.36	29.04	1710.45	44.87	1882.36	17.05	2.75	0.09	2.74	3.08	1.38
SAMPLE3_95.1	1443.48	17.28	1408.64	18.12	1446.07	18.62	1853.54	10.84	1853.54	10.84	1409.01	23.04	905.62	27.17	1881.16	14.14	24.66	0.05	3.07	3.99	1.34
SAMPLE2_41.1	1947.68	28.44	1961.36	32.97	1948.88	31.11	1852.74	18.99	1852.74	18.99	1933.33	43.15	2110.16	75.25	1862.64	21.58	-5.94	0.11	3.76	2.82	1.69
SAMPLE1_25.1	1772.49	41.31	1763.00	45.88	1775.92	42.98	1851.46	17.50	1851.46	17.50	1681.07	58.69	1416.99	151.34	1882.00	18.35	4.88	0.07	11.06	3.15	2.66
SAMPLE2_51.1	1888.99	30.29	1894.12	34.61	1892.96	34.84	1851.18	24.91	1851.18	24.91	1859.24	44.10	1902.82	60.44	1884.53	36.83	-2.36	0.10	3.33	2.93	1.85
SAMPLE1_59.1	1738.06	25.19	1724.96	27.78	1740.78	27.11	1851.12	15.59	1851.12	15.59	1698.89	36.51	1496.36	57.37	1875.82	17.79	6.97	0.08	3.98	3.22	1.65
SAMPLE3_52.1	1523.02	22.80	1492.70	24.21	1448.53	27.80	1850.84	20.01	1850.84	20.01	1984.45	44.98	1767.30	43.71	698.09	161.92	19.87	0.09	2.58	3.74	1.68
SAMPLE1_77.1	1814.01	22.50	1809.41	25.26	1817.79	23.39	1850.67	13.25	1850.67	13.25	1717.24	38.68	1594.42	92.75	1883.62	15.04	2.27	0.08	6.05	3.08	1.42
SAMPLE1_58.1	1867.39	25.91	1869.64	29.42	1867.47	27.00	1850.40	9.63	1850.40	9.63	1865.15	36.02	1924.12	102.99	1851.17	11.50	-1.06	0.10	5.61	2.97	1.60
SAMPLE3_1.1	1886.72	26.69	1891.63	30.46	1894.17	29.21	1850.34	13.95	1850.34	13.95	1801.03	36.06	1862.53	61.88	1912.39	18.51	-2.27	0.10	3.48	2.93	1.63
SAMPLE1_50.1	1731.08	26.31	1717.39	29.01	1734.30	31.03	1850.13	18.63	1850.13	18.63	1710.88	34.95	1611.05	41.03	1879.39	37.85	7.33	0.08	2.65	3.24	1.73
SAMPLE1_81.1	1744.75	30.22	1732.48	33.39	1745.09	31.33	1849.93	12.78	1849.93	12.78	1734.79	60.63	1361.80	122.62	1853.05	18.86	6.49	0.07	9.31	3.22	1.98
SAMPLE1_15.1	1926.08	37.03	1936.91	42.66	1931.07	40.33	1849.14	17.80	1849.14	17.80	1864.95	50.41	2008.18	91.29	1890.37	21.61	-4.81	0.10	4.78	2.86	2.22
SAMPLE2_65.1	1832.46	27.70	1830.43	31.20	1835.62	29.07	1848.40	20.38	1848.40	20.38	1761.23	53.82	1713.15	104.55	1875.79	19.05	0.99	0.09	6.36	3.03	1.73
SAMPLE1_10.1	1883.88	28.04	1888.69	32.00	1885.33	29.22	1848.15	15.41	1848.15	15.41	1846.57	47.42	1974.23	122.51	1860.57	16.77	-2.23	0.10	6.51	2.94	1.72
SAMPLE2_47.1	1884.98	27.09	1889.97	30.90	1893.18	29.49	1847.96	15.60	1847.96	15.60	1784.34	37.17	1850.66	65.47	1916.23	18.29	-2.31	0.10	3.70	2.93	1.66
SAMPLE3_100.1	1917.93	39.47	1927.71	45.40	1917.24	42.38	1847.85	11.93	1847.85	11.93	1927.94	49.73	2079.06	106.73	1842.01	18.45	-4.39	0.11	5.40	2.88	2.38
SAMPLE1_29.1	1727.59	25.51	1713.91	28.10	1734.39	25.82	1847.09	10.24	1847.09	10.24	1189.34	36.26	47.69	259.37	1908.35	10.54	7.37	0.00	544.53	3.25	1.68
SAMPLE1_74.1	1837.36	24.21	1836.15	27.35	1839.88	25.93	1846.77	17.29	1846.77	17.29	1799.02	39.07	1779.37	70.57	1868.59	20.18	0.59	0.09	4.14	3.03	1.51
SAMPLE3_57.1	1919.19	28.28	1929.34	32.58	1919.95	30.26	1846.54	17.27	1846.54	17.27	1907.34	43.79	2075.13	88.25	1852.93	20.17	-4.55	0.11	4.47	2.88	1.70
SAMPLE2_24.1	1868.69	26.33	1871.74	29.91	1870.30	29.60	1845.69	14.43	1845.69	14.43	1854.26	37.18	1884.66	53.04	1859.50	23.79	-1.44	0.10	2.95	2.96	1.62
SAMPLE3_77.1	1846.49	24.75	1846.62	28.03	1848.77	26.82	1845.50	17.33	1845.50	17.33	1817.73	38.12	1819.48	64.21	1865.23	21.88	-0.06	0.09	3.69	3.02	1.54
SAMPLE3_15.1	1829.39	26.68	1827.41	30.06	1826.72	28.52	1844.96	15.22	1844.96	15.22	1871.82	43.16	1838.36	76.57	1821.26	20.81	0.97	0.10	4.36	3.04	1.67
SAMPLE2_62.1	1875.98	39.72	1880.17	45.21	1881.28	42.87	1844.61	15.04	1844.61	15.04	1802.24	58.65	1864.79	103.79	1889.39	25.81	-1.96	0.10	5.83	2.95	2.44
SAMPLE2_18.1	1894.08	27.11	1900.89	31.04	1895.68	29.35	1844.06	14.97	1844.06	14.97	1873.48	37.23	1968.33	70.73	1857.57	19.60	-3.13	0.10	3.77	2.92	1.65
SAMPLE3_46.1	1685.85	37.17	1668.70	40.62	1688.52	39.45	1842.33	20.51	1842.33	20.51	1639.12	52.17	1319.90	90.66	1867.37	23.86	9.65	0.07	7.10	3.34	2.50
SAMPLE2_8.1	1877.47	28.74	1882.43	32.78	1880.03	31.42	1840.34	20.21	1840.34	20.21	1846.97	46.43	1911.05	74.25	1862.22	26.53	-2.33	0.10	4.07	2.95	1.76
SAMPLE2_7.1	1889.67	26.90	1896.56	30.76	1891.35	27.94	1838.74	13.77	1838.74	13.77	1842.40	44.74	2041.58	126.03	1853.15	14.55	-3.20	0.11	6.49	2.93	1.64
SAMPLE3_64.1	1884.78	37.28	1891.02	42.55	1889.33	38.86	1838.47	19.49	1838.47	19.49	1763.40	63.09	1936.03	163.14	1876.98	18.04	-2.91	0.10	8.84	2.93	2.28
SAMPLE3_21.1	1846.38	27.55	1847.44	31.18	1846.95	29.15	1838.16	17.65	1838.16	17.65	1835.53	45.70	1856.65	91.40	1843.19	19.36	-0.51	0.10	5.15	3.01	1.71
SAMPLE2_33.1	1885.67	34.09	1892.07	38.86	1880.02	42.79	1838.15	40.83	1838.15	40.83	1917.38	79.87	1959.66	87.26	1788.54	187.32	-2.98	0.10	4.67	2.92	2.07
SAMPLE1_52.1	1885.94	28.56	1892.40	32.63	1886.00	31.06	1838.04	20.58	1838.04	20.58	1885.24	45.12	1973.83	75.49	1838.51	23.36	-3.01	0.10	4.01	2.93	1.74
SAMPLE3_68.1	1907.83	23.51	1917.46	27.03	1905.57	24.55	1837.99	13.73	1837.99	13.73	1970.31	80.69	2244.15	140.22	1818.59	25.38	-4.39	0.12	6.60	2.90	1.42
SAMPLE3_33.1	997.91	14.40	955.94	14.32	1007.12	15.24	1837.70	10.07	1837.70	10.07	833.83	19.52	27.36	19.70	1970.44	13.03	49.24	0.00	72.07	5.96	1.56
SAMPLE2_61.1	1848.81	28.64	1850.39	32.43	1855.01	29.46	1836.60	16.17	1836.60	16.17	1619.44	54.23	1679.81	165.24	1889.79	15.41	-0.76	0.09	10.25	3.00	1.78
SAMPLE3_94.1	1905.71	23.70	1915.26	27.25	1913.27	25.98	1836.30	14.02	1836.30	14.02	1819.51	37.67	1937.82	61.76	1899.25	22.64	-4.37	0.10	3.34	2.90	1.44
SAMPLE3_76.1	1499.57	18.19	1469.40	19.26	1507.89	20.62	1835.24	10.88	1835.24	10.88	1432.93	22.23	1159.43	24.19	1919.90	18.21	20.48	0.06	2.15	3.82	1.36
SAMPLE2_90.1	1665.29	20.67	1647.10	22.52	1682.64	21.52	1835.14	14.22	1835.14	14.22	1224.32	43.45	733.89	73.37	1991.54	15.65	10.50	0.04	10.18	3.39	1.41
SAMPLE2_39.1	1860.43	27.34	1863.90	31.05	1868.47	29.69	1833.95	15.96	1833.95	15.96	1759.90	37.48	1806.85	65.86	1902.23	19.03	-1.66	0.09	3.81	2.98	1.69
SAMPLE3_37.1	1720.80	29.31	1708.00	32.35	1721.27	32.52	1833.69	26.29	1833.69	26.29	1716.08	46.66	1572.64	62.99	1838.02	34.31	7.01	0.08	4.16	3.26	1.94
SAMPLE3_13.1	1828.03	26.95	1827.38	30.38	1828.01	28.13	1833.19	18.46	1833.19	18.46	1828.64	52.64	1811.37	112.37	1832.97	18.37	0.32	0.09	6.49	3.04	1.69
SAMPLE3_2.1	1799.20	25.29	1795.02	28.41	1807.72	26.10	1833.14	23.09	1833.14	23.09	1522.63	39.04	1381.59	143.76	1907.49	21.85	2.12	0.07	10.77	3.10	1.61
SAMPLE1_95.1	1450.84	29.86	1418.36	31.37	1454.86	31.05	1832.00	9.41	1832.00	9.41	1348.74	33.61	472.50	65.04	1874.95	11.75	23.21	0.02	13.93	3.96	2.30
SAMPLE2_32.1	1868.36	27.68	1873.24	31.49	1872.63	29.20	1831.48	19.35	1831.48	19.35	1782.19	49.36	1885.41	99.19	1868.13	18.59	-2.32	0.10	5.51	2.96	1.70
SAMPLE3_26.1	1485.44	21.14	1454.95	22.38	1489.92	22.25	1830.18	18.18	1830.18	18.18	1395.45	27.82	743.53	53.50	1877.12	18.77	21.07	0.04	7.33	3.85	1.59
SAMPLE2_38.1	1827.38	28.22	1827.10	31.82	1828.01	30.33	1829.59	16.27	1829.59	16.27	1818.12	41.06	1813.74	73.48	1835.20	19.51	0.14	0.09	4.24	3.04	1.77
SAMPLE1_76.1	1899.78	25.89	1909.53	30.05	1906.11	27.41	1828.31	36.64	1828.31	36.64	1785.25	43.07	1971.04	122.96	1881.67	35.56	-4.51	0.10	6.55	2.91	1.57
SAMPLE1_43.1	1810.30	27.03	1808.15	30.44	1808.81	29.10	1827.64	22.43	1827.64	22.43	1831.45	37.63	1798.80	74.75	1814.15	25.24	1.09	0.09	4.34	3.08	1.71
SAMPLE1_23.1	1512.03	42.91	1483.35	45.60	1522.06	45.20	1827.51	32.75	1827.51	32.75	1312.47	54.51	702.37	103.83	1928.66	33.85	19.35	0.04	15.04	3.77	3.18
SAMPLE2_97.1	1260.55	18.48	1221.67	18.94	1288.49	19.09	1825.53	10.08	1825.53	10.08	474.36	17.13	-709.49	50.15	2130.47	12.74	34.02	-0.03	-6.94	4.63	1.61
SAMPLE3_63.1	1958.63	32.21	1978.02	37.46	1970.15	36.01	1824.92	36.22	1824.92	36.22	1846.29	57.61	2054.51	80.96	1918.12	31.69	-8.50	0.11	4.14	2.79	1.90
SAMPLE1_35.1	1368.16	20.10	1332.72	21.00	1378.69	21.00	1824.25	27.19	1824.25	27.19	1121.17	30.03	233.24	69.78	1940.00	25.33	27.71	0.01	30.09	4.22	1.63
SAMPLE3_87.1	1305.89	15.76	1268.35	16.35	1332.61	16.61	1824.08	22.69	1824.08	22.69	819.71	18.20	79.87	43.47	2109.12	20.31	31.34	0.00	54.54	4.45	1.33
SAMPLE3_93.1	1383.28	37.58	1348.48	39.11	1407.86	39.61	1823.77	11.79	1823.77	11.79	932.42	43.60	243.03	57.90	2076.43	21.65	26.80	0.01	23.97	4.17	3.02
SAMPLE1_93.1	1313.24	19.12	1276.08	19.72	1317.36	22.16	1822.12	9.26	1822.12	9.26	1284.63	25.66	987.65	22.70	1870.41	29.30	30.83	0.05	2.36	4.43	1.61
SAMPLE2_36.1	1906.96	27.84	1918.65	32.04	1909.87	30.34	1821.79	16.79	1821.79	16.79	1871.60	39.90	2024.94	71.52	1846.62	21.07	-5.40	0.11	3.71	2.90	1.69
SAMPLE1_41.1	1861.05	29.13	1866.24	33.13	1871.05	31.38	1821.43	24.90	1821.43	24.90	1718.48	49.66	1797.71	82.52	1906.44	23.54	-2.50	0.09	4.80	2.97	1.80
SAMPLE3_55.1	1859.68	27.63	1864.80	31.39	1867.78	29.40	1820.56	22.24	1820.56	22.24	1719.91	49.95	1813.42	87.16	1889.88	19.48	-2.47	0.09	5.03	2.97	1.71
SAMPLE3_8.1	1890.17	30.04	1899.76	34.40	1890.97	31.76	1819.12	26.92	1819.12	26.92	1874.37	64.88	2072.87	116.07	1826.04	24.42	-4.51	0.11	5.89	2.92	1.83
SAMPLE2_30.1	1868.96	28.60	1876.06	32.62	1872.36	30.32	1815.10	21.60	1815.10	21.60	1806.58	51.33	1943.74	98.34	1844.66	21.49	-3.42	0.10	5.31	2.96	1.76
SAMPLE2_37.1	1363.83	19.93	1329.30	20.72	1387.28	20.64	1811.76	18.25	1811.76	18.25	698.47	33.82	-355.73	62.43	2058.37	13.64	27.40	-0.02	-17.40	4.23	1.62
SAMPLE1_98.1	1723.20	22.66	1713.53	25.05	1716.79	24.85	1808.71	17.16	1808.71	17.16	1796.67	39.12	1676.19	52.61	1746.37	26.88	5.39	0.09	3.27	3.27	1.50
SAMPLE2_89.1	1527.48	22.27	1501.68	23.78	1520.33	27.67	1807.83	24.63	1807.83	24.63	1562.36	32.15	1410.56	33.00	1730.05	87.27	17.40	0.07	2.42	3.73	1.64
SAMPLE1_33.1	1195.47	35.94	1156.67	36.59	1181.99	36.94	1806.56	14.53	1806.56	14.53	1746.48	75.09	79.77	95.31	1615.57	22.48	37.01	0.00	119.73	4.91	3.29
SAMPLE2_59.1	1853.05	29.53	1859.25	33.54	1859.16	31.46	1805.10	28.09	1805.10	28.09	1746.72	60.18	1860.77	97.68	1858.51	23.96	-3.06	0.10	5.49	2.98	1.83
SAMPLE1_14.1	1926.09	63.65	1943.14	73.76	1932.68	67.47	1803.94	32.28	1803.94	32.28	1802.13	85.49	2138.52	227.69	1859.59	35.87	-7.84	0.11	11.22	2.86	3.82
SAMPLE2_46.1	1864.96	40.86	1873.11	46.65	1867.59	44.05	1802.70	27.81	1802.70	27.81	1825.92	77.57	1954.59	130.77	1825.90	38.30	-3.98	0.10	7.02	2.97	2.52
SAMPLE1_83.1	1311.37	27.34	1276.11	28.22	1320.22	29.01	1798.03	16.91	1798.03	16.91	1153.20	42.15	475.71	42.68	1901.22	20.88	29.87	0.02	9.08	4.42	2.30
SAMPLE3_24.1	1108.00	16.07	1068.88	16.22	1107.73	17.09	1795.13	12.61	1795.13	12.61	1112.88	30.14	374.55	26.74	1791.19	20.11	41.60	0.02	7.21	5.32	1.58
SAMPLE3_59.1	1866.53	33.82	1876.03	38.37	1876.40	36.87	1793.96	51.37	1793.96	51.37	1737.61	84.23	1871.23	99.02	1879.11	34.73	-4.66	0.10	5.54	2.93	2.07
SAMPLE3_4.1	1864.43	29.83	1873.86	34.00	1872.19	32.17	1792.22	30.13	1792.22	30.13	1751.88	57.68	1898.02	87.96	1859.78	25.49	-4.64	0.10	4.86	2.96	1.84
SAMPLE2_54.1	1281.01	20.29	1245.30	20.88	1289.00	21.99	1791.91	25.08	1791.91	25.08	1176.15	33.36	665.77	32.85	1887.78	23.29	31.40	0.03	5.02	4.52	1.74
SAMPLE1_78.1	1763.07	23.18	1759.70	25.90	1768.57	24.55	1791.76	19.09	1791.76	19.09	1663.33	39.85	1598.56	75.59	1842.34	21.11	1.83	0.08	4.92	3.18	1.50
SAMPLE3_35.1	1927.45	33.28	1947.10	38.53	1938.00	41.12	1786.40	39.82	1786.40	39.82	1874.65	49.24	1992.58	59.54	1875.23	88.99	-9.14	0.10	3.14	2.84	1.99
SAMPLE2_45.1	1857.75	37.56	1867.16	42.72	1859.08	40.87	1785.06	52.51	1785.06	52.51	1840.33	88.69	1972.61	115.88	1797.00	43.26	-4.69	0.10	6.17	2.97	2.31
SAMPLE1_64.1	1109.33	35.51	1071.06	35.97	1158.08	38.31	1782.49	57.99	1782.49	57.99	501.48	42.38	-25.84	61.12	2333.23	44.00	41.05	0.00	-236.39	5.30	3.48
SAMPLE3_39.1	1832.49	33.92	1838.90	38.29	1835.68	36.85	1781.63	49.57	1781.63	49.57	1789.49	82.68	1882.07	101.22	1810.51	37.48	-3.28	0.10	5.63	3.01	2.11
SAMPLE1_84.1	1174.43	19.80	1137.51	20.19	1179.13	20.52	1775.32	21.83	1775.32	21.83	1036.86	31.55	-115.16	64.55	1837.72	22.20	36.97	-0.01	-55.89	5.00	1.84
SAMPLE1_73.1	1843.83	45.67	1852.86	51.92	1850.34	48.03	1772.89	28.80	1772.89	28.80	1697.46	93.31	1909.30	179.33	1831.04	31.55	-4.60	0.10	9.84	3.00	2.84
SAMPLE3_36.1	1192.10	17.40	1155.83	17.75	1198.22	17.92	1770.69	15.03	1770.69	15.03	974.76	31.07	-399.50	57.20	1850.56	13.82	35.74	-0.02	-14.18	4.91	1.60
SAMPLE1_3.1	1934.65	35.86	1957.68	41.76	1934.68	38.85	1770.12	44.61	1770.12	44.61	1934.28	112.75	2308.18	161.46	1770.33	44.12	-10.77	0.12	7.40	2.84	2.14

Spot	Total 207Pb/206Pb	±%	(1) 238U/206Pb*	±%	(1) 207Pb*/206Pb*	±%	(1) 207Pb*/235U	±%	(1) 206Pb*/238U	±%	err corr	(3) 238U/206Pb*	±%	(3) 207Pb*/206Pb*	±%	(3) 207Pb*/235U	±%	(3) 206Pb*/238U	±%	err. corr.
SAMPLE1_92.1	0.11	0.67	2.93	1.42	0.11	0.69	5.40	1.58	0.34	1.42	0.90	2.93	1.48	0.11	0.82	5.39	1.53	0.34	1.48	0.85
SAMPLE1_96.1	0.11	0.51	3.39	1.33	0.11	0.52	4.66	1.43	0.30	1.33	0.93	3.39	1.47	0.11	1.76	4.58	2.18	0.29	1.47	0.59
SAMPLE2_27.1	0.12	0.62	3.03	1.62	0.11	0.72	5.21	1.78	0.33	1.62	0.91	3.03	1.66	0.11	0.71	5.21	1.72	0.33	1.66	0.91
SAMPLE1_40.1	0.11	0.70	2.89	1.67	0.11	0.73	5.46	1.82	0.35	1.67	0.92	2.89	1.73	0.11	0.84	5.42	1.79	0.35	1.73	0.89
SAMPLE2_52.1	0.12	0.70	2.86	1.65	0.11	0.75	5.52	1.81	0.35	1.65	0.91	2.86	1.80	0.11	1.07	5.47	1.77	0.35	1.80	0.82
SAMPLE2_55.1	0.12	0.68	2.93	1.65	0.11	0.74	5.38	1.81	0.34	1.65	0.91	2.93	1.77	0.12	0.98	5.47	1.74	0.34	1.77	0.85
SAMPLE1_27.1	0.12	0.56	3.16	1.60	0.11	0.66	4.99	1.73	0.32	1.60	0.93	3.16	1.64	0.11	0.76	4.99	1.68	0.32	1.64	0.89
SAMPLE2_9.1	0.12	0.71	2.95	1.66	0.11	0.85	5.35	1.86	0.34	1.66	0.89	2.94	1.77	0.12	0.98	5.46	1.76	0.34	1.77	0.85
SAMPLE3_86.1	0.11	1.19	2.97	1.44	0.11	1.22	5.30	1.88	0.34	1.44	0.76	2.98	1.56	0.11	1.40	5.17	1.88	0.34	1.56	0.68
SAMPLE1_68.1	0.11	0.58	3.11	1.76	0.11	0.62	5.07	1.87	0.32	1.76	0.94	3.10	1.82	0.12	0.71	5.12	1.81	0.32	1.82	0.92
SAMPLE2_21.1	0.12	0.85	3.01	2.30	0.11	1.10	5.23	2.55	0.33	2.30	0.90	3.01	2.48	0.11	1.28	5.27	2.38	0.33	2.48	0.86
SAMPLE3_54.1	0.12	0.58	2.93	1.60	0.11	0.70	5.38	1.75	0.34	1.60	0.92	2.93	1.72	0.11	0.85	5.40	1.66	0.34	1.72	0.87
SAMPLE1_6.1	0.11	0.66	3.01	1.68	0.11	0.70	5.22	1.82	0.33	1.68	0.92	3.01	1.73	0.12	0.76	5.28	1.78	0.33	1.73	0.91
SAMPLE3_5.1	0.12	0.70	3.03	1.66	0.11	0.85	5.20	1.86	0.33	1.66	0.89	3.01	1.75	0.12	0.95	5.41	1.79	0.33	1.75	0.86
SAMPLE3_11.1	0.12	0.68	3.26	1.65	0.11	0.75	4.83	1.81	0.31	1.65	0.91	3.24	1.76	0.12	0.92	5.08	1.73	0.31	1.76	0.86
SAMPLE3_42.1	0.12	0.50	3.07	1.59	0.11	0.55	5.13	1.68	0.33	1.59	0.95	3.07	1.64	0.11	0.74	5.17	1.64	0.33	1.64	0.90
SAMPLE2_58.1	0.12	0.58	2.99	1.61	0.11	0.63	5.26	1.73	0.33	1.61	0.93	2.98	1.67	0.12	0.72	5.35	1.67	0.34	1.67	0.91
SAMPLE2_20.1	0.12	0.75	2.77	1.68	0.11	0.94	5.68	1.92	0.36	1.68	0.87	2.77	1.76	0.12	0.95	5.74	1.80	0.36	1.76	0.86
SAMPLE1_80.1	0.12	0.59	2.97	2.61	0.11	0.64	5.30	2.69	0.34	2.61	0.97	2.96	2.68	0.11	0.71	5.35	2.62	0.34	2.68	0.96
SAMPLE1_71.1	0.11	2.55	3.51	1.37	0.11	2.54	4.47	2.89	0.28	1.37	0.48	3.49	1.43	0.12	2.57	4.77	2.94	0.29	1.43	0.49
SAMPLE3_16.1	0.12	0.67	3.12	1.64	0.11	0.72	5.04	1.79	0.32	1.64	0.92	3.12	1.69	0.11	0.79	5.04	1.74	0.32	1.69	0.90
SAMPLE3_7.1	0.12	0.73	2.96	1.68	0.11	0.90	5.31	1.91	0.34	1.68	0.88	2.96	1.77	0.11	0.95	5.36	1.80	0.34	1.77	0.86
SAMPLE1_39.1	0.12	0.56	3.23	1.61	0.11	0.64	4.87	1.73	0.31	1.61	0.93	3.21	1.65	0.12	0.69	5.03	1.70	0.31	1.65	0.92
SAMPLE2_34.1	0.12	0.62	3.01	1.62	0.11	0.73	5.21	1.78	0.33	1.62	0.91	3.01	1.67	0.12	0.73	5.28	1.71	0.33	1.67	0.91
SAMPLE3_50.1	0.11	0.53	2.91	4.10	0.11	0.57	5.40	4.14	0.34	4.10	0.99	2.92	4.63	0.11	1.68	5.24	3.89	0.34	4.63	0.94
SAMPLE1_63.1	0.11	0.58	2.77	1.63	0.11	0.61	5.66	1.74	0.36	1.63	0.94	2.78	1.71	0.11	1.07	5.63	1.75	0.36	1.71	0.81
SAMPLE3_45.1	0.11	0.60	3.16	1.61	0.11	0.64	4.97	1.73	0.32	1.61	0.93	3.15	1.66	0.12	0.70	5.05	1.69	0.32	1.66	0.91
SAMPLE2_82.1	0.11	0.50	3.21	1.35	0.11	0.53	4.90	1.45	0.31	1.35	0.93	3.20	1.41	0.12	0.61	4.98	1.41	0.31	1.41	0.91
SAMPLE3_17.1	0.11	0.52	2.99	1.60	0.11	0.56	5.26	1.69	0.33	1.60	0.94	2.99	1.68	0.11	1.23	5.18	1.90	0.33	1.68	0.77
SAMPLE2_42.1	0.12	0.99	2.95	1.81	0.11	1.18	5.31	2.16	0.34	1.81	0.84	2.95	1.93	0.11	1.32	5.34	2.04	0.34	1.93	0.78
SAMPLE1_20.1	0.12	1.05	3.87	1.75	0.11	1.17	4.06	2.11	0.26	1.75	0.83	3.85	1.77	0.12	1.13	4.17	2.05	0.26	1.77	0.83
SAMPLE3_44.1	0.12	0.73	3.55	1.65	0.11	0.81	4.41	1.84	0.28	1.65	0.90	3.54	1.76	0.12	0.95	4.61	1.76	0.28	1.76	0.86
SAMPLE3_83.1	0.11	0.59	3.43	2.92	0.11	0.61	4.57	2.98	0.29	2.92	0.98	3.45	3.23	0.11	1.99	4.36	3.07	0.29	3.23	0.80
SAMPLE2_6.1	0.12	0.75	2.94	1.68	0.11	0.88	5.34	1.89	0.34	1.68	0.89	2.93	1.81	0.11	1.09	5.39	1.81	0.34	1.81	0.82
SAMPLE3_48.1	0.12	0.68	2.94	1.64	0.11	0.85	5.34	1.85	0.34	1.64	0.89	2.94	1.70	0.11	0.82	5.39	1.75	0.34	1.70	0.89
SAMPLE2_17.1	0.11	1.33	2.92	1.62	0.11	1.36	5.36	2.11	0.34	1.62	0.77	2.92	1.75	0.11	1.47	5.43	2.06	0.34	1.75	0.71
SAMPLE1_88.1	0.11	0.54	2.98	1.36	0.11	0.57	5.26	1.48	0.34	1.36	0.92	2.97	1.42	0.11	0.70	5.31	1.47	0.34	1.42	0.88
SAMPLE2_95.1	0.11	0.61	3.07	1.38	0.11	0.62	5.10	1.51	0.33	1.38	0.91	3.07	1.52	0.11	1.59	5.13	2.09	0.33	1.52	0.65
SAMPLE3_73.1	0.11	0.63	3.52	1.39	0.11	0.64	4.45	1.53	0.28	1.39	0.91	3.46	1.45	0.13	0.71	5.15	1.46	0.29	1.45	0.88
SAMPLE2_66.1	0.11	0.59	3.05	1.38	0.11	0.61	5.13	1.50	0.33	1.38	0.92	3.04	1.49	0.12	0.89	5.28	1.48	0.33	1.49	0.82
SAMPLE2_35.1	0.12	0.74	2.88	1.66	0.11	0.87	5.43	1.88	0.35	1.66	0.89	2.89	1.80	0.11	1.36	5.38	1.85	0.35	1.80	0.72
SAMPLE3_67.1	0.11	0.99	2.79	4.79	0.11	1.02	5.61	4.90	0.36	4.79	0.98	2.80	5.50	0.11	2.38	5.45	4.68	0.36	5.50	0.90
SAMPLE1_48.1	0.12	0.67	2.93	1.66	0.11	0.76	5.35	1.82	0.34	1.66	0.91	2.92	1.78	0.11	1.12	5.38	1.87	0.34	1.78	0.81
SAMPLE1_100.1	0.11	0.87	3.03	1.58	0.11	0.99	5.17	1.87	0.33	1.58	0.85	3.02	1.76	0.12	1.34	5.33	1.91	0.33	1.76	0.74
SAMPLE3_38.1	0.12	0.82	3.00	1.71	0.11	0.97	5.21	1.96	0.33	1.71	0.87	3.00	1.90	0.11	1.33	5.26	1.97	0.33	1.90	0.76
SAMPLE1_44.1	0.12	0.66	3.10	1.66	0.11	0.79	5.04	1.84	0.32	1.66	0.90	3.09	1.72	0.12	0.78	5.19	1.75	0.32	1.72	0.90
SAMPLE3_72.1	0.11	0.61	3.09	1.38	0.11	0.65	5.07	1.53	0.32	1.38	0.90	3.08	1.52	0.12	0.95	5.16	1.51	0.32	1.52	0.80
SAMPLE3_95.1	0.11	0.56	3.98	1.34	0.11	0.60	3.92	1.46	0.25	1.34	0.91	3.98	1.44	0.12	0.78	3.99	1.42	0.25	1.44	0.85
SAMPLE2_41.1	0.12	0.78	2.83	1.69	0.11	1.05	5.51	1.99	0.35	1.69	0.85	2.83	1.85	0.11	1.20	5.54	1.86	0.35	1.85	0.79
SAMPLE1_25.1	0.12	0.81	3.16	2.67	0.11	0.97	4.94	2.84	0.32	2.67	0.94	3.15	2.77	0.12	1.02	5.04	2.72	0.32	2.77	0.93
SAMPLE2_51.1	0.12	1.09	2.94	1.85	0.11	1.38	5.31	2.31	0.34	1.85	0.80	2.93	2.12	0.12	2.05	5.43	2.74	0.34	2.12	0.67
SAMPLE1_59.1	0.12	0.69	3.23	1.65	0.11	0.86	4.83	1.86	0.31	1.65	0.89	3.23	1.78	0.11	0.99	4.90	1.76	0.31	1.78	0.84
SAMPLE3_52.1	0.12	0.91	3.75	1.68	0.11	1.11	4.16	2.01	0.27	1.68	0.84	3.97	2.14	0.06	7.60	2.18	8.23	0.25	2.14	0.41
SAMPLE1_77.1	0.11	0.70	3.08	1.42	0.11	0.73	5.07	1.60	0.32	1.42	0.89	3.07	1.48	0.12	0.84	5.18	1.55	0.33	1.48	0.85
SAMPLE1_58.1	0.11	0.49	2.98	1.60	0.11	0.53	5.24	1.68	0.34	1.60	0.95	2.98	1.67	0.11	0.64	5.24	1.63	0.34	1.67	0.93
SAMPLE3_1.1	0.12	0.64	2.94	1.63	0.11	0.77	5.30	1.80	0.34	1.63	0.90	2.93	1.78	0.12	1.03	5.51	1.73	0.34	1.78	0.83
SAMPLE1_50.1	0.11	0.89	3.25	1.73	0.11	1.03	4.80	2.02	0.31	1.73	0.86	3.24	2.04	0.11	2.10	4.89	2.87	0.31	2.04	0.68
SAMPLE1_81.1	0.11	0.67	3.22	1.98	0.11	0.71	4.85	2.10	0.31	1.98	0.94	3.22	2.05	0.11	1.04	4.86	2.05	0.31	2.05	0.87
SAMPLE1_15.1	0.12	0.67	2.87	2.22	0.11	0.98	5.43	2.43	0.35	2.22	0.91	2.86	2.42	0.12	1.20	5.57	2.31	0.35	2.42	0.87
SAMPLE2_65.1	0.12	0.84	3.04	1.74	0.11	1.13	5.12	2.07	0.33	1.74	0.84	3.04	1.82	0.11	1.06	5.21	1.90	0.33	1.82	0.84
SAMPLE1_10.1	0.11	0.75	2.95	1.72	0.11	0.85	5.29	1.92	0.34	1.72	0.90	2.94	1.79	0.11	0.93	5.33	1.84	0.34	1.79	0.87
SAMPLE2_47.1	0.12	0.68	2.94	1.66	0.11	0.86	5.29	1.87	0.34	1.66	0.89	2.93	1.80	0.12	1.02	5.52	1.76	0.34	1.80	0.84
SAMPLE3_100.1	0.11	0.59	2.89	2.38	0.11	0.66	5.40	2.47	0.35	2.38	0.96	2.89	2.56	0.11	1.02	5.38	2.37	0.35	2.56	0.92
SAMPLE1_29.1	0.11	0.56	3.25	1.68	0.11	0.57	4.79	1.78	0.31	1.68	0.95	3.24	1.70	0.12	0.59	4.97	1.76	0.31	1.70	0.94
SAMPLE1_74.1	0.11	0.86	3.03	1.51	0.11	0.96	5.13	1.79	0.33	1.51	0.85	3.03	1.62	0.11	1.12	5.20	1.72	0.33	1.62	0.78
SAMPLE3_57.1	0.11	0.81	2.88	1.70	0.11	0.95	5.40	1.95	0.35	1.70	0.87	2.88	1.82	0.11	1.12	5.42	1.85	0.35	1.82	0.82
SAMPLE2_24.1	0.12	0.62	2.97	1.62	0.11	0.80	5.23	1.81	0.34	1.62	0.90	2.97	1.82	0.11	1.32	5.28	1.97	0.34	1.82	0.76
SAMPLE3_77.1	0.11	0.90	3.02	1.54	0.11	0.96	5.16	1.82	0.33	1.54	0.85	3.01	1.67	0.11	1.21	5.22	1.78	0.33	1.67	0.75
SAMPLE3_15.1	0.11	0.76	3.05	1.68	0.11	0.84	5.10	1.87	0.33	1.68	0.89	3.05	1.79	0.11	1.15	5.03	1.89	0.33	1.79	0.81
SAMPLE2_62.1	0.12	0.67	2.96	2.44	0.11	0.83	5.25	2.58	0.34	2.44	0.95	2.95	2.63	0.12	1.43	5.40	2.46	0.34	2.63	0.84
SAMPLE2_18.1	0.11	0.77	2.93	1.65	0.11	0.83	5.31	1.85	0.34	1.65	0.89	2.93	1.79	0.11	1.09	5.35	1.79	0.34	1.79	0.82
SAMPLE3_46.1	0.11	0.99	3.35	2.51	0.11	1.13	4.64	2.75	0.30	2.51	0.91	3.34	2.66	0.11	1.32	4.72	2.62	0.30	2.66	0.87
SAMPLE2_8.1	0.11	0.93	2.96	1.76	0.11	1.12	5.24	2.09	0.34	1.76	0.85	2.95	1.93	0.11	1.47	5.32	2.13	0.34	1.93	0.74
SAMPLE2_7.1	0.11	0.66	2.94	1.64	0.11	0.76	5.28	1.81	0.34	1.64	0.91	2.93	1.70	0.11	0.80	5.33	1.73	0.34	1.70	0.89
SAMPLE3_64.1	0.12	0.79	2.94	2.28	0.11	1.08	5.26	2.52	0.34	2.28	0.90	2.94	2.37	0.11	1.00	5.39	2.36	0.34	2.37	0.91
SAMPLE3_21.1	0.11	0.81	3.02	1.72	0.11	0.97	5.14	1.97	0.33	1.72	0.87	3.01	1.82	0.11	1.07	5.15	1.87	0.33	1.82	0.83
SAMPLE2_33.1	0.12	1.39	2.94	2.09	0.11	2.25	5.26	3.07	0.34	2.09	0.68	2.95	2.62	0.11	10.28	5.11	11.48	0.34	2.62	0.55
SAMPLE1_52.1	0.12	0.88	2.94	1.75	0.11	1.14	5.27	2.08	0.34	1.75	0.84	2.94	1.90	0.11	1.29	5.27	1.96	0.34	1.90	0.78
SAMPLE3_68.1	0.11	0.70	2.90	1.42	0.11	0.76	5.34	1.61	0.34	1.42	0.88	2.91	1.49	0.11	1.40	5.27	2.03	0.34	1.49	0.72
SAMPLE3_33.1	0.11	0.47	5.97	1.56	0.11	0.56	2.59	1.65	0.17	1.56	0.94	5.91	1.63	0.12	0.73	2.82	1.60	0.17	1.63	0.90
SAMPLE2_61.1	0.11	0.76	3.01	1.78	0.11	0.89	5.14	1.99	0.33	1.78	0.89	3.00	1.83	0.12	0.86	5.32	1.90	0.33	1.83	0.90
SAMPLE3_94.1	0.11	0.72	2.91	1.44	0.11	0.77	5.32	1.63	0.34	1.44	0.88	2.89	1.57	0.12	1.26	5.54	1.82	0.35	1.57	0.73
SAMPLE3_76.1	0.11	0.59	3.82	1.36	0.11	0.60	4.05	1.49	0.26	1.36	0.91	3.79	1.53	0.12	1.02	4.27	1.59	0.26	1.53	0.79
SAMPLE2_90.1	0.11	0.68	3.39	1.41	0.11	0.79	4.56	1.61	0.29	1.41	0.87	3.35	1.45	0.12	0.88	5.03	1.54	0.30	1.45	0.83
SAMPLE2_39.1	0.11	0.73	2.99	1.69	0.11	0.88	5.17	1.91	0.33	1.69	0.89	2.97	1.83	0.12	1.06	5.40	1.80	0.34	1.83	0.83
SAMPLE3_37.1	0.11	1.31	3.27	1.94	0.11	1.45	4.73	2.42	0.31	1.94	0.80	3.27	2.15	0.11	1.89	4.74	2.52	0.31	2.15	0.68
SAMPLE3_13.1	0.11	0.81	3.05	1.69	0.11	1.02	5.07	1.98	0.33	1.69	0.86	3.05	1.77	0.11	1.01	5.07	1.85	0.33	1.77	0.84
SAMPLE3_2.1	0.11	1.21	3.11	1.61	0.11	1.27	4.97	2.05	0.32	1.61	0.78	3.09	1.66	0.12	1.22	5.21	1.97	0.32	1.66	0.79
SAMPLE1_95.1	0.11	0.52	3.96	2.30	0.11	0.52	3.90	2.36	0.25	2.30	0.98	3.95	2.38	0.11	0.65	4.00	2.30	0.25	2.38	0.96
SAMPLE2_32.1	0.12	0.80	2.97	1.71	0.11	1.07	5.19	2.01	0.34	1.71	0.85	2.97	1.80	0.11	1.03	5.31	1.85	0.34	1.80	0.84
SAMPLE3_26.1	0.11	0.96	3.86	1.59	0.11	1.00	4.00	1.88	0.26	1.59	0.85	3.85	1.67	0.11	1.04	4.12	1.82	0.26	1.67	0.83
SAMPLE2_38.1	0.11	0.75	3.05	1.77	0.11	0.90	5.05	1.99	0.33	1.77	0.89	3.05	1.91	0.11	1.08	5.07	1.89	0.33	1.91	0.84
SAMPLE1_76.1	0.11	1.95	2.92	1.57	0.11	2.02	5.28	2.56	0.34	1.57	0.61	2.91	1.66	0.12	1.97	5.46	2.45	0.34	1.66	0.60
SAMPLE1_43.1	0.11	1.18	3.08	1.71	0.11	1.24	4.99	2.11	0.32	1.71	0.81	3.09	1.84	0.11	1.39	4.95	2.07	0.32	1.84	0.75
SAMPLE1_23.1	0.11	1.69	3.78	3.18	0.11	1.81	4.07	3.66	0.26	3.18	0.87	3.75	3.33	0.12	1.89	4.34	3.58	0.27	3.33	0.85
SAMPLE2_97.1	0.11	0.52	4.63	1.61	0.11	0.56	3.32	1.71	0.22	1.61	0.95	4.52	1.63	0.13	0.73	4.04	1.76	0.22	1.63	0.91
SAMPLE3_63.1	0.12	1.13	2.82	1.91	0.11	2.00	5.46	2.76	0.36	1.91	0.69	2.80	2.12	0.12	1.77	5.79	2.40	0.36	2.12	0.70
SAMPLE1_35.1	0.11	1.41	4.23	1.63	0.11	1.50	3.64	2.22	0.24	1.63	0.74	4.19	1.69	0.12	1.42	3.91	2.11	0.24	1.69	0.74
SAMPLE3_87.1	0.11	1.23	4.45	1.33	0.11	1.25	3.45	1.83	0.22	1.33	0.73	4.35	1.38	0.13	1.16	4.14	1.74	0.23	1.38	0.75
SAMPLE3_93.1	0.11	0.60	4.18	3.02	0.11	0.65	3.68	3.09	0.24	3.02	0.98	4.10	3.13	0.13	1.23	4.32	3.09	0.24	3.13	0.92
SAMPLE1_93.1	0.11	0.51	4.43	1.61	0.11	0.51	3.47	1.69	0.23	1.61	0.95	4.41	1.86	0.11	1.62	3.58	2.31	0.23	1.86	0.72
SAMPLE2_36.1	0.11	0.78	2.91	1.69	0.11	0.93	5.29	1.92	0.34	1.69	0.88	2.90	1.84	0.11	1.16	5.37	1.84	0.34	1.84	0.80
SAMPLE1_41.1	0.12	0.99	2.99	1.80	0.11	1.37	5.14	2.27	0.33	1.80	0.80	2.97	1.93	0.12	1.31	5.42	2.03	0.34	1.93	0.78
SAMPLE3_55.1	0.12	0.83	2.99	1.71	0.11	1.23	5.13	2.10	0.33	1.71	0.81	2.98	1.81	0.12	1.08	5.36	1.86	0.34	1.81	0.83
SAMPLE3_8.1	0.12	1.04	2.93	1.83	0.11	1.48	5.22	2.36	0.34	1.83	0.78	2.93	1.94	0.11	1.35	5.25	2.10	0.34	1.94	0.78
SAMPLE2_30.1	0.11	0.92	2.97	1.76	0.11	1.19	5.15	2.13	0.34	1.76	0.83	2.97	1.87	0.11	1.19	5.24	1.96	0.34	1.87	0.81
SAMPLE2_37.1	0.11	0.78	4.24	1.62	0.11	1.00	3.60	1.91	0.24	1.62	0.85	4.16	1.65	0.13	0.77	4.21	1.74	0.24	1.65	0.90
SAMPLE1_98.1	0.11	0.89	3.26	1.50	0.11	0.94	4.67	1.77	0.31	1.50	0.85	3.28	1.65	0.11	1.47	4.50	1.98	0.31	1.65	0.69
SAMPLE2_89.1	0.11	1.16	3.74	1.64	0.11	1.36	4.07	2.13	0.27	1.64	0.77	3.76	2.04	0.11	4.76	3.88	5.56	0.27	2.04	0.55
SAMPLE1_33.1	0.11	0.79	4.91	3.29	0.11	0.80	3.10	3.39	0.20	3.29	0.97	4.97	3.42	0.10	1.21	2.76	3.42	0.20	3.42	0.94
SAMPLE2_59.1	0.12	1.02	3.00	1.83	0.11	1.54	5.07	2.40	0.33	1.83	0.76	2.99	1.95	0.11	1.33	5.24	2.07	0.33	1.95	0.78
SAMPLE1_14.1	0.11	1.54	2.87	3.82	0.11	1.78	5.29	4.22	0.35	3.82	0.91	2.86	4.04	0.11	1.99	5.48	3.98	0.35	4.04	0.88
SAMPLE2_46.1	0.11	1.38	2.98	2.52	0.11	1.53	5.10	2.95	0.34	2.52	0.86	2.98	2.72	0.11	2.11	5.17	2.87	0.34	2.72	0.72
SAMPLE1_83.1	0.11	0.61	4.43	2.30	0.11	0.93	3.42	2.48	0.23	2.30	0.93	4.40	2.43	0.12	1.16	3.65	2.38	0.23	2.43	0.88
SAMPLE3_24.1	0.11	0.60	5.33	1.58	0.11	0.69	2.84	1.72	0.19	1.58	0.92	5.33	1.68	0.11	1.10	2.83	1.75	0.19	1.68	0.79
SAMPLE3_59.1	0.12	1.37	2.98	2.09	0.11	2.82	5.08	3.51	0.34	2.09	0.59	2.96	2.26	0.11	1.93	5.35	2.58	0.34	2.26	0.69
SAMPLE3_4.1	0.12	1.04	2.98	1.84	0.11	1.65	5.07	2.48	0.34	1.84	0.74	2.97	1.98	0.11	1.41	5.28	2.11	0.34	1.98	0.76
SAMPLE2_54.1	0.11	0.98	4.55	1.75	0.11	1.38	3.32	2.22	0.22	1.75	0.79	4.52	1.88	0.12	1.29	3.53	2.00	0.22	1.88	0.78
SAMPLE1_78.1	0.11	0.98	3.18	1.50	0.11	1.05	4.75	1.83	0.31	1.50	0.82	3.17	1.59	0.11	1.17	4.90	1.76	0.32	1.59	0.76
SAMPLE3_35.1	0.12	1.27	2.87	2.00	0.11	2.18	5.25	2.96	0.35	2.00	0.67	2.85	2.46	0.11	4.94	5.55	5.94	0.35	2.46	0.58
SAMPLE2_45.1	0.12	1.74	2.99	2.33	0.11	2.88	5.03	3.70	0.33	2.33	0.63	2.99	2.53	0.11	2.38	5.06	3.05	0.33	2.53	0.65
SAMPLE1_64.1	0.11	2.70	5.33	3.48	0.11	3.18	2.82	4.72	0.19	3.48	0.74	5.08	3.61	0.15	2.57	4.04	4.24	0.20	3.61	0.80
SAMPLE3_39.1	0.12	1.49	3.04	2.13	0.11	2.72	4.94	3.45	0.33	2.13	0.62	3.04	2.31	0.11	2.06	5.03	2.71	0.33	2.31	0.67
SAMPLE1_84.1	0.11	1.15	5.00	1.84	0.11	1.20	2.99	2.20	0.20	1.84	0.84	4.98	1.90	0.11	1.23	3.11	2.13	0.20	1.90	0.82
SAMPLE1_73.1	0.11	1.10	3.02	2.85	0.11	1.58	4.95	3.26	0.33	2.85	0.87	3.01	2.99	0.11	1.74	5.13	2.98	0.33	2.99	0.83
SAMPLE3_36.1	0.11	0.69	4.92	1.60	0.11	0.82	3.03	1.80	0.20	1.60	0.89	4.90	1.64	0.11	0.76	3.19	1.72	0.20	1.64	0.90
SAMPLE1_3.1	0.11	1.68	2.86	2.15	0.11	2.44	5.22	3.25	0.35	2.15	0.66	2.86	2.32	0.11	2.42	5.22	2.89	0.35	2.32	0.59

Spot	204Pb /206Pb	±%	207Pb /206Pb	±%	208Pb /206Pb	±%	206Pb /238U	±%	% $206Pb_c$	ppm U	ppm Th	4-corr ppm $206Pb^*$	4-corr ppm $208Pb^*$
SAMPLE3_92.1	0.00	20.85	0.11	0.71	0.08	1.57	0.23	4.57	0.29	976.61	468.76	77.32	5.37
SAMPLE2_56.1	0.00	23.58	0.12	1.56	0.21	2.22	0.83	2.23	1.22	47.06	31.43	13.63	2.59
SAMPLE2_99.1	0.00	21.60	0.11	0.69	0.11	1.26	0.49	1.58	0.26	384.64	101.56	76.15	8.05
SAMPLE1_56.1	0.00	19.62	0.11	1.09	0.24	1.45	0.79	2.74	0.81	89.33	71.73	25.39	5.82
SAMPLE1_7.1	0.00	16.76	0.11	3.07	0.15	1.05	0.44	5.49	0.42	640.50	277.41	112.94	15.73
SAMPLE1_38.1	0.00	20.96	0.11	1.28	0.12	2.29	0.73	3.18	0.95	81.43	31.87	23.98	2.52
SAMPLE2_22.1	0.00	19.74	0.11	1.52	0.03	2.00	0.40	3.65	0.21	636.87	70.17	89.83	2.20
SAMPLE1_47.1	0.00	55.58	0.11	0.95	0.11	1.82	0.81	2.36	1.07	121.00	44.24	34.43	3.00
SAMPLE3_56.1	0.00	20.68	0.11	0.58	0.19	1.49	0.37	2.14	0.28	712.07	449.95	99.08	18.30
SAMPLE3_30.1	0.00	20.57	0.12	1.66	0.43	1.74	0.81	2.29	1.83	30.55	42.65	8.43	3.39
SAMPLE3_23.1	0.00	11.36	0.11	0.64	0.06	1.53	0.21	1.68	0.89	846.30	243.23	68.76	3.04
SAMPLE2_96.1	0.00	18.57	0.10	1.26	0.04	1.69	0.29	3.58	0.21	861.34	181.95	103.89	3.28
SAMPLE3_97.1	0.00	28.66	0.09	0.67	0.15	1.00	0.16	1.72	0.32	1031.80	497.07	68.12	10.03
SAMPLE2_87.1	0.00	27.16	0.08	0.89	0.34	0.89	0.45	3.43	0.23	288.63	308.20	48.71	16.57
SAMPLE2_48.1	0.00	33.34	0.08	1.08	0.20	1.34	0.36	5.39	0.21	403.11	247.76	73.12	14.39
SAMPLE2_84.1	0.00	29.14	0.08	1.33	0.32	1.33	0.35	2.50	0.45	195.83	176.07	39.55	12.64
SAMPLE3_10.1	0.00	23.58	0.06	1.70	0.23	1.95	0.19	1.73	0.94	251.51	165.87	16.99	3.59
SAMPLE3_14.1	0.00	19.62	0.07	5.51	0.22	2.08	0.20	2.66	1.82	165.13	110.77	11.05	2.10
SAMPLE2_29.1	0.00	23.36	0.06	1.94	0.31	1.77	0.20	1.82	0.24	1094.41	1051.47	73.55	22.54
SAMPLE1_99.1	0.00	67.08	0.06	1.05	0.32	0.91	0.19	0.50	-0.04	633.14	634.91	42.13	13.78
SAMPLE1_79.1	0.00	19.09	0.06	1.35	0.33	1.72	0.19	1.83	0.70	526.95	526.63	34.91	11.21
SAMPLE2_85.1	0.00	43.30	0.06	1.11	0.15	1.34	0.18	1.81	-0.11	579.06	267.52	38.31	6.01
SAMPLE2_80.1	0.00	83.21	0.06	1.73	0.13	2.22	0.18	1.54	-0.07	287.27	119.81	18.79	2.59
SAMPLE1_16.1	0.00	36.93	0.06	1.45	0.25	1.43	0.20	2.46	-0.26	397.13	300.87	26.00	6.59
SAMPLE1_17.1	0.00	41.61	0.07	2.12	0.22	2.33	0.19	3.15	0.50	170.20	97.47	11.17	2.38
SAMPLE3_66.1	0.00	9999.00	0.06	1.68	0.22	1.71	0.18	2.95	0.00	291.75	195.99	18.94	4.20
SAMPLE1_26.1	0.00	112.66	0.06	1.78	0.13	2.25	0.17	1.86	1.43	270.94	117.44	17.30	1.87
SAMPLE2_64.1	0.00	18.26	0.06	1.65	0.31	1.49	0.20	1.85	1.48	281.62	265.52	18.00	5.09
SAMPLE1_91.1	0.00	24.02	0.06	1.46	0.16	1.72	0.19	0.60	-0.62	320.53	160.23	20.77	3.67
SAMPLE1_12.1	0.00	28.87	0.06	1.93	0.17	2.31	0.19	0.82	0.82	208.06	103.61	13.07	2.06
SAMPLE1_34.1	0.00	100.00	0.06	2.92	0.17	2.01	0.19	0.81	0.05	265.46	137.66	16.60	2.78
SAMPLE1_37.1	0.00	37.21	0.06	1.71	0.27	1.64	0.18	3.59	0.36	322.32	249.75	20.03	5.29
SAMPLE2_71.1	0.00	113.40	0.06	4.17	0.22	4.38	0.17	2.64	-0.23	37.19	23.28	2.25	0.50
SAMPLE2_93.1	0.00	26.41	0.06	2.07	0.17	5.17	0.12	2.87	2.75	274.94	218.31	15.79	1.90
SAMPLE3_25.1	0.00	35.36	0.06	1.19	0.21	1.26	0.17	2.37	0.19	528.88	336.21	30.31	6.22

Spot	232Th /238U	±%	(1) 206Pb /238U Age		(2) 206Pb /238U Age		(3) 206Pb /238U Age		(1) 207Pb /206Pb Age		preferred age		(1) 208Pb /232Th Age		(2) 208Pb /232Th Age	
SAMPLE3_92.1	0.50	3.72	568.35	7.04	538.76	6.80	590.18	7.57	1769.36	16.06	1769.36	16.06	256.57	11.98	-202.88	13.77
SAMPLE2_56.1	0.69	0.86	1873.17	35.69	1887.25	40.73	1881.50	39.25	1765.74	53.31	1765.74	53.31	1777.08	80.83	1953.28	100.01
SAMPLE2_99.1	0.27	0.47	1337.03	20.50	1305.90	21.31	1323.85	21.34	1758.28	15.38	1758.28	15.38	1713.18	40.68	782.28	54.82
SAMPLE1_56.1	0.83	0.64	1842.75	29.82	1854.60	33.94	1852.52	33.43	1749.17	32.14	1749.17	32.14	1751.21	48.86	1874.04	66.13
SAMPLE1_7.1	0.45	1.81	1203.56	38.36	1169.12	39.46	1201.39	40.93	1748.37	59.29	1748.37	59.29	1240.27	50.97	616.09	91.69
SAMPLE1_38.1	0.40	0.85	1900.39	32.59	1921.50	37.64	1909.64	34.43	1743.19	39.40	1743.19	39.40	1711.13	88.07	2161.66	138.92
SAMPLE2_22.1	0.11	3.10	980.03	14.78	943.93	14.86	984.19	15.02	1738.24	29.03	1738.24	29.03	696.07	38.14	-1985.68	175.61
SAMPLE1_47.1	0.38	0.71	1844.32	30.29	1859.65	32.89	1861.15	30.37	1722.70	92.75	1722.70	92.75	1475.50	225.43	1827.12	113.72
SAMPLE3_56.1	0.65	1.05	967.80	13.97	932.91	13.96	974.21	15.38	1716.84	13.95	1716.84	13.95	897.46	21.92	475.40	16.26
SAMPLE3_30.1	1.44	0.76	1796.04	35.79	1815.21	40.36	1811.94	44.55	1633.26	71.34	1633.26	71.34	1718.25	60.16	1831.17	62.99
SAMPLE3_23.1	0.30	2.38	582.56	12.07	557.91	11.77	595.04	12.66	1598.76	21.17	1598.76	21.17	279.81	17.58	-355.49	21.26
SAMPLE2_96.1	0.22	2.65	847.01	45.25	820.83	45.20	859.96	46.60	1543.18	25.09	1543.18	25.09	402.54	28.29	-552.07	75.01
SAMPLE3_97.1	0.50	0.91	477.37	7.25	461.11	7.09	479.38	7.82	1369.93	21.70	1369.93	21.70	449.99	11.05	211.85	7.05
SAMPLE2_87.1	1.10	0.46	1156.27	14.73	1157.83	15.53	1152.65	17.55	1126.23	22.62	1126.23	22.62	1177.84	20.10	1188.66	21.49
SAMPLE2_48.1	0.63	0.64	1235.00	19.94	1241.86	21.25	1231.90	21.91	1114.54	26.89	1114.54	26.89	1269.72	30.05	1352.99	36.64
SAMPLE2_84.1	0.93	0.50	1361.32	18.57	1379.71	20.17	1334.68	21.62	1080.65	40.65	1080.65	40.65	1557.85	33.86	1711.00	38.55
SAMPLE3_10.1	0.68	0.51	487.90	7.75	491.11	7.87	488.48	8.73	265.72	91.54	491.11	7.87	482.68	16.30	515.06	14.60
SAMPLE3_14.1	0.69	0.54	483.55	18.31	486.15	18.74	490.31	20.77	302.81	207.13	486.15	18.74	422.92	25.19	448.85	29.90
SAMPLE2_29.1	0.99	0.42	485.56	8.87	486.14	9.04	486.83	10.58	447.02	48.20	486.14	9.04	477.96	12.55	481.98	13.37
SAMPLE1_99.1	1.04	0.40	481.01	6.31	481.50	6.42	480.49	7.55	448.24	24.82	481.50	6.42	483.95	8.12	487.19	8.46
SAMPLE1_79.1	1.03	0.42	478.91	6.17	480.04	6.26	479.67	7.51	401.55	55.76	480.04	6.26	474.58	11.19	482.12	11.04
SAMPLE2_85.1	0.48	0.64	478.35	6.09	477.99	6.18	476.71	6.57	502.56	28.32	477.99	6.18	500.61	10.34	495.38	11.12
SAMPLE2_80.1	0.43	0.47	473.14	6.36	472.68	6.46	472.60	6.82	504.51	42.04	472.68	6.46	481.27	13.39	473.86	15.44
SAMPLE1_16.1	0.78	0.49	473.55	7.38	471.28	7.46	471.70	8.39	623.66	40.87	471.28	7.46	488.13	11.34	467.95	11.12
SAMPLE1_17.1	0.59	0.60	474.79	8.44	470.75	8.48	468.53	9.35	734.54	74.39	470.75	8.48	542.50	19.71	494.83	17.75
SAMPLE3_66.1	0.69	0.43	469.63	6.30	470.04	6.42	468.74	7.10	440.74	37.40	470.04	6.42	477.59	10.59	481.72	11.95
SAMPLE1_26.1	0.45	0.95	462.08	15.06	469.25	13.59	469.60	14.35	-116.13	727.05	469.25	13.59	355.13	111.83	464.26	19.89
SAMPLE2_64.1	0.97	0.48	462.61	7.34	467.45	7.44	468.37	8.74	93.21	118.50	467.45	7.44	427.98	12.85	461.88	11.08
SAMPLE1_91.1	0.52	1.79	468.73	21.65	464.77	21.77	465.46	23.32	727.47	49.52	464.77	21.77	509.75	28.61	456.21	25.46
SAMPLE1_12.1	0.51	0.58	454.87	7.55	454.84	7.61	455.85	8.22	456.99	90.83	454.84	7.61	442.75	19.11	442.35	15.76
SAMPLE1_34.1	0.54	0.55	453.12	13.76	453.74	14.00	453.37	14.99	407.43	67.89	453.74	14.00	450.12	17.02	458.16	20.69
SAMPLE1_37.1	0.80	1.85	450.27	19.52	449.66	19.75	447.45	22.38	494.87	58.18	449.66	19.75	471.84	24.42	466.51	24.21
SAMPLE2_71.1	0.65	0.76	439.57	8.09	434.00	8.13	435.20	9.11	823.19	107.63	434.00	8.13	482.50	25.87	422.16	25.24
SAMPLE2_93.1	0.82	4.30	417.12	11.39	424.72	11.35	447.40	13.10	-310.84	383.49	424.72	11.35	195.87	28.86	259.15	19.66
SAMPLE3_25.1	0.66	0.47	416.30	6.36	416.27	6.44	416.66	7.09	418.96	34.92	416.27	6.44	412.91	8.92	412.57	9.21

Spot	(3) 207Pb /206Pb Age		% Dis- cor- dant	7corr $208Pb^*$ /232Th	±%	Total 238U /206Pb	±%	Total 207Pb /206Pb	±%	(1) 238U $/206Pb^*$	±%	(1) $207Pb^*$ $/206Pb^*$	±%
SAMPLE3_92.1	2249.76	29.16	70.82	-0.01	-6.75	10.82	1.29	0.11	0.71	10.85	1.29	0.11	0.88
SAMPLE2_56.1	1839.07	40.06	-7.01	0.10	5.37	2.93	2.18	0.12	1.56	2.97	2.20	0.11	2.92
SAMPLE2_99.1	1585.62	18.05	26.50	0.04	7.14	4.33	1.70	0.11	0.69	4.34	1.70	0.11	0.84
SAMPLE1_56.1	1836.72	31.44	-6.15	0.10	3.69	3.00	1.85	0.11	1.09	3.02	1.86	0.11	1.76
SAMPLE1_7.1	1718.58	63.10	34.12	0.03	15.11	4.85	3.49	0.11	3.07	4.87	3.49	0.11	3.24
SAMPLE1_38.1	1824.47	28.76	-10.42	0.11	6.78	2.89	1.97	0.11	1.28	2.92	1.98	0.11	2.15
SAMPLE2_22.1	1804.96	27.98	46.95	-0.09	-8.42	6.08	1.62	0.11	1.52	6.09	1.63	0.11	1.58
SAMPLE1_47.1	1872.25	21.01	-8.12	0.09	6.51	2.99	1.79	0.11	0.95	3.02	1.89	0.11	5.05
SAMPLE3_56.1	1820.61	23.13	46.92	0.02	3.46	6.16	1.55	0.11	0.58	6.17	1.55	0.11	0.76
SAMPLE3_30.1	1785.97	127.32	-11.43	0.09	3.60	3.06	2.25	0.12	1.66	3.11	2.28	0.10	3.84
SAMPLE3_23.1	1920.04	17.55	66.40	-0.02	-5.93	10.48	2.16	0.11	0.64	10.57	2.17	0.10	1.13
SAMPLE2_96.1	1792.81	24.26	48.08	-0.03	-13.40	7.11	5.70	0.10	1.26	7.12	5.70	0.10	1.33
SAMPLE3_97.1	1449.75	20.76	67.54	0.01	3.35	12.97	1.57	0.09	0.67	13.01	1.57	0.09	1.13
SAMPLE2_87.1	1049.78	52.16	-2.91	0.06	1.86	5.08	1.39	0.08	0.89	5.09	1.39	0.08	1.14
SAMPLE2_48.1	1052.68	35.39	-11.88	0.07	2.80	4.73	1.77	0.08	1.08	4.74	1.77	0.08	1.35
SAMPLE2_84.1	485.96	83.23	-28.83	0.09	2.35	4.23	1.51	0.08	1.33	4.25	1.51	0.08	2.03
SAMPLE3_10.1	308.67	65.53	-86.84	0.03	2.87	12.60	1.63	0.06	1.70	12.72	1.65	0.05	3.99
SAMPLE3_14.1	738.52	130.55	-61.96	0.02	6.74	12.60	3.91	0.07	5.51	12.84	3.93	0.05	9.09
SAMPLE2_29.1	531.96	102.26	-8.95	0.02	2.81	12.75	1.90	0.06	1.94	12.78	1.90	0.06	2.17
SAMPLE1_99.1	411.85	66.92	-7.59	0.02	1.76	12.91	1.36	0.06	1.05	12.91	1.36	0.06	1.12
SAMPLE1_79.1	454.98	115.32	-19.99	0.02	2.32	12.88	1.33	0.06	1.35	12.97	1.34	0.05	2.49
SAMPLE2_85.1	386.70	36.46	5.00	0.02	2.27	13.00	1.32	0.06	1.11	12.98	1.32	0.06	1.29
SAMPLE2_80.1	467.22	45.04	6.45	0.02	3.30	13.14	1.39	0.06	1.73	13.13	1.39	0.06	1.91
SAMPLE1_16.1	500.83	56.23	24.96	0.02	2.40	13.15	1.61	0.06	1.45	13.12	1.62	0.06	1.89
SAMPLE1_17.1	302.02	78.98	36.67	0.02	3.63	13.02	1.83	0.07	2.12	13.08	1.84	0.06	3.51
SAMPLE3_66.1	375.92	56.16	-6.80	0.02	2.51	13.23	1.39	0.06	1.68	13.23	1.39	0.06	1.68
SAMPLE1_26.1	492.78	55.67	516.25	0.02	4.33	13.26	2.96	0.06	1.78	13.46	3.38	0.04	29.50
SAMPLE2_64.1	529.51	84.80	-410.83	0.02	2.43	13.24	1.62	0.06	1.65	13.44	1.64	0.05	5.00
SAMPLE1_91.1	513.67	69.70	36.87	0.02	5.64	13.34	4.79	0.06	1.46	13.26	4.79	0.06	2.34
SAMPLE1_12.1	526.41	56.07	0.48	0.02	3.60	13.56	1.70	0.06	1.93	13.68	1.72	0.06	4.09
SAMPLE1_34.1	426.32	76.77	-11.61	0.02	4.57	13.73	3.14	0.06	2.92	13.73	3.14	0.05	3.03
SAMPLE1_37.1	275.69	122.96	9.33	0.02	5.25	13.77	4.49	0.06	1.71	13.82	4.49	0.06	2.64
SAMPLE2_71.1	527.42	122.15	48.19	0.02	6.04	14.20	1.89	0.06	4.17	14.17	1.90	0.07	5.16
SAMPLE2_93.1	1551.27	101.30	242.00	0.01	7.64	14.55	2.72	0.06	2.07	14.96	2.82	0.04	14.97
SAMPLE3_25.1	447.72	42.59	0.66	0.02	2.26	14.96	1.58	0.06	1.19	14.99	1.58	0.06	1.56

Spot	(1) $207Pb^*$ /235U	±%	(1) $206Pb^*$ /238U	±%	err corr	(3) 238U $/206Pb^*$	±%	(3) $207Pb^*$ $/206Pb^*$	±%	(3) $207Pb^*$ /235U	±%	(3) $206Pb^*$ /238U	±%	err. corr.
SAMPLE3_92.1	1.38	1.56	0.09	1.29	0.83	10.43	1.34	0.14	1.69	1.87	2.32	0.10	1.34	0.70
SAMPLE2_56.1	5.02	3.65	0.34	2.20	0.60	2.95	2.41	0.11	2.21	5.25	2.86	0.34	2.41	0.66
SAMPLE2_99.1	3.42	1.89	0.23	1.70	0.90	4.39	1.78	0.10	0.97	3.08	1.84	0.23	1.78	0.86
SAMPLE1_56.1	4.88	2.56	0.33	1.86	0.73	3.00	2.08	0.11	1.74	5.15	2.36	0.33	2.08	0.70
SAMPLE1_7.1	3.03	4.76	0.21	3.49	0.73	4.88	3.73	0.11	3.43	2.97	4.80	0.20	3.73	0.70
SAMPLE1_38.1	5.04	2.92	0.34	1.98	0.68	2.90	2.08	0.11	1.59	5.30	2.34	0.34	2.08	0.75
SAMPLE2_22.1	2.41	2.27	0.16	1.63	0.72	6.06	1.65	0.11	1.54	2.51	2.23	0.16	1.65	0.72
SAMPLE1_47.1	4.82	5.39	0.33	1.89	0.35	2.99	1.88	0.11	1.16	5.28	1.99	0.33	1.88	0.82
SAMPLE3_56.1	2.35	1.73	0.16	1.55	0.90	6.13	1.70	0.11	1.27	2.50	1.89	0.16	1.70	0.75
SAMPLE3_30.1	4.45	4.47	0.32	2.28	0.51	3.08	2.82	0.11	6.99	4.89	8.10	0.32	2.82	0.54
SAMPLE3_23.1	1.29	2.45	0.09	2.17	0.89	10.34	2.23	0.12	0.98	1.57	2.37	0.10	2.23	0.91
SAMPLE2_96.1	1.85	5.86	0.14	5.70	0.97	7.01	5.79	0.11	1.33	2.16	5.79	0.14	5.79	0.97
SAMPLE3_97.1	0.93	1.94	0.08	1.57	0.81	12.95	1.69	0.09	1.09	0.97	1.83	0.08	1.69	0.81
SAMPLE2_87.1	2.09	1.80	0.20	1.39	0.77	5.11	1.66	0.07	2.59	2.01	3.08	0.20	1.66	0.54
SAMPLE2_48.1	2.23	2.23	0.21	1.77	0.80	4.75	1.95	0.07	1.76	2.16	2.24	0.21	1.95	0.66
SAMPLE2_84.1	2.45	2.53	0.24	1.51	0.60	4.35	1.79	0.06	3.77	1.80	4.14	0.23	1.79	0.41
SAMPLE3_10.1	0.56	4.32	0.08	1.65	0.38	12.70	1.86	0.05	2.88	0.57	3.20	0.08	1.86	0.45
SAMPLE3_14.1	0.56	9.90	0.08	3.93	0.40	12.65	4.40	0.06	6.17	0.70	7.14	0.08	4.40	0.51
SAMPLE2_29.1	0.60	2.88	0.08	1.90	0.66	12.75	2.26	0.06	4.67	0.63	5.28	0.08	2.26	0.47
SAMPLE1_99.1	0.60	1.76	0.08	1.36	0.77	12.92	1.63	0.05	2.99	0.59	3.44	0.08	1.63	0.49
SAMPLE1_79.1	0.58	2.83	0.08	1.34	0.47	12.95	1.63	0.06	5.20	0.60	5.71	0.08	1.63	0.45
SAMPLE2_85.1	0.61	1.84	0.08	1.32	0.72	13.03	1.43	0.05	1.62	0.58	1.93	0.08	1.43	0.57
SAMPLE2_80.1	0.60	2.36	0.08	1.39	0.59	13.15	1.50	0.06	2.03	0.59	2.30	0.08	1.50	0.49
SAMPLE1_16.1	0.64	2.49	0.08	1.62	0.65	13.17	1.84	0.06	2.55	0.60	2.94	0.08	1.84	0.51
SAMPLE1_17.1	0.67	3.97	0.08	1.84	0.46	13.26	2.07	0.05	3.46	0.54	3.79	0.08	2.07	0.43
SAMPLE3_66.1	0.58	2.18	0.08	1.39	0.64	13.26	1.57	0.05	2.50	0.56	2.78	0.08	1.57	0.45
SAMPLE1_26.1	0.45	29.69	0.07	3.38	0.11	13.23	3.17	0.06	2.53	0.59	3.53	0.08	3.17	0.72
SAMPLE2_64.1	0.49	5.27	0.07	1.64	0.31	13.27	1.93	0.06	3.87	0.60	4.39	0.08	1.93	0.47
SAMPLE1_91.1	0.66	5.33	0.08	4.79	0.90	13.36	5.19	0.06	3.17	0.59	5.40	0.07	5.19	0.82
SAMPLE1_12.1	0.57	4.44	0.07	1.72	0.39	13.65	1.87	0.06	2.56	0.59	2.89	0.07	1.87	0.49
SAMPLE1_34.1	0.55	4.37	0.07	3.14	0.72	13.72	3.42	0.06	3.44	0.56	4.35	0.07	3.42	0.63
SAMPLE1_37.1	0.57	5.21	0.07	4.49	0.86	13.91	5.18	0.05	5.37	0.51	6.79	0.07	5.18	0.63
SAMPLE2_71.1	0.65	5.50	0.07	1.90	0.35	14.32	2.16	0.06	5.57	0.56	6.09	0.07	2.16	0.41
SAMPLE2_93.1	0.37	15.23	0.07	2.82	0.19	13.91	3.03	0.10	5.39	0.95	6.62	0.07	3.03	0.60
SAMPLE3_25.1	0.51	2.22	0.07	1.58	0.71	14.98	1.76	0.06	1.92	0.51	2.28	0.07	1.76	0.58

Table S5-4. LA-MC-ICP-MS zircon U-Pb isotope age data of PBSC unit from Biryong-dong (Jang, 2017).

	207_235	2SE	206_238	2SE	Error Correlation	238_206	2SE	207_206	2SE	207_235	2SE	206_238	2SE	Error Correlation	238_206	2SE	207_206	2SE	207_235	2SE	206_238	2SE	207_206	2SE	207_235	2SE	206_238	2SE	207_206	2SE	Approx_U_PPM	Approx_Th_PPM	Approx_Pb_PPM	Th/U	Final Disc Percent
HM-1084-55	15.99	0.18	0.55	0.01	0.97	1.81	0.02	0.20	0.00	15.98	0.18	0.55	0.01	0.95	1.81	0.02	0.20	0.00	2875.00	11.00	2831.00	24.00	2857.00	2.70	2874.00	11.00	2833.00	24.00	2861.70	4.70	79.70	80.90	118.00	1.06	0.85
HM-1084-1	12.56	0.09	0.54	0.00	0.92	1.84	0.01	0.17	0.00	12.56	0.09	0.54	0.00	0.92	1.84	0.01	0.17	0.00	2647.00	6.60	2803.00	16.00	2524.20	2.80	2647.00	6.60	2803.00	16.00	2524.20	2.80	109.30	109.30	181.20	1.07	-11.03
HM-1084-25	11.82	0.12	0.51	0.00	0.93	1.95	0.02	0.17	0.00	11.82	0.12	0.51	0.00	0.93	1.95	0.02	0.17	0.00	2589.30	9.70	2668.00	20.00	2526.20	3.50	2589.30	9.70	2668.00	20.00	2526.20	3.50	55.90	47.60	61.40	0.85	-5.54
HM-1084-6	12.06	0.22	0.50	0.01	0.97	2.01	0.04	0.17	0.00	12.08	0.27	0.50	0.01	0.91	2.00	0.04	0.17	0.00	2608.00	17.00	2606.00	38.00	2608.20	4.80	2609.00	22.00	2609.00	39.00	2600.00	12.00	491.00	142.70	173.40	0.31	0.00
HM-1084-10	10.95	0.13	0.49	0.01	0.97	2.05	0.02	0.16	0.00	10.96	0.13	0.49	0.01	0.94	2.05	0.02	0.16	0.00	2520.00	11.00	2565.00	22.00	2484.40	3.00	2519.00	11.00	2561.00	23.00	2485.20	3.20	255.00	55.90	65.00	0.23	-3.20
HM-1084-33	11.36	0.17	0.49	0.01	0.99	2.05	0.03	0.17	0.00	11.44	0.14	0.49	0.01	0.92	2.07	0.03	0.17	0.00	2551.00	14.00	2556.00	31.00	2552.70	2.50	2558.00	12.00	2553.00	31.00	2568.00	16.00	197.50	162.30	188.00	0.82	0.10
HM-1084-51	12.55	0.14	0.49	0.01	0.95	2.05	0.02	0.18	0.00	12.66	0.13	0.49	0.01	0.90	2.06	0.02	0.18	0.00	2646.00	11.00	2556.00	25.00	2666.60	3.50	2657.30	9.80	2558.00	25.00	2691.00	11.00	147.00	137.00	179.60	0.99	4.25
HM-1084-98	11.40	0.16	0.49	0.01	0.96	2.06	0.02	0.17	0.00	11.40	0.16	0.49	0.01	0.64	2.06	0.03	0.17	0.00	2556.00	13.00	2555.00	26.00	2557.20	3.60	2556.00	13.00	2555.00	26.00	2557.20	3.60	340.00	171.80	202.50	0.52	0.10
HM-1084-5	10.93	0.18	0.48	0.01	0.98	2.08	0.03	0.16	0.00	10.87	0.23	0.48	0.01	0.94	2.09	0.04	0.16	0.00	2515.00	16.00	2525.00	33.00	2498.80	4.70	2503.00	23.00	2526.00	34.00	2480.00	16.00	111.20	83.70	100.50	0.84	-0.80
HM-1084-16	11.03	0.11	0.48	0.00	0.94	2.09	0.02	0.17	0.00	11.18	0.11	0.48	0.00	0.83	2.09	0.02	0.17	0.00	2525.00	9.10	2523.00	18.00	2526.10	3.10	2535.90	9.30	2527.00	18.00	2525.20	7.40	63.20	38.70	46.40	0.62	0.19
HM-1084-50	10.80	0.28	0.47	0.01	0.99	2.12	0.05	0.16	0.00	11.19	0.18	0.48	0.01	0.86	2.12	0.06	0.17	0.00	2501.00	25.00	2500.00	52.00	2505.30	3.30	2537.00	15.00	2512.00	49.00	2533.00	17.00	114.90	54.40	64.70	0.47	0.50
HM-1084-84	10.12	0.21	0.46	0.01	0.97	2.17	0.04	0.16	0.00	10.22	0.23	0.46	0.01	0.88	2.18	0.04	0.16	0.00	2442.00	19.00	2440.00	38.00	2431.10	5.30	2450.00	21.00	2439.00	39.00	2423.00	19.00	51.80	43.60	53.80	0.81	-0.30
HM-1084-32	8.93	0.26	0.44	0.01	0.87	2.30	0.05	0.15	0.00	8.93	0.26	0.44	0.01	0.87	2.28	0.06	0.15	0.00	2330.00	27.00	2327.00	47.00	2321.00	15.00	2330.00	27.00	2327.00	47.00	2321.00	15.00	45.90	32.10	33.60	0.72	-0.20
HM-1084-2	7.32	0.11	0.43	0.01	0.91	2.33	0.03	0.12	0.00	7.32	0.11	0.43	0.01	0.91	2.34	0.03	0.12	0.00	2150.00	14.00	2297.00	28.00	2013.30	7.40	2150.00	14.00	2297.00	28.00	2013.30	7.40	58.40	49.60	63.80	0.92	-14.60
HM-1084-15	8.69	0.72	0.43	0.03	1.00	2.34	0.19	0.15	0.00	8.68	0.80	0.43	0.03	0.98	2.50	0.17	0.15	0.00	2275.00	70.00	2280.00	150.00	2324.40	9.90	2287.00	87.00	2270.00	150.00	2384.00	27.00	292.00	135.50	131.90	0.48	2.50
HM-1084-19	7.87	0.15	0.41	0.01	0.95	2.43	0.05	0.14	0.00	7.86	0.17	0.41	0.01	0.84	2.42	0.05	0.14	0.00	2217.00	17.00	2220.00	36.00	2199.00	6.60	2214.00	20.00	2239.00	40.00	2194.00	14.00	68.10	44.30	54.10	0.68	-0.90
HM-1084-4	7.27	0.09	0.40	0.00	0.97	2.53	0.03	0.13	0.00	7.34	0.09	0.40	0.00	0.96	2.52	0.03	0.13	0.00	2145.00	10.00	2149.00	21.00	2139.00	3.40	2153.00	10.00	2151.00	22.00	2140.20	3.90	170.10	112.70	114.40	0.68	-0.44
HM-1084-39	6.45	0.06	0.39	0.00	0.89	2.58	0.02	0.12	0.00	6.45	0.06	0.39	0.00	0.89	2.58	0.02	0.12	0.00	2038.30	7.90	2114.00	15.00	1958.00	4.70	2038.30	7.90	2114.00	15.00	1958.00	4.70	96.00	17.40	18.92	0.17	-7.93
HM-1084-40	8.39	0.12	0.39	0.00	0.99	2.59	0.03	0.16	0.00	6.74	0.19	0.37	0.01	0.99	2.68	0.04	0.13	0.00	2275.00	13.00	2107.00	23.00	2426.60	3.30	2076.00	25.00	2052.00	25.00	2104.00	23.00	339.00	66.30	28.55	0.19	13.17
HM-1084-42	6.43	0.07	0.38	0.00	0.95	2.61	0.03	0.12	0.00	6.43	0.07	0.38	0.00	0.95	2.61	0.03	0.12	0.00	2036.10	9.10	2092.00	18.00	1973.10	3.50	2036.10	9.10	2092.00	18.00	1973.10	3.50	173.00	56.20	58.80	0.33	-5.95
HM-1084-26	6.13	0.08	0.38	0.00	0.90	2.63	0.03	0.12	0.00	6.13	0.08	0.38	0.00	0.91	2.63	0.03	0.12	0.00	1994.00	11.00	2078.00	20.00	1915.60	5.00	1995.00	11.00	2078.00	20.00	1914.40	5.10	64.40	108.20	110.90	1.68	-8.80
HM-1084-18	6.67	0.09	0.38	0.00	0.95	2.64	0.03	0.13	0.00	6.72	0.10	0.38	0.00	0.80	2.63	0.03	0.13	0.00	2068.00	12.00	2072.00	21.00	2058.60	5.10	2074.00	13.00	2077.00	21.00	2057.30	5.70	177.00	68.60	64.60	0.39	-0.90
HM-1084-38	6.69	0.08	0.38	0.00	0.95	2.64	0.03	0.13	0.00	6.71	0.08	0.38	0.00	0.92	2.64	0.03	0.13	0.00	2070.00	10.00	2072.00	19.00	2063.60	3.70	2073.00	10.00	2071.00	19.00	2063.10	4.60	104.60	122.00	117.20	1.21	-0.38
HM-1084-85	6.64	0.06	0.38	0.00	0.91	2.65	0.02	0.13	0.00	6.66	0.08	0.38	0.00	0.72	2.65	0.02	0.13	0.00	2065.00	8.40	2062.00	16.00	2068.50	3.60	2066.00	11.00	2065.00	16.00	2057.00	18.00	173.10	46.10	42.57	0.27	0.28
HM-1084-81	6.43	0.06	0.37	0.00	0.92	2.73	0.02	0.13	0.00	6.48	0.13	0.37	0.00	0.72	2.72	0.02	0.12	0.00	2036.20	7.90	2010.00	14.00	2043.90	4.10	2043.00	19.00	2021.00	15.00	2052.00	32.00	152.40	48.40	46.80	0.32	1.62
HM-1084-64	5.97	0.07	0.36	0.00	0.95	2.79	0.03	0.12	0.00	6.02	0.08	0.36	0.00	0.73	2.80	0.03	0.12	0.00	1971.00	10.00	1973.00	20.00	1953.20	3.90	1980.00	13.00	1975.00	20.00	1945.00	18.00	117.60	26.70	28.10	0.24	-1.20
HM-1084-12	5.66	0.06	0.36	0.00	0.90	2.80	0.02	0.12	0.00	5.66	0.06	0.36	0.00	0.90	2.80	0.02	0.11	0.00	1925.30	8.60	1968.00	14.00	1879.10	4.00	1925.30	8.60	1968.00	14.00	1879.10	4.00	203.00	77.90	70.40	0.40	-4.70
HM-1084-41	5.65	0.04	0.36	0.00	0.91	2.80	0.02	0.11	0.00	5.65	0.04	0.36	0.00	0.85	2.80	0.02	0.11	0.00	1923.00	6.70	1968.00	13.00	1874.40	2.90	1923.60	6.70	1967.00	13.00	1873.40	3.10	106.30	39.20	38.00	0.36	-5.16
HM-1084-9	5.43	0.05	0.35	0.00	0.92	2.88	0.02	0.11	0.00	5.43	0.04	0.35	0.00	0.90	2.88	0.02	0.11	0.00	1888.60	7.10	1925.00	13.00	1860.00	3.30	1889.30	6.80	1922.00	13.00	1858.70	3.50	138.70	90.60	86.07	0.69	-3.51
HM-1084-30	5.42	0.05	0.35	0.00	0.93	2.89	0.02	0.11	0.00	5.45	0.04	0.35	0.00	0.78	2.90	0.02	0.11	0.00	1886.90	7.40	1916.00	13.00	1866.60	2.90	1891.30	6.60	1915.00	13.00	1865.80	3.90	120.10	78.80	68.70	0.65	-2.65
HM-1084-49	5.47	0.05	0.35	0.00	0.96	2.90	0.03	0.11	0.00	5.46	0.06	0.35	0.00	0.93	2.90	0.03	0.11	0.00	1894.90	8.50	1911.00	16.00	1862.90	2.90	1893.60	8.80	1911.00	16.00	1864.70	3.40	308.00	119.40	106.10	0.41	-2.47
HM-1084-29	5.24	0.07	0.34	0.00	0.89	2.90	0.04	0.11	0.00	5.26	0.07	0.34	0.00	0.87	2.89	0.04	0.11	0.00	1859.00	11.00	1909.00	20.00	1813.90	6.50	1862.00	12.00	1908.00	21.00	1812.40	7.10	78.70	73.80	72.40	0.94	-5.30
HM-1084-76	5.40	0.04	0.34	0.00	0.93	2.90	0.02	0.11	0.00	5.40	0.04	0.34	0.00	0.93	2.90	0.02	0.11	0.00	1884.70	6.00	1909.00	11.00	1860.70	3.10	1884.00	6.10	1909.00	11.00	1860.60	3.20	232.00	69.20	65.50	0.30	-2.52
HM-1084-28	5.36	0.10	0.34	0.01	0.98	2.92	0.05	0.11	0.00	5.39	0.09	0.34	0.01	0.62	2.92	0.05	0.11	0.00	1878.00	16.00	1897.00	30.00	1861.10	4.60	1882.00	14.00	1900.00	27.00	1848.00	21.00	457.00	188.00	133.10	0.41	-1.00
HM-1084-34	5.41	0.11	0.34	0.01	0.98	2.93	0.06	0.12	0.00	5.32	0.15	0.34	0.01	0.97	2.96	0.06	0.11	0.00	1889.00	17.00	1889.00	32.00	1893.50	4.60	1864.00	25.00	1887.00	33.00	1840.00	19.00	274.00	62.90	48.50	0.22	0.20
HM-1084-94	5.45	0.04	0.34	0.00	0.90	2.94	0.02	0.12	0.00	5.49	0.05	0.34	0.00	0.83	2.93	0.02	0.12	0.00	1893.50	6.80	1890.00	11.00	1899.80	3.50	1897.90	7.40	1891.00	11.00	1899.30	5.20	153.80	111.70	105.30	0.74	0.23
HM-1084-65	5.41	0.08	0.34	0.00	0.96	2.94	0.04	0.11	0.00	5.47	0.08	0.34	0.00	0.77	2.95	0.04	0.11	0.00	1885.00	13.00	1887.00	23.00	1870.10	4.00	1895.00	12.00	1889.00	23.00	1878.00	10.00	237.00	104.00	89.20	0.44	-0.60
HM-1084-37	5.37	0.08	0.34	0.00	0.99	2.95	0.04	0.11	0.00	5.51	0.07	0.34	0.00	0.73	2.94	0.04	0.12	0.00	1879.00	13.00	1883.00	23.00	1873.40	3.70	1901.00	10.00	1884.00	22.00	1917.00	18.00	143.20	71.20	62.40	0.48	-0.40
HM-1084-74	5.41	0.05	0.34	0.00	0.92	2.95	0.03	0.12	0.00	5.39	0.08	0.34	0.00	0.70	2.95	0.03	0.12	0.00	1885.60	7.60	1883.00	15.00	1912.80	3.90	1887.00	15.00	1883.00	16.00	1900.00	24.00	194.00	48.50	41.60	0.26	1.80
HM-1084-77	5.38	0.14	0.34	0.01	0.99	2.96	0.08	0.12	0.00	5.16	0.22	0.34	0.01	0.95	3.04	0.10	0.11	0.00	1875.00	23.00	1873.00	47.00	1878.40	6.40	1826.00	39.00	1860.00	50.00	1783.00	31.00	437.00	112.00	95.90	0.31	0.50
HM-1084-59	5.30	0.06	0.34	0.00	0.97	2.96	0.04	0.11	0.00	5.32	0.07	0.34	0.00	0.30	2.96	0.04	0.11	0.00	1872.00	11.00	1876.00	19.00	1865.00	4.40	1872.00	11.00	1876.00	19.00	1863.70	5.10	457.00	234.00	194.00	0.52	-1.00
HM-1084-99	5.34	0.05	0.34	0.00	0.94	2.96	0.03	0.11	0.00	5.38	0.06	0.34	0.00	0.79	2.95	0.03	0.11	0.00	1874.00	8.00	1873.00	14.00	1864.50	3.00	1880.00	10.00	1878.00	14.00	1858.00	14.00	211.00	110.00	109.60	0.54	-0.43
HM-1084-20	5.35	0.10	0.34	0.01	0.99	2.97	0.05	0.12	0.00	5.34	0.14	0.34	0.01	0.94	3.01	0.07	0.11	0.00	1873.00	17.00	1871.00	30.00	1878.50	2.80	1863.00	27.00	1867.00	32.00	1859.00	29.00	283.00	118.60	96.80	0.47	0.50
HM-1084-62	5.37	0.04	0.34	0.00	0.88	2.97	0.03	0.11	0.00	5.42	0.05	0.34	0.00	0.64	2.97	0.03	0.12	0.00	1880.80	7.10	1872.00	14.00	1878.40	4.40	1889.30	9.50	1874.00	14.00	1879.00	10.00	120.70	40.20	36.20	0.36	0.24
HM-1084-97	5.31	0.07	0.34	0.00	0.97	2.97	0.04	0.11	0.00	5.43	0.07	0.34	0.00	0.70	2.97	0.04	0.11	0.00	1869.00	12.00	1870.00	20.00	1868.40	3.70	1893.00	12.00	1874.00	19.00	1879.00	13.00	167.00	87.10	85.30	0.52	-0.30
HM-1084-8	5.29	0.08	0.34	0.00	0.92	2.98	0.04	0.11	0.00	5.31	0.08	0.34	0.00	0.77	2.98	0.04	0.11	0.00	1867.00	13.00	1866.00	21.00	1868.50	4.40	1870.00	13.00	1866.00	21.00	1868.50	4.40	162.20	95.90	95.10	0.62	-0.10
HM-1084-86	5.28	0.10	0.34	0.00	0.91	2.98	0.04	0.11	0.00	5.29	0.10	0.34	0.00	0.86	2.97	0.04	0.11	0.00	1864.00	15.00	1865.00	23.00	1867.80	7.30	1866.00	15.00	1864.00	24.00	1861.80	9.60	150.40	65.40	64.80	0.45	-0.10
HM-1084-14	5.26	0.07	0.33	0.00	0.94	2.99	0.04	0.11	0.00	5.34	0.07	0.33	0.00	0.71	3.00	0.04	0.11	0.00	1862.00	11.00	1858.00	21.00	1860.30	5.20	1874.00	11.00	1860.00	21.00	1870.00	13.00	95.40	68.80	63.39	0.74	0.40
HM-1084-48	5.25	0.08	0.33	0.01	0.97	3.00	0.04	0.11	0.00	5.49	0.07	0.34	0.00	0.69	2.99	0.04	0.11	0.00	1860.00	14.00	1858.00	25.00	1857.10	3.60	1897.00	12.00	1870.00	23.00	1878.00	18.00	227.00	115.00	98.60	0.52	0.40
HM-1084-27	4.94	0.08	0.33	0.00	0.94	3.01	0.04	0.11	0.00	4.96	0.07	0.33	0.00	0.95	3.00	0.04	0.11	0.00	1809.00	13.00	1855.00	20.00	1769.80	4.80	1811.00	12.00	1855.00	20.00	1769.60	5.30	242.00	47.20	39.10	0.20	-4.30
HM-1084-3	5.19	0.11	0.33	0.01	0.99	3.01	0.06	0.11	0.00	5.10	0.16	0.33	0.01	0.99	3.06	0.08	0.11	0.00	1848.00	20.00	1845.00	33.00	1852.30	3.40	1829.00	30.00	1847.00	33.00	1810.00	28.00	320.70	54.10	42.40	0.16	0.20
HM-1084-31	5.18	0.08	0.33	0.00	0.97	3.02	0.04	0.11	0.00	5.25	0.07	0.33	0.00	0.72	3.01	0.04	0.11	0.00	1848.00	12.00	1848.00	22.00	1855.40	3.50	1860.00	11.00	1851.00	21.00	1862.00	11.00	182.00	109.70	99.80	0.62	0.40
HM-1084-7	5.20	0.22	0.33	0.01	0.99	3.02	0.12	0.11	0.00	5.04	0.34	0.33	0.01	0.99	3.09	0.18	0.11	0.00	1846.00	41.00	1842.00	66.00	1864.90	8.50	1808.00	70.00	1836.00	71.00	1808.00	59.00	166.00	60.20	61.10	0.37	1.30
HM-1084-23	5.20	0.16	0.33	0.01	1.00	3.02	0.09	0.11	0.00	4.89	0.27	0.33	0.01	0.98	3.13	0.14	0.11	0.00	1846.00	29.00	1840.00	51.00	1845.10	7.00	1788.00	54.00	1832.00	54.00	1713.00	61.00	400.00	233.00	206.30	0.61	0.40
HM-1084-71	5.10	0.09	0.33	0.01	0.99	3.03	0.05	0.11	0.00	5.15	0.09	0.33	0.01	0.94	3.05	0.05	0.11	0.00	1834.00	15.00	1835.00	26.00	1855.40	2.90	1843.00	15.00	1835.00	26.00	1853.80	8.70	225.00	154.10	131.90	0.68	0.90
HM-1084-69	5.05	0.05	0.33	0.00	0.93	3.05	0.03	0.11	0.00	5.08	0.04	0.33	0.00	0.84	3.05	0.03	0.11	0.00	1827.00	9.00	1829.00	18.00	1848.50	5.00	1832.00	7.30	1830.00	17.00	1848.50	6.10	250.00	218.00	174.00	0.89	1.09
HM-1084-82	7.56	0.22	0.32	0.01	1.00	3.08	0.09	0.17	0.00	5.99	0.47	0.31	0.01	0.96	3.24	0.12	0.13	0.01	2178.00	27.00	1808.00	44.00	2525.70	3.70	1903.00	73.00	1743.00	53.00	2063.00	90.00	298.00	231.80	193.10	0.78	28.50
HM-1084-92	5.03	0.03	0.32	0.00	0.93	3.11	0.02	0.11	0.00	5.08	0.09	0.32	0.00	0.71	3.10	0.02	0.11	0.00	1824.50	5.30	1797.80	9.60	1858.20	2.10	1830.00	16.00	1801.00	10.00	1853.00	30.00	326.00	93.10	80.40	0.30	3.20
HM-1084-60	5.04	0.06	0.32	0.00	0.95	3.12	0.04	0.11	0.00	5.13	0.14	0.32	0.00	0.38	3.09	0.03	0.11	0.00	1826.00	11.00	1791.00	18.00	1862.30	4.30	1850.00	27.00	1801.00	17.00	1873.00	41.00	324.00	86.00	75.40	0.28	3.90
HM-1084-88	4.97	0.22	0.32	0.01	1.00	3.13	0.14	0.11	0.00	4.94	0.27	0.32	0.01	0.99	3.79	0.43	0.11	0.00	1779.00	52.00	1775.00	73.00	1860.90	3.10	1750.00	73.00	1771.00	76.00	1743.00	72.00	313.00	144.00	95.10	0.47	5.30
HM-1084-21	4.73	0.08	0.32	0.01	0.90	3.15	0.06	0.11	0.00	4.82	0.14	0.32	0.01	0.76	3.15	0.06	0.11	0.00	1774.00	14.00	1777.00	28.00	1794.00	7.20	1784.00	26.00	1779.00	29.00	1800.00	33.00	48.30	40.40	37.61	0.85	1.00
HM-1084-78	4.75	0.10	0.32	0.01	0.91	3.15	0.06	0.11	0.00	4.82	0.13	0.32	0.01	0.82	3.14	0.06	0.11	0.00	1777.00	18.00	1774.00	28.00	1768.00	9.60	1788.00	24.00	1775.00	29.00	1767.00	25.00	53.40	39.40	35.50	0.73	-0.60
HM-1084-80	4.98	0.08	0.32	0.01	0.99	3.16	0.05	0.11	0.00	4.93	0.12	0.32	0.01	0.96	3.20	0.07	0.11	0.00	1814.00	14.00	1772.00	25.00	1851.90	2.80	1803.00	23.00	1768.00	27.00	1822.00	26.00	291.00	303.00	246.00	1.06	4.40
HM-1084-46	4.16	0.09	0.27	0.01	0.98	3.73	0.07	0.11	0.00	3.46	0.12	0.26	0.01	1.00	3.83	0.08	0.10	0.00	1665.00	17.00	1531.00	26.00	1846.20	3.50	1514.00	27.00	1502.00	27.00	1531.00	26.00	359.00	90.00	42.10	0.26	17.10
HM-1084-24	0.62	0.01	0.08	0.00	0.57	12.70	0.09	0.06	0.00	0.61	0.01	0.08	0.00	0.71	12.76	0.10	0.06	0.00	489.20	3.90	488.80	3.30	501.00	16.00	482.00	5.20	486.90	3.70	461.80	9.70	313.40	192.80	51.50	0.61	1.60
HM-1084-43	0.61	0.01	0.08	0.00	0.66	12.80	0.13	0.06	0.00	0.58	0.01	0.08	0.00	0.70	12.84	0.13	0.06	0.00	484.50	5.20	485.00	4.60	461.00	16.00	467.50	7.50	484.00	4.70	415.00	19.00	77.30	52.30	11.53	0.69	-7.70
HM-1084-44	0.61	0.01	0.08	0.00	0.81	12.80	0.14	0.06	0.00	0.60	0.01	0.08	0.00	0.81	12.81	0.14	0.06	0.00	483.40	5.50	485.00	5.20	462.00	9.80	478.10	6.40	485.10	5.20	446.00	13.00	215.00	118.00	26.00	0.57	-4.80
HM-1084-35	0.61	0.01	0.08	0.00	0.67	12.82	0.16	0.06	0.00	0.59	0.01	0.08	0.00	0.66	12.88	0.17	0.06	0.00	479.60	8.00	483.90	6.00	464.00	19.00	468.00	8.30	483.00	6.00	419.00	24.00	102.30	76.00	15.75	0.77	-6.50
HM-1084-22	0.61	0.01	0.08	0.00	0.85	12.85	0.10	0.06	0.00	0.61	0.01	0.08	0.00	0.78	12.84	0.11	0.06	0.00	482.40	3.40	483.00	3.70	478.20	5.40	483.40	4.10	482.40	3.90	477.00	7.10	788.00	509.00	105.40	0.65	-1.60
HM-1084-47	0.59	0.01	0.08	0.00	0.73	13.20	0.12	0.06	0.00	0.58	0.01	0.08	0.00	0.74	13.24	0.12	0.06	0.00	472.70	4.40	470.50	4.10	466.00	11.00	467.30	5.10	469.50	4.20	446.00	12.00	212.30	170.80	38.41	0.84	-2.50
HM-1084-13	0.59	0.01	0.08	0.00	0.46	13.23	0.13	0.06	0.00	0.56	0.01	0.08	0.00	0.71	13.23	0.14	0.05	0.00	473.20	4.60	470.10	4.50	495.00	17.00	453.00	8.80	468.80	4.80	354.00	40.00	76.80	65.40	15.08	0.88	3.90
HM-1084-11	0.59	0.01	0.08	0.00	0.61	13.26	0.14	0.06	0.00	0.54	0.01	0.08	0.00	0.70	13.33	0.14	0.05	0.00	470.40	7.20	468.70	4.70	475.00	22.00	440.90	7.90	467.00	4.80	313.00	32.00	49.00	37.30	8.15	0.78	-4.90
HM-1084-36	0.59	0.01	0.08	0.00	0.60	13.30	0.11	0.06	0.00	0.58	0.01	0.08	0.00	0.67	13.29	0.10	0.06	0.00	467.50	3.90	467.40	3.70	468.00	10.00	464.40	4.70	467.50	3.60	441.00	15.00	310.00	242.10	51.20	0.82	-0.70
HM-1084-45	0.59	0.01	0.08	0.00	0.68	13.31	0.13	0.06	0.00	0.58	0.01	0.08	0.00	0.75	13.32	0.13	0.06	0.00	467.90	5.00	467.00	4.20	466.00	11.00	464.50	5.60	467.10	4.40	448.00	11.00	186.00	194.00	42.80	1.06	-1.20
HM-1084-75	0.59	0.01	0.08	0.00	0.71	13.31	0.17	0.06	0.00	0.56	0.01	0.07	0.00	0.92	13.45	0.20	0.05	0.00	467.50	3.60	466.80	5.80	485.00	18.00	448.00	8.40	465.00	6.20	356.00	33.00	609.00	223.00	50.80	0.37	0.90
HM-1084-61	0.59	0.01	0.08	0.00	0.81	13.33	0.25	0.06	0.00	0.55	0.02	0.07	0.00	0.76	13.47	0.25	0.05	0.00	469.80	8.00	466.00	8.10	484.00	14.00	442.00	13.00	463.70	8.30	314.00	58.00	250.00	83.00	17.60	0.31	2.90
HM-1084-73	0.58	0.02	0.08	0.00	0.55	13.33	0.27	0.06	0.00	0.52	0.02	0.07	0.00	0.64	13.45	0.27	0.05	0.00	463.00	15.00	465.90	8.70	518.00	36.00	425.00	14.00	461.60	9.40	292.00	77.00	68.00	47.00	10.21	0.71	7.10

	207_235	2SE	206_238	2SE	Error Correlation	238_206	2SE	207_206	2SE	207_235	2SE	206_238	2SE	Error Correlation	238_206	2SE	207_206	2SE	207_235	2SE	206_238	2SE	207_206	2SE	207_235	2SE	206_238	2SE	207_206	2SE	Approx_U_PPM	Approx_Th_PPM	Approx_Pb_PPM	Th/U	Final Disc Percent
HM-1084-56	0.58	0.01	0.07	0.00	0.73	13.39	0.12	0.06	0.00	0.56	0.01	0.07	0.00	0.71	13.45	0.13	0.05	0.00	464.50	4.30	464.30	4.10	456.00	11.00	454.60	7.10	463.10	4.20	404.00	26.00	382.00	225.00	48.70	0.61	-2.90
HM-1084-58	0.58	0.01	0.07	0.00	0.79	13.40	0.15	0.06	0.00	0.57	0.02	0.07	0.00	0.68	13.45	0.16	0.06	0.00	465.20	6.50	464.00	5.20	454.00	12.00	457.00	10.00	462.90	5.30	444.00	13.00	660.00	132.00	27.90	0.21	-2.80
HM-1084-17	0.58	0.01	0.07	0.00	0.83	13.47	0.16	0.06	0.00	0.57	0.01	0.07	0.00	0.88	13.49	0.17	0.06	0.00	462.30	5.30	461.50	5.30	466.00	11.00	461.70	6.40	461.20	5.50	449.00	11.00	194.90	207.10	45.96	1.08	-0.30
HM-1084-90	0.56	0.01	0.07	0.00	0.58	13.64	0.11	0.06	0.00	0.54	0.01	0.07	0.00	0.50	13.65	0.11	0.05	0.00	453.80	5.70	456.20	3.50	464.00	16.00	437.30	7.70	454.90	3.60	337.00	43.00	203.00	138.40	28.30	0.68	-2.40
HM-1084-57	0.57	0.01	0.07	0.00	0.68	13.66	0.26	0.06	0.00	0.57	0.01	0.07	0.00	0.77	13.66	0.25	0.06	0.00	455.70	8.40	455.60	8.20	461.00	12.00	459.60	7.30	455.80	8.20	451.00	12.00	337.90	347.00	80.40	1.06	0.50
HM-1084-87	0.56	0.00	0.07	0.00	0.60	13.72	0.13	0.06	0.00	0.56	0.00	0.07	0.00	0.78	13.72	0.13	0.06	0.00	452.40	2.80	453.70	4.20	455.00	5.70	451.10	2.70	453.60	4.30	452.70	4.90	1202.00	482.00	96.40	0.42	0.90
HM-1084-67	0.56	0.01	0.07	0.00	0.47	13.73	0.15	0.06	0.00	0.51	0.01	0.07	0.00	0.53	13.85	0.15	0.05	0.00	453.80	6.50	453.00	4.80	458.00	18.00	416.70	9.00	449.00	4.60	232.00	43.00	77.80	34.90	7.94	0.44	-2.20
HM-1084-68	0.56	0.01	0.07	0.00	0.80	13.75	0.13	0.06	0.00	0.55	0.01	0.07	0.00	0.80	13.76	0.13	0.06	0.00	451.10	3.80	452.60	4.20	459.90	8.90	447.20	4.70	451.30	4.40	431.00	15.00	719.00	312.00	61.40	0.45	2.20
HM-1084-66	0.56	0.01	0.07	0.00	0.58	13.77	0.16	0.06	0.00	0.52	0.02	0.07	0.00	0.71	13.80	0.16	0.05	0.00	453.90	7.10	451.90	5.00	453.00	20.00	426.00	9.80	451.40	5.30	301.00	42.00	88.80	52.70	12.04	0.61	-2.60
HM-1084-79	0.57	0.01	0.07	0.00	0.57	13.78	0.11	0.06	0.00	0.56	0.01	0.07	0.00	0.75	13.82	0.12	0.06	0.00	455.60	7.90	451.70	3.40	470.00	27.00	452.30	8.00	450.60	3.80	448.00	28.00	441.00	225.50	44.20	0.52	3.40
HM-1084-63	0.56	0.01	0.07	0.00	0.66	13.79	0.15	0.06	0.00	0.56	0.01	0.07	0.00	0.74	13.82	0.15	0.06	0.00	451.20	5.50	451.30	4.70	464.00	15.00	448.70	5.00	451.00	4.70	433.00	11.00	561.00	566.00	110.70	1.04	2.10
HM-1084-83	0.57	0.02	0.07	0.00	0.92	13.79	0.30	0.06	0.00	0.57	0.02	0.07	0.00	0.70	13.82	0.31	0.06	0.00	455.90	9.60	451.30	9.70	456.00	21.00	456.00	14.00	451.30	9.90	468.00	45.00	587.00	402.90	74.10	0.70	0.50
HM-1084-70	0.56	0.01	0.07	0.00	0.87	13.81	0.19	0.06	0.00	0.57	0.02	0.07	0.00	0.81	13.82	0.20	0.06	0.00	450.00	6.60	450.40	6.00	475.00	12.00	454.00	10.00	450.70	6.20	476.00	38.00	921.00	310.00	60.70	0.35	4.70
HM-1084-52	0.58	0.01	0.07	0.00	0.64	13.85	0.12	0.06	0.00	0.55	0.01	0.07	0.00	0.73	13.90	0.13	0.06	0.00	462.80	4.10	449.30	3.70	471.00	11.00	447.30	6.20	448.50	3.90	423.00	20.00	269.00	176.00	37.10	0.68	3.30
HM-1084-95	0.56	0.01	0.07	0.00	0.68	13.88	0.19	0.06	0.00	0.51	0.02	0.07	0.00	0.74	13.99	0.21	0.05	0.00	452.00	5.50	448.40	5.80	464.00	17.00	415.00	16.00	445.70	6.40	246.00	92.00	471.00	220.00	42.90	0.48	3.50
HM-1084-72	0.55	0.01	0.07	0.00	0.83	13.89	0.18	0.06	0.00	0.56	0.01	0.07	0.00	0.54	13.90	0.18	0.06	0.00	447.20	6.70	448.20	5.70	484.00	11.00	448.30	6.90	448.10	5.60	460.00	31.00	789.00	356.00	67.50	0.46	7.20
HM-1084-53	0.57	0.01	0.07	0.00	0.80	13.91	0.18	0.06	0.00	0.54	0.01	0.07	0.00	0.60	13.99	0.18	0.06	0.00	459.40	6.30	447.60	5.60	485.00	13.00	439.60	9.60	445.80	5.60	420.00	40.00	422.00	296.00	58.00	0.74	8.20
HM-1084-54	0.57	0.01	0.07	0.00	0.60	13.91	0.11	0.06	0.00	0.54	0.01	0.07	0.00	0.75	13.99	0.11	0.05	0.00	459.40	3.70	447.60	3.30	466.20	9.90	438.60	7.10	445.80	3.50	347.00	33.00	231.00	132.10	29.40	0.59	3.60
HM-1084-100	0.56	0.01	0.07	0.00	0.75	13.92	0.17	0.06	0.00	0.51	0.02	0.07	0.00	0.69	14.02	0.18	0.05	0.00	448.80	6.00	447.10	5.20	468.00	16.00	415.00	15.00	444.50	5.40	248.00	80.00	556.00	224.70	43.60	0.42	3.90
HM-1084-96	0.55	0.01	0.07	0.00	0.77	13.94	0.17	0.06	0.00	0.52	0.02	0.07	0.00	0.62	14.01	0.17	0.05	0.00	446.50	6.80	446.70	5.20	469.00	16.00	424.00	12.00	444.90	5.30	315.00	69.00	232.00	97.80	19.55	0.43	5.10
HM-1084-93	0.55	0.01	0.07	0.00	0.62	13.98	0.13	0.06	0.00	0.52	0.01	0.07	0.00	0.62	14.02	0.14	0.05	0.00	444.60	5.40	445.30	4.00	451.00	16.00	426.00	6.80	444.40	4.10	333.00	36.00	155.70	135.50	27.04	0.84	-0.50
HM-1084-91	0.55	0.01	0.07	0.00	0.63	14.01	0.13	0.06	0.00	0.53	0.01	0.07	0.00	0.66	14.07	0.13	0.05	0.00	445.80	5.50	444.50	4.00	445.00	17.00	429.70	7.10	443.20	4.00	380.00	22.00	125.90	121.60	27.41	0.97	0.30
